# Atmosphere Effects on Arene Reduction with Lithium and Ethylenediamine in THF

**DOI:** 10.1021/acs.joc.4c03118

**Published:** 2025-03-03

**Authors:** Zachary S. Shellnutt, Kazunori Koide

**Affiliations:** Department of Chemistry, University of Pittsburgh, 219 Parkman Avenue, Pittsburgh, Pennsylvania 15260, United States

## Abstract

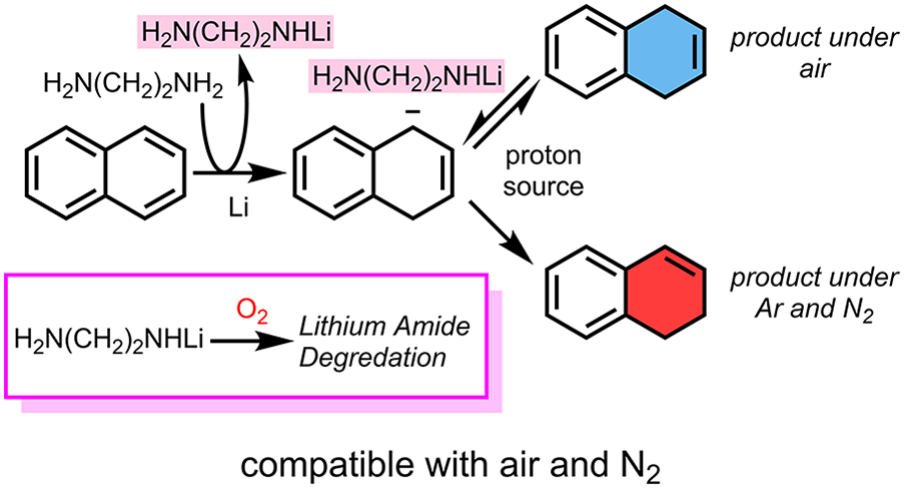

Birch reductions employing lithium metal have been performed mostly under argon due to concerns about forming metal nitrides from the reduction of dinitrogen if performed under nitrogen. Although it is generally understood that inert atmospheres are standard for Birch and Birch-type (lithium, ethylenediamine, *t*-BuOH, THF) reductions, the atmosphere effect on Birch reduction has not been studied. Herein, we report the reduction of model substrates using lithium metal and ethylenediamine in THF under various atmospheric conditions. The reductions under argon and nitrogen atmospheres afforded essentially the same yields. Surprisingly, oxygen not only perturbed the yields in some cases but also controlled regioselectivity for a subset of naphthalenes. We propose a mechanism underlying the unexpected oxygen-dependent regioselectivity for the Birch-type reduction of naphthalenes. This work shows that the Birch-type reduction may be performed under a nitrogen atmosphere and may account for a fraction of oxygen-sensitive Birch-type reductions.

## Introduction

**Figure 1:**
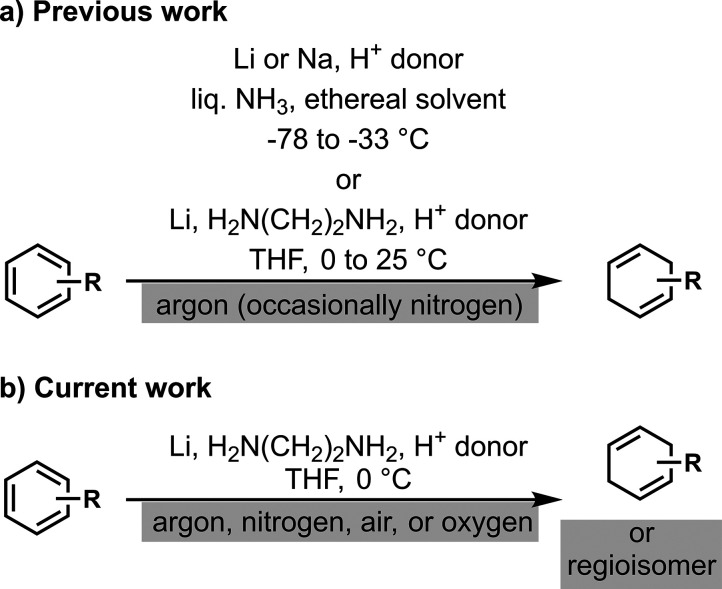
Previous work and current work. (a) Previous Birch reduction reactions were performed under argon or, occasionally, nitrogen atmosphere. Concerns regarding nitride formation were not experimentally addressed. (b) Current work shows that Birch-type reduction can be employed under an argon, nitrogen, air, or oxygen atmosphere. In the presence of oxygen, regioisomers may be produced from naphthalenes.

The Birch reduction converts arenes to the corresponding 1,4-cyclohexadienes in the presence of lithium, sodium, or potassium in liquid ammonia (Figure 1a).^1,2^ In general, dissolving metal reductions are performed under an argon (Ar) atmosphere^3,4^ instead of a nitrogen (N_2_) atmosphere due to concerns about lithium reacting with N_2_ to form lithium nitride (Li_3_N),^5,6^ a flammable and explosive base.^6^ To our knowledge, there is one report on an industrial accident regarding the formation of Li_3_N in the literature.^7^ Nonetheless, it should be noted that in the energy sector, the reduction of N_2_ to NH_3_ is desirable and has been actively studied.^8^

In addition to the safety concerns with nitrogen, argon is about five times as expensive as nitrogen. Moreover, the need for argon would limit the utility of Birch reductions in the chemical and pharmaceutical industry because the industrial infrastructure is often developed for a nitrogen atmosphere (*e.g*., Merck,^9^ Novartis,^10^ Eli Lilly^11^). Also, the lithium-di-*t*-butyl biphenyl-mediated reduction method is particularly sensitive to oxygen.^12^ Altogether, the Birch reduction continues to encounter two unfavorable needs: liquid ammonia and argon (although Pfizer recently used nitrogen for the Birch reduction^13^).

In 2021, our laboratory reported a Birch-type reduction method that employed lithium, ethylenediamine, and *t*-BuOH in tetrahydrofuran (THF),^14^ which has been used by various groups.^15−22^ This method omits ammonia (for other ammonia-free Birch reduction methods, see the cited references^23−34^), mitigating one of the challenges; however, the significance of its reaction atmosphere was not addressed (we note that the Sugai group reported the detrimental effect of oxygen for the O-demethylation of anisole derivatives under similar conditions^35^). Therefore, although the reduction of dinitrogen with lithium is notoriously slow^36^ and intermediates of the dinitrogen reduction in trace amounts likely undergo proton exchange with ethylenediamine or *t*-BuOH not to form highly negatively charged nitrogen species, we decided to analyze the impact of atmospheres on reaction outcomes (we acknowledge that the Ito group has reported similar conditions under an ambient atmosphere mechanochemically^32^). In this manuscript, we report the first systematic study for the Birch-type reduction using lithium, ethylenediamine, and optionally *t*-BuOH, in THF to compare the yields under argon, nitrogen, air, and oxygen atmospheres (Figure 1b). This study shows that our Birch-type reduction is compatible with nitrogen and often with air, and its chemoselectivity may change under an air or oxygen atmosphere.

## Results and Discussion

To begin the study, we tested our previously reported Birch-type condition on a series of mono- and disubstituted benzenes **1**–**7** and naphthalene (**8**) under various atmospheres (Table 1). Although the reduction of naphthalene would require twice the reagents for full tetrahydro-reduction, we decided to use the same condition for monoarenes and **8**, because its reduction efficacy could still be determined from the population of the reduction products. The reductions under argon and nitrogen atmospheres were near quantitative conversion with the exception of **8**, as over-reduction exhausted the reductant before the full conversion of naphthalene to the dihydro product. There was no distinguishable difference between an argon and nitrogen atmosphere, both by color and chemical yield. Interestingly, the reductions under an air atmosphere for benzenes **1**–**7** showed high conversions. The reduction of benzenes **1**–**7** under an atmosphere of oxygen led to lower conversions. Both monoalkyl- and monoalkoxybenzenes **1**, **4** and **5** showed higher conversions compared to their respective disubstituted systems. Similarly, the alkoxy-substituted benzenes had better reactivity then the alkyl-substituted benzenes, which could be attributed to their relative rates of reduction.^37^ All reductions yielded primarily the anticipated 1,4-dihydro system with respect to their aromatic precursor. These results show the robust nature and high efficiency of the lithium-ethylenediamine-THF system even under an air atmosphere. However, under both an air and oxygen atmosphere, the conversion of **8** could not be determined due to coelution of an unexpected byproduct not seen under an argon or nitrogen atmosphere.

**Table 1:**
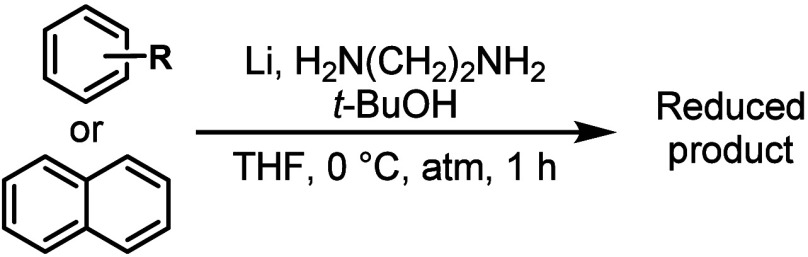

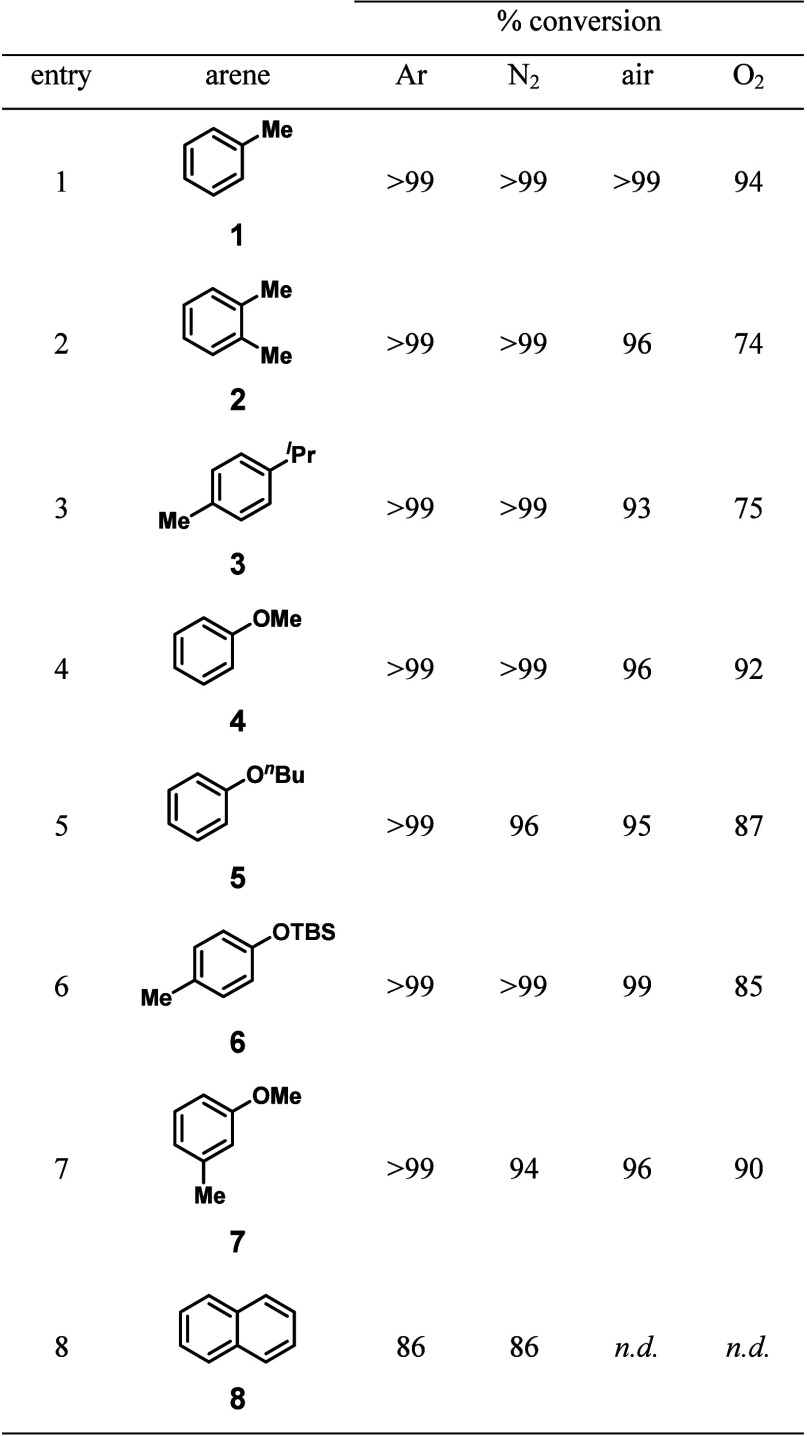
Arene Reduction under Various Atmospheres^a^

a Reaction condition: arene (2.5 mmol), lithium (7.5 mmol), ethylenediamine (15 mmol), *t*-BuOH (6.3 mmol), THF (8.3 mL), 0 °C, indicated atmosphere (1 atm), 1 h. Percent conversion was determined by GC-MS comparison of remaining starting material to 1-methoxyadamantane as an internal standard. The reduction of **8** under air and oxygen could not be accurately analyzed due to the coelution of an unknown reduction isomer with the starting material. *n.d.* = not determined.

The de Vlieger and Rabideau groups previously analyzed the reaction crude mixtures of the reduction of **8** with lithium in liquid ammonia and diethyl ether at −33 °C and determined the kinetic product **9** as the minor product and thermodynamic product **10** as the major product.^38,39^ Our experiment under an argon atmosphere afforded **10** as the major dihydro product (Table 2, Ar). This observation is similar compared to the results from de Vlieger and Rabideau.^38,39^ Furthermore, the selectivity for the dihydro products is reversed for **9** under an air and oxygen atmosphere. This would suggest that either 1) the second protonation step to form the dihydro species is influenced by the atmosphere or 2) there is a strong base generated in situ that is isomerizing **9** to thermodynamically more favorable **10** and is being inhibited under an oxygen-containing atmosphere.

**Table 2:**
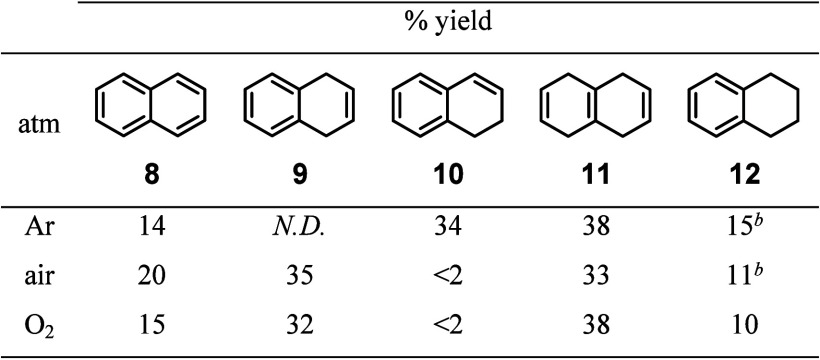
Product Distribution of Naphthalene (**8**) Reduction under Various Atmospheres^a^

a Reaction condition: **8** (2.5 mmol), lithium (7.5 mmol), ethylenediamine (15 mmol), *t*-BuOH (6.3 mmol), THF (8.3 mL), 0 °C, indicated atmosphere (1 atm), 1 h. Percent yields were determined by ^1^H NMR comparison to 1-methoxyadamantane as an internal standard.

b Percent yield determined by ^1^H NMR may not be accurate due to peak overlaps. *N.D*. = not detected.

**Figure 2:**
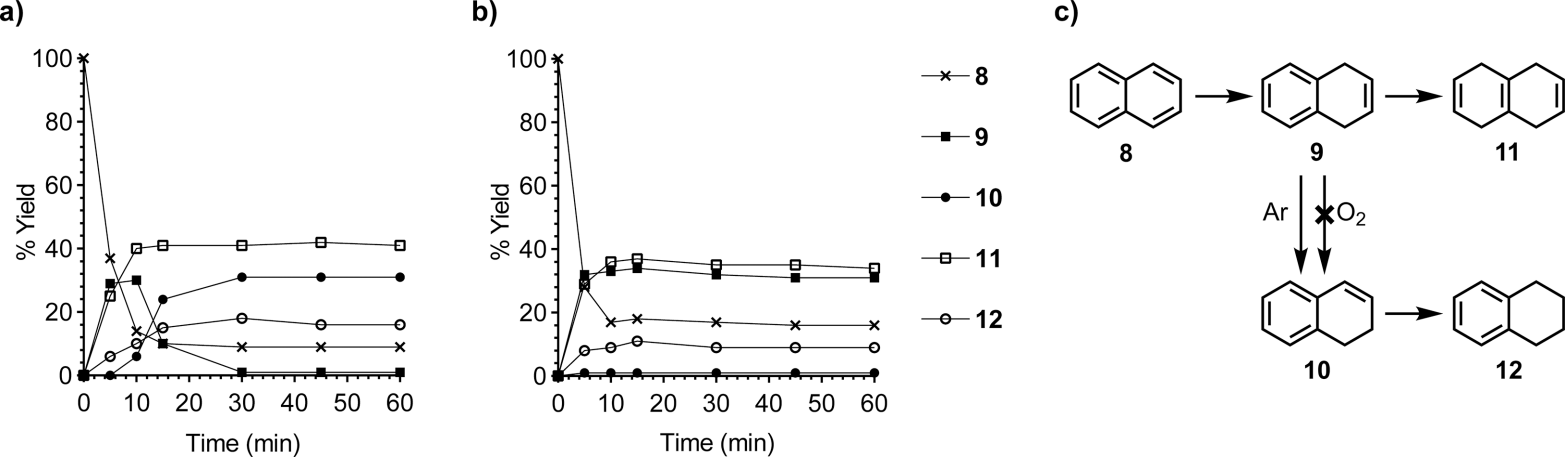
Reduction of naphthalene (**8**) measured over time. Reaction condition: **8** (3.0 mmol), lithium (9.0 mmol), ethylenediamine (18 mmol), *t*-BuOH (7.5 mmol), THF (10 mL), 0 °C, indicated atmosphere (1 atm), 1 h. Aliquots (0.1 mL) of the reaction mixture were taken at 0, 5, 10, 15, 30, 45, and 60 min for analysis. (a) Reduction of **8** under an argon atmosphere. (b) Reduction of **8** under an oxygen atmosphere. Percent yields were determined by ^1^H NMR using 1-methoxyadamantane as an internal standard. (c) The reduction of naphthalene with two competing pathways.

To further investigate the naphthalene system, we monitored its reduction over time. The reaction proceeded rapidly and with selectivity for the kinetic products **9** and **11** under an argon atmosphere (Figure 2a). In this experiment, at approximately 10 min, **9** began to decrease while **10** increased, suggesting an isomerization step, as discussed above, that competed with the reduction of **9** to **11**. In contrast, under an oxygen atmosphere, the isomerization of **9** did not occur (Figure 2b). This suggests that the reaction initially behaved like a traditional Birch reduction, favoring the kinetic 1,4-dihydro product **9**, and a putative strong base generated in situ isomerized **9** to **10** via deprotonation-protonation. This isomerization has been studied under traditional Birch conditions, which showed that LiNH_2_ can perform the deprotonation^38,39^ but alkoxides cannot.^40^

To better understand the reaction pathway, we used a sub- and superstoichiometric amount of reductant (Table 3). With two equivalents of lithium, there was no significant difference between argon and oxygen in product distribution with clear selectivity to 1,4-dihydronaphthalene (**9**, entries 1 and 2). Five equivalents of lithium made a significant difference between argon and oxygen; under argon, kinetic product **11** and thermodynamic products **12** and **13** were formed in 27, 31, and 21% yield, respectively (entry 3). In contrast, under oxygen, the reaction afforded the kinetic products **9** and **11** and the thermodynamic products **12** and **13** in 9, 51, 10, and 13% yield, respectively (entry 4). These data corroborated the kinetic plots (Figure 2), indicating that the 1,2-dihydronaphthalene (**10**) was the product of the isomerization of 1,4-dihydronaphthalene (**9**).

**Table 3:**
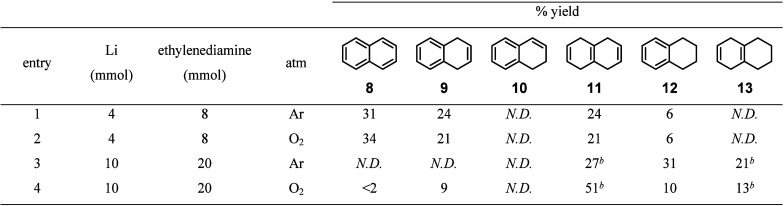
Reduction of Naphthalene (**8**) Using Sub- and Superstoichiometric Amounts of Reductant^a^

a Reaction condition: **8** (2.0 mmol), lithium (see table), ethylenediamine (see table), *t*-BuOH (5.0 mmol), THF (6.7 mL), 0 °C, indicated atmosphere (1 atm), 1 h. Percent yields were determined by ^1^H NMR comparison to 1-methoxyadamantane as an internal standard.

b Percent yield determined by ^1^H NMR may not be accurate due to peak overlaps. *N.D*. = not detected.

We next elucidated oxygen’s impact on the reduction outcome. Under traditional Birch conditions, lithium can react with oxygen to form LiO_2_, which will break down into LiOH and LiOH·H_2_O in the presence of NH_3_.^41^ Superoxide formation from lithium and oxygen^42^ has been hypothesized to accelerate traditional Birch reductions, as will be further discussed below. To test the effect of superoxides, it would be ideal to use LiO_2_. However, LiO_2_ is nontrivial to produce and is known to be unstable.^41,43−45^ Therefore, KO_2_ was used as a commercially available alternative, although it proved to be poorly soluble under our reaction conditions. When adding less than two equivalents of KO_2_ to the reaction mixture, no significant prevention of isomerization was observed (Table 4, entries 1–5 vs Table 2, Ar). When five equivalents were added (entry 6), a noticeable amount of **9** was observed. This result does not necessarily indicate superoxide’s reactivity because protic additives, such as *t*-BuOH and ethylenediamine, can cause the degradation of superoxide species.^44^ If LiO_2_ degrades under the lithium-ethylenediamine-THF condition, then the species of interest could be O_2_ generated from superoxide oxidation/degradation. This notion is supported by entry 7, in which the THF was purged with oxygen prior to use, and the reduction reaction afforded the similar amount of **9** as seen in Table 2 under aerobic conditions. Similarly, when 1,4-dihydronaphthalene **9** and 1,2-dihydronaphthalene **10** were separately subjected to the same conditions as entry 6 without lithium, there was no change, suggesting that superoxide was not involved in isomerizing **9** to **10** (see the Experimental Section and Spectrum S44 and Spectrum S45 for the ^1^H NMR spectra in the Supporting Information). A control experiment to observe the formation of LiO_2_ in situ under relevant reaction conditions using a UV spectrometer was unsuccessful, as expected for the instability of LiO_2_ known in the literature.^44^ However, we could observe a color change, presumably due to an unstable electride complex (Figure S1 (page S11) in the Supporting Information). The formation of the presumable electride complex in the presence of oxygen may suggest that the lithium-ethylenediamine-THF condition does not form LiO_2_ under an oxygen atmosphere.

**Table 4:**
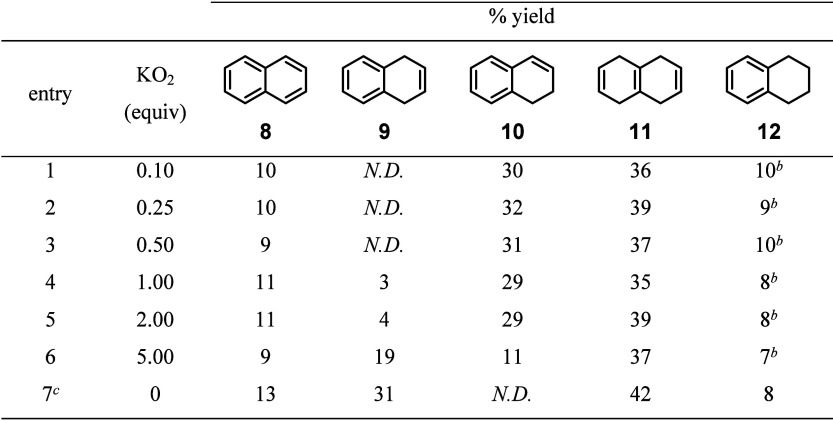
Reduction of Naphthalene (**8**) in the Presence of KO_2_^a^

a Reaction condition: **8** (2.0 mmol), lithium (6.0 mmol), ethylenediamine (12 mmol), *t*-BuOH (5.0 mmol), KO_2_ (see table), THF (6.7 mL), 0 °C, argon (1 atm), 1 h. Percent yields were determined by ^1^H NMR comparison to 1-methoxyadamantane as an internal standard.

b Percent yield determined by ^1^H NMR may not be accurate due to peak overlaps.

c THF was saturated with O_2_. *N.D*. = not detected.

**Scheme 1:**
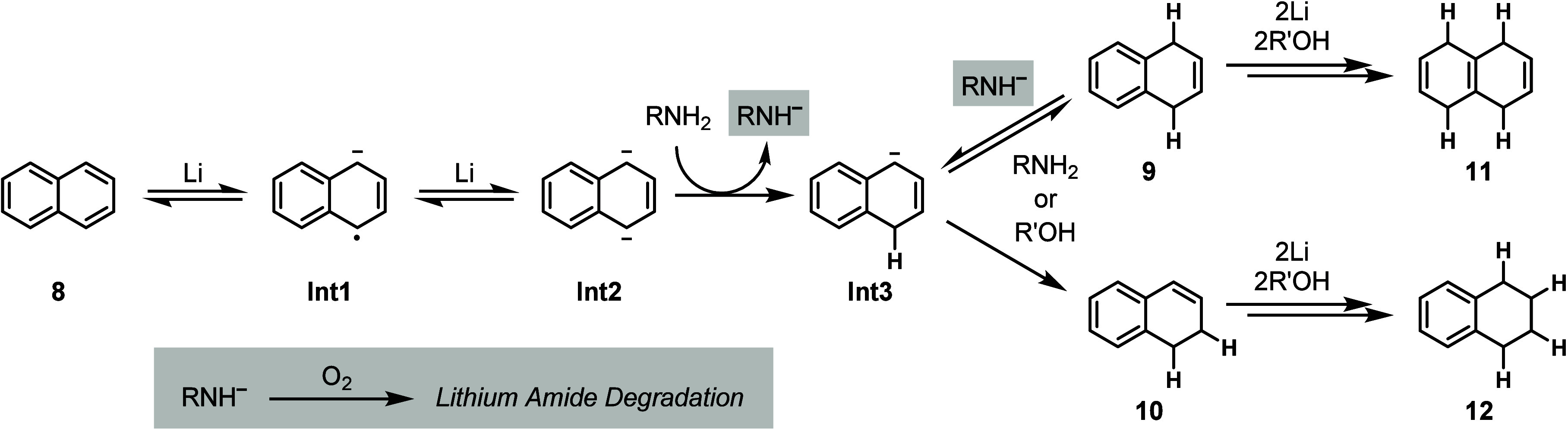
Reduction Pathway for Naphthalene (**8**) under Lithium-Ethylenediamine-THF Condition^,^ R = −(CH_2_)_2_NH_2_ under lithium-ethylenediamine-THF condition. R′ = *t*-Bu under lithium-ethylenediamine-THF condition.

Having performed the kinetic studies and control experiments, we hypothesized that the reduction under our conditions proceeded through a traditional Birch mechanism in which two subsequent electron transfers toward **8** form highly basic dianion **Int2** (Scheme 1). This dianion is initially protonated by *t*-BuOH to form monoanion **Int3**. Similarly, it is possible to protonate radical anion **Int1** then reduce to monoanion **Int3** (pathway not shown). Monoanion **Int3** undergoes a kinetic proton transfer to form **9**. This product then reacts like a 1,2-dialkylbenzene and is further reduced to 1,4,5,8-tetrahydronaphthalene **11**. As a minor pathway, monoanion **Int3** can be protonated thermodynamically to form **10** that, in the presence of excess reductant, is reduced to 1,2,3,4-tetrahydronaphthalene **12**. Once *t*-BuOH has been consumed, dianion **Int2** is protonated by ethylenediamine to form **Int3** and the respective lithium amide (highlighted with a gray box). This strong base is proposed to be the isomerizing species to deprotonate **9** en route to **10**. It is plausible that both proton sources are being deprotonated by dianion **Int2**, as the reduction of **8** is more favorable than benzene and, therefore, does not need a proton source to shift the equilibrium from **Int1** to **Int2**.^46^ However, under oxygen, the lithium amide is degraded via an oxidation pathway, removing the deprotonating agent for isomerization (see Figure S2 (page S12) in the Supporting Information).

**Figure 3:**
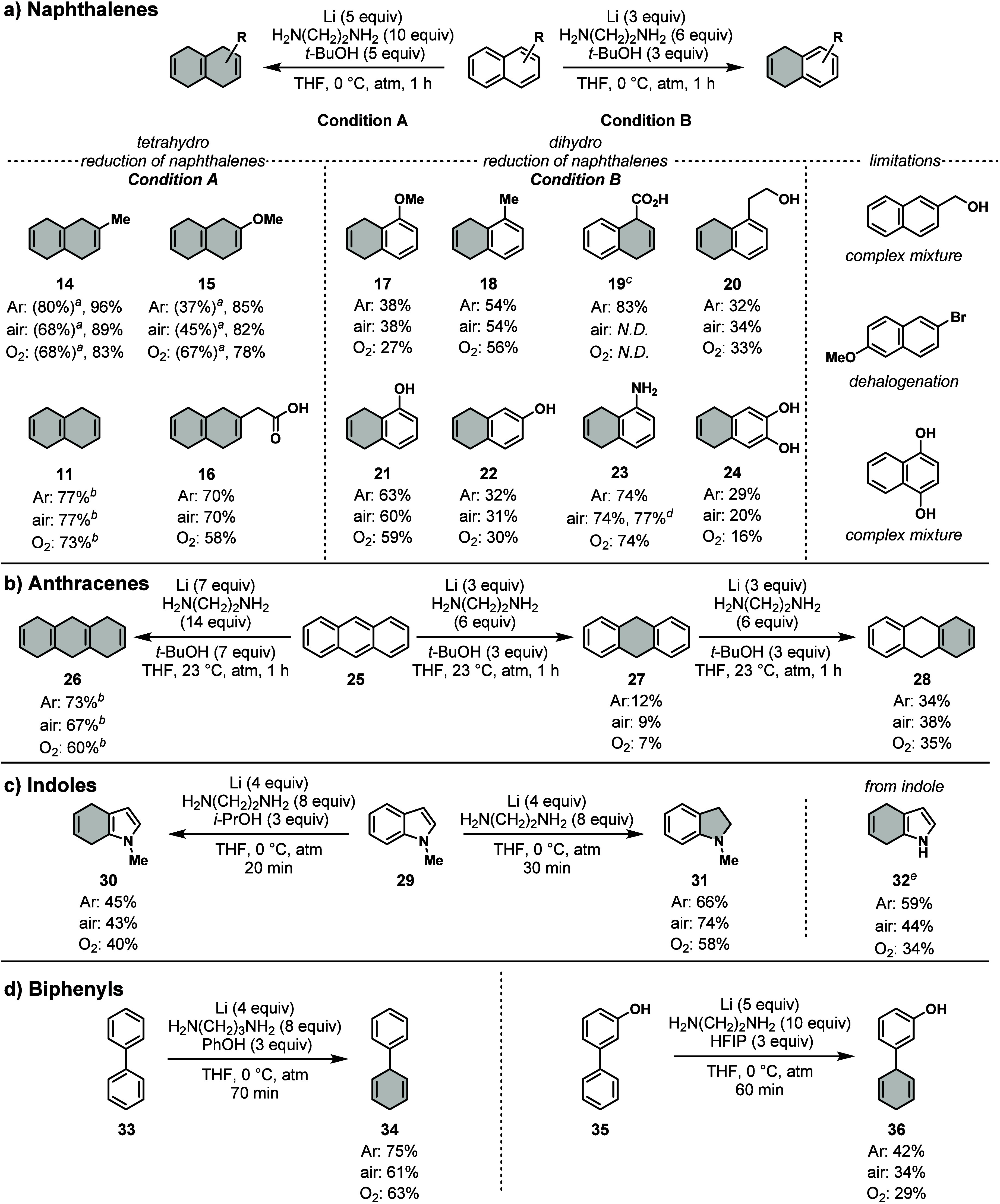
Substrate scope of polyaromatic reduction under alternative atmospheres. Reaction condition A: arene (2.0 mmol), lithium (10 mmol), ethylenediamine (20 mmol), *t*-BuOH (10 mmol), THF (6.7 mL), 0 °C, atmosphere (1 atm), 1 h. Reaction condition B: arene (2.0 mmol), lithium (6.0 mmol), ethylenediamine (12 mmol), *t*-BuOH (6.0 mmol), THF (6.7 mL), 0 °C, atmosphere (1 atm), 1 h. ^*a*^Following condition A using 6.0 mmol of *t*-BuOH. ^*b*^Percent yield determined by ^1^H NMR may not be accurate due to peak overlaps. ^*c*^*t*-BuOH was not used. ^*d*^Reduction was performed on a 20 mmol scale. ^*e*^Using the same condition as **30**. Percent yields were determined by ^1^H NMR comparison to 1-methoxyadamantane or 1,3,5-trimethoxybenzene as an internal standard. For more details, see the Supporting Information.

Finally, we tested substituted naphthalenes to determine whether other substrates besides **8** respond to an oxygen atmosphere. After pilot studies, the reduced products of substituted naphthalenes could be grouped into tetrahydronaphthalenes and dihydronaphthalenes (Figure 3a left and middle). Under modified condition A (5 equiv lithium, 10 equiv ethylenediamine, 3 equiv *t*-BuOH, THF, 0 °C, 1 h^14^), 2-methylnaphthalene was reduced to tetrahydronaphthalene **14** in 80, 68, and 68% yield under argon, air, and oxygen, respectively (Figure 3a, yields in parentheses). In contrast, the reduction of 2-methoxynapththalene afforded **15** in a reverse trend (from 37% to 67% yield with argon and oxygen). The GC-MS data showed that both substrates underwent isomerization, which was partially prevented under an oxygen atmosphere (Figures S3a and S3c in the Supporting Information).

To minimize the isomerization from 1,4-dihydronaphthalenes to 1,2-dihydronaphthalenes (*e*.*g*., **9** to **10**, Scheme 1), we hypothesized that increasing the proton donor amount would quench the lithium amide generated in situ, promoting the reaction between the speculated in situ generated LiO_2_ and arene described by Thompson and Kleinberg.^42^ With five equivalents of *t*-BuOH (condition A), the yields under argon increased to 95 and 85%, respectively, for these substrates and slightly diminished yields (83 and 78%) under oxygen. Interestingly, demethoxylation during the reduction of 2-methoxynaphthalene was largely avoided under an oxygen atmosphere, suggesting that the demethoxylation was promoted by a base (most likely lithium amide). Using five equivalents of *t*-BuOH improved chemoselectivity toward **14** and **15** and largely suppressed elimination or isomerization (Figures S3b and S3d in the Supporting Information). This would also suggest that the reduction outcome is independent of humidity and more on the proton donor equivalents (see Table 2 vs Figure 3).

With these results, we next wanted to test other substrates under condition A. Tetrahydro products **11** and **16** were formed with similar efficiency with decreasing yields with more oxygen and no indication of isomerization. The dihydro reduction of 1-methoxy- and 1-methylnaphthalene under condition B (3 equiv lithium, 6 equiv ethylenediamine, 3 equiv *t*-BuOH, THF, 0 °C, 1 h) was accompanied by a competitive pathway for over-reduction, resulting in 27–38% and 54–56% yields of **17** and **18** under all atmospheres (Figure 3a, middle). The reduction of 1-methoxynaphthalene also showed the loss of the methoxy group, which has been observed under traditional Birch reductions.^6^ Although the reduction of 1-naphthoic acid generated **19** in 83% yield under argon, that under air or oxygen was unsuccessful, yielding mainly the starting material. This result may be attributed to the sensitivity of the anionic intermediates to oxidative rearomatization.

1-Naphthaleneethanol was reduced to **20** in 32–34% yields under argon, air, and oxygen. Likewise, the reductions of electron-rich substrates, 1-hydroxynaphthalene, 2-hydroxyaphthalene, 1-aminonaphthalene, and 2,3-dihydroxynaphthalene, did not yield an observable effect under an O_2_ atmosphere, affording **21**, **22**, **23**, and **24** in 16–74% yields. Previous large-scale reductions of 1-aminonaphthalene have been done electrochemically as it is an intermediate en route to the API Ropinirole.^53^ Gratifyingly, using our conditions under an air atmosphere on a 20 mmol scale proved consistent with the smaller scales. The reduction of anthracene (**25**, Figure 3b) to hexahydro product **26** showed 60–73% yields with minor over-reduction and a mild negative impact of oxygen. The reduction of **25** to the dihydro product **27** with less reagents was inefficient (7–12% yields). Reducing 9,10-dihydroanthracene (**27**) to tetrahydro product **28** with 3 equiv lithium, 6 equiv ethylenediamine, and 3 equiv *t*-BuOH also showed low yields, ranging from 34 to 38%, with no observable effect of oxygen.

In a separate study, our group discovered that the reduction of indoles is most effective with lithium and ethylenediamine with or without *i*-PrOH (manuscript in preparation). In the current study, we wished to determine the impact of atmospheres. In effect, the reduction of 1-methylindole (**29**) to the 4,7-dihydro product **30** did not show any change with oxygen (Figure 3c). However, the reduction to indoline **31** without *i*-PrOH illustrated an improved yield under an air atmosphere, which was confirmed to be reproducible within 5% margin of error. Interestingly, 1*H*-indole was reduced to **32** in 34–59% yields with a heightened sensitivity to oxygen content than other substrates. Similarly, under the optimized conditions for biaryls (manuscript in preparation), the reduction of biphenyl (**33**) and 3-phenylphenol (**35**) showed lower yields in the presence of oxygen (Figure 3d).

## Conclusion

In summary, the Birch-type reductions for monoarenes under lithium-ethylenediamine-THF conditions are robust and tolerant to aerobic conditions. On the contrary, the method with polyaromatic substrates can be sensitive to the reaction atmosphere and the stoichiometry of *t*-BuOH. An important distinction from the traditional mechanism is that oxygen reacts with the in situ-generated lithium amide, perturbing the propensity for isomerization of the kinetic product. The current work did not show conclusive experimental evidence for improving reduction efficiency promoted by in situ-generated superoxide species. The headspace is likely filled with ammonia vapor under the traditional Birch conditions and with the atmosphere of choice under the lithium-ethylenediamine-THF conditions. Therefore, it is important to choose the atmosphere carefully if our method is employed.

## Experimental Section

**Caution!***Reactions performed with THF in the presence of oxygen are not typical, as the formation of peroxides is of concern. Similarly, quenching alkali metals in the presence of oxygen is to be taken slowly, in a functioning fume hood, at lower temperatures, and away from any flame source to avoid combustion from the evolved hydrogen gas*.

### General Procedures

All reagents used were purchased from commercial suppliers and used as provided. All the flasks used to carry out reactions were dried in an oven at 100 °C prior to use. Unless otherwise stated, all reactions that required heating used an oil bath as the heating source, with a thermometer submerged in the bath to monitor the temperature. Unless specifically stated, the temperature of a water bath during the evaporation of organic solvents using a rotary evaporator was about 35 ± 5 °C. THF was distilled over Na metal and benzophenone. Li metal was used in granule form and cut into approximately 2 mm pieces and stored under argon in a desiccator with Drierite. Isolated yields refer to chromatographically and spectroscopically (^1^H NMR) homogeneous materials unless otherwise stated. NMR yields refer to quantitative ^1^H NMR of the crude reaction mixture after workup using 1-methoxyadamantane or 1,3,5-trimethoxybenzene as an internal standard and D1 time set to 15 s. All reactions were monitored by thin-layer chromatography (TLC) carried out on 0.25 mm Merck silica gel plates (60F-254) using either UV light (254 nm) for visualization or anisaldehyde in ethanol, 0.2% ninhydrin in ethanol, or KMnO_4_ in water as the developing agent and heat for visualization. Silica gel (230–400 mesh) was used for flash column chromatography. NMR spectra were recorded on a Bruker ADVANCE spectrometer at 300, 400, or 500 MHz and processed using TopSpin 3.6.5 (Bruker) software. The chemical shifts are given in parts per million (ppm) on a delta (δ) scale. The solvent peak was used as a reference value for ^1^H NMR: C*H*Cl_3_ = 7.26 ppm, for ^13^C NMR: CDCl_3_ = 77.16 ppm. The following abbreviations are used to indicate the multiplicities: s = singlet; d = doublet; t = triplet; q = quartet; m = multiplet; br = broad. GC-MS chromatograms were recorded on a Shimadzu GC-2010 gas chromatograph fitted with a Shimadzu SH-Rxi-5Sil MS (L = 30 m, ID = 0.25, DF = 0.25) column and AOC-6000 autosampler in sequence with a Shimadzu GCMS-QP2010S mass spectrometer with an EI source. Collected data were processed using GC-MS solution 4.45 (Shimadzu) software. As Birch and Nadamuni stated (“Although the detector (of GC) was not calibrated for each compound individually, the probable error is not considered to be greater than ±5% of the estimated value”,^47^ we use the integration of the starting material compared to 1-methoxyadamantane or 1,3,5-trimethoxybenzene as the internal standard, to estimate reaction efficiency over time.

Percent conversion by GC-MS was calculated from the integration of the starting material relative to the integration of the internal standard, measured at 0 (initial, (i) and 60 min (final, f) as shown below in eq 1:
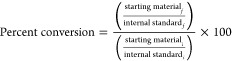
1

Structural determination of peaks from GC-MS experimentation was determined by comparing the mass spectra to NIST11 and NIST11s databases. Structures were assumed when the subtraction of the experiment peak mass spectra from the literature spectra gave a similarity score of ≥93% and were logical from a typical Birch reduction pattern. Structures that could not be unambiguously predicted from the reduction pattern or did not have matching similarities were not assumed.

GC-MS method A:

Column: Shimadzu SH-Rxi-5Sil MS (L = 30 m, ID = 0.25, DF = 0.25).

Elution condition: 35 °C hold for 5 min, 35 to 155 °C over 60 min, linear gradient at 2 °C/min. Data collection begins at 6 or 7 min due to GC-MS solvent cutoff time.

GC-MS method B:

Column: Shimadzu SH-Rxi-5Sil MS (L = 30 m, ID = 0.25, DF = 0.25).

Elution condition: 35 °C hold for 8 min, 35 to 200 °C over 17 min, linear gradient at 10 °C/min, 200 to 250 °C over 3 min, linear gradient at 15 °C/min, 250 °C hold for 5 min. Data collection begins at 7 min due to GC-MS solvent cut off time.

GC-MS method C:

Column: Shimadzu SH-Rxi-5Sil MS (L = 30 m, ID = 0.25, DF = 0.25).

Elution condition: 35 °C hold for 5 min, 35 to 200 °C over 85 min, linear gradient at 2 °C/min. Data collection begins at 7 min due to GC-MS solvent cutoff time.

NMR figures reported in this body of work, after processing, were exported as a PNG file and imported into PowerPoint version 2411 build 16.0.18227.20002 (Microsoft) for labeling. GC-MS figures reported in this body of work, after processing, were exported as a txt file and imported into Excel version 2411 build 16.0.18227.20002 (Microsoft) for organization before being transferred to GraphPad Prism 9.5.0 to make the figures. These figures were then imported into PowerPoint (see above) for organizing and labeling.

### Specific Procedures

#### Preparation of 1-Methoxyadamantane as an Internal Standard

A single-neck, 500 mL round-bottom flask was equipped with a magnetic stir bar and a septum fitted with a needle connected to a nitrogen inlet. THF (50 mL) and NaH (3.94 g, 98.5 mmol, 60% wt. in mineral oil, 1.5 equiv) were added to the flask, and the resulting suspension was cooled to 0 °C under nitrogen. Next, 1-adamantanol (10.0 g, 65.7 mmol) and MeI (16.3 mL, 263 mmol, 4.0 equiv) were dissolved in THF (200 mL) in an addition funnel, and the resulting solution was added to the suspension of NaH dropwise over 1 h. The resulting solution was slowly warmed to 23 °C and stirred for 24 h. The reaction mixture was quenched with water (30 mL) and stirred until the remaining NaH was destroyed. The solvent was evaporated *in vacuo* and extracted with Et_2_O (70 mL × 2). The combined organic layers were washed with water and brine, dried over Na_2_SO_4_, filtered through cotton, and concentrated *in vacuo* to afford the crude reaction mixture. The resulting residue was purified by vacuum distillation (10 mmHg, bp 54 °C) to give 1-methoxyadamantane (9.83 g, 90% yield) as a colorless oil.

1-Methoxyadamantane: ^1^H NMR (300 MHz, CDCl_3_, 298 K) δ 3.23 (s, 3 H), 2.15 (br s, 3 H), 1.73 (d, *J* = 2.6 Hz, 6 H), 1.68–1.56 (m, 6 H). The spectroscopic data matched those in the literature.^48^

#### Preparation of *t*-Butyldimethyl(*p*-tolyloxy)silane (**6**)

A single neck, 100 mL round-bottom flask was equipped with a magnetic stir bar and a septum fitted with a needle connected to a nitrogen inlet. 4-Methylphenol (2.00 g, 18.5 mmol), imidazole (3.80 g, 55.5 mmol, 3.0 equiv), TBSCl (3.50 g, 23.1 mmol, 1.25 equiv), and DMF (15 mL) were added to the flask at 23 °C and allowed to stir at this temperature overnight. The reaction mixture was diluted with water and extracted with hexanes (20 mL × 2). The combined organic layers were washed with water and brine, dried over Na_2_SO_4_, filtered through cotton, and concentrated *in vacuo* to give *t*-butyldimethyl(*p*-tolyloxy)silane (4.23 g, quant. yield) as a colorless oil with no starting material, as confirmed by ^1^H NMR.

*t*-Butyldimethyl(*p*-tolyloxy)silane: ^1^H NMR (400 MHz, CDCl_3_, 298 K) δ 7.02 (d, 2H, *J* = 8.0 Hz), 6.73 (d, 2H, J = 8.0 Hz), 2.27 (s, 3H), 0.97 (s, 9H), 0.18 (s, 6H). The spectroscopic data match those in the literature.^49^

#### General Arene Reduction Method A

A single-neck, 50 mL round-bottom flask was equipped with a magnetic stir bar and 2-way adapter with a glass stopcock connected to an argon, nitrogen, or oxygen balloon and subsequently purged. Under ambient atmosphere, no balloon was used, and the flask was lightly capped with a plastic stopper. A solution of an arene and 1-methoxyadamantane (internal standard, ca. 0.1–0.3 equiv) in dry THF (8.5 mL, 0.3 M) were added to the flask. The resulting mixture was cooled to 0 °C, then ethylenediamine (1.0 mL, 15 mmol, 6.0 equiv) and *t*-butanol (0.6 mL, 6.25 mmol, 2.5 equiv) were added using syringes. Lithium metal (52.1 mg, 7.5 mmol, 3.0 equiv) was cut into 2 mm pieces and added to the solution, and the reaction mixture was stirred at 0 °C (external temperature) for 1 h. An aliquot (ca. 2 mL) was taken and quenched with sat. NH_4_Cl (2 mL; CAUTION: Evolution of hydrogen gas), and the remaining reaction mixture was quenched with cold water (10 mL). The aliquot mixture was extracted with Et_2_O (2 mL × 3), and a sample (1 mL) was collected for GC-MS or concentrated *in vacuo* for ^1^H NMR analysis.

#### General Arene Reduction Method B

A single-neck, 50 mL round-bottom flask was equipped with a magnetic stir bar and 2-way adapter with a glass stopcock connected to an argon, nitrogen, or oxygen balloon and subsequently purged. Under ambient atmosphere, no balloon was used, and the flask was lightly capped with a plastic stopper. An arene substrate (2.5 mmol), 1-methoxyadamantane (internal standard, ca. 0.1–0.3 equiv), and dry THF (8.5 mL, 0.3 M) were added to the flask. The resulting mixture was cooled to 0 °C, then ethylenediamine (1.0 mL, 15 mmol, 6.0 equiv) and *t*-butanol (0.6 mL, 6.25 mmol, 2.5 equiv) were added using syringes. Lithium metal (52.1 mg, 7.5 mmol, 3.0 equiv) was cut into 2 mm pieces and added to the solution, and the reaction mixture was stirred at 0 °C (external temperature) for 1 h. An aliquot (ca. 2 mL) was taken and quenched with sat. NH_4_Cl (2 mL; CAUTION: Evolution of hydrogen gas), and the remaining reaction mixture was quenched with cold water (10 mL). The aliquot mixture was extracted with Et_2_O (2 mL × 3), and a sample (1 mL) was collected for GC-MS or concentrated *in vacuo* for ^1^H NMR analysis.

#### General Arene Reduction Method C

Unless stated otherwise, a single-neck, 50 mL round-bottom flask was equipped with a magnetic stir bar and 2-way adapter with a glass stopcock connected to an argon, nitrogen, or oxygen balloon and subsequently purged. Under ambient atmosphere, no balloon was used, and the flask was lightly capped with a plastic stopper. An arene substrate (2.0 mmol) and dry THF (6.7 mL, 0.3 M) were added to the flask. The resulting mixture was cooled to 0 °C, and then ethylenediamine (see specific procedure) and *t*-butanol (see specific procedure) were added using syringes. Lithium metal was cut into 2 mm pieces and added to the solution, and the reaction mixture was stirred at 0 °C (external temperature) for 1 h. The reaction mixture was quenched with sat. NH_4_Cl (8 mL; CAUTION: Evolution of hydrogen gas) then extracted with Et_2_O (20 mL × 2). The combined organic layers were washed with water, brine, dried with Na_2_SO_4_, then filtered through cotton. 1-Methoxyadamantane or 1,3,5-trimethoxybenzene (internal standard, 0.1–0.3 equiv) was added to the reaction crude mixture and an aliquot (ca. 2 mL) was collected for GC-MS or concentrated *in vacuo* for ^1^H NMR analysis.

#### General Arene Reduction Method D

Unless stated otherwise, a single-neck, 50 mL round-bottom flask was equipped with a magnetic stir bar and 2-way adapter with a glass stopcock connected to an argon, nitrogen, or oxygen balloon and subsequently purged. Under ambient atmosphere, no balloon was used, and the flask was lightly capped with a plastic stopper. An arene substrate (2.0 mmol) and dry THF (6.7 mL, 0.3 M) were added to the flask. The resulting mixture was cooled to 0 °C, then ethylenediamine (see specific procedure) and *t*-butanol (see specific procedure) were added using syringes. Lithium metal was cut into 2 mm pieces and added to the solution, and the reaction mixture was stirred at 0 °C (external temperature) for 1 h. The reaction mixture was quenched with sat. NH_4_Cl (8 mL, CAUTION: Evolution of hydrogen gas). The reaction mixture is acidified to pH 2 with concentrated HCl before extracting with Et_2_O (20 mL × 2). The combined organic layers were washed with water, brine, dried with Na_2_SO_4_, then filtered through cotton. 1-Methoxyadamantane or 1,3,5-trimethoxybenzene (internal standard, 0.1–0.3 equiv) was added to the reaction crude mixture and an aliquot (ca. 2 mL) was concentrated *in vacuo* for ^1^H NMR analysis.

#### Kinetic Study Monitoring the Reduction of Naphthalene (**8**)

A three-neck, 50 mL round-bottom flask was equipped with a magnetic stir bar. A 2-way adapter with a glass stopcock connected to an argon or oxygen balloon was placed on the center neck and subsequently purged. The left and right arms of the flask were sealed with a rubber septum. Naphthalene (384.5 mg, 3.0 mmol), 1-methoxyadamantane (internal standard, 0.1–0.3 equiv), and dry THF (10 mL, 0.3 M) were added to the flask. The resulting mixture was cooled to 0 °C, then ethylenediamine (1.2 mL, 18 mmol, 6.0 equiv) and *t*-butanol (720 μL, 7.5 mmol, 2.5 equiv) were added using syringes. Lithium metal (62.5 mg, 9.0 mmol, 3.0 equiv) was cut into 2 mm pieces and added to the solution, and the reaction mixture was stirred at 0 °C (external temperature) for 1 h. An aliquot (0.1 mL) of the reaction mixture was taken via syringe and quenched with sat. NH_4_Cl (0.1 mL) at 0, 5, 10, 15, 30, 45, and 60 min. The aliquots are extracted with Et_2_O (1 mL × 2) and concentrated *in vacuo* until most of the solvent was removed. The crude mixture is then used directly for ^1^H NMR analysis.

#### Reduction of Naphthalene (**8**) in the Presence of KO_2_

A single-neck, 50 mL round-bottom flask was equipped with a magnetic stir bar and 2-way adapter with a glass stopcock connected to an argon balloon and subsequently purged. Naphthalene (256.3 mg, 2.0 mmol) and dry THF (6.7 mL, 0.3 M) were added to the flask. The resulting mixture was cooled to 0 °C, then ethylenediamine (0.8 mL, 12 mmol, 6.0 equiv) and *t*-butanol (480 μL, 5.0 mmol, 2.5 equiv) were added using syringes. Lithium metal (41.6 mg, 6.0 mmol, 3.0 equiv) was cut into 2 mm pieces and placed in a separate vial purged with argon. KO_2_ was measured (CAUTION: KO_2_ is very moisture sensitive and reacts violently with water). Lithium then KO_2_ were added to the solution, and the reaction mixture was stirred at 0 °C (external temperature) for 1 h. The reaction mixture was quenched with sat. NH_4_Cl (CAUTION: Evolution of hydrogen gas, see previous warning) slowly then extracted with Et_2_O (20 mL × 2). The combined organic layers were washed with water, brine, dried with Na_2_SO_4_, then filtered through cotton. 1-Methoxyadamantane (internal standard, 0.1–0.3 equiv) was added to the reaction crude mixture, and an aliquot (ca. 2 mL) was collected and concentrated *in vacuo* for ^1^H NMR analysis.

#### Reduction of Toluene (**1**)

##### under Ar Following General Arene Reduction Method A

A stock solution of toluene (552.8 mg, 6.0 mmol) and 1-methoxyadamantane (133.0 mg, 0.80 mmol) in THF (20 mL) was made for the reaction (8.5 mL). The conversion of toluene in the reaction mixture was determined to be >99% based on the internal standard compared to the remaining starting material as shown in Chromatogram S1.

##### Under N_2_ Following General Arene Reduction Method A

A stock solution of toluene (552.8 mg, 6.0 mmol) and 1-methoxyadamantane (133.0 mg, 0.80 mmol) in THF (20 mL) was made for the reaction (8.5 mL). The conversion of toluene in the reaction mixture was determined to be >99% based on the internal standard compared to the remaining starting material as shown in Chromatogram S2.

##### under Air Following General Arene Reduction Method B

Toluene (230.4 mg, 2.5 mmol) and 1-methoxyadamantane (51.5 mg, 0.31 mmol) were measured for the reaction. The conversion of toluene in the reaction mixture was determined to be 99% based on the internal standard compared to the remaining starting material as shown in Chromatogram S3.

##### Under O_2_ Following General Arene Reduction Method B

Toluene (230.4 mg, 2.5 mmol) and 1-methoxyadamantane (73.1 mg, 0.44 mmol) were measured for the reaction. The conversion of toluene in the reaction mixture was determined to be 94% based on the internal standard compared to the remaining starting material as shown in Chromatogram S4.

#### Reduction of *o*-Xylene (**2**)

##### Under Ar Following General Arene Reduction Method A

A stock solution of *o*-xylene (637.0 mg, 6.0 mmol) and 1-methoxyadamantane (148.5 mg, 0.89 mmol) in THF (20 mL) was made for the reaction (8.5 mL). The conversion of *o*-xylene in the reaction mixture was determined to be >99% based on the internal standard compared to the remaining starting material as shown in Chromatogram S5.

##### Under N_2_ Following General Arene Reduction Method A

A stock solution of *o*-xylene (637.0 mg, 6.0 mmol) and 1-methoxyadamantane (148.5 mg, 0.89 mmol) in THF (20 mL) was made for the reaction (8.5 mL). The conversion of *o*-xylene in the reaction mixture was determined to be >99% based on the internal standard compared to the remaining starting material as shown in Chromatogram S6.

##### Under Air Following General Arene Reduction Method B

*o*-Xylene (265.4 mg, 2.5 mmol) and 1-methoxyadamantane (50.6 mg, 0.30 mmol) were measured for the reaction. The conversion of *o*-xylene in the reaction mixture was determined to be 96% based on the internal standard compared to the remaining starting material as shown in Chromatogram S7.

##### Under O_2_ Following General Arene Reduction Method B

*o*-Xylene (265.4 mg, 2.5 mmol) and 1-methoxyadamantane (54.1 mg, 0.33 mmol) were measured for the reaction. The conversion of *o*-xylene in the reaction mixture was determined to be 74% based on the internal standard compared to the remaining starting material as shown in Chromatogram S8.

#### Reduction of *p*-Pymene (**3**)

##### Under Ar Following General Arene Reduction Method A

A stock solution of *p*-cymene (805.3 mg, 6.0 mmol) and 1-methoxyadamantane (124.2 mg, 0.75 mmol) in THF (20 mL) was made for the reaction (8.5 mL). The conversion of *p*-cymene in the reaction mixture was determined to be >99% based on the internal standard compared to the remaining starting material as shown in Chromatogram S9.

##### Under N_2_ Following General Arene Reduction Method A

A stock solution of *p*-cymene (805.3 mg, 6.0 mmol) and 1-methoxyadamantane (124.2 mg, 0.75 mmol) in THF (20 mL) was made for the reaction (8.5 mL). The conversion of *p*-cymene in the reaction mixture was determined to be >99% based on the internal standard compared to the remaining starting material as shown in Chromatogram S10.

##### Under Air Following General Arene Reduction Method B

*p*-Cymene (335.5 mg, 2.5 mmol) and 1-methoxyadamantane (63.5 mg, 0.38 mmol) were measured for the reaction. The conversion of *p*-cymene in the reaction mixture was determined to be 93% based on the internal standard compared to the remaining starting material as shown in Chromatogram S11.

##### Under O_2_ Following General Arene Reduction Method B

*p*-Cymene (335.5 mg, 2.5 mmol) and 1-methoxyadamantane (53.2 mg, 0.32 mmol) were measured for the reaction. The conversion of *p*-cymene in the reaction mixture was determined to be 75% based on the internal standard compared to the remaining starting material as shown in Chromatogram S12.

#### Reduction of Anisole (**4**)

##### Under Ar Following General Arene Reduction Method A

A stock solution of anisole (648.8 mg, 6.0 mmol) and 1-methoxyadamantane (117.2 mg, 0.70 mmol) in THF (20 mL) was made for the reaction (8.5 mL). The conversion of anisole in the reaction mixture was determined to be >99% based on the internal standard compared to the remaining starting material, as shown in Chromatogram S13.

##### Under N_2_ Following General Arene Reduction Method A

A stock solution of anisole (648.8 mg, 6.0 mmol) and 1-methoxyadamantane (117.2 mg, 0.70 mmol) in THF (20 mL) was made for the reaction (8.5 mL). The conversion of anisole in the reaction mixture was determined to be >99% based on the internal standard compared to the remaining starting material as shown in Chromatogram S14.

##### Under Air Following General Arene Reduction Method B

Anisole (270.4 mg, 2.5 mmol) and 1-methoxyadamantane (44.6 mg, 0.27 mmol) were measured for the reaction. The conversion of anisole in the reaction mixture was determined to be 96% based on the internal standard compared to the remaining starting material as shown in Chromatogram S15.

##### Under O_2_ Following General Arene Reduction Method B

Anisole (270.4 mg, 2.5 mmol) and 1-methoxyadamantane (43.5 mg, 0.26 mmol) were measured for the reaction. The conversion of anisole in the reaction mixture was determined to be 92% based on the internal standard compared to the remaining starting material as shown in Chromatogram S16.

#### Reduction of *n*-Butoxybenzene (**5**)

##### Under Ar Following General Arene Reduction Method A

A stock solution of *n*-butoxybenzene (901.3 mg, 6.0 mmol) and 1-methoxyadamantane (127.0 mg, 0.76 mmol) in THF (20 mL) was made for the reaction (8.5 mL). The conversion of *n*-butoxybenzene in the reaction mixture was determined to be >99% based on the internal standard compared to the remaining starting material as shown in Chromatogram S17.

##### Under N_2_ Following General Arene Reduction Method A

A stock solution of *n*-butoxybenzene (901.3 mg, 6.0 mmol) and 1-methoxyadamantane (127.0 mg, 0.76 mmol) in THF (20 mL) was made for the reaction (8.5 mL). The conversion of *n*-butoxybenzene in the reaction mixture was determined to be 96% based on the internal standard compared to the remaining starting material as shown in Chromatogram S18.

##### Under Air Following General Arene Reduction Method B

*n*-Butoxybenzene (375.5 mg, 2.5 mmol) and 1-methoxyadamantane (68.5 mg, 0.41 mmol) were measured for the reaction. The conversion of *n*-butoxybenzene in the reaction mixture was determined to be 95% based on the internal standard compared to the remaining starting material as shown in Chromatogram S19.

##### Under O_2_ Following General Arene Reduction Method B

*n*-Butoxybenzene (375.5 mg, 2.5 mmol) and 1-methoxyadamantane (50.3 mg, 0.30 mmol) were measured for the reaction. The conversion of *n*-butoxybenzene in the reaction mixture was determined to be 87% based on the internal standard compared to the remaining starting material as shown in Chromatogram S20.

#### Reduction of *t*-Butyldimethyl(*p*-tolyloxy)silane (**6**)

##### Under Ar Following General Arene Reduction Method A

A stock solution of *t*-butyldimethyl(*p*-tolyloxy)silane (1,334.4 mg, 6.0 mmol) and 1-methoxyadamantane (106.2 mg, 0.64 mmol) in THF (20 mL) was made for the reaction (8.5 mL). The conversion of *t*-butyldimethyl(*p*-tolyloxy)silane in the reaction mixture was determined to be >99% based on the internal standard compared to the remaining starting material as shown in Chromatogram S21.

##### Under N_2_ Following General Arene Reduction Method A

A stock solution of *t*-butyldimethyl(*p*-tolyloxy)silane (1,334.4 mg, 6.0 mmol) and 1-methoxyadamantane (106.2 mg, 0.64 mmol) in THF (20 mL) was made for the reaction (8.5 mL). The conversion of *t*-butyldimethyl(*p*-tolyloxy)silane in the reaction mixture was determined to be >99%% based on the internal standard compared to the remaining starting material as shown in Chromatogram S22.

##### Under Air Following General Arene Reduction Method B

*t*-Butyldimethyl(*p*-tolyloxy)silane (556.0 mg, 2.5 mmol) and 1-methoxyadamantane (63.2 mg, 0.38 mmol) were measured for the reaction. The conversion of *t*-butyldimethyl(*p*-tolyloxy)silane in the reaction mixture was determined to be 99% based on the internal standard compared to the remaining starting material as shown in Chromatogram S23.

##### Under O_2_ Following General Arene Reduction Method B

*t*-Butyldimethyl(*p*-tolyloxy)silane (556.0 mg, 2.5 mmol) and 1-methoxyadamantane (44.9 mg, 0.27 mmol) were measured for the reaction. The conversion of *t*-butyldimethyl(*p*-tolyloxy)silane in the reaction mixture was determined to be 85% based on the internal standard compared to the remaining starting material as shown in Chromatogram S24.

#### Reduction of 3-Methylanisole (**7**)

##### Under Ar Following General Arene Reduction Method A

A stock solution of 3-methyl anisole (733.0 mg, 6.0 mmol) and 1-methoxy adamantane (105.8 mg, 0.64 mmol) in THF (20 mL) was made for the reaction (8.5 mL). The conversion of 3-methyl anisole in the reaction mixture was determined to be >99% based on the internal standard compared to the remaining starting material, as shown in Chromatogram S25.

##### Under N_2_ Following General Arene Reduction Method A

A stock solution of 3-methylanisole (733.0 mg, 6.0 mmol) and 1-methoxyadamantane (105.8 mg, 0.64 mmol) in THF (20 mL) was made for the reaction (8.5 mL). The conversion of 3-methylanisole in the reaction mixture was determined to be 94%% based on the internal standard compared to the remaining starting material as shown in Chromatogram S26.

##### Under Air Following General Arene Reduction Method B

3-Methylanisole (305.4 mg, 2.5 mmol) and 1-methoxyadamantane (59.4 mg, 0.36 mmol) were measured for the reaction. The conversion of 3-methylanisole in the reaction mixture was determined to be 96% based on the internal standard compared to the remaining starting material as shown in Chromatogram S27.

##### Under O_2_ Following General Arene Reduction Method B

3-Methylanisole (305.4 mg, 2.5 mmol) and 1-methoxyadamantane (56.2 mg, 0.34 mmol) were measured for the reaction. The conversion of 3-methylanisole in the reaction mixture was determined to be 90% based on the internal standard compared to the remaining starting material as shown in Chromatogram S28.

#### Reduction of Naphthalene (**8**)

##### Under Ar Following General Arene Reduction Method A

A stock solution of naphthalene (769.0 mg, 6.0 mmol) and 1-methoxyadamantane (116.5 mg, 0.70 mmol) in THF (20 mL) was made for the reaction (8.5 mL). The conversion of naphthalene in the reaction mixture was determined to be 86% based on the internal standard compared to the remaining starting material as shown in Chromatogram S29.

##### Under N_2_ Following General Arene Reduction Method A

A stock solution of naphthalene (769.0 mg, 6.0 mmol) and 1-methoxyadamantane (116.5 mg, 0.70 mmol) in THF (20 mL) was made for the reaction (8.5 mL). The conversion of naphthalene in the reaction mixture was determined to be 86% based on the internal standard compared to the remaining starting material as shown in Chromatogram S30.

##### Under Air Following General Arene Reduction Method B

Naphthalene (320.4 mg, 2.5 mmol) and 1-methoxyadamantane (60.0 mg, 0.36 mmol) were measured for the reaction. The conversion of naphthalene in the reaction mixture was unable to be determined due to coelution of an additional reduction byproduct as shown in Chromatogram S31.

##### Under O_2_ Following General Arene Reduction Method B

Naphthalene (320.4 mg, 2.5 mmol) and 1-methoxyadamantane (51.4 mg, 0.31 mmol) were measured for the reaction. The conversion of naphthalene in the reaction mixture was unable to be determined due to coelution of an additional reduction byproduct as shown in Chromatogram S32.

#### Reduction of Naphthalene (**8**) and Analysis of Product Distribution

##### Under Ar Following General Arene Reduction Method A

A stock solution of naphthalene (769.0 mg, 6.0 mmol) and 1-methoxyadamantane (103.4 mg, 0.62 mmol) in THF (20 mL) was made for the reaction (8.5 mL). The yield of the reaction was determined to be 34% 1,2-dihydronaphthalene, 38% 1,4,5,8-tetrahydronaphthalene, ∼ 15% 1,2,3,4-tetrahydronaphthalene, and 14% remaining naphthalene based on the internal standard as shown in Spectrum S28.

##### Under Air Following General Arene Reduction Method A

A stock solution of naphthalene (769.0 mg, 6.0 mmol) and 1-methoxyadamantane (103.4 mg, 0.62 mmol) in THF (20 mL) was made for the reaction (8.5 mL). The yield of the reaction was determined to be 35% 1,4-dihydronaphthalene, < 2% 1,2-dihydronaphthalene, 33% 1,4,5,8-tetrahydronaphthalene, ∼ 11% 1,2,3,4-tetrahydronaphthalene, and 20% remaining naphthalene based on the internal standard as shown in Spectrum S29.

##### Under O_2_ Following General Arene Reduction Method B

Naphthalene (320.4 mg, 2.5 mmol) and 1-methoxyadamantane (51.4 mg, 0.31 mmol) were measured for the reaction. The yield of the reaction was determined to be 32% 1,4-dihydronaphthalene, < 2% 1,2-dihydronaphthalene, 38% 1,4,5,8-tetrahydronaphthalene, 10% 1,2,3,4-tetrahydronaphthalene, and 15% remaining naphthalene based on the internal standard as shown in Spectrum S30.

Naphthalene: ^1^H NMR (400 MHz, CDCl_3_): δ 7.85 (dd, 4H, *J* = 6.2, 3.3 Hz), 7.48 (dd, 4H, J = 6.3, 3.3 Hz). The spectral data match those previously reported.^50^

1,4-Dihydronaphthalene: ^1^H NMR (400 MHz, CDCl_3_): δ 7.19–7.10 (m, 4H), 5.94–5.91 (m, 2H), 3.42–3.37 (m, 4H). The spectral data match those previously reported.^51^

1,2-Dihydronaphthalene: ^1^H NMR (400 MHz, CDCl_3_): δ 7.18–7.09 (m, 3H), 7.04–6.99 (m, 1H), 6.46 (dt, 1H, *J* = 9.6, 1.6 Hz), 6.02 (dt, 1H, *J* = 9.6, 4.4 Hz), 2.80 (t, 2H, *J* = 8.2 Hz), 2.32 (tdd, 2H, *J* = 8.5, 4.4, 1.8 Hz). The spectral data match those previously reported.^52^

1,4,5,8-Tetrahydronaphthalene: ^1^H NMR (400 MHz, CDCl_3_): δ 5.73 (s, 4H), 2.54 (s, 8H). The spectral data match those previously reported.^53^

1,2,3,4-Tetrahydronaphthalene: ^1^H NMR (400 MHz, CDCl_3_): δ 7.09–7.04 (m, 4H), 2.80–2.74 (m, 4H), 1.83–1.77 (m, 4H). The spectral data match those previously reported.^54^

#### Kinetic Study Monitoring the Reduction of Naphthalene (**8**)

##### Under Ar Following the General Kinetic Method

Naphthalene (384.5 mg, 3.0 mmol) and 1-methoxyadamantane (53.9 mg, 0.32 mmol) were measured for the reaction. Yields over time based on the internal standard are shown in Table S1 and Spectrum S31.

##### Under O_2_ Following the General Kinetic Method

Naphthalene (384.5 mg, 3.0 mmol) and 1-methoxyadamantane (60.6 mg, 0.36 mmol) were measured for the reaction. Yields over time based on the internal standard are shown in Table S2 and Spectrum S32.

#### Reduction of Naphthalene (**8**) Using Sub- and Superstoichiometric Amounts of Reductant

Using general arene reduction method C with naphthalene (256.3 mg, 2.0 mmol) and *t*-butanol (480 μL, 5.0 mmol, 2.5 equiv); reagent amounts, atmosphere, and results are shown in Table S3 and Spectra S33–S36.

#### Reduction of Napththalene (**8**) in the Presence of KO_2_

See general KO_2_ procedure; KO_2_ amounts and results are shown in Table S4 and Spectra S37–S43. Entry 7 of Table S3 was carried out using the general KO_2_ procedure but instead of KO_2_ as the oxygen species, molecular oxygen was bubbled into THF for 15 min prior to use in the reaction.

#### Reaction of 1,4-Dihydronaphthalene (**9**) with KO_2_

A single-neck, 50 mL round-bottom flask was equipped with a magnetic stir bar and 2-way adapter with a glass stopcock connected to an argon balloon and subsequently purged. 1,4-Dihydronaphthalene (130.2 mg, 1.0 mmol) and dry THF (3.4 mL, 0.3 M) were added to the flask. The resulting mixture was cooled to 0 °C, then ethylenediamine (0.4 mL, 6.0 mmol, 6.0 equiv) and *t*-butanol (240 μL, 2.5 mmol, 2.5 equiv) were added using syringes. Quickly, KO_2_ (356 mg, 5.0 mmol, 5.0 equiv) was measured (CAUTION: KO_2_ is very moisture sensitive and reacts violently with water). KO_2_ was added to the solution, and the reaction mixture was stirred at 0 °C (external temperature) for 1 h. The reaction mixture was quenched with sat. NH_4_Cl (CAUTION: Evolution of hydrogen gas, see previous warning) slowly then extracted with Et_2_O (20 mL × 2). The collected organic layer was washed with water, brine, dried with Na_2_SO_4_, then filtered through cotton. 1-methoxyadamantane (internal standard, 36.2 mg, 0.22 mmol) was added to the reaction crude mixture and an aliquot (ca. 2 mL) was collected and concentrated *in vacuo* for ^1^H NMR analysis. From the reaction crude mixture, it was determined that there was no reactivity when compared to a ^1^H NMR spectrum of the starting material as shown in Spectrum S44.

#### Reaction of 1,2-Dihydronaphthalene (**10**) with KO_2_

A single-neck, 50 mL round-bottom flask was equipped with a magnetic stir bar and 2-way adapter with a glass stopcock connected to an argon balloon and subsequently purged. 1,2-Dihydronaphthalene (130.2 mg, 1.0 mmol) and dry THF (3.4 mL, 0.3 M) were added to the flask. The resulting mixture was cooled to 0 °C, then ethylenediamine (0.4 mL, 6.0 mmol, 6.0 equiv) and *t*-butanol (240 μL, 2.5 mmol, 2.5 equiv) were added using syringes. Quickly, KO_2_ (356 mg, 5.0 mmol, 5.0 equiv) was measured (CAUTION: KO_2_ is very moisture sensitive and reacts violently with water). KO_2_ was added to the solution, and the reaction mixture was stirred at 0 °C (external temperature) for 1 h. The reaction mixture was quenched with sat. NH_4_Cl (CAUTION: Evolution of hydrogen gas, see previous warning) slowly then extracted with Et_2_O (20 mL × 2). The collected organic layer was washed with water, brine, dried with Na_2_SO_4_, then filtered through cotton. 1-methoxyadamantane (internal standard, 38.7 mg, 0.23 mmol) was added to the reaction crude mixture and an aliquot (ca. 2 mL) was collected and concentrated *in vacuo* for ^1^H NMR analysis. From the reaction crude mixture, it was determined that there was no reactivity when compared to a ^1^H NMR spectrum of the starting material as shown in Spectrum S45.

#### Reduction of 2-Methylnaphthalene to 2-Methyl-1,4,5,8**-**tetrahydronaphthalene (**14**)

Using general arene reduction method C with 2-methylnaphthalene (284.4 mg, 2.0 mmol), lithium metal (69.4 mg, 10 mmol, 5.0 equiv), ethylenediamine (1.34 mL, 20 mmol, 10 equiv), and THF (6.7 mL); reagent amounts, atmosphere, and results are shown in Table S5, Spectra S46–S51, and Chromatograms S35–S40.

2-Methyl-1,4,5,8-tetrahydronaphthalene: ^1^H NMR (300 MHz, CDCl_3_): δ 5.76–5.70 (m, 2H), 5.42 (tq, 1H, 3.0, 1.5 Hz), 2.59–2.49 (m, 6H), 2.48–2.39 (m, 2H), 1.69 (s, 3H). The spectral data match those previously reported.^14^

#### Reduction of 2-Methoxynaphthalene to 2-Methoxy-1,4,5,8**-**tetrahydronaphthalene (**15**)

Using general arene reduction method C with 2-methoxynaphthalene (316.4 mg, 2.0 mmol), lithium metal (69.4 mg, 10 mmol, 5.0 equiv), ethylenediamine (1.34 mL, 20 mmol, 10 equiv), and THF (6.7 mL); reagent amounts, atmosphere, and results are shown in Table S6, Spectra S52–S57, and Chromatograms S41–S46.

2-Methoxy-1,4,5,8-tetrahydronaphthalene: ^1^H NMR (300 MHz, CDCl_3_): δ 5.69 (s, 2H), 4.60 (t, 1H, *J* = 3.3 Hz), 3.52 (s, 3H), 2.68–2.59 (m, 2H), 2.59–2.46 (m, 6H). The spectral data match those previously reported.^55^

#### Reduction of Naphthalene (**8**) to 1,4,5,8-Tetrahydronaphthalene (**11**)

Using general arene reduction method C with naphthalene (256.3 mg, 2.0 mmol), lithium metal (69.4 mg, 10 mmol, 5.0 equiv), ethylenediamine (1.34 mL, 20 mmol, 10 equiv), *t*-butanol (960 μL, 10 mmol, 5.0 equiv), and THF (6.7 mL); atmosphere and results are shown in Table S7, Spectra S58–S60, and Chromatograms S47–S49.

1,4,5,8-Tetrahydronaphthalene: ^1^H NMR (400 MHz, CDCl_3_): δ 5.73 (s, 4H), 2.54 (s, 8H). The spectral data match those previously reported.^53^

#### Reduction of 2-(Naphthalen-2-yl)acetic acid to 2-(1,4,5,8-Tetrahydronaphthalen-2-yl)acetic acid (**16**)

Using general arene reduction method D with 2-(naphthalene-2-yl)acetic acid (372.4 mg, 2.0 mmol), lithium metal (69.4 mg, 10 mmol, 5.0 equiv), ethylenediamine (1.34 mL, 20 mmol, 10 equiv), *t*-butanol (960 μL, 10 mmol, 5.0 equiv), and THF (6.7 mL); atmosphere and results are shown in Table S8 and Spectra S63–S65. A portion of the reaction crude mixture was used to recrystallize from MeOH, following a previously reported procedure,^56^ to yield 2-(1,4,5,8-tetrahydronaphthalen-2-yl)acetic acid as white needle-like crystals.

2-(1,4,5,8-Tetrahydronaphthalen-2-yl)acetic acid: mp = 171–173 °C; *R*_*f*_ = 0.29 (25% EtOAc in hexanes with 1% AcOH); IR (neat): ν_max_ = 2952 (broad, O–H), 2886, 2855, 2822, 1691, 1405, 1345, 1248 cm^–1^; ^1^H NMR (300 MHz, CDCl_3_): δ 5.72 (s, 2H), 5.66 (s, 1H), 3.05 (s, 2H), 2.64–2.57 (m, 4H), 2.54 (s, 4H). The spectral data match those previously reported.^56^^13^C NMR (75 MHz, DMSO-*d*_6_): δ 172.5, 128.9, 124.2, 124.1, 122.6, 122.3, 121.9, 42.2, 33.5, 31.2, 30.1, 29.9; HRMS (ESI-TOF+) *m*/*z* for [M + H]^+^ C_12_H_15_O_2_, calcd 191.1066, found 191.1063.

#### Reduction of 1-Methoxynaphthalene to 5-Methoxy-1,4-dihydronaphthalene (**17**)

Using general arene reduction method C with 1-methoxynaphthalene (316.4 mg, 2.0 mmol), lithium metal (41.6 mg, 6.0 mmol, 3.0 equiv), ethylenediamine (0.8 mL, 12 mmol, 6.0 equiv), *t*-butanol (574 μL, 6.0 mmol, 3.0 equiv), and THF (6.7 mL); atmosphere and results are shown in Table S9, Spectra S66–S68, and Chromatograms S50–S52.

5-Methoxy-1,4-dihydronaphthalene: ^1^H NMR (300 MHz, CDCl_3_): δ 7.12–7.04 (m, 1H), 6.69 (d, 1H, *J* = 7.7 Hz), 6.64 (d, 1H *J* = 8.1 Hz), 5.92–5.81 (m, 2H), 3.78 (s, 3H), 3.38–3.31 (m, 2H), 3.27–3.18 (m, 2H). The spectral data match those previously reported.^57^

#### Reduction of 1-Methylnaphthalene to 5-Methyl-1,4-dihydronaphthalene (**18**)

Using general arene reduction method C with 1-methylnaphthalene (284.4 mg, 2.0 mmol), lithium metal (41.6 mg, 6.0 mmol, 3.0 equiv), ethylenediamine (0.8 mL, 12 mmol, 6.0 equiv), *t*-butanol (574 μL, 6.0 mmol, 3.0 equiv), and THF (6.7 mL); atmosphere and results are shown in Table S10, Spectra S69–S71, and Chromatograms S53–S55.

5-Methyl-1,4-dihydronaphthalene: ^1^H NMR (300 MHz, CDCl_3_): δ 7.07–6.86 (m, 3H), 5.87 (bs, 2H), 3.42–3.35 (m, 2H), 3.25–3.17 (m, 2H), 2.21 (s, 3H). The spectral data match those previously reported.^57^

#### Reduction of 1-Naphthoic acid to 1,4-Dihydronaphthalene-1-carboxylic acid (**19**)

Using general arene reduction method D with 1-naphthoic acid (344.4 mg, 2.0 mmol), lithium metal (41.6 mg, 6.0 mmol, 3.0 equiv), ethylenediamine (0.8 mL, 12 mmol, 6.0 equiv), and THF (6.7 mL); no proton donor was used; atmosphere and results are shown in Table S11 and Spectra S72–S74.

1,4-Dihydronaphthalene-1-carboxylic acid: ^1^H NMR (300 MHz, CDCl_3_): δ 7.32–7.10 (m, 4H), 6.19 (dddd, 1H, *J* = 9.7, 4.6, 2.7, 1.3 Hz), 5.99 (dddd, 1H, *J* = 9.8, 4.7, 2.8, 1.3 Hz), 4.44 (q, 1H, *J* = 4.1 Hz), 3.62–3.30 (m, 2H). The spectral data match those previously reported.^32^

#### Reduction of 2-(Naphthalen-1-yl)ethan-1-ol to 2-(5,8-Dihydronaphthalen-1-yl)ethan-1-ol (**20**)

Using general arene reduction method C with 2-(naphthalen-1-yl)ethan-1-ol (344.4 mg, 2.0 mmol), lithium metal (41.6 mg, 6.0 mmol, 3.0 equiv), ethylenediamine (0.8 mL, 12 mmol, 6.0 equiv), *t*-butanol (574 μL, 6.0 mmol, 3.0 equiv), and THF (6.7 mL); atmosphere and results are shown in Table S12 and Spectra S75–S77.

2-(Dihydronaphthalen-1-yl)ethan-1-ol: ^1^H NMR (MHz, CDCl_3_): δ 7.18–6.94 (m, 3H), 5.91 (t, 2H, *J* = 1.5 Hz), 3.84 (t, 2H, *J* = 6.7 Hz), 3.46–3.32 (m, 4H), 2.88 (t, 2H, *J* = 6.8 Hz). The spectral data match those previously reported.^57^

#### Reduction of 1-Naphthol to 5,8-Dihydronaphthalen-1-ol (**21**)

Using general arene reduction method C with 1-naphthol (288.3 mg, 2.0 mmol), lithium metal (41.6 mg, 6.0 mmol, 3.0 equiv), ethylenediamine (0.8 mL, 12 mmol, 6.0 equiv), *t*-butanol (574 μL, 6.0 mmol, 3.0 equiv), and THF (6.7 mL); atmosphere and results are shown in Table S13, Spectra S78–S80, and Chromatograms S56–S58.

5,8-Dihydronaphthalen-1-ol: ^1^H NMR (300 MHz, CDCl_3_): δ 7.07–6.99 (m, 1H), 6.74–6.67 (m, 1H), 6.64–6.59 (m, 1H), 5.97–5.84 (m, 2H), 3.44–3.36 (m, 2H), 3.32–3.26 (m, 2H). The spectral data match those previously reported.^58^

#### Reduction of 2-Naphthol to 5,8-Dihydronaphthalen-2-ol (**22**)

Using general arene reduction method C with 2-naphthol (288.3 mg, 2.0 mmol), lithium metal (41.6 mg, 6.0 mmol, 3.0 equiv), ethylenediamine (0.8 mL, 12 mmol, 6.0 equiv), *t*-butanol (574 μL, 6.0 mmol, 3.0 equiv), and THF (6.7 mL); atmosphere and results are shown in Table S14, Spectra S81–S83, and Chromatograms S59–S61.

5,8-Dihydronaphthalen-2-ol: ^1^H NMR (300 MHz, CDCl_3_): δ 6.92 (d, 1H, *J* = 8.2 Hz), 6.61 (dd, 1H, *J* = 8.2, 2.7 Hz), 6.56–6.54 (m, 1H), 5.91–5.78 (m, 2H), 3.30–3.25 (m, 4H). The spectral data match those previously reported.^59^

#### Reduction of 1-Aminonaphthalene to 5,8-Dihydronaphthalene-1-amine (**23**)

Using general arene reduction method C with 1-aminonaphthalene (286.4 mg, 2.0 mmol), lithium metal (41.6 mg, 6.0 mmol, 3.0 equiv), ethylenediamine (0.8 mL, 12 mmol, 6.0 equiv), *t*-butanol (574 μL, 6.0 mmol, 3.0 equiv), and THF (6.7 mL); atmosphere and results are shown in Table S15, Spectra S84–S86, and Chromatograms S62–S64.

5,8-Dihydronaphthalen-1-amine: ^1^H NMR (300 MHz, CDCl_3_): δ 6.96 (t, 1H, *J* = 7.7 Hz), 6.58–6.49 (m, 2H), 5.92–5.81 (m, 2H), 3.40–3.33 (m, 2H), 3.11–3.03 (m, 2H). The spectral data match those previously reported.^53^

#### Reduction of 1-Aminonapthtalene to 5,8-Dihydronaphthalene-amine (**23**) on a 20 mmol Scale

A three-neck, 250 mL round-bottom flask was equipped with a magnetic stir bar, a PTFE thermometer holder on the left neck, and a rubber septum on the right neck while the middle neck was open to air. 1-Aminonaphthalene (2.864 g, 20 mmol) and dry THF (67 mL, 0.3 M) were added to the flask. The resulting mixture was cooled to 0 °C, and then ethylenediamine (8.02 mL, 120 mmol, 6.0 equiv) and *t*-butanol (5.74 mL, 60 mL, 3.0 equiv) were added using syringes. The external and internal temperatures were monitored to ensure the reaction mixture stayed below 5 °C. Lithium metal was cut into 2 mm pieces and added to the solution portion-wise over ca. 30 min (ca. 50–80 mg portions), and the reaction mixture was stirred at 0 °C (external temperature) for 1 h, monitoring the internal temperature did not spike above 5 °C. The reaction mixture was quenched with sat. NH_4_Cl slowly (30 mL; CAUTION: Evolution of hydrogen gas! An exotherm was observed and the internal temperature rose to 25 °C) The reaction mixture was allowed to stir until all the remaining lithium was destroyed and the internal temperature returned to below 5 °C. The THF was mostly removed in vacuo before extracting with Et_2_O (30 mL × 2). The combined organic layers were washed with water, brine, dried with Na_2_SO_4_, then filtered through cotton. 1-Methoxyadamantane (331.5 mg, 1.99 mmol) was added to the reaction crude mixture and an aliquot (ca. 2 mL) was collected and concentrated *in vacuo* for ^1^H NMR analysis showing a yield of 77%, see Spectrum S87.

#### Reduction of 2,3-Naphthalenediol to 5,8-Dihydronaphthalene-2,3-diol (**24**)

Using general arene reduction method C with 2,3-naphthalenediol (320.3 mg, 2.0 mmol), lithium metal (41.6 mg, 6.0 mmol, 3.0 equiv), ethylenediamine (0.8 mL, 12 mmol, 6.0 equiv), *t*-butanol (574 μL, 6.0 mmol, 3.0 equiv), and THF (6.7 mL); the reaction crude mixture was extracted with EtOAc instead of Et_2_O; atmosphere and results are shown in Table S16 and Spectra S90–S92. A portion of the reaction crude mixture was purified by flash column chromatography (SiO_2_, 30% EtOAc in hexanes) to yield 5,8-dihydronaphthalene-2,3-diol as a waxy white solid.

5,8-Dihydronaphthalene-2,3-diol: *R*_*f*_ = 0.29 (30% EtOAc in hexanes); IR (neat): ν_max_ = 3393, 3025, 2922, 2856, 2824, 1529, 1452, 1275, 1099 cm^–1^; ^1^H NMR (300 MHz, DMSO-*d*_6_): δ 8.60 (s, 2H), 6.45 (s, 2H), 5.83 (s, 2H), 3.14 (d, 4H, *J* = 0.9 Hz); ^13^C NMR (75 MHz, CDCl_3_): δ 143.6, 124.6, 123.7, 114.9, 28.5; HRMS (ESI-TOF+) *m*/*z* for [M - H]^−^ C_10_H_9_O_2_, calcd 161.0597, found 161.0602.

#### Reduction of Anthracene (**25**) to 1,4,5,8,9,10-Hexahydroanthracene (**26**)

Using general arene reduction method C with anthracene (356.4 mg, 2.0 mmol), lithium metal (97.2 mg, 14 mmol, 7.0 equiv), ethylenediamine (1.87 mL, 28 mmol, 14 equiv), *t*-butanol (1.34 mL, 14 mmol, 7.0 equiv), and THF (6.7 mL) performed at 23 °C; atmosphere and results are shown in Table S17, Spectra S95–S97, and Chromatograms S65–S67. A portion of the reaction crude mixture was used to recrystallize from CHCl_3_:C_6_H_6_, following a previously reported procedure,^60^ to yield 1,4,5,8,9,10-hexahydroanthracene as clear needle-like crystals.

1,4,5,8,9,10-Hexahydroanthracene: mp = 146–147 °C; *R*_*f*_ = 0.40 (100% hexanes); IR (neat): ν_max_ = 3026, 2878, 2842, 2814, 1674, 985 cm^–1^; ^1^H NMR (300 MHz, CDCl_3_): δ 5.74 (s, 4H), 2.57 (s, 8H), 2.43 (s, 4H). The spectral data match those previously reported.^61^^13^C NMR (75 MHz, CDCl_3_): δ 124.6, 123.5, 36.1, 30.7; HRMS (ESI-TOF+) *m*/*z* for [M + H]^+^ C_14_H_17_, calcd 185.1325, found 185.1319.

#### Reduction of Anthracene (**25**) to 9,10-Dihydroanthracene (**27**)

Using general arene reduction method C with anthracene (356.4 mg, 2.0 mmol), lithium metal (41.6 mg, 6.0 mmol, 3.0 equiv), ethylenediamine (0.8 mL, 12 mmol, 6.0 equiv), *t*-butanol (574 μL, 6.0 mmol, 3.0 equiv), and THF (6.7 mL) performed at 23 °C; reaction crude mixture was extracted with CHCl_3_ instead of Et_2_O; atmosphere and results are shown in Table S18, Spectra S98–S100, and Chromatograms S68–S70.

9,10-Dihydroanthracene: ^1^H NMR (300 MHz, CDCl_3_): δ 7.29–7.23 (m, 4H, overlapping with CHCl_3_ peak), 7.17 (dt, 4H, J = 5.5, 3.3 Hz), 3.92 (s, 4H). The spectral data match those previously reported.^62^

#### Reduction of 9,10-Dihydroanthracene (27) to 1,4,9,10-Tetrahydroanthracene (**28**)

Using general arene reduction method C with 9,10-dihydroanthracene (360.5 mg, 2.0 mmol), lithium metal (41.6 mg, 6.0 mmol, 3.0 equiv), ethylenediamine (0.8 mL, 12 mmol, 6.0 equiv), *t*-butanol (574 μL, 6.0 mmol, 3.0 equiv), and THF (6.7 mL) performed at 23 °C; atmosphere and results are shown in Table S19, Spectra S103–S105, and Chromatograms S71–S73. A portion of the reaction crude mixture was purified by flash column chromatography (SiO_2_, 100% hexanes) to yield a mixture of 1,4,9,10-tetrahydroanthracene (93%) and 1,4,5,8,9,10-hexahydroanthracene (7%) as a white solid.

1,4,9,10-Tetrahydroanthracene: *R*_*f*_ = 0.50 (100% hexanes); ^1^H NMR (300 MHz, CDCl_3_): δ 7.14 (s, 4H), 5.78 (s, 2H), 3.27 (s, 4H), 2.70 (s, 4H); ^13^C NMR (75 MHz, CDCl_3_): δ 134.8, 128.0, 125.9, 124.6, 123.9, 34.8, 30.9; HRMS (ESI-TOF+) *m*/*z* for [M + H]^+^ C_14_H_15_, calcd 183.1168, found 183.1173.

#### Reduction of 1-Methyl-1*H*-indole (**29**) to 1-Methyl-4,7-dihydro-1*H*-indole (**30**)

Using general arene reduction method C with 1-methyl-1*H*-indole (262.3 mg, 2.0 mmol), lithium metal (55.5 mg, 8.0 mmol, 4.0 equiv), ethylenediamine (1.07 mL, 16 mmol, 8.0 equiv), *i*-propanol (460 μL, 6.0 mmol, 3.0 equiv), and THF (6.7 mL); the reaction was carried out for 20 min instead of 1 h; atmosphere and results are shown in Table S20, Spectra S106–S108, and Chromatograms S74–S76.

1-Methyl-4,7-dihydro-1*H*-indole: ^1^H NMR (300 MHz, CDCl_3_): δ 6.52 (d, 1H, *J* = 2.7 Hz), 5.91 (d, 1H, *J* = 2.7 Hz), 5.90–5.83 (m, 1H), 5.83–5.77 (m, 1H), 3.51–3.40 (s, 3H, overlapping with Et_2_O), 3.28–3.15 (m, 4H). The spectral data match those previously reported.^14^

#### Reduction of 1-Methyl-1*H*-indole (**29**) to 1-Methylindoline (**31**)

Using general arene reduction method C with 1-methyl-1*H*-indole (262.3 mg, 2.0 mmol), lithium metal (55.5 mg, 8.0 mmol, 4.0 equiv), ethylenediamine (1.07 mL, 16 mmol, 8.0 equiv), and THF (6.7 mL); the reaction was carried out for 30 min instead of 1 h and no proton donor was used; atmosphere and results are shown in Table S21, Spectra S109–S111, and Chromatograms S77–S79.

1-Methylindoline: ^1^H NMR (300 MHz, CDCl_3_): δ 7.09–7.00 (m, 2H), 6.63 (td, 1H, *J* = 7.4, 0.9 Hz), 6.46 (d, 1H, *J* = 8.0 Hz), 3.26 (t, 2H, *J* = 8.0 Hz), 2.91 (t, 2H, *J* = 8.1 Hz), 2.72 (s, 3H). The spectral data match those previously reported.^32^

#### Reduction of 1*H*-Indole to 4,7-Dihydro-1*H*-indole (**32**)

Using general arene reduction method C with 1*H*-indole (234.3 mg, 2.0 mmol), lithium metal (55.5 mg, 8.0 mmol, 4.0 equiv), ethylenediamine (1.07 mL, 16 mmol, 8.0 equiv), *i*-propanol (460 μL, 6.0 mmol, 3.0 equiv), and THF (6.7 mL); the reaction was carried out for 20 min instead of 1 h; atmosphere and results are shown in Table S22, Spectra S112–S114, and Chromatograms S80–S82.

4,7-Dihydro-1*H*-indole: ^1^H NMR (300 MHz, CDCl_3_): δ 6.65 (t, 1H, *J* = 2.7 Hz), 5.98 (t, 1H, *J* = 2.6 Hz), 5.91–5.75 (m, 2H), 3.29–3.16 (m, 4H). The spectral data match those previously reported.^32^

#### Reduction of Biphenyl (33) to 1,4-Dihydro-1,1′-biphenyl (**34**)

Using general arene reduction method C with biphenyl (308.4 mg, 2.0 mmol), lithium metal (55.5 mg, 8.0 mmol, 4.0 equiv), 1,3-diaminopropane (0.67 mL, 8.0 mmol, 4.0 equiv), phenol (564.7 mg, 6.0 mmol, 3.0 equiv), and THF (6.7 mL); the reaction was carried out for 70 min instead of 1 h; phenol was added with the solid starting material; atmosphere and results are shown in Table S23, Spectra S115–S117, and Chromatograms S83–S85.

1,4-Dihydro-1,1′-biphenyl: ^1^H NMR (300 MHz, CDCl_3_ + 1% CD_3_OD): δ 7.25–7.10 (m, 5H, overlapping with phenol), 5.81–5.73 (m, 2H), 5.72–5.64 (m, 2H), 3.99–3.86 (m, 1H), 2.75–2.67 (m, 2H). The spectral data match those previously reported.^63^

#### Reduction of 3-Phenylphenol (**35**) to 1′,4′-Dihydro-[1,1′-biphenyl]-3-ol (**36**)

Using general arene reduction method C with 3-phenylphenol (340.4 mg, 2.0 mmol), lithium metal (69.4 mg, 10 mmol, 5.0 equiv), ethylenediamine (1.34 mL, 20 mmol, 10 equiv), 1,1,1,3,3,3-hexafluoro-2-propanol (632 μL, 6.0 mmol, 3.0 equiv), and THF (6.7 mL); the reaction crude mixture was extracted with hexanes instead of Et_2_O; atmosphere and results are shown in Table S24, Spectra S120–S122, and Chromatograms S86–S88. A portion of the reaction crude mixture was purified by prep-TLC (20% EtOAc in hexanes) to yield 1′,4′-dihydro-[1,1′-biphenyl]-3-ol as a clear oil.

1′,4′-Dihydro-[1,1′-biphenyl]-3-ol: *R*_*f*_ = 0.47 (20% EtOAc in hexanes); IR (neat): ν_max_ = 3337 (broad, O–H), 3026, 2926, 2856, 2820, 1591, 1454, 1259, 1150 cm^–1^; ^1^H NMR (300 MHz, CDCl_3_): δ 7.18 (t, 1H, *J* = 8.0 Hz), 6.81 (dt, 1H, *J* = 7.6, 1.1 Hz), 6.73–6.64 (m, 2H), 5.87–5.78 (m, 2H), 5.77–5.67 (m, 2H), 4.66 (brs, 1H), 3.97–3.86 (m, 1H), 2.79–2.70 (m, 2H); ^13^C NMR (75 MHz, CDCl_3_): δ 155.9, 147.3, 129.9, 128.5, 124.0, 120.6, 115.0, 113.4, 41.9, 25.9; HRMS (ESI-TOF+) *m*/*z* for [M + H]^+^ C_12_H_13_O, calcd 173.0961, found 173.0962.

## Data Availability

The data underlying this study are available in the published article and its Supporting Information.

## Supporting Information Available

Experimental procedures; control experiments; characterization data; ^1^H and ^13^C NMR spectra, and GC-MS chromatograms and mass spectra (PDF)
FAIR data, including the primary NMR FID files, for compounds 1-methoxyadamantane, **6**, **8**–**36** (ZIP)
