# Supplementary figures and images for: Atmosphere Effects on Arene Reduction with Lithium and Ethylenediamine in THF
Source: J Org Chem. 2025 Mar 3;90(10):3684–97. doi: 10.1021/acs.joc.4c03118 (PMC11915386; doi:10.1021/acs.joc.4c03118)

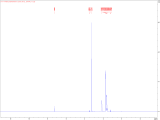

Supplement: Supplementary file 1 — jo4c03118_si_001.zip [file jo4c03118_si_001.zip › FID for Publication/1-methoxyadamantane/1H/pdata/1/thumb.png]
