# Supplementary material for: Atmosphere Effects on Arene Reduction with Lithium and Ethylenediamine in THF
Source: J Org Chem. 2025 Mar 3;90(10):3684–97. doi: 10.1021/acs.joc.4c03118 (PMC11915386; doi:10.1021/acs.joc.4c03118)
Supplement: Supplementary file 2 — jo4c03118_si_002.pdf [file jo4c03118_si_002.pdf]

Supporting Information

for

Atmosphere effects on arene reduction with lithium and ethylenediamine in THF

Zachary S. Shellnutt and Kazunori Koide*

Department of Chemistry, University of Pittsburgh

219 Parkman Avenue, Pittsburgh, Pennsylvania 15260, United States

koide@pitt.edu

Table of Contents

Tables	S2
Discussion about UV-VIS Absorbance Study	S11
Discussion about Possible Lithium Amide Inhibition	S12
Comparison of Reduction Conditions for 2-Methyl- and 2-Methoxynaphthalene	S13
Gas Chromatograms and Mass Spectra	S14
NMR Spectra	S116
Cited References	S217

Table S1. Reduction of naphthalene (**8**) under Ar using the general kinetic method (Figure 2a**)**.

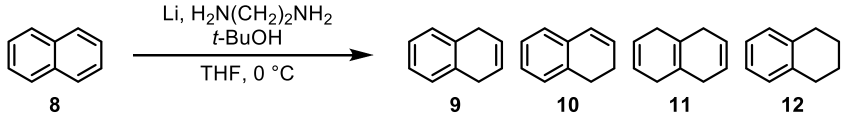

entry	time (min)	% yield<br>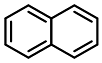<br>**8**	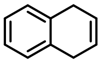<br>**9**	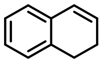<br>**10**	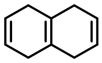<br>**11**	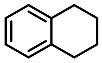<br>**12**
1	0	100	0	0	0	0
2	5	37	29	0	25	6
3	10	14	30	6	40	10
4	15	10	10	24	41	15
5	30	9	<1	31	41	18
6	45	9	<1	31	42	16
7	60	9	<1	31	41	16

Percent yield determined by ^{1}H NMR.

Table S2. Reduction of naphthalene (**8**) under O_2 using the general kinetic method (Figure 2b**)**.

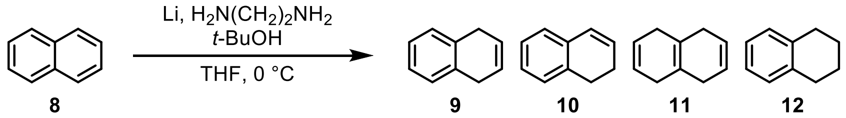

entry	time (min)	% yield<br>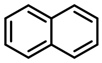<br>**8**	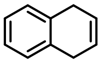<br>**9**	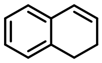<br>**10**	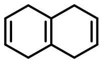<br>**11**	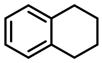<br>**12**
1	0	100	0	0	0	0
2	5	28	32	<1	29	8
3	10	17	33	<1	36	9
4	15	18	34	<1	37	11
5	30	17	32	<1	35	9
6	45	16	31	<1	35	9
7	60	16	31	<1	34	9

Percent yield determined by ^{1}H NMR.

Table S3. Reduction of naphthalene (**8**) using sub- and superstoichiometric amounts of reductant (Table 3).

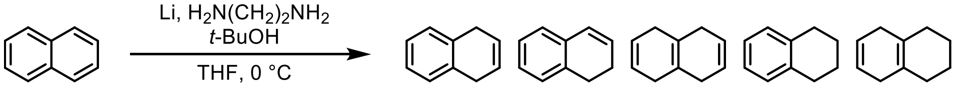

8 **9** **10** **11** **12** **13**

					% yield					
entry	Li (equiv)	en (equiv)	atm	1-methoxy-adamantane (mg)	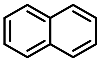 **8**	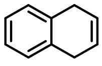 **9**	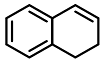 **10**	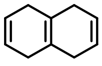 **11**	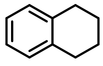 **12**	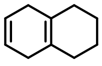 **13**
1	2.0	4.0	Ar	42.5	31	24	*N.D.*	24	6	*N.D.*
2	2.0	4.0	O_2	40.7	34	21	*N.D.*	21	6	*N.D.*
3	5.0	10.0	Ar	38.7	*N.D.*	*N.D.*	*N.D.*	27[a,b]	31	21[a,b]
4	5.0	10.0	O_2	45.5	<2	9	*N.D.*	51[a,c]	10	13[a,c]

[a]Percent yield determined by ^{1}H NMR may not be accurate due to peak overlaps. [b]See Chromatogram S33. [c]See Chromatogram S34. Percent yield determined by ^{1}H NMR. *N.D.* = not detected. en = ethylenediamine.

Table S4. Reduction of naphthalene (**8**) using the general KO_2 method (Table 4).

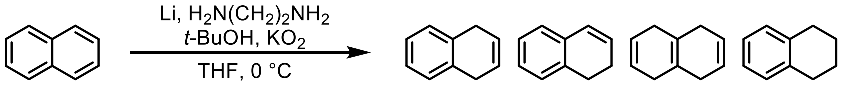

8 **9** **10** **11** **12**

			% yield				
entry	internal standard (mg)	KO_2 (equiv)	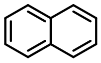 **8**	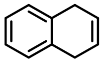 **9**	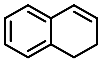 **10**	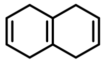 **11**	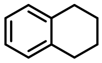 **12**
1	38.1	0.10	10	*N.D.*	30	36	10[a]
2	50.5	0.25	10	*N.D.*	32	39	9[a]
3	43.8	0.50	9	*N.D.*	31	37	10[a]
4	46.2	1.00	11	3	29	35	8[a]
5	58.3	2.00	11	4	29	39	8[a]
6	53.8	5.00	9	19	11	37	7[a]
7	68.2	0	13	31	*N.D.*	42	8

[a]Percent yield determined by ^{1}H NMR may not be accurate due to peak overlaps. Percent yield determined by ^{1}H NMR. *N.D.* = not detected.

Table S5. Reduction of 2-methylnaphthalene under various atmospheres.

entry	*t*-BuOH (equiv)	atm	1-methoxy-adamantane (mg)	% yield	
				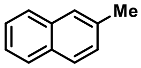	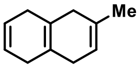 **14**
1	3.0	Ar	33.4	*N.D.*	80
2	3.0	air	36.0	*N.D.*	68
3	3.0	O_2	35.7	<1	68
4	5.0	Ar	48.4	*N.D.*	96
5	5.0	air	49.7	<1	89
6	5.0	O_2	50.5	5	83

Percent yield determined by ^{1}H NMR. *N.D.* = not detected.

Table S6. Reduction of 2-methoxynaphthalene under various atmospheres.

entry	*t*-BuOH (equiv)	atm	internal standard (mg)	% yield	
				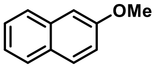	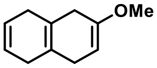 **15**
1	3.0	Ar	MAD (45.4)	3	37
2	3.0	air	MAD (42.7)	4	45
3	3.0	O_2	MAD (37.4)	5	67
4	5.0	Ar	TMB (41.1)	3	85
5	5.0	air	TMB (43.2)	4	82
6	5.0	O_2	TMB (39.0)	9	78

Percent yield determined by ^{1}H NMR. MAD = 1-methoxyadamantane. TMB = 1,3,5-trimethoxybenzene.

Table S7. Reduction of naphthalene (**8**) under various atmospheres.

entry	atm	1,3,5-trimethoxybenzene (mg)	% yield		
			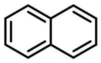 **8**	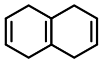 **11**	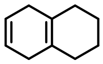 **13**
1	Ar	31.4	*N.D.*	77	21
2	air	37.8	1	77	17
3	O_2	34.3	4	73	17

Percent yield determined by ^{1}H NMR. *N.D.* = not detected.

Table S8. Reduction of 2-(naphthalen-2-yl)acetic acid under various atmospheres.

entry	atm	1,3,5-trimethoxybenzene (mg)	% yield 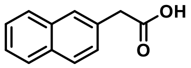	% yield 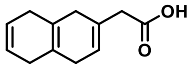 **16**
1	Ar	30.3	14	70
2	air	35.2	13	70
3	O_2	43.8	13	58

Percent yield determined by ^{1}H NMR.

Table S9. Reduction of 1-methoxynaphthalene under various atmospheres. Percent yields were determined by ^{1}H NMR.

entry	atm	1,3,5-trimethoxybenzene (mg)	% yield 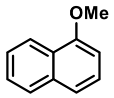	% yield 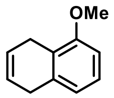 **17**
1	Ar	41.6	24	38
2	air	34.3	23	38
3	O_2	34.6	31	27

Percent yield determined by ^{1}H NMR.

Table S10. Reduction of 1-methylnaphthalene under various atmospheres.

entry	atm	1,3,5-trimethoxybenzene (mg)	% yield 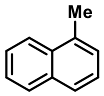	% yield 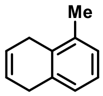 **18**
1	Ar	38.4	5	54
2	air	33.7	8	54
3	O_2	45.2	10	56

Percent yield determined by ^{1}H NMR.

Table S11. Reduction of 1-naphthoic acid under various atmospheres.

entry	atm	1-methoxy-adamantane (mg)	% yield	
			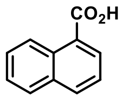	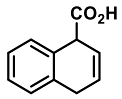 **19**
1	Ar	31.8	<1	83
2	air	36.8	70	*N.D.*
3	O_2	36.0	63	*N.D.*

Percent yield determined by ^{1}H NMR. *N.D.* = not detected.

Table S12. Reduction of under various atmospheres.

entry	atm	1-methoxy-adamantane (mg)	% yield	
			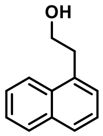	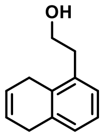 **20**
1	Ar	37.3	23	32
2	air	36.3	27	34
3	O_2	47.9	38	33

Percent yield determined by ^{1}H NMR.

Table S13. Reduction of 1-methylnaphthalene under various atmospheres.

entry	atm	1-methoxy-adamantane (mg)	% yield	
			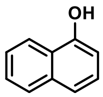	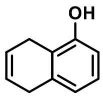 **21**
1	Ar	29.6	5	63
2	air	32.8	12	60
3	O_2	36.3	15	59

Percent yield determined by ^{1}H NMR.

Table S14. Reduction of 2-naphthol under various atmospheres.

			% yield	
entry	atm	1-methoxy-adamantane (mg)	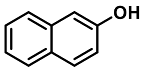	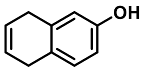 **22**
1	Ar	41.4	14	32
2	air	50.3	22	31
3	O_2	61.5	27	30

Percent yield determined by ^{1}H NMR.

Table S15. Reduction of 1-aminonaphthalene under various atmospheres.

			% yield	
entry	atm	1,3,5-trimethoxybenzene (mg)	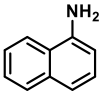	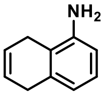 **23**
1	Ar	40.3	2	74
2	air	40.5	6	74
3	O_2	44.5	9	74

Percent yield determined by ^{1}H NMR.

Table S16. Reduction of 2,3-naphthalenediol under various atmospheres.

			% yield		
entry	atm	1,3,5-trimethoxybenzene (mg)	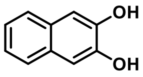	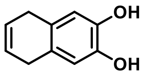 **24**	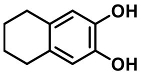
1	Ar	42.7	37	29	29
2	air	60.4	45	20	23
3	O_2	51.1	39	16	20

Percent yield determined by ^{1}H NMR.

Table S17. Reduction of anthracene under various atmospheres.

entry	atm	1-methoxy-adamantane (mg)	% yield		
			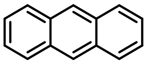 **25**	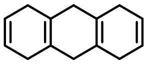 **26**	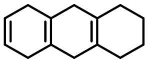
1	Ar	47.3	<1	73[a]	21[a]
2	air	30.2	3	67[a]	19[a]
3	O_2	38.0	7	60[a]	18[a]

[a]Percent yield determined by ^{1}H NMR may not be accurate due to peak overlaps. Percent yield determined by ^{1}H NMR.

Table S18. Reduction of anthracene under various atmospheres.

entry	atm	1-methoxy-adamantane (mg)	% yield		
			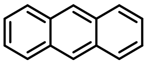 **25**	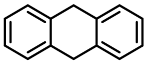 **27**	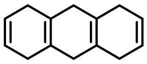 **26**
1	Ar	33.2	34	12	25
2	air	45.7	44	9	22
3	O_2	51.5	45	7	26

Percent yield determined by ^{1}H NMR.

Table S19. Reduction of 9,10-dihydroanthracene under various atmospheres.

entry	atm	1,3,5-trimethoxybenzene (mg)	% yield			
			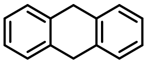 **27**	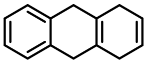 **28**	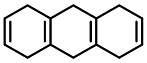 **26**	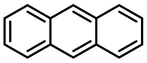 **25**
1a	Ar	34.2	16	34	45	*N.D.*
2	air	37.5	14	38	42	1
3	O_2	34.5	16	35	40	3

Percent yield determined by ^{1}H NMR. *N.D.* = not detected.

Table S20. Reduction of 1-methyl-1*H*-indole under various atmospheres.

			% yield		
entry	atm	1,3,5-trimethoxybenzene (mg)	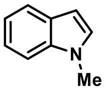 **29**	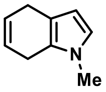 **30**	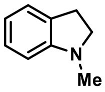 **31**
1	Ar	42.7	*N.D.*	45	4
2	air	42.4	1	43	10
3	O_2	55.1	5	40	16

Percent yield determined by ^{1}H NMR. *N.D.* = not detected.

Table S21. Reduction of 1-methyl-1*H*-indole under various atmospheres.

			% yield	
entry	atm	1,3,5-trimethoxybenzene (mg)	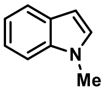 **29**	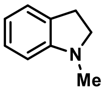 **31**
1	Ar	44.4	1	66
2	air	44.2	4	74
3	O_2	39.8	24	58

Percent yield determined by ^{1}H NMR.

Table S22. Reduction of 1*H*-indole under various atmospheres.

			% yield	
entry	atm	1,3,5-trimethoxybenzene (mg)	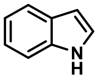	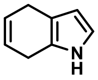 **32**
1	Ar	44.9	3	59
2	air	57.4	19	44
3	O_2	55.5	30	34

Percent yield determined by ^{1}H NMR.

Table S23. Reduction of biphenyl under various atmospheres.

entry	atm	1,3,5-trimethoxybenzene (mg)	% yield 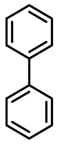 **33**	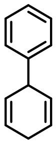 **34**
1	Ar	48.0	10	75
2	air	57.8	24	61
3	O_2	53.0	10	63

Percent yield determined by ^{1}H NMR.

Table S24. Reduction of 3-phenylphenol under various atmospheres.

entry	atm	1-methoxy-adamantane (mg)	% yield 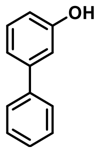 **35**	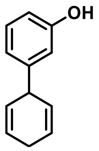 **36**
1	Ar	62.0	8	42
2	air	64.7	27	34
3	O_2	57.8	38	29

Percent yield determined by ^{1}H NMR.

Discussion about UV-VIS Absorbance Study

Inspired by the work of Thompson and Kleinberg,[1] we attempted to measure the UV-Vis spectrum of the hypothesized lithium superoxide formed during the reduction of arenes under an oxygen-containing atmosphere. In a 2-dram vial under an ambient atmosphere, THF (2.7 mL, purged with O_2 for 15 min) and ethylenediamine (0.3 mL) were added via syringe. Lithium metal (17 mg) was added to the solution and placed in a sonicator at 23 °C for 15 min. The reaction solution was removed and a photo was taken as shown in Figure S1. We did not observe the "lemon-yellow solution" seen by Thompson and Kleinberg but observed the formation of a grey suspension with a blue solution generated in the proximity of the lithium metal pieces, which is speculated to be the typical electride complex (dissolved electron) seen in traditional liquid ammonia Birch reactions. During filtration, the solution degraded, and there were no observable bands in the UV-Vis spectrum. It is known that alkali metal superoxides generally have limited solubility in organic solvents and limited stability in protic solvents.[2] Unlike other superoxide sources, lithium superoxide is difficult to characterize due to its fleeting half-life at room temperature, and previous attempts have been done at cryogenic temperatures.[3] It is speculated that at room temperature in the THF/ethylenediamine solution, lithium superoxide is not well stabilized and immediately degrades/reacts similarly to what has been shown with NH_3.[3]

Interestingly, the blue solution produced on the surface of the lithium metal shows that some form of dissolved electron complex is made under an ambient atmosphere at room temperature before degrading into the perceived grey suspension. The grey suspension could be insoluble lithium salts or micro particles of the lithium metal pulverized from sonication.

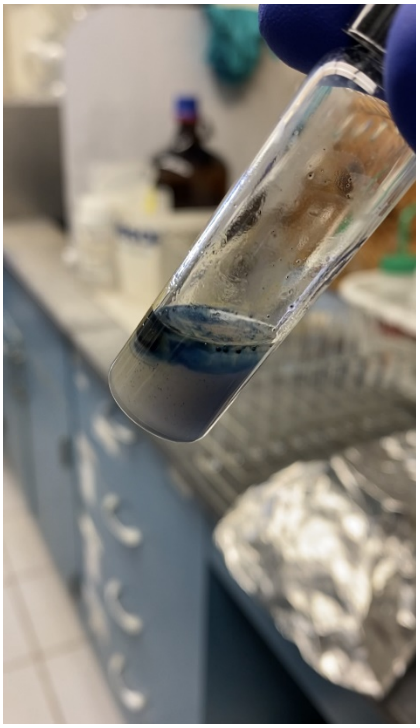

Figure S1. Photograph of lithium oxidation with dioxygen.

Discussion about Possible Lithium Amide Inhibition

After the formation of lithium (2-aminoethyl)amide (**A**), O_2 could oxidize **A** to **B** (Figure S2), and the LiO_2 would quickly degrade. This oxidation step could explain the reduced basicity of the reaction solution preventing isomerization. This type of reaction has been shown for lithium dialkylamides where electrochemical oxidation is heavily dependent on aggregation.[4] This could explain the suspended material shown in Figure S1. Similar oxidations have been shown for secondary and tertiary amines that would lead to the formation of iminium cations.[5-7]

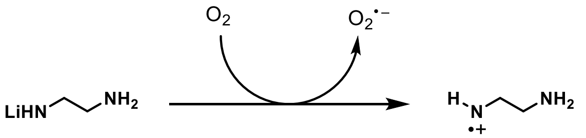

A B

Figure S2. Possible mechanism for lithium amide degradation.

Comparison of Reduction Conditions for 2-Methyl- and 2-Methoxynaphthalene

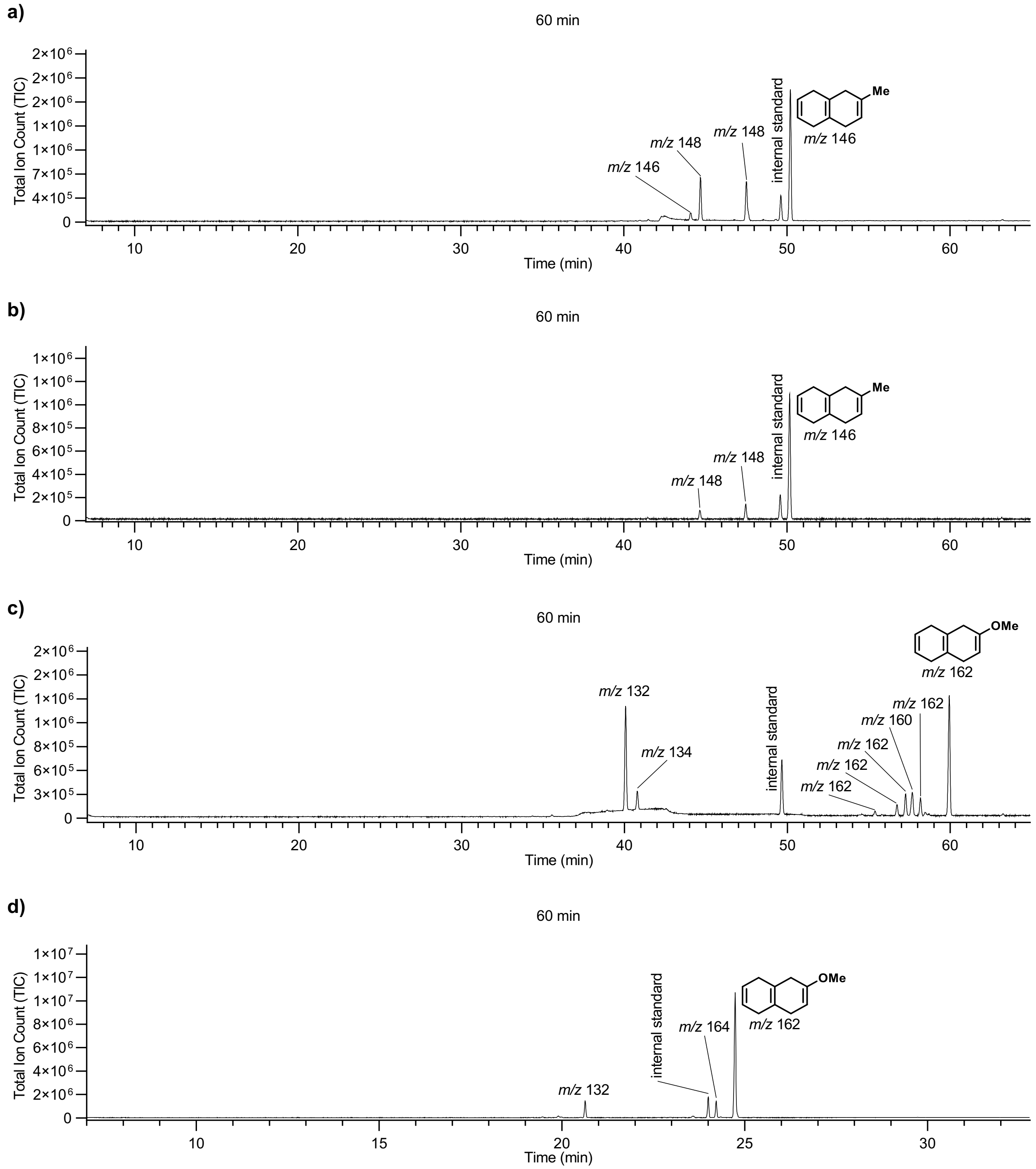

Figure S3. Reduction of 2-methylnaphthalene using a) 6.0 mmol of *t*-BuOH and b) 10 mmol of *t*-BuOH. Reduction of 2-methoxynaphthalene using c) 6.0 mmol of *t*-BuOH and d) 10 mmol of *t*-BuOH. Reaction condition: arene (2.0 mmol), lithium (10 mmol), ethylenediamine (20 mmol), *t*-BuOH (see above), THF (6.7 mL), 0 °C, argon (1 atm), 1 h. For full reaction conditions and data sets, see Table S5 and S6, respectively.

Gas Chromatograms and Mass Spectra

GC-MS method A

Chromatogram S1. Reduction of toluene (**1**) under Ar using general method A (Table 1, entry 1).

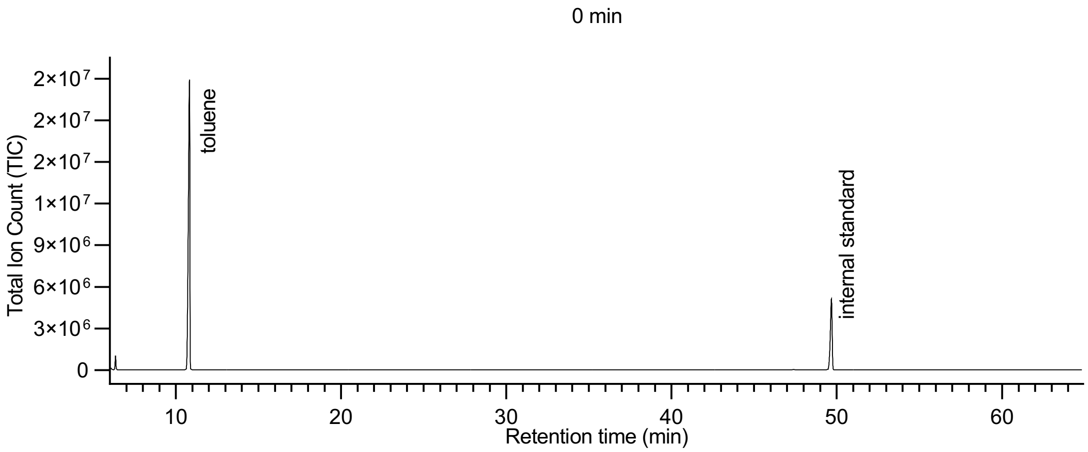

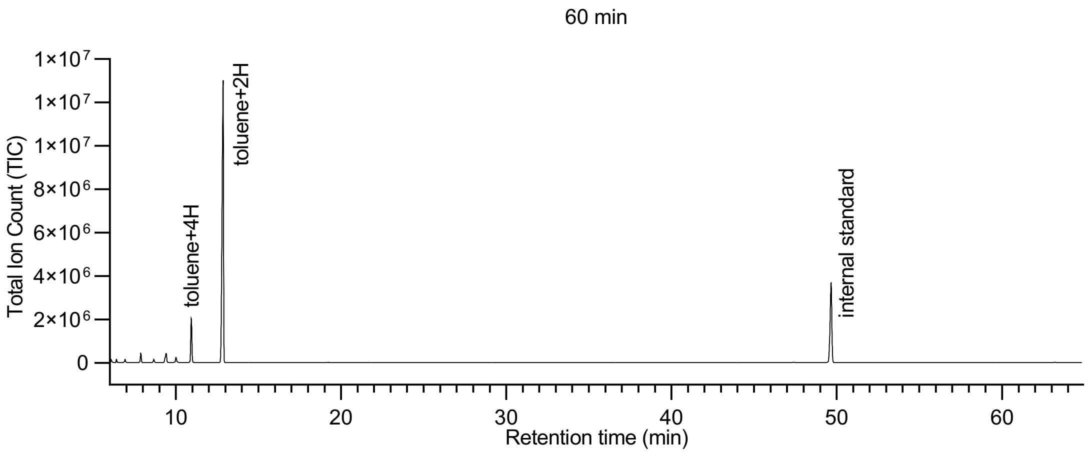

Table S25. Reduction of toluene (**1**) under Ar using general method A (Table 1, entry 1).

reaction time	0 min		60 min	
peak	area	retention time (min)	area	retention time (min)
toluene	132322145	10.83		
toluene+2H			77586845	12.86
toluene+4H			9302694	10.94
internal standard	39206789	49.68	26877519	49.66

Spectrum S1. Mass spectrums of toluene (**1**) and reduction products from Chromatogram S1.

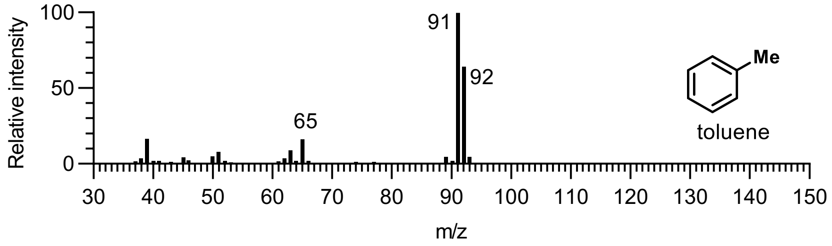

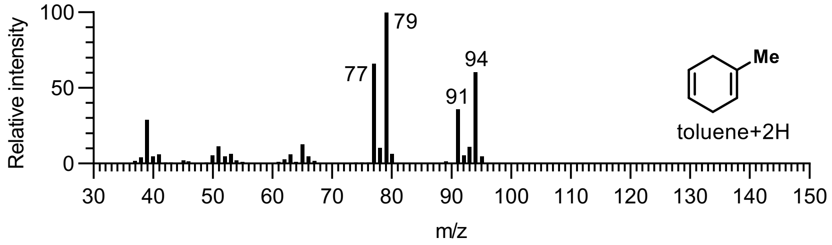

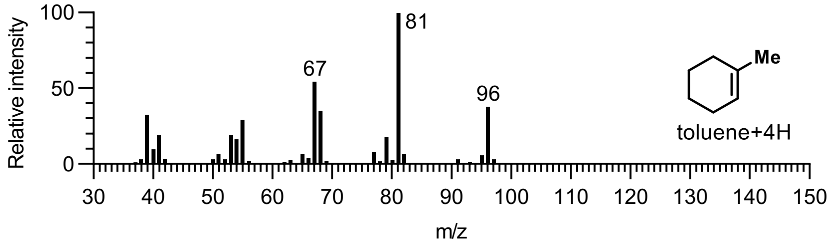

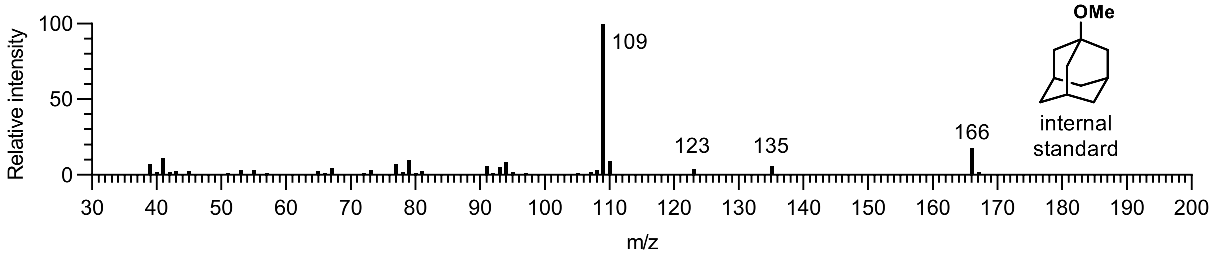

GC-MS method A

Chromatogram S2. Reduction of toluene (**1**) under N_2 using general method A (Table 1, entry 1).

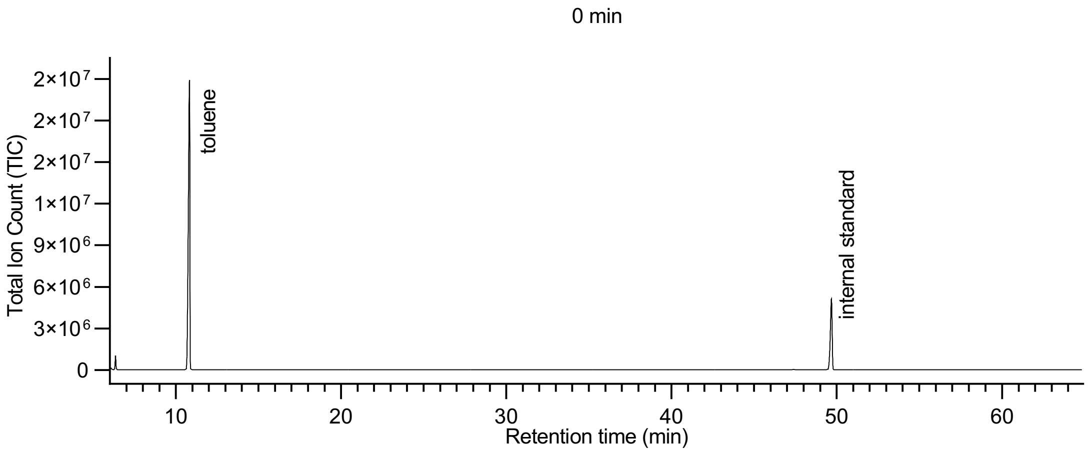

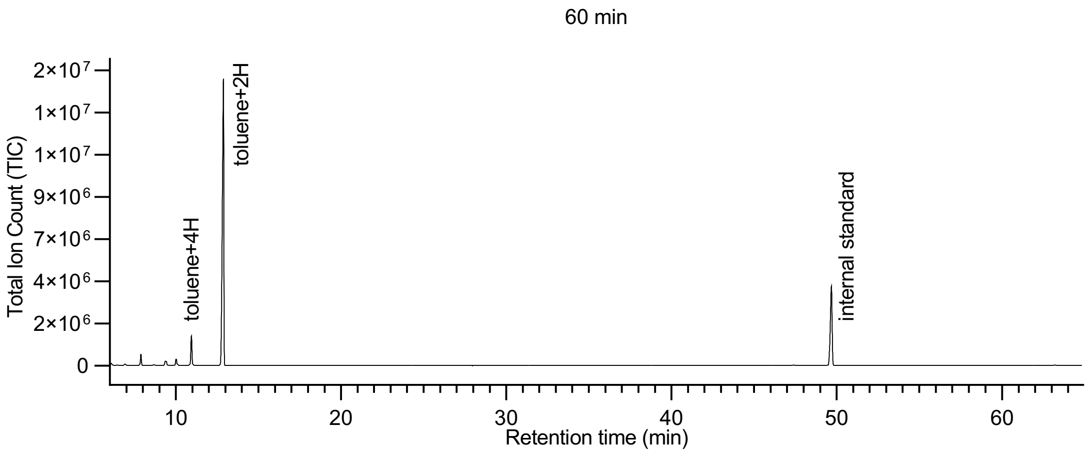

Table S26. Reduction of toluene (**1**) under N_2 using general method A (Table 1, entry 1).

reaction time	0 min		60 min	
peak	area	retention time (min)	area	retention time (min)
toluene	132322145	10.83		
toluene+2H			93502844	12.88
toluene+4H			7057890	10.94
internal standard	39206789	49.68	31089500	49.68

GC-MS method A

Chromatogram S3. Reduction of toluene (**1**) under air using general method B (Table 1, entry 1).

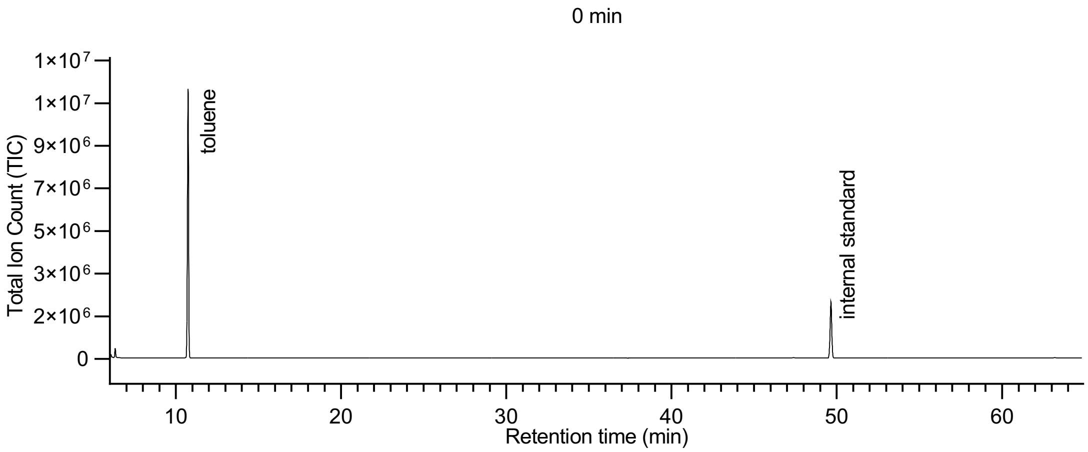

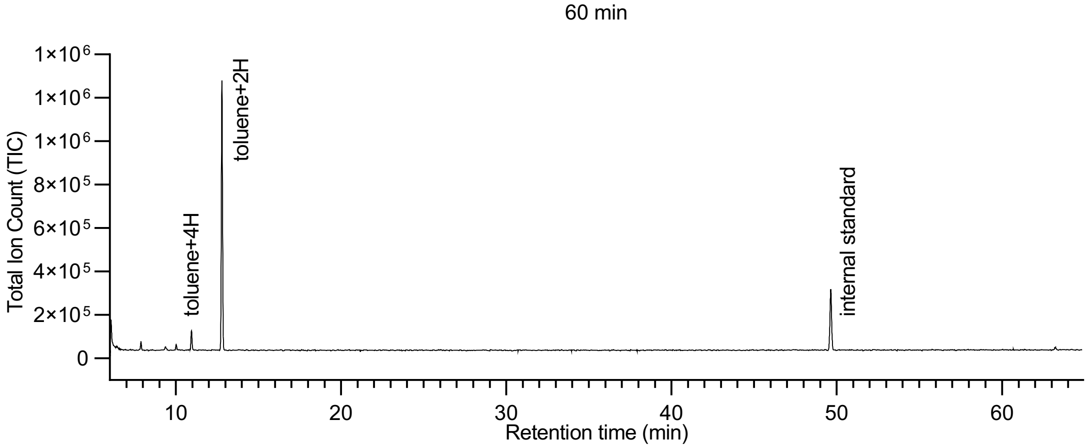

Table S27. Reduction of toluene (**1**) under air using general method B (Table 1, entry 1).

reaction time	0 min		60 min	
peak	area	retention time (min)	area	retention time (min)
toluene	54083429	10.74		
toluene+2H			6323534	12.79
toluene+4H			422733	10.94
internal standard	15818632	49.65	1956449	49.64

GC-MS method A

Chromatogram S4. Reduction of toluene (**1**) under O_2 using general method B (Table 1, entry 1).

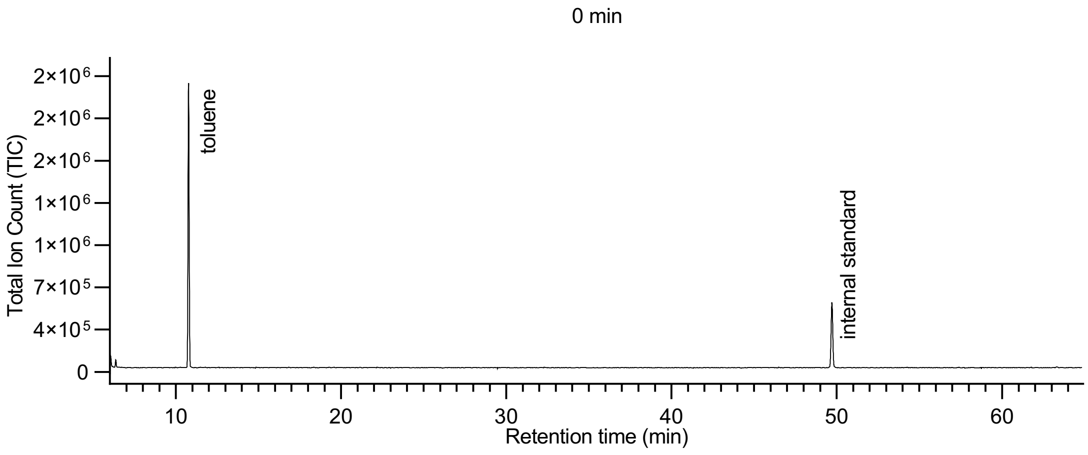

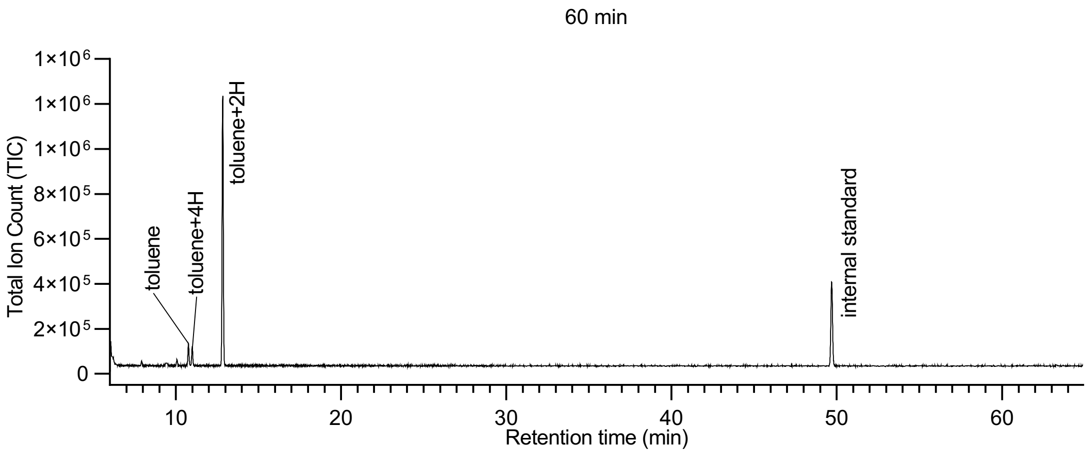

Table S28. Reduction of toluene (**1**) under O_2 using general method B (Table 1, entry 1).

reaction time	0 min		60 min	
peak	area	retention time (min)	area	retention time (min)
toluene	11236738	10.77	461745	10.76
toluene+2H			6054958	12.84
toluene+4H			388969	11.00
internal standard	3833139	49.71	2646783	49.70

GC-MS method A

Chromatogram S5. Reduction of *o*-xylene (**2**) under Ar using general method A (Table 1, entry 2).

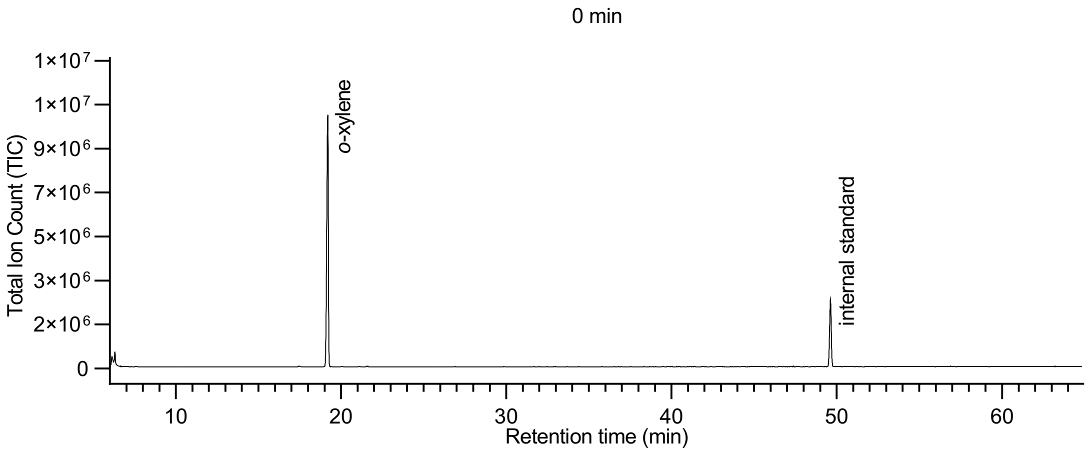

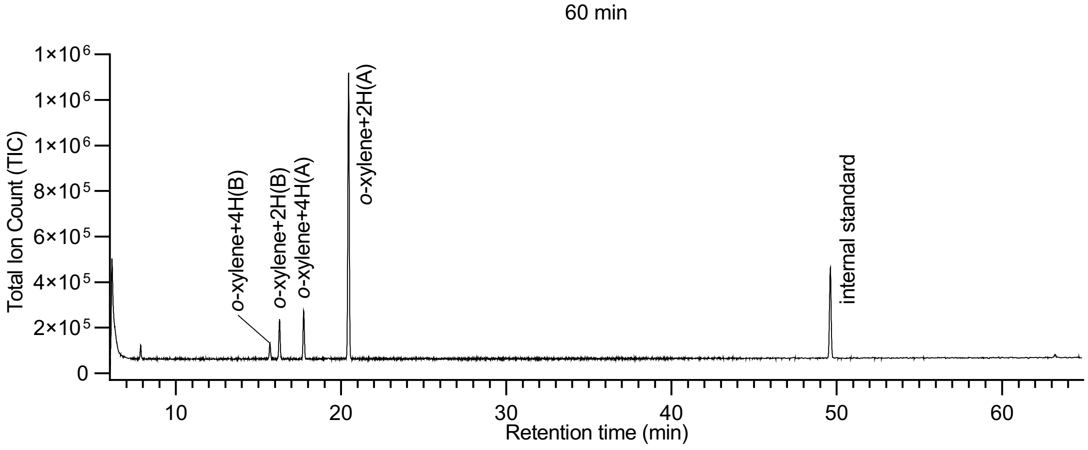

Table S29. Reduction of *o*-xylene (**2**) under Ar using general method A (Table 1, entry 2).

reaction time	0 min		60 min	
peak	area	retention time (min)	area	retention time (min)
o-xylene	64232298	19.20		
o-xylene+2H(A)			7425260	20.46
o-xylene+2H(B)			928309	16.28
o-xylene+4H(A)			1191260	17.74
o-xylene+4H(B)			400481	15.70
internal standard	18330675	49.62	2746975	49.62

Spectrum S2. Mass spectrums of *o*-xylene (**2**) and reduction products from Chromatogram S5.

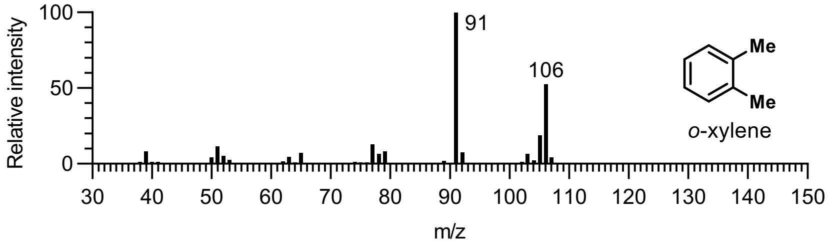

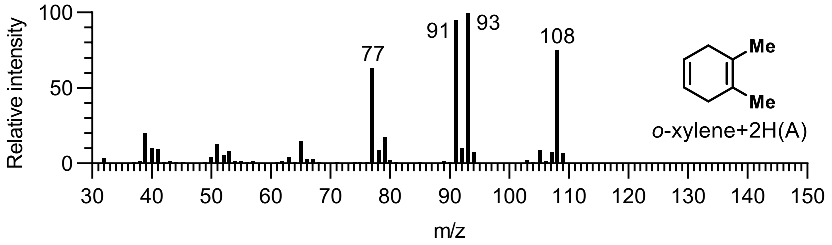

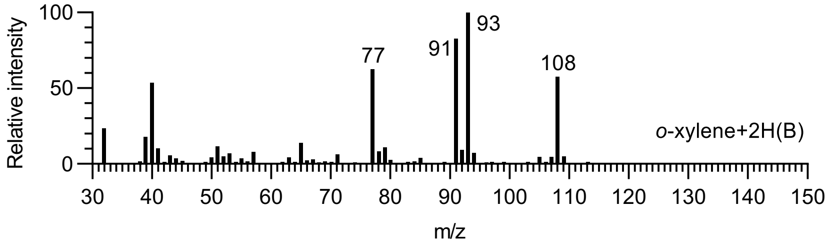

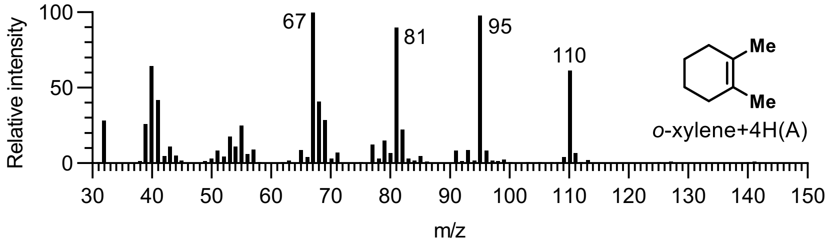

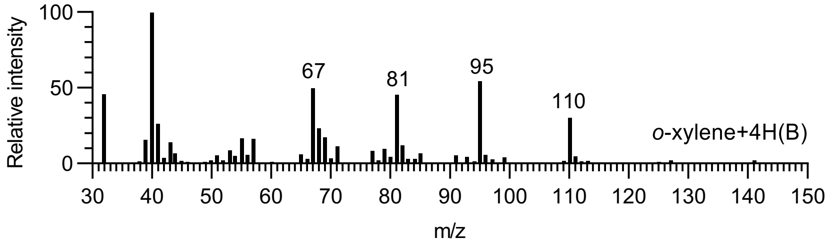

GC-MS method A

Chromatogram S6. Reduction of *o*-xylene (**2**) under N_2 using general method A (Table 1, entry 2).

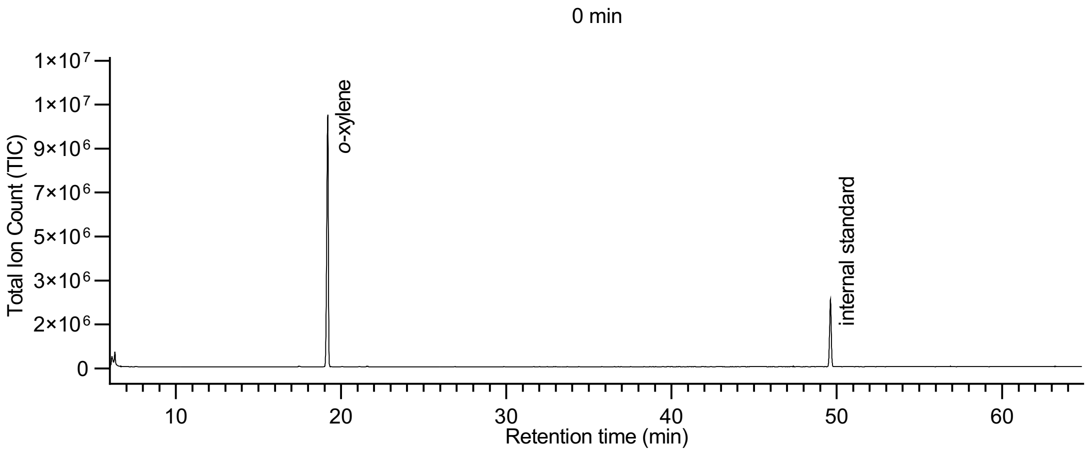

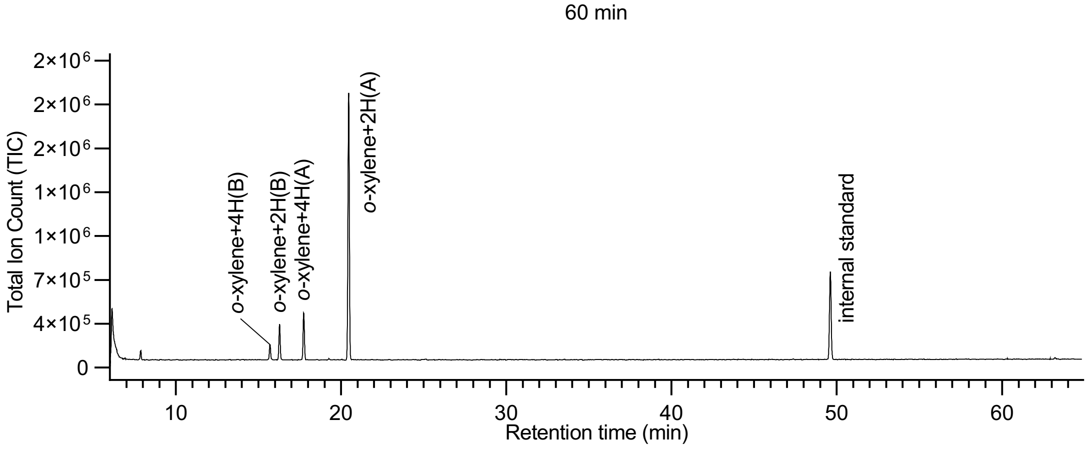

Table S30. Reduction of *o*-xylene (**2**) under N_2 using general method A (Table 1, entry 2).

reaction time	0 min		60 min	
peak	area	retention time (min)	area	retention time (min)
o-xylene	64232298	19.20		
o-xylene+2H(A)			12668997	20.46
o-xylene+2H(B)			1527876	16.28
o-xylene+4H(A)			2074727	17.74
o-xylene+4H(B)			725647	15.70
internal standard	18330675	49.62	4972945	49.61

GC-MS method A

Chromatogram S7. Reduction of *o*-xylene (**2**) under air using general method B (Table 1, entry 2).

0 min

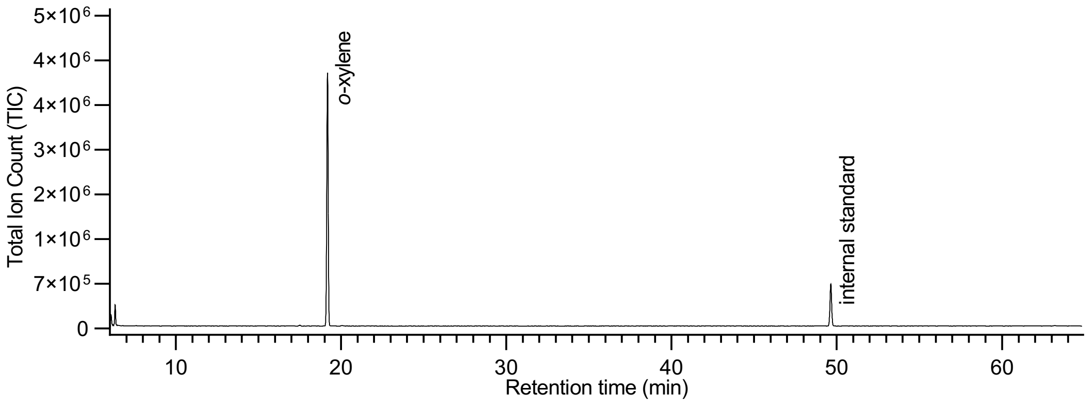

60 min

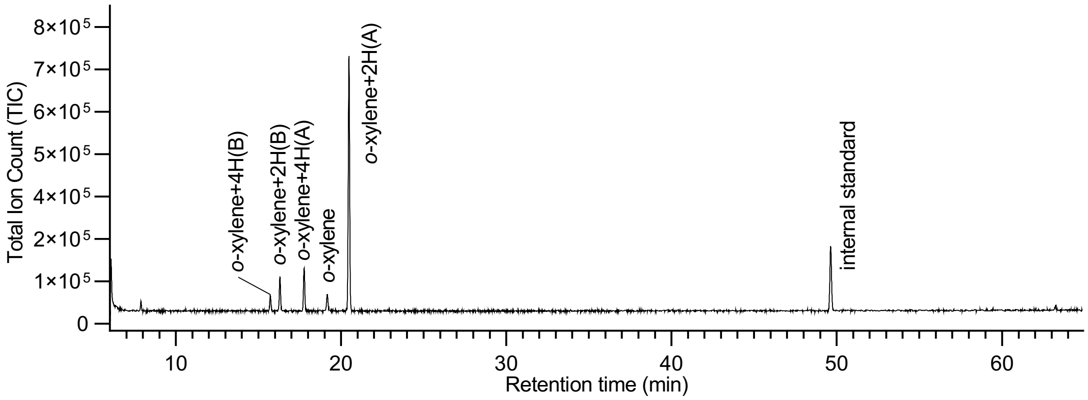

Table S31. Reduction of *o*-xylene (**2**) under air using general method B (Table 1, entry 2).

reaction time	0 min		60 min	
peak	area	retention time (min)	area	retention time (min)
o-xylene	23476691	19.18	274575	19.17
o-xylene+2H(A)			4260004	20.48
o-xylene+2H(B)			509175	16.30
o-xylene+4H(A)			688475	17.77
o-xylene+4H(B)			220416	15.72
internal standard	4706916	49.64	1314304	49.63

GC-MS method A

Chromatogram S8. Reduction of *o*-xylene (**2**) under O_2 using general method B (Table 1, entry 2).

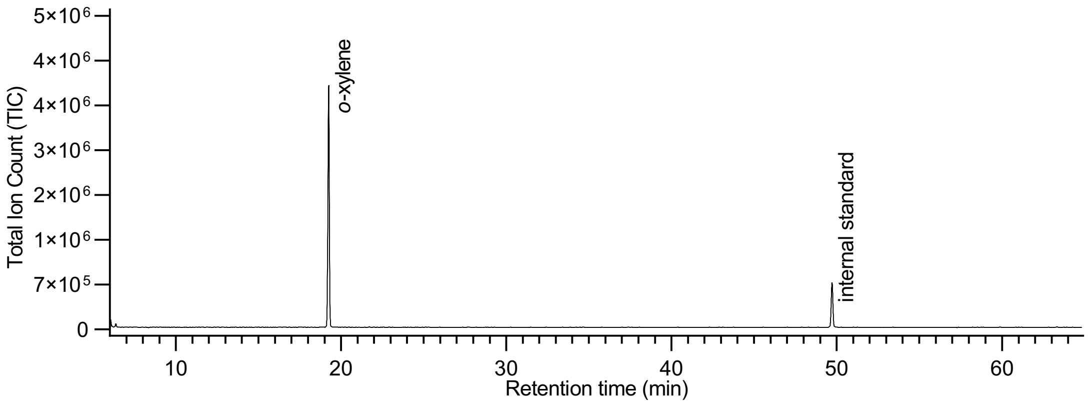

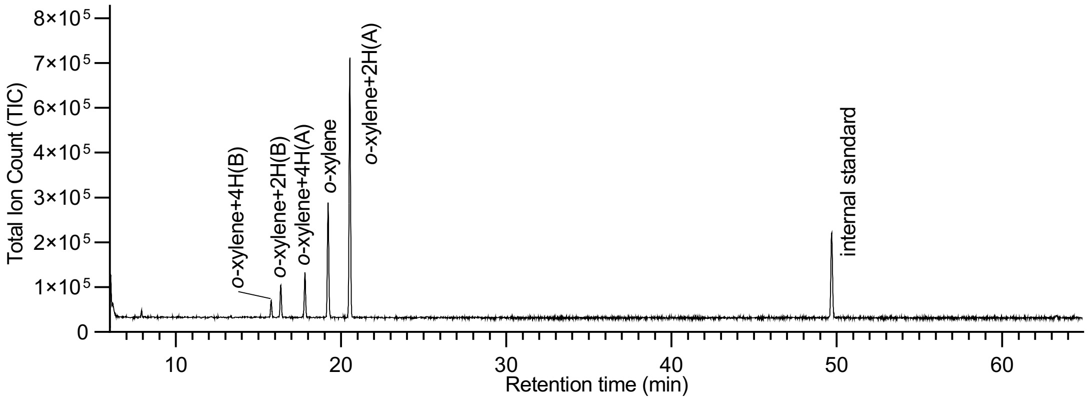

Table S32. Reduction of *o*-xylene (**2**) under O_2 using general method B (Table 1, entry 2).

reaction time	0 min		60 min	
peak	area	retention time (min)	area	retention time (min)
o-xylene	22436568	19.26	1667647	19.22
o-xylene+2H(A)			3709130	20.54
o-xylene+2H(B)			430897	16.35
o-xylene+4H(A)			618896	17.81
o-xylene+4H(B)			202836	15.77
internal standard	4945272	49.71	1437526	49.69

GC-MS method A

Chromatogram S9. Reduction of *p*-cymene (**3**) under Ar using general method A (Table 1, entry 3).

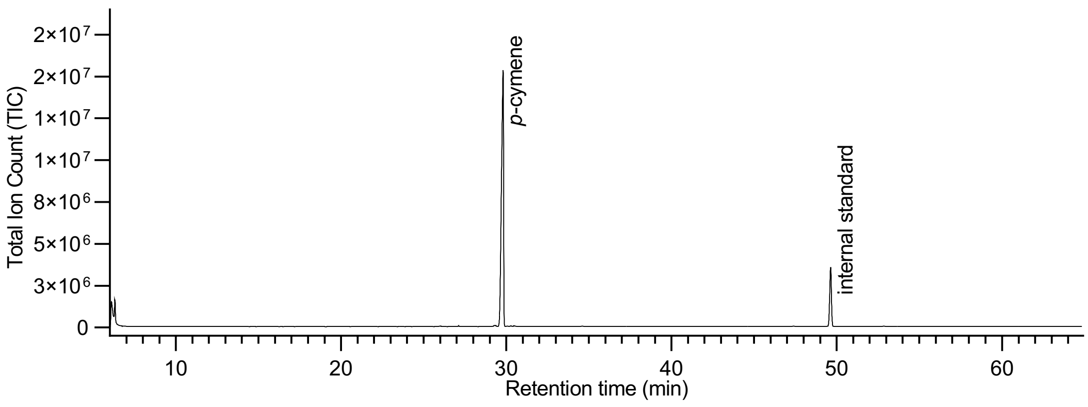

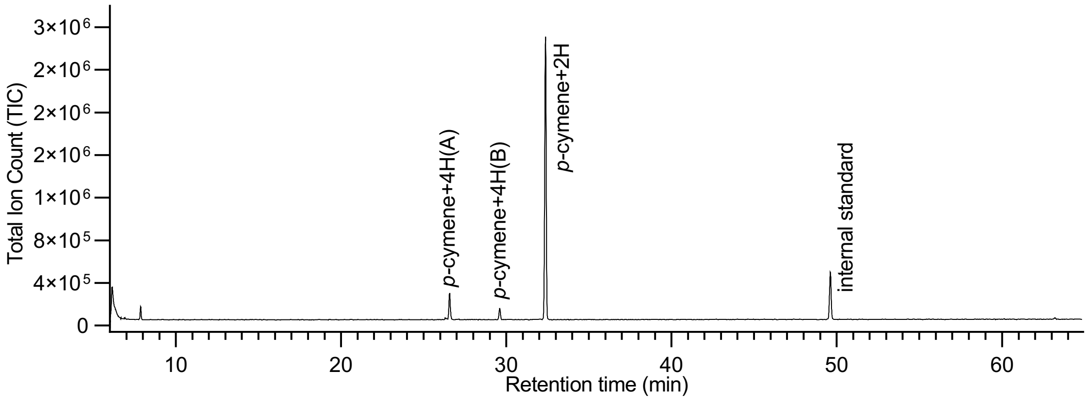

Table S33. Reduction of *p*-cymene (**3**) under Ar using general method A (Table 1, entry 3).

reaction time	0 min		60 min	
peak	area	retention time (min)	area	retention time (min)
p-cymene	138140711	29.80		
p-cymene+2H			16483374	32.34
p-cymene+4H(A)			1489275	26.57
p-cymene+4H(B)			635725	29.60
internal standard	25880407	49.63	3085862	49.61

Spectrum S3. Mass spectrums of *p*-cymene (**3**) and reduction products from Chromatogram S9.

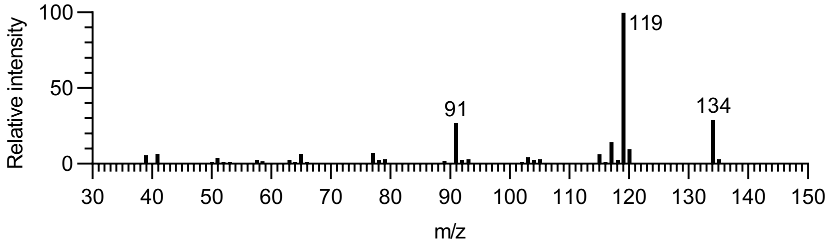

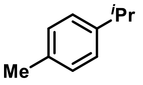

p-cymene

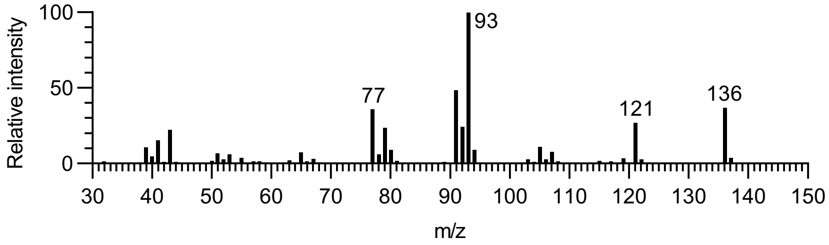

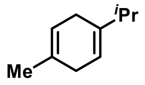

p-cymene+2H

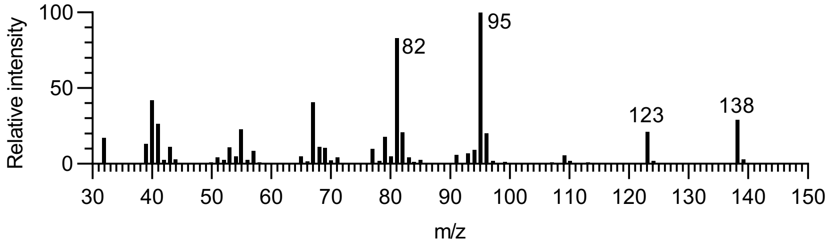

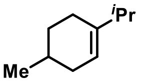

p-cymene+4H(A)

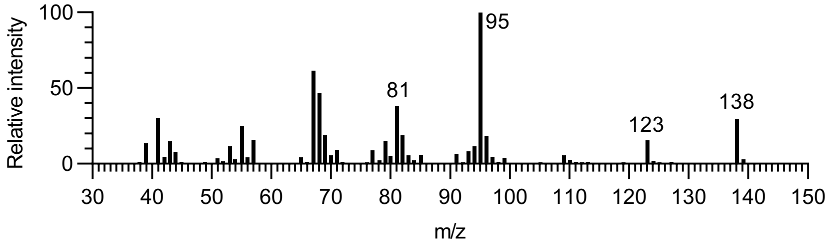

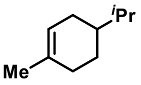

p-cymene+4H(B)

GC-MS method A

Chromatogram S10. Reduction of *p*-cymene (**3**) under N_2 using general method A (Table 1, entry 3).

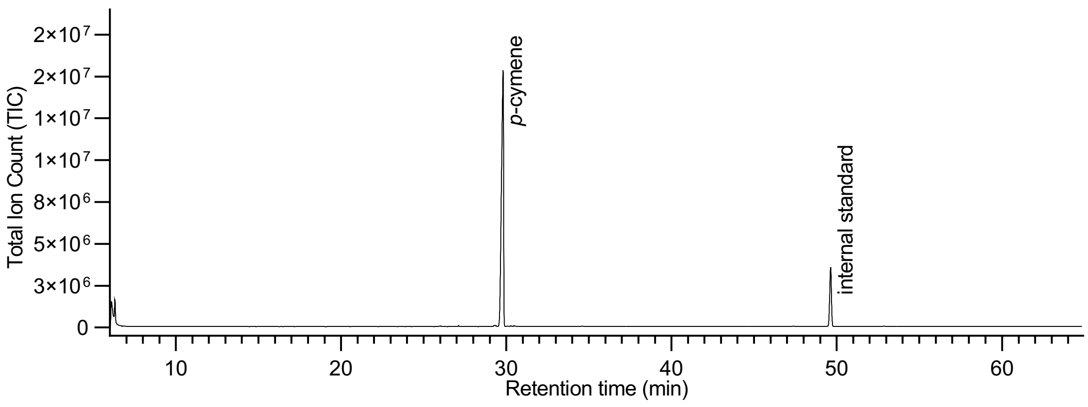

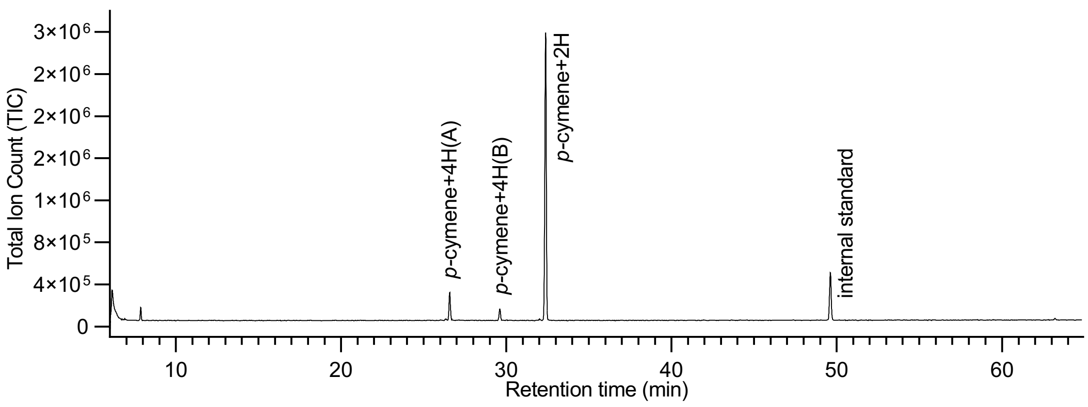

Table S34. Reduction of *p*-cymene (**3**) under N_2 using general method A (Table 1, entry 3).

reaction time	0 min		60 min	
peak	area	retention time (min)	area	retention time (min)
p-cymene	138140711	29.80		
p-cymene+2H			16946594	32.38
p-cymene+4H(A)			1608894	26.58
p-cymene+4H(B)			699482	29.61
internal standard	25880407	49.63	3199394	49.62

GC-MS method A

Chromatogram S11. Reduction of *p*-cymene (**3**) under air using general method B (Table 1, entry 3).

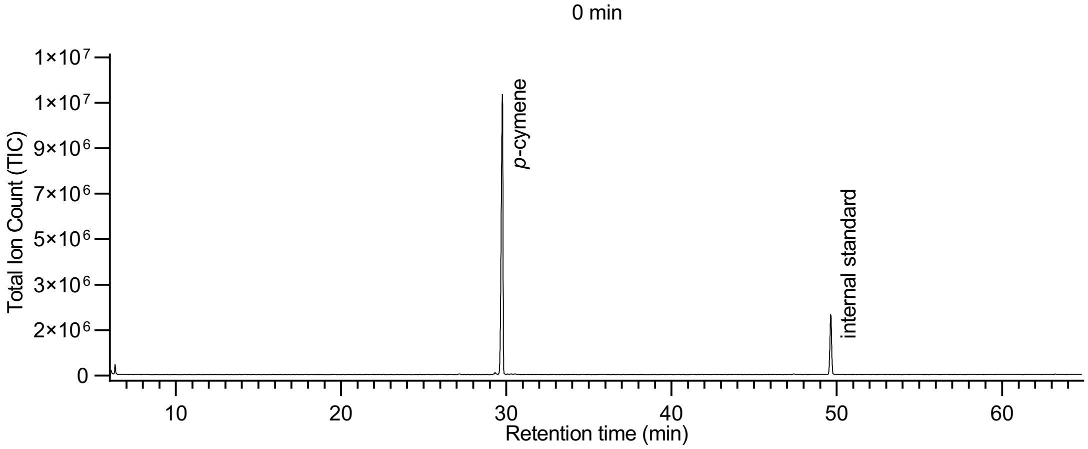

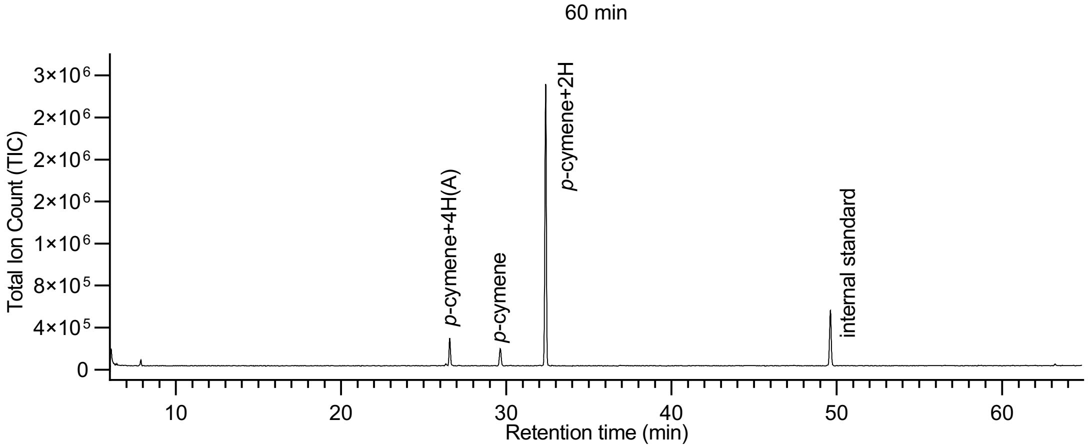

Table S35. Reduction of *p*-cymene (**3**) under air using general method B (Table 1, entry 3).

reaction time	0 min		60 min	
peak	area	retention time (min)	area	retention time (min)
p-cymene	76352812	29.76	1224660	29.64
p-cymene+2H			16556481	32.39
p-cymene+4H(A)			1524745	26.59
p-cymene+4H(B)				
internal standard	16013802	49.64	3746671	49.61

GC-MS method A

Chromatogram S12. Reduction of *p*-cymene (**3**) under O_2 using general method B (Table 1, entry 3).

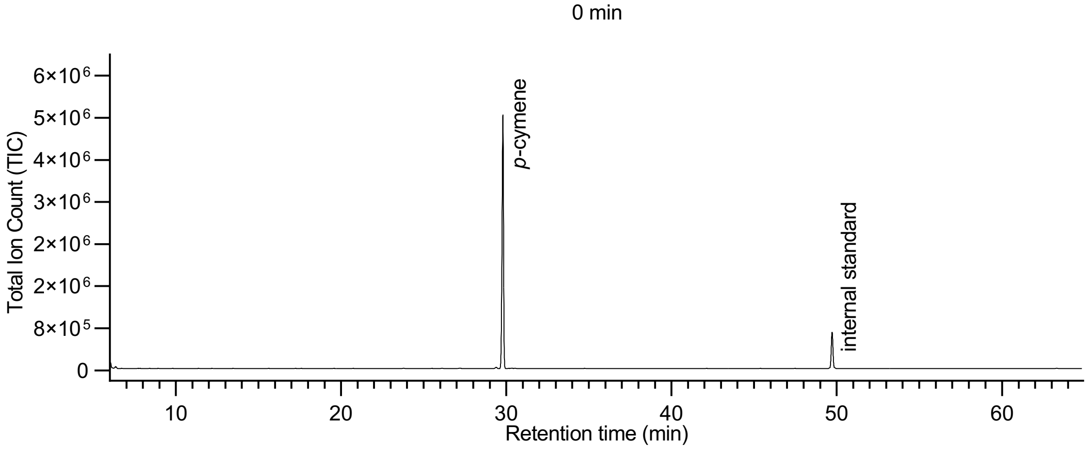

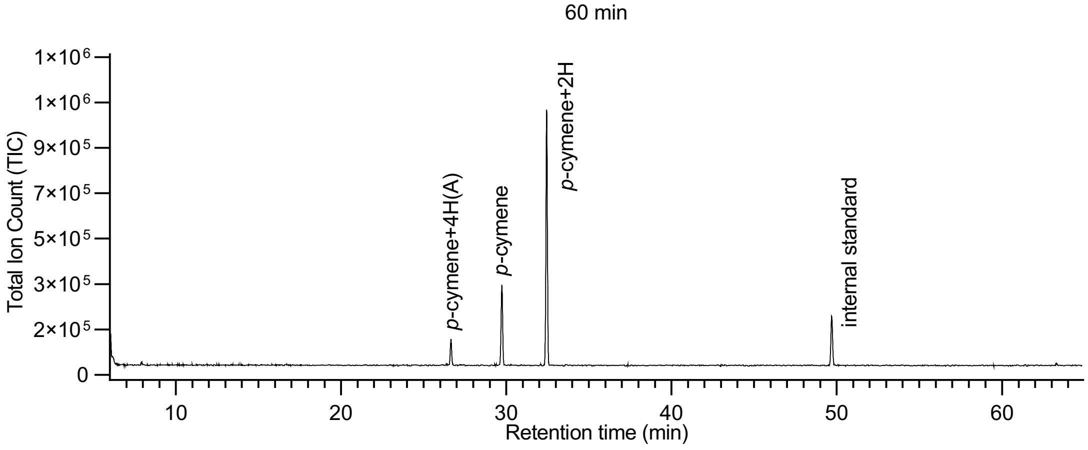

Table S36. Reduction of *p*-cymene (**3**) under O_2 using general method B (Table 1, entry 3).

reaction time	0 min		60 min	
peak	area	retention time (min)	area	retention time (min)
p-cymene	30568985	29.79	1982835	29.74
p-cymene+2H			5828539	32.45
p-cymene+4H(A)			581049	26.65
p-cymene+4H(B)				
internal standard	4977014	49.72	1317257	49.70

GC-MS method A

Chromatogram S13. Reduction of anisole (**4**) under Ar using general method A (Table 1, entry 4).

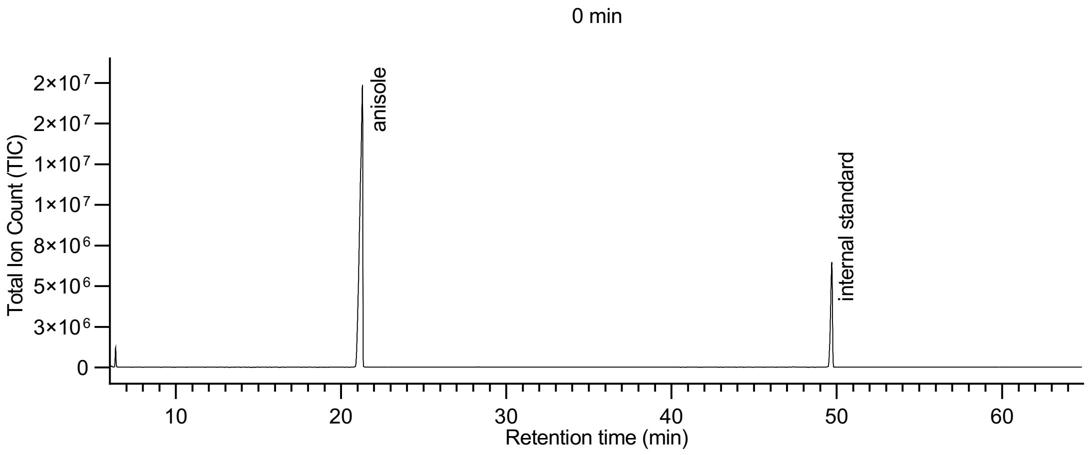

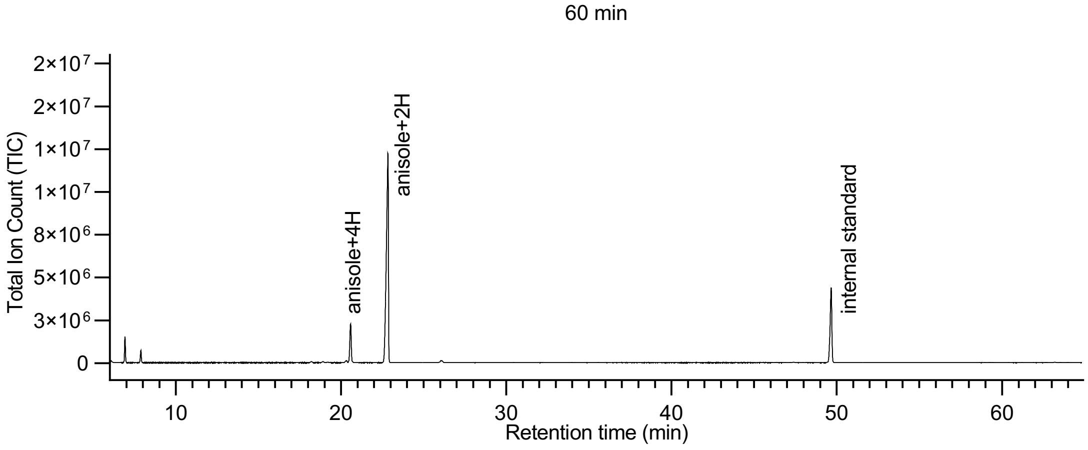

Table S37. Reduction of anisole (**4**) under Ar using general method A (Table 1, entry 4).

reaction time	0 min		60 min	
peak	area	retention time (min)	area	retention time (min)
anisole	214418651	21.29		
anisole+2H			107123194	22.84
anisole+4H			13381542	20.58
internal standard	50739398	49.70	32033101	49.66

Spectrum S4. Mass spectrums of anisole (**4**) and reduction products from Chromatogram S13.

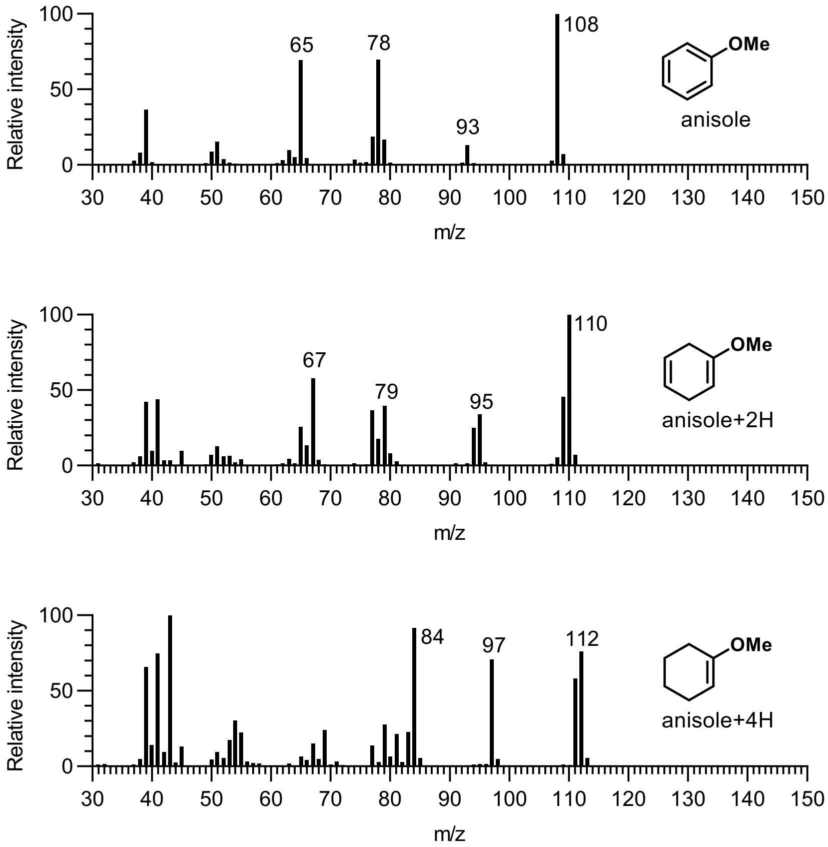

GC-MS method A

Chromatogram S14. Reduction of anisole (**4**) under N_2 using general method A (Table 1, entry 4).

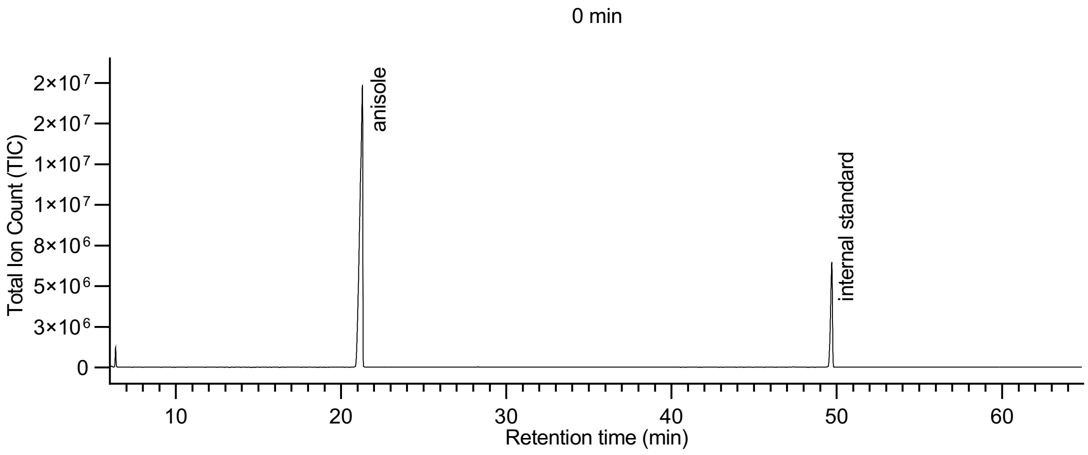

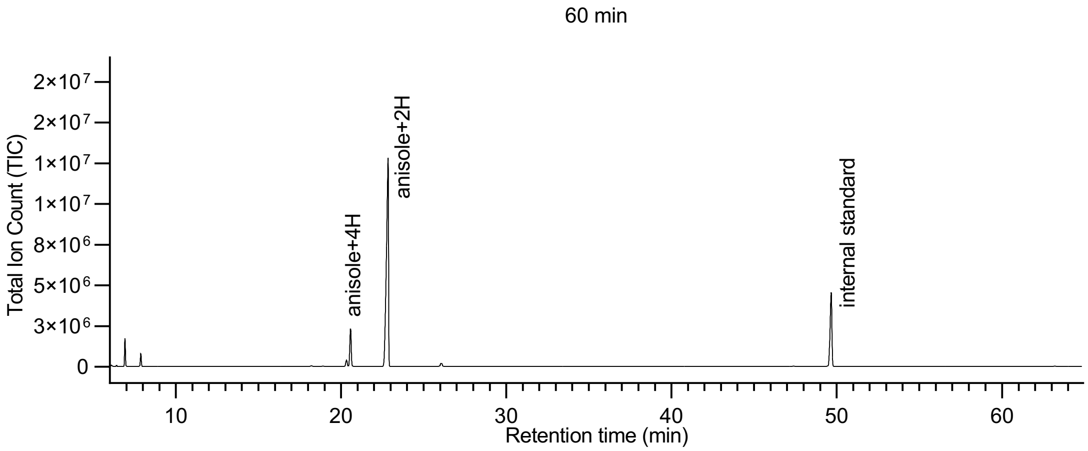

Table S38. Reduction of anisole (**4**) under N_2 using general method A (Table 1, entry 4).

reaction time	0 min		60 min	
peak	area	retention time (min)	area	retention time (min)
anisole	214418651	21.29		
anisole+2H			114632742	22.84
anisole+4H			13622213	20.58
internal standard	50739398	49.70	34673146	49.66

GC-MS method A

Chromatogram S15. Reduction of anisole (**4**) under air using general method B (Table 1, entry 4).

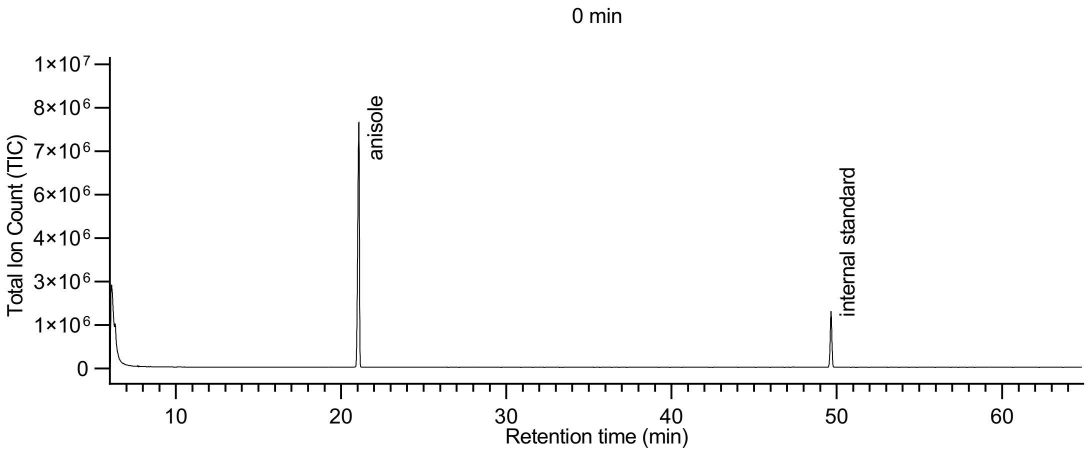

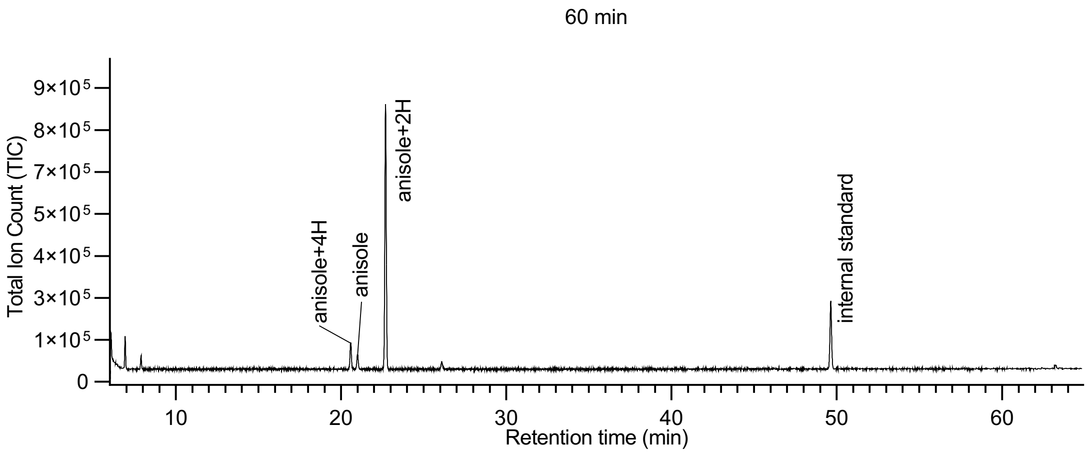

Table S39. Reduction of anisole (**4**) under air using general method B (Table 1, entry 4).

reaction time	0 min		60 min	
peak	area	retention time (min)	area	retention time (min)
anisole	55084153	21.07	273687	21.00
anisole+2H			5095302	22.69
anisole+4H			496637	20.59
internal standard	12619422	49.65	1397769	49.64

GC-MS method A

Chromatogram S16. Reduction of anisole (**4**) under O_2 using general method B (Table 1, entry 4).

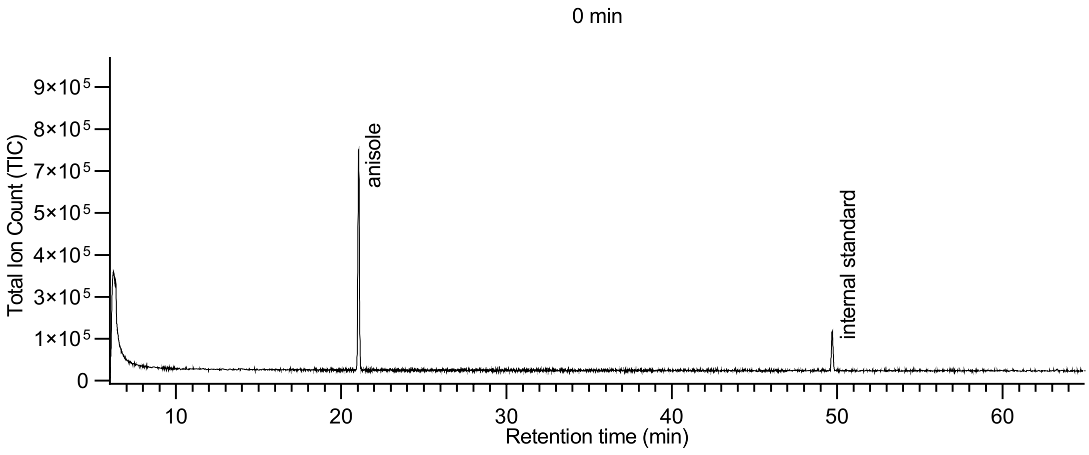

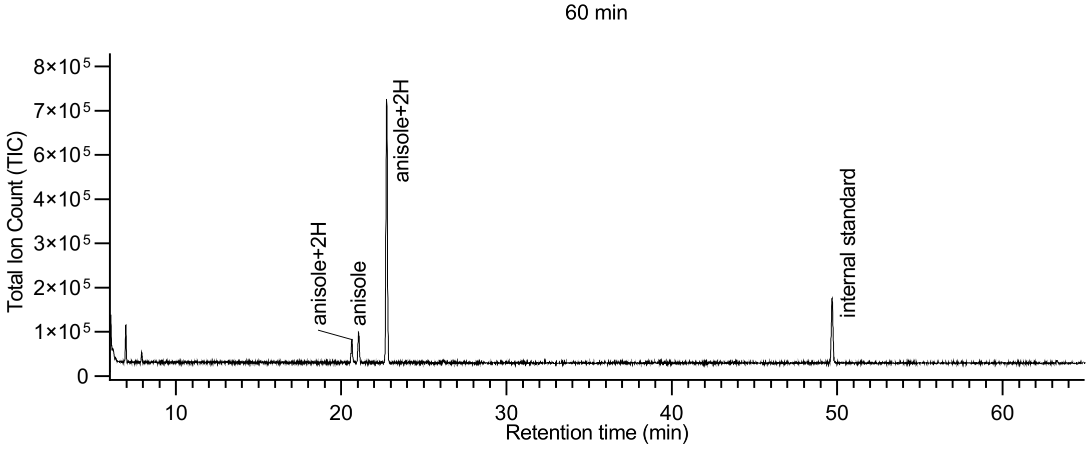

Table S40. Reduction of anisole (**4**) under O_2 using general method B (Table 1, entry 4).

reaction time	0 min		60 min	
peak	area	retention time (min)	area	retention time (min)
anisole	4170121	21.06	441718	21.06
anisole+2H			4083079	22.75
anisole+4H			316760	20.62
internal standard	862123	49.70	1161394	49.70

GC-MS method A

Chromatogram S17. Reduction of *n*-butoxybenzene (**5**) under Ar using general method A (Table 1, entry 5).

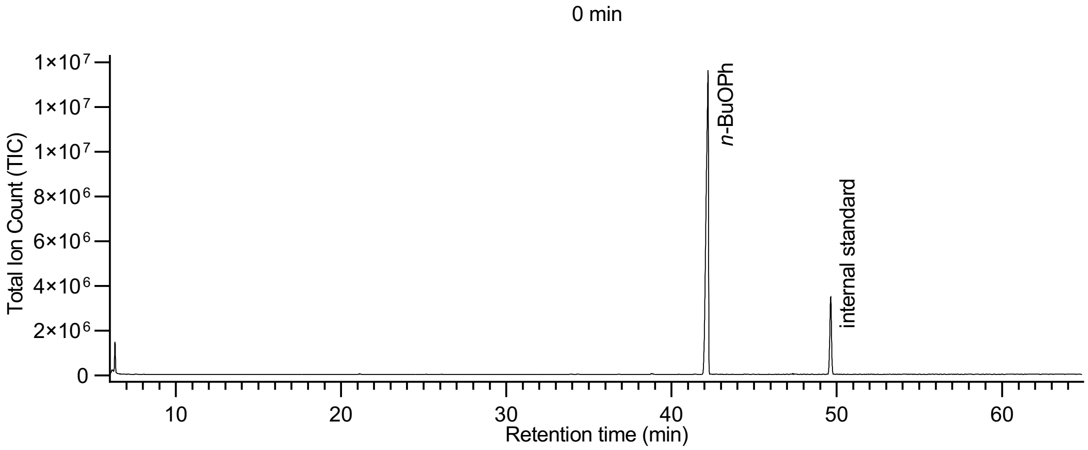

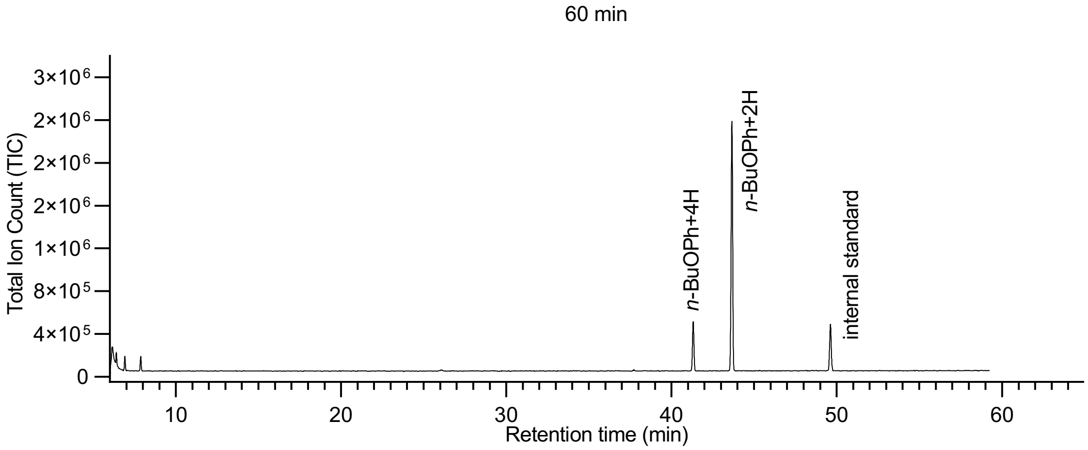

Table S41. Reduction of *n*-butoxybenzene (**5**) under Ar using general method A (Table 1, entry 5).

reaction time	0 min		60 min	
peak	area	retention time (min)	area	retention time (min)
n-BuOPh	145019754	42.21		
n-BuOPh+2H			14951412	43.66
n-BuOPh+4H			2856128	41.31
internal standard	26178107	49.64	3034771	49.62

Spectrum S5. Mass spectrums of *n*-butoxybenzene (**5**) and reduction products from Chromatogram S17.

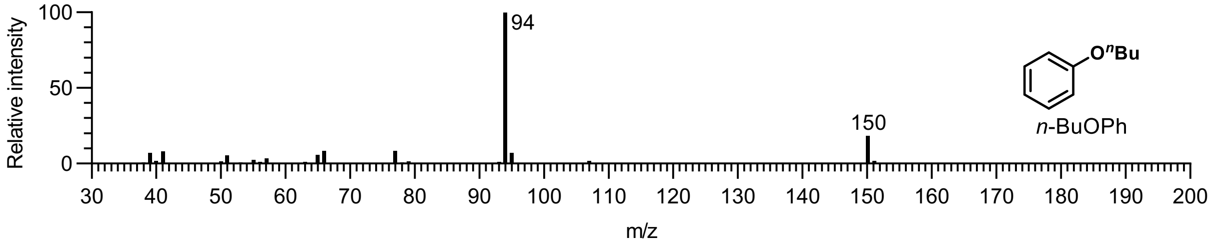

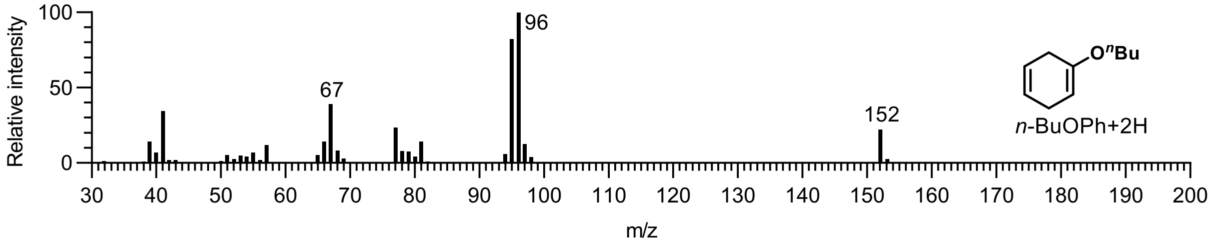

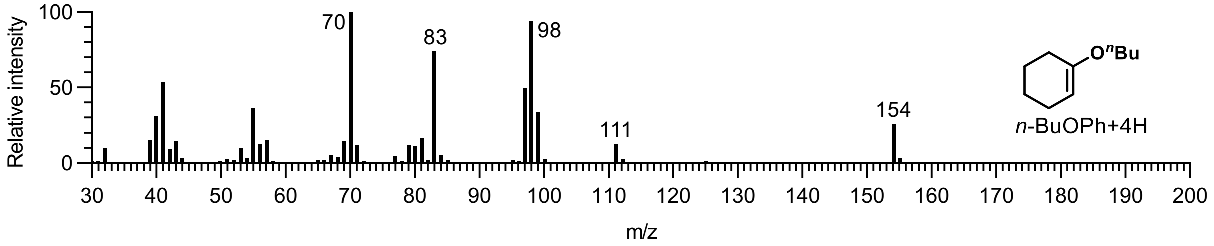

GC-MS method A

Chromatogram S18. Reduction of *n*-butoxybenzene (**5**) under N_2 using general method A (Table 1, entry 5).

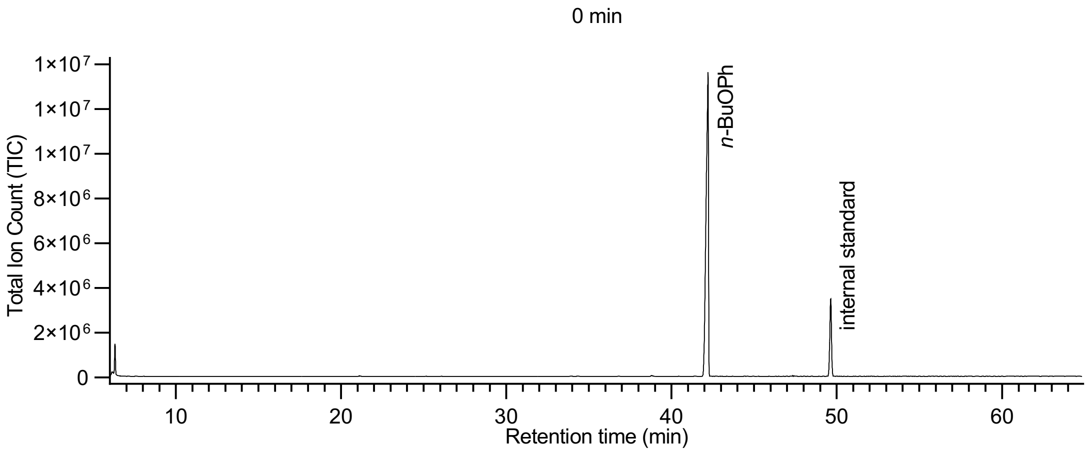

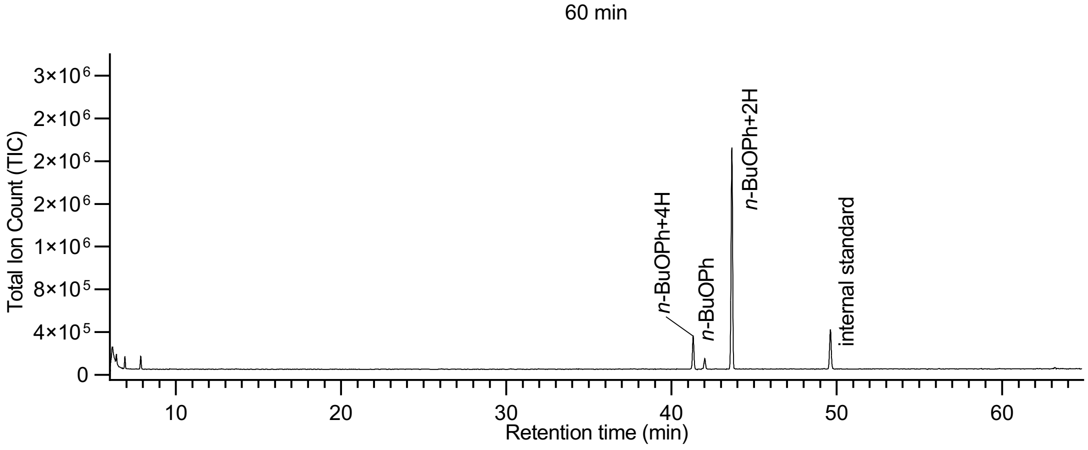

Table S42. Reduction of *n*-butoxybenzene (**5**) under N_2 using general method A (Table 1, entry 5).

reaction time	0 min		60 min	
peak	area	retention time (min)	area	retention time (min)
n-BuOPh	145019754	42.21	622593	42.01
n-BuOPh+2H			13127243	43.65
n-BuOPh+4H			1894455	41.31
internal standard	26178107	49.64	2619962	49.62

GC-MS method A

Chromatogram S19. Reduction of *n*-butoxybenzene (**5**) under air using general method B (Table 1, entry 5).

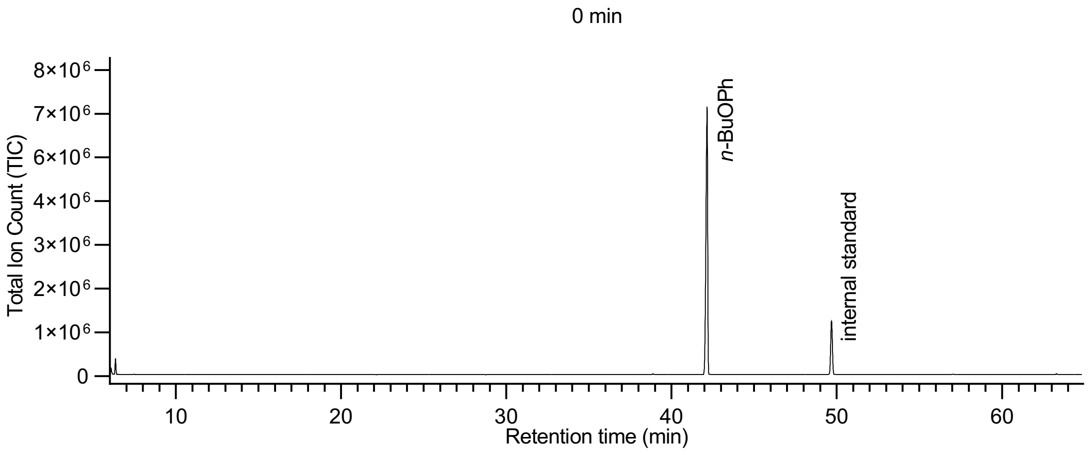

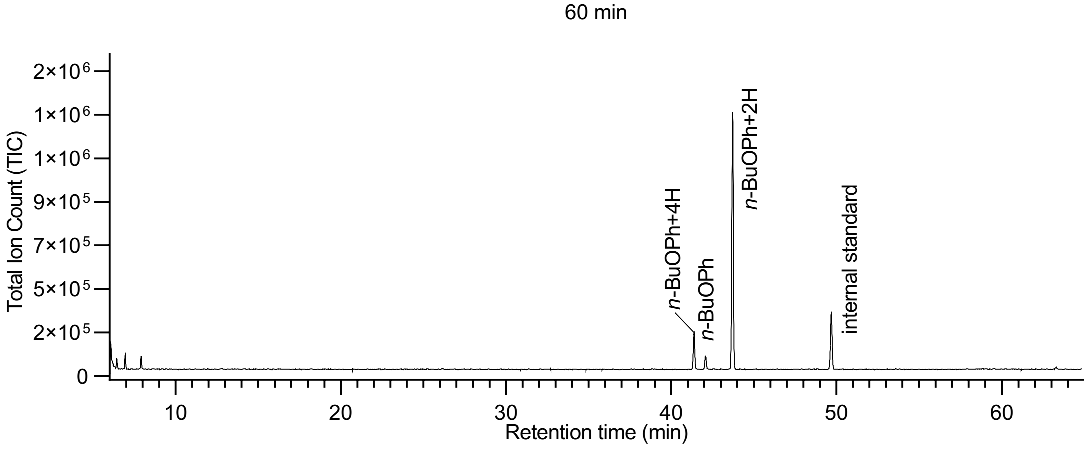

Table S43. Reduction of *n*-butoxybenzene (**5**) under air using general method B (Table 1, entry 5).

reaction time	0 min		60 min	
peak	area	retention time (min)	area	retention time (min)
n-BuOPh	48528412	42.15	481575	42.08
n-BuOPh+2H			8565294	43.71
n-BuOPh+4H			1191034	41.38
internal standard	9721082	49.69	2036390	49.68

GC-MS method A

Chromatogram S20. Reduction of *n*-butoxybenzene (**5**) under O_2 using general method B (Table 1, entry 5).

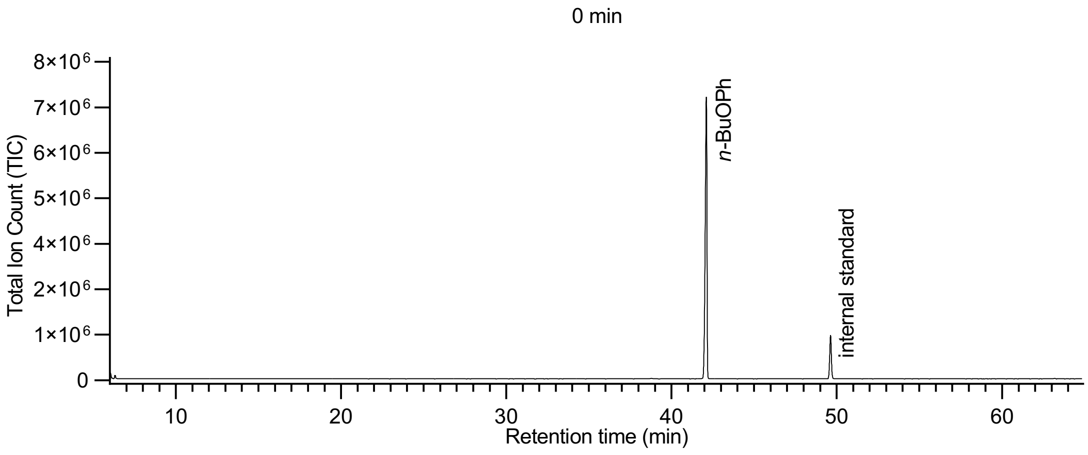

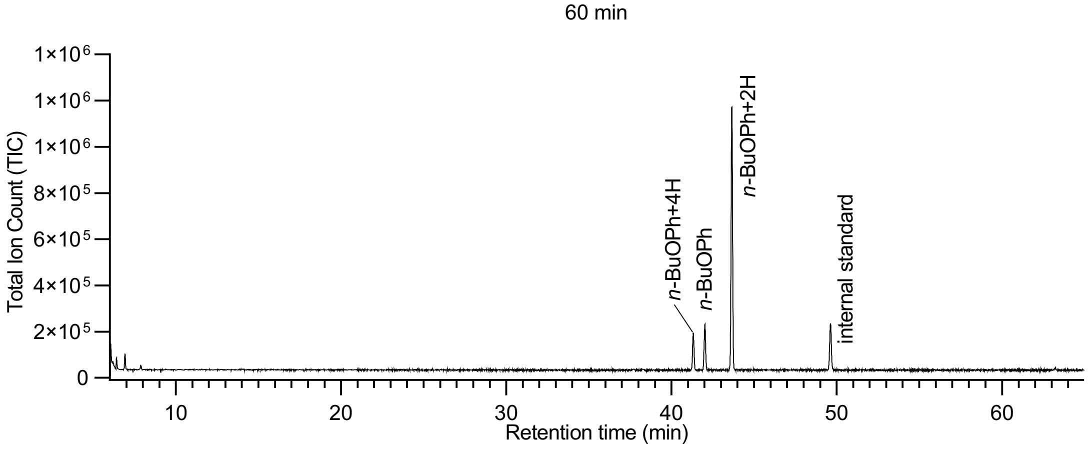

Table S44. Reduction of *n*-butoxybenzene (**5**) under O_2 using general method B (Table 1, entry 5).

reaction time	0 min		60 min	
peak	area	retention time (min)	area	retention time (min)
n-BuOPh	54268554	42.11	1229397	42.02
n-BuOPh+2H			7214001	43.65
n-BuOPh+4H			956699	41.32
internal standard	8038410	49.63	1411022	49.62

GC-MS method A

Chromatogram S21. Reduction of *t*-butyldimethyl(*p*-tolyloxy)silane (**6**) under Ar using general method A (Table 1, entry 6).

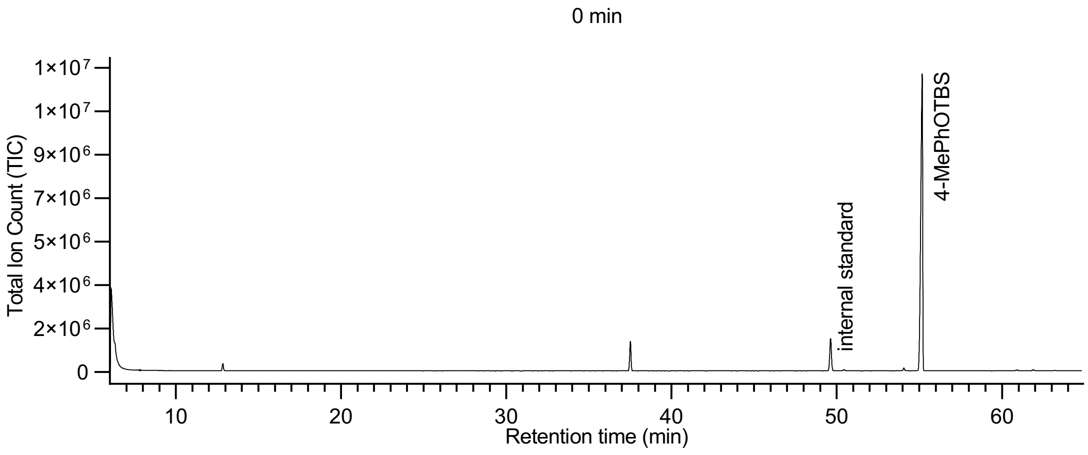

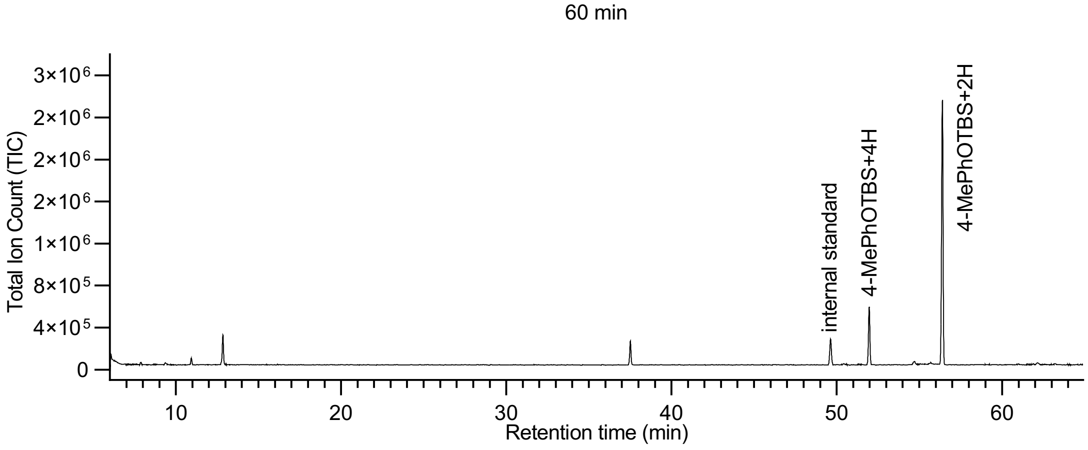

Table S45. Reduction of *t*-butyldimethyl(*p*-tolyloxy)silane (**6**) under Ar using general method A (Table 1, entry 6).

reaction time	0 min		60 min	
peak	area	retention time (min)	area	retention time (min)
4-MePhOTBS	98578989	55.17		
4-MePhOTBS+2H			15523702	56.39
4-MePhOTBS+4H			3378937	51.96
internal standard	9529868	49.63	1770683	49.62

Spectrum S6. Mass spectrums of *t*-butyldimethyl(*p*-tolyloxy)silane (**6**) and reduction products from Chromatogram S21.

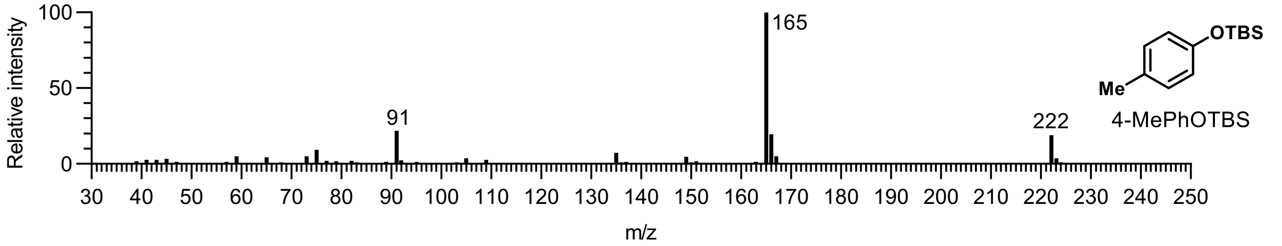

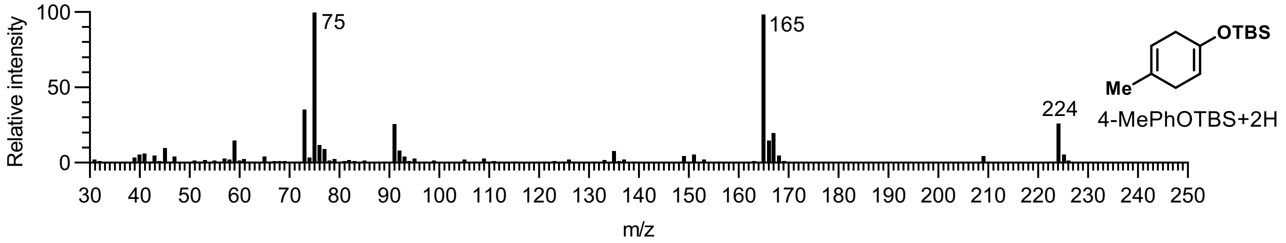

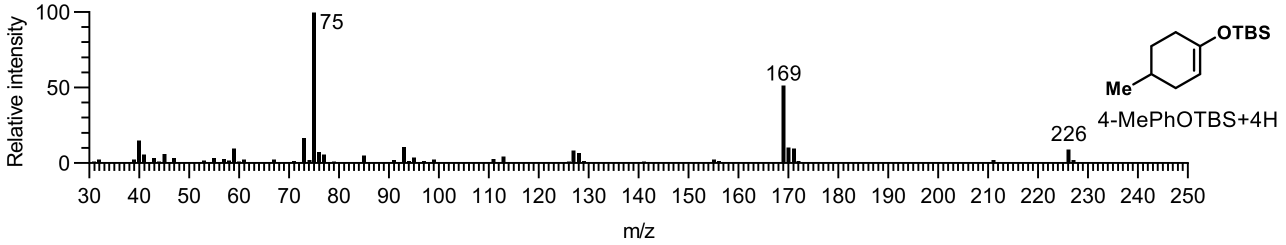

GC-MS method A

Chromatogram S22. Reduction of *t*-butyldimethyl(*p*-tolyloxy)silane (**6**) under N_2 using general method A (Table 1, entry 6).

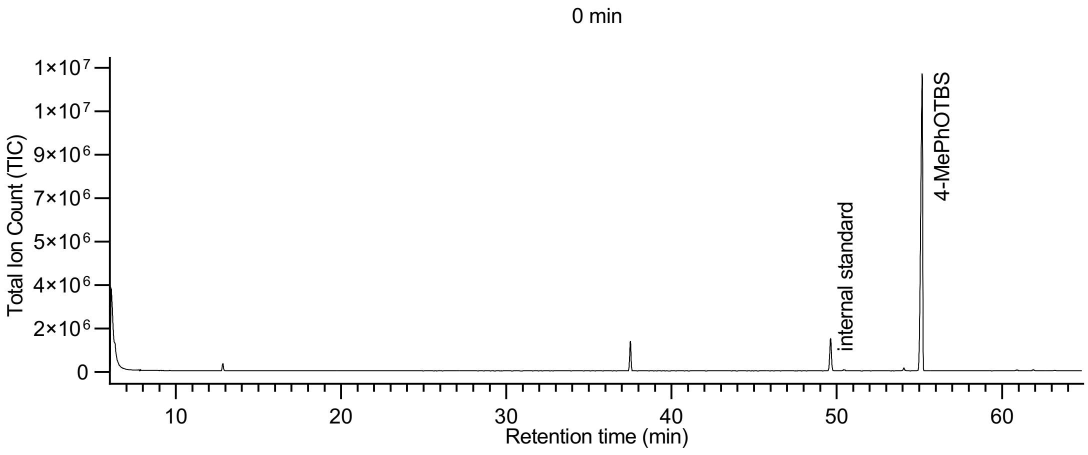

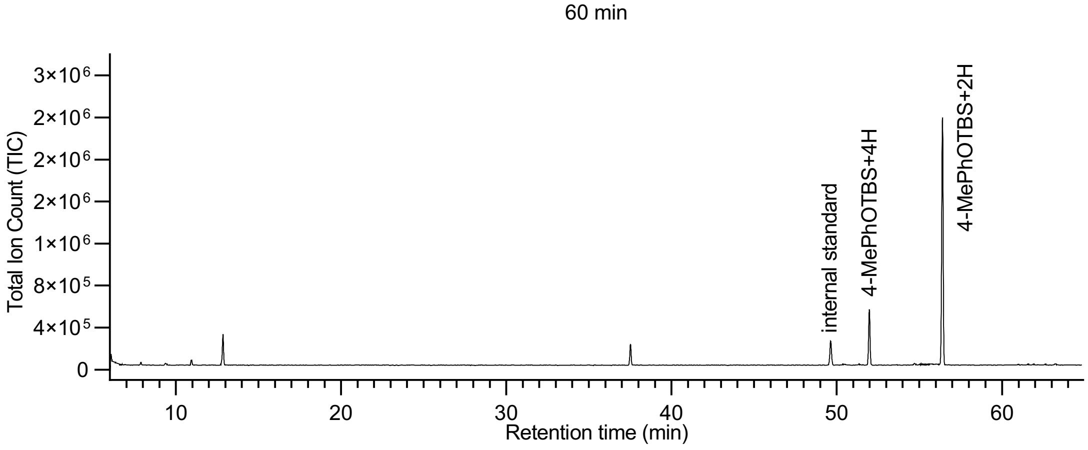

Table S46. Reduction of *t*-butyldimethyl(*p*-tolyloxy)silane (**6**) under N_2 using general method A (Table 1, entry 6).

reaction time	0 min		60 min	
peak	area	retention time (min)	area	retention time (min)
4-MePhOTBS	98578989	55.17		
4-MePhOTBS+2H			14469217	56.40
4-MePhOTBS+4H			3209082	51.97
internal standard	9529868	49.63	1638627	49.63

GC-MS method A

Chromatogram S23. Reduction of *t*-butyldimethyl(*p*-tolyloxy)silane (**6**) under air using general method B (Table 1, entry 6).

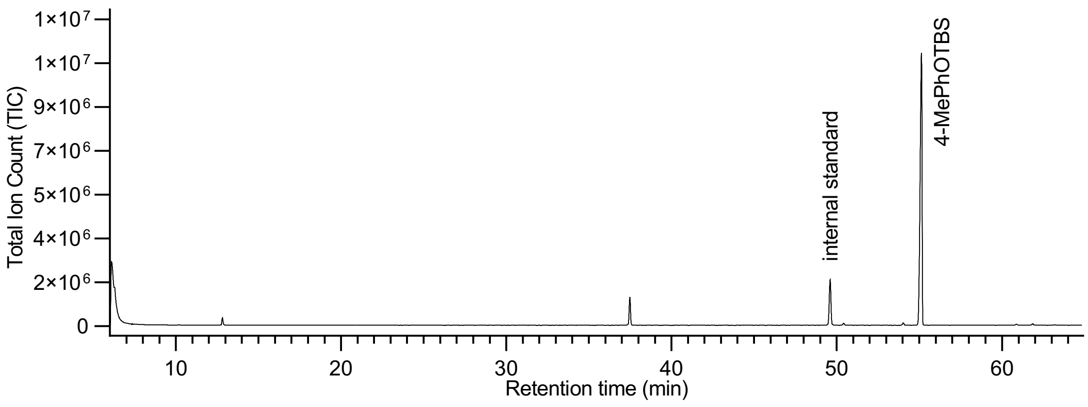

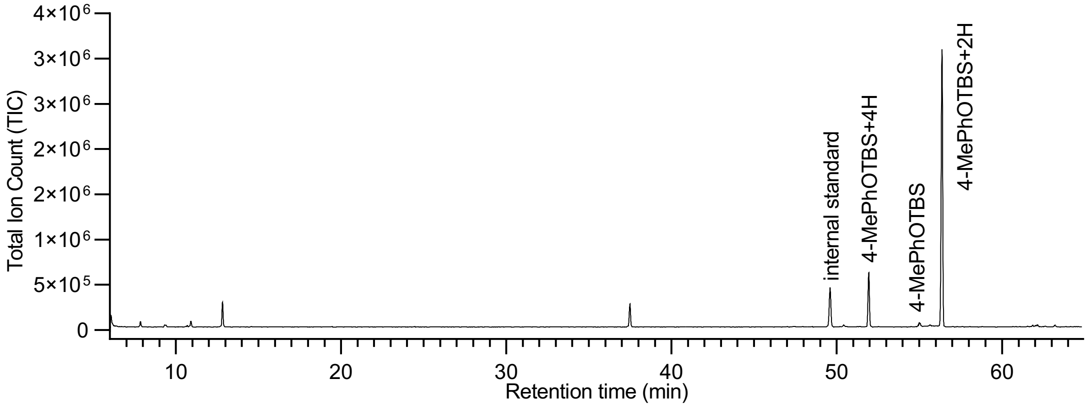

Table S47. Reduction of *t*-butyldimethyl(*p*-tolyloxy)silane (**6**) under air using general method B (Table 1, entry 6).

reaction time	0 min		60 min	
peak	area	retention time (min)	area	retention time (min)
4-MePhOTBS	90451990	55.12	296184	55.01
4-MePhOTBS+2H			19212767	56.37
4-MePhOTBS+4H			3664673	51.94
internal standard	13298901	49.60	3078266	49.60

GC-MS method A

Chromatogram S24. Reduction of *t*-butyldimethyl(*p*-tolyloxy)silane (**6**) under O_2 using general method B (Table 1, entry 6).

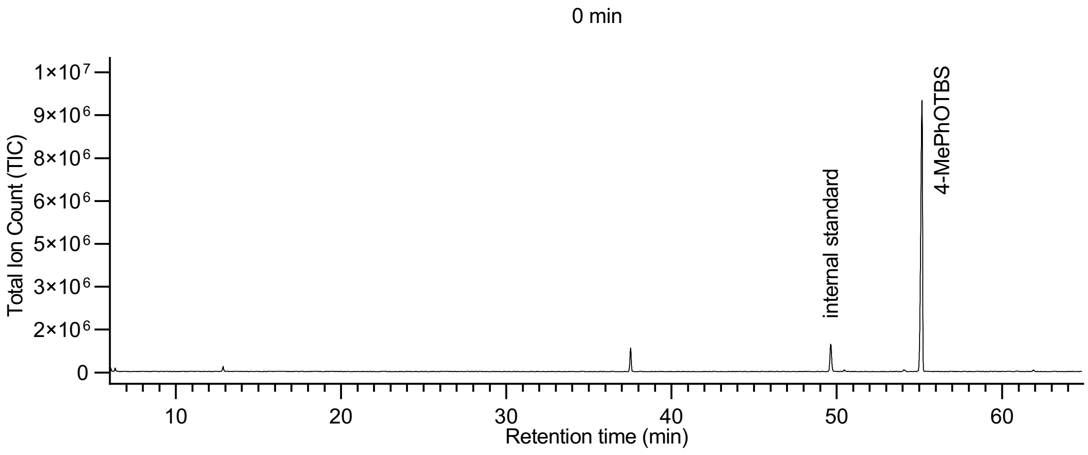

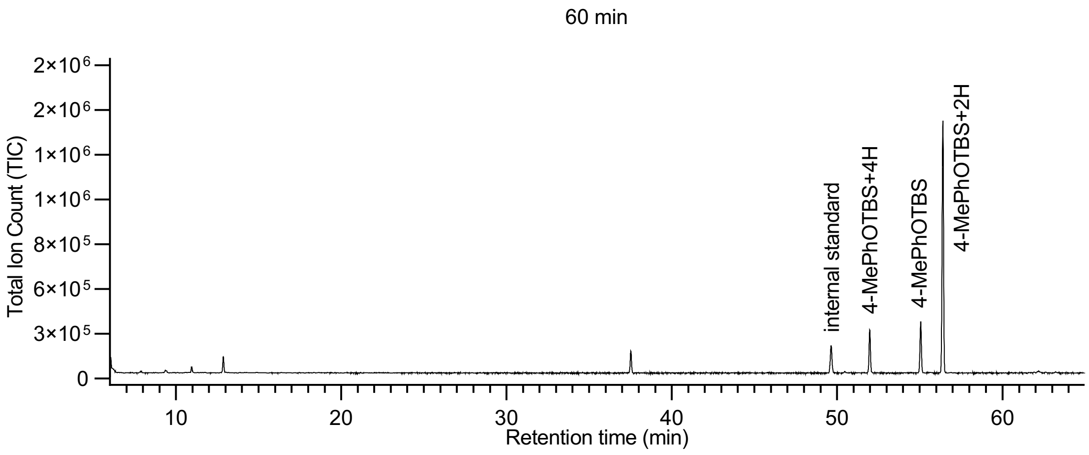

Table S48. Reduction of *t*-butyldimethyl(*p*-tolyloxy)silane (**6**) under O_2 using general method B (Table 1, entry 6).

reaction time	0 min		60 min	
peak	area	retention time (min)	area	retention time (min)
4-MePhOTBS	71486247	55.16	1917890	55.05
4-MePhOTBS+2H			9464877	56.40
4-MePhOTBS+4H			1614030	51.97
internal standard	6880403	49.64	1198704	49.64

GC-MS method A

Chromatogram S25. Reduction of 3-methylanisole (**7**) under Ar using general method A (Table 1, entry 7).

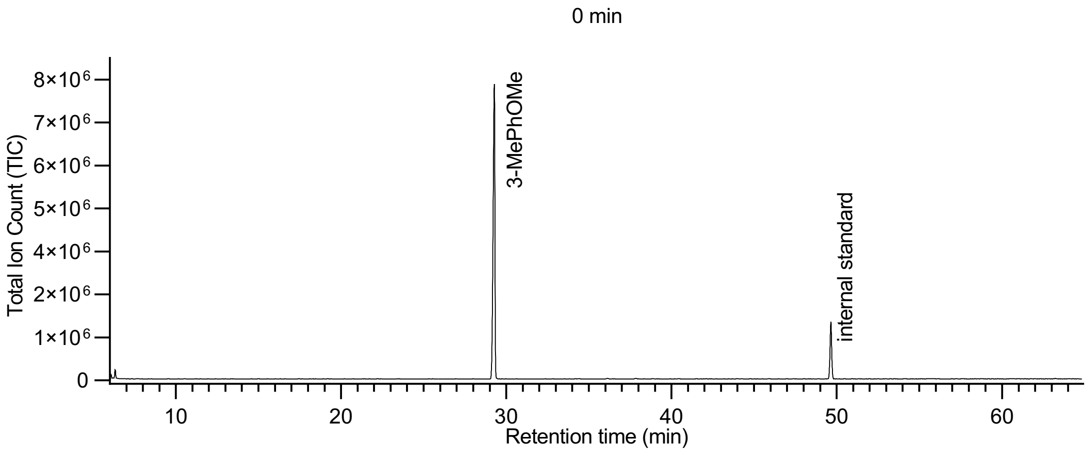

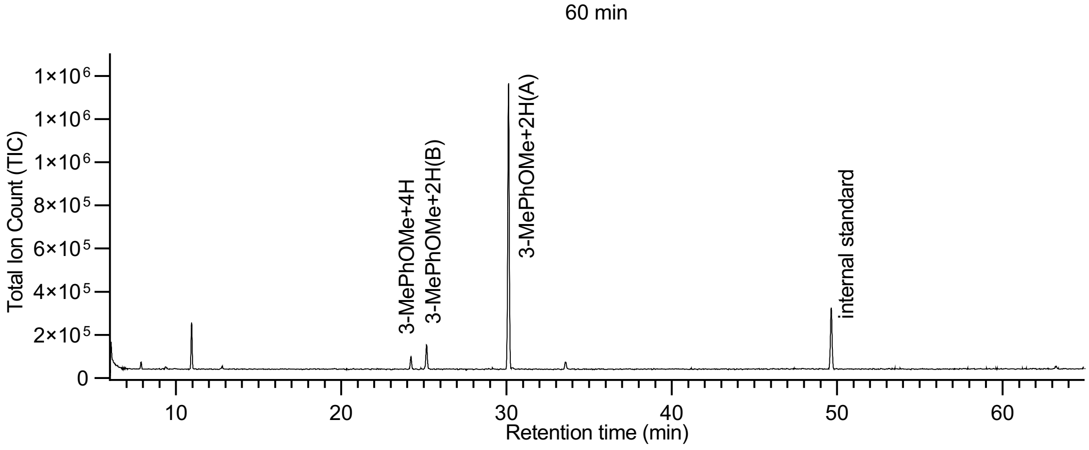

Table S49. Reduction of 3-methylanisole (**7**) under Ar using general method A (Table 1, entry 7).

reaction time	0 min		60 min	
peak	area	retention time (min)	area	retention time (min)
3-MePhOMe	62113248	29.28		
3-MePhOMe+2H(A)			8271109	30.12
3-MePhOMe+2H(B)			718567	25.16
3-MePhOMe+4H			323582	24.22
internal standard	11139537	49.65	2024148	49.64

Spectrum S7. Mass spectrums of 3-methylanisole (**7**) and reduction products from Chromatogram S25.

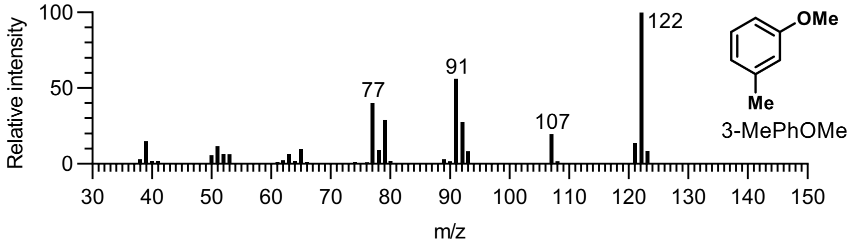

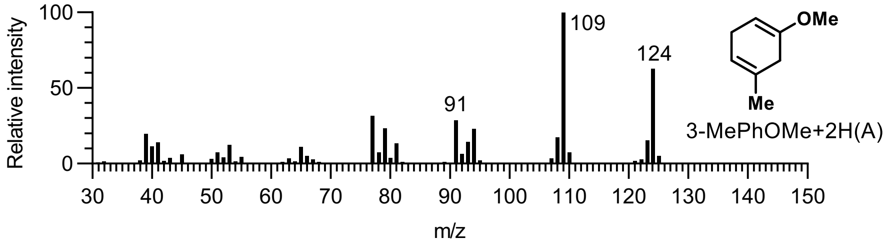

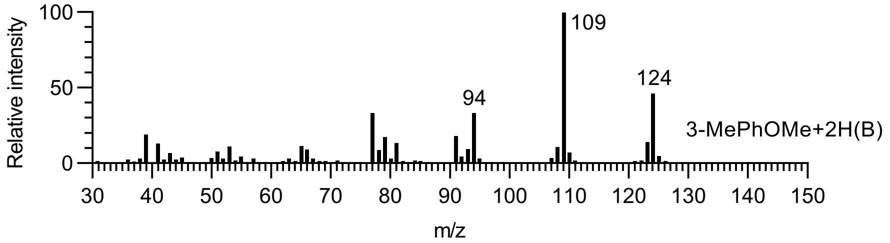

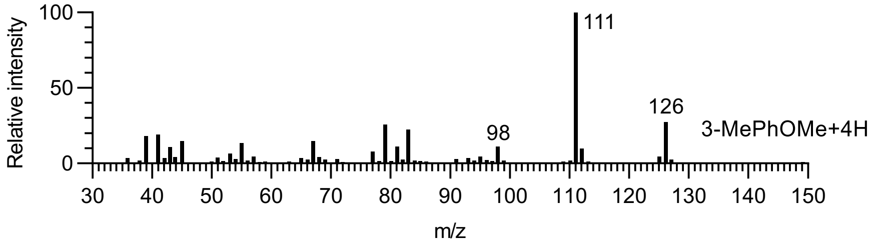

GC-MS method A

Chromatogram S26. Reduction of 3-methylanisole (**7**) under N_2 using general method A (Table 1, entry 7).

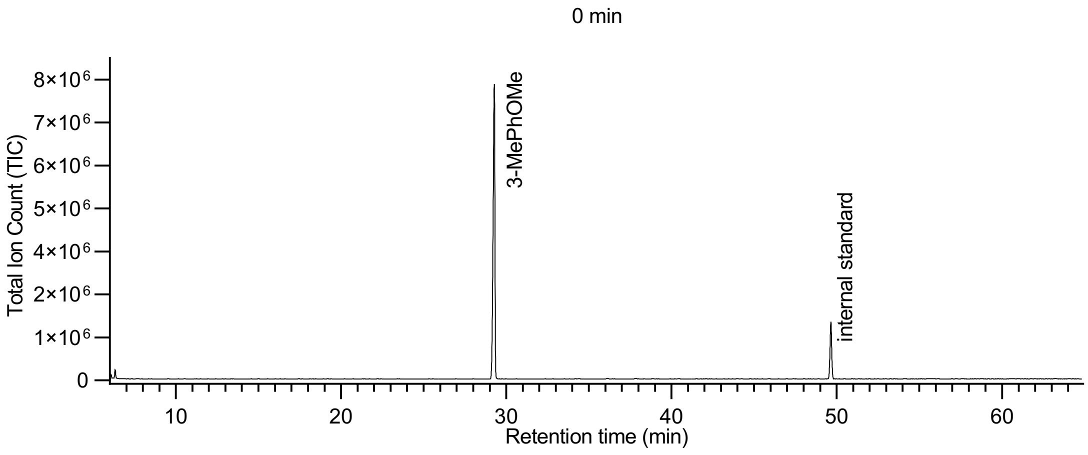

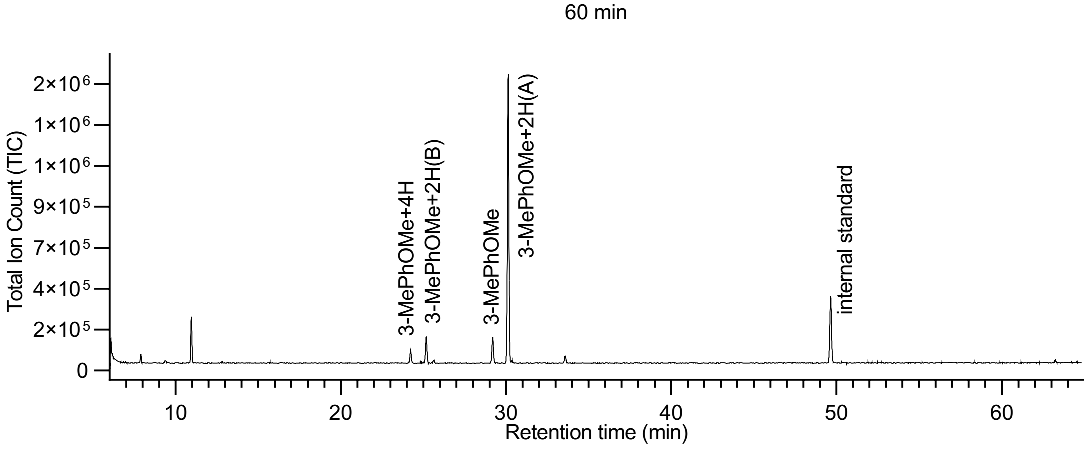

Table S50. Reduction of 3-methylanisole (**7**) under N_2 using general method A (Table 1, entry 7).

reaction time	0 min		60 min	
peak	area	retention time (min)	area	retention time (min)
3-MePhOMe	62113248	29.28	869532	29.19
3-MePhOMe+2H(A)			9728205	30.12
3-MePhOMe+2H(B)			904140	25.16
3-MePhOMe+4H			374318	24.23
internal standard	11139537	49.65	2557170	49.65

GC-MS method A

Chromatogram S27. Reduction of 3-methylanisole (**7**) under air using general method B (Table 1, entry 7).

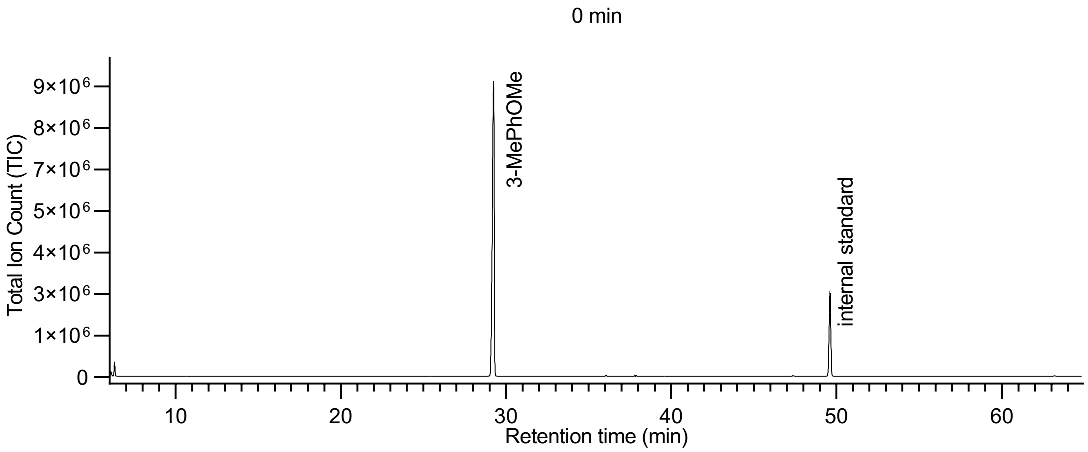

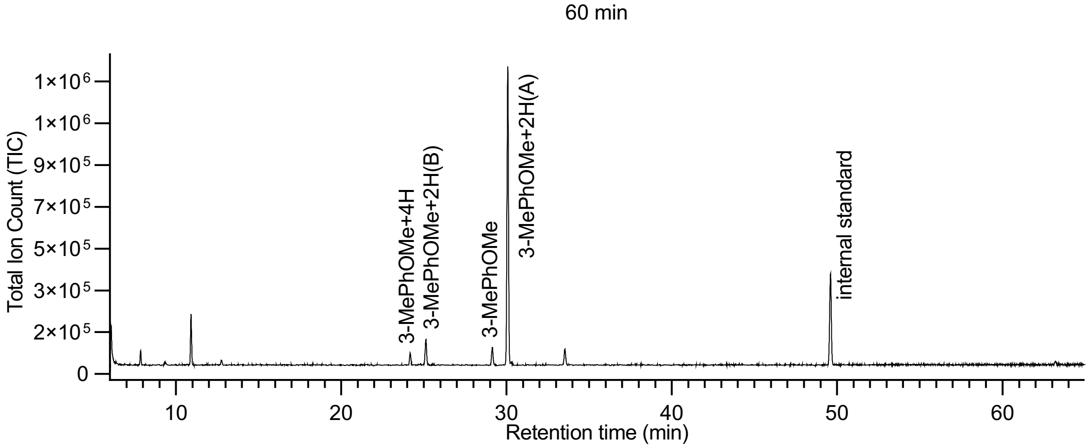

Table S51. Reduction of 3-methylanisole (**7**) under air using general method B (Table 1, entry 7).

reaction time	0 min		60 min	
peak	area	retention time (min)	area	retention time (min)
3-MePhOMe	74065483	29.25	461580	29.14
3-MePhOMe+2H(A)			7708828	30.08
3-MePhOMe+2H(B)			659649	25.12
3-MePhOMe+4H			272613	24.17
internal standard	18559940	49.61	2628548	49.60

GC-MS method A

Chromatogram S28. Reduction of 3-methylanisole (**7**) under O_2 using general method B (Table 1, entry 7).

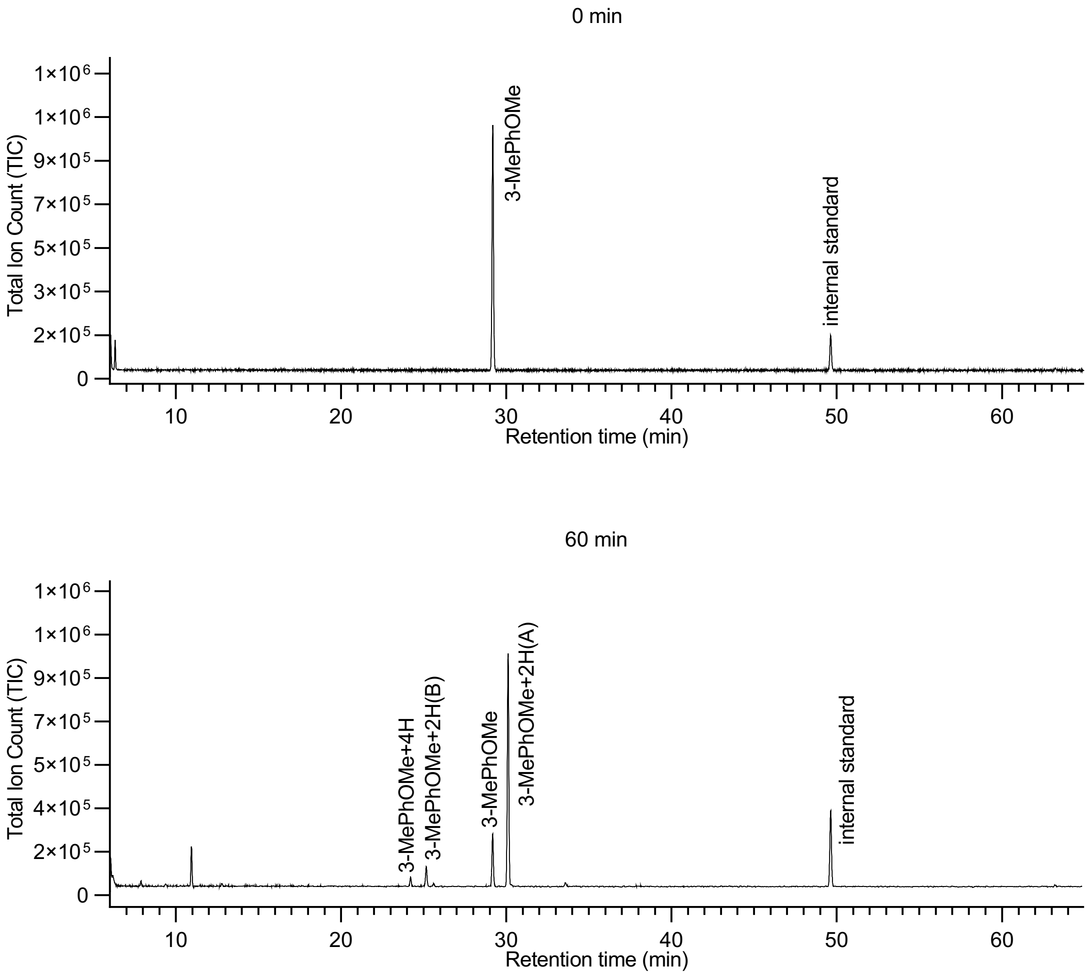

Table S52. Reduction of 3-methylanisole (**7**) under O_2 using general method B (Table 1, entry 7).

reaction time	0 min		60 min	
peak	area	retention time (min)	area	retention time (min)
3-MePhOMe	6140381	29.19	1387487	29.18
3-MePhOMe+2H(A)			6165162	30.11
3-MePhOMe+2H(B)			510145	25.16
3-MePhOMe+4H			207070	24.21
internal standard	955346	49.64	2246521	49.63

GC-MS method A

Chromatogram S29. Reduction of naphthalene (**8**) under Ar using general method A (Table 1, entry 8).

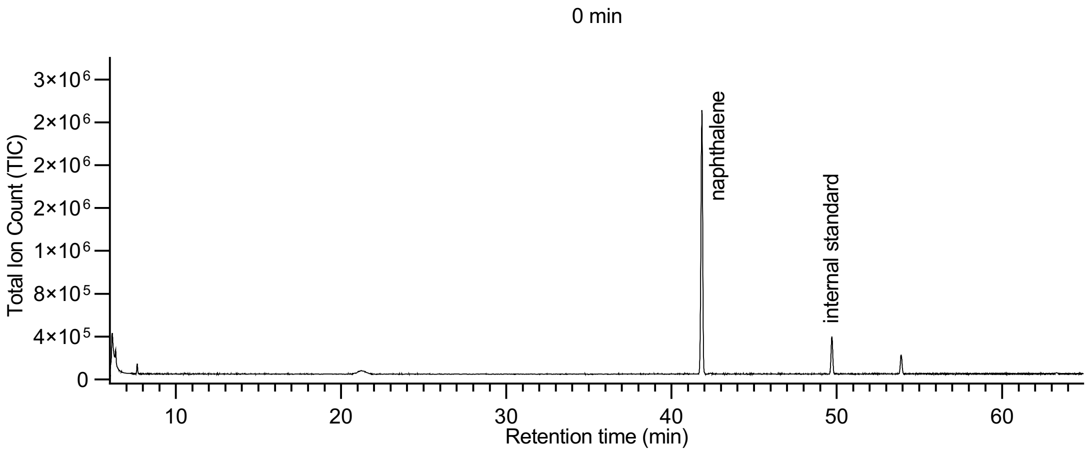

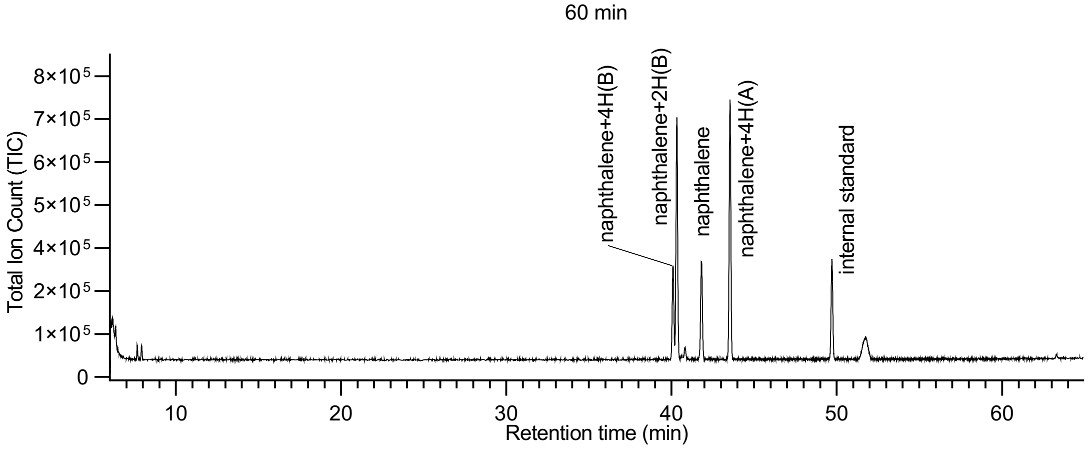

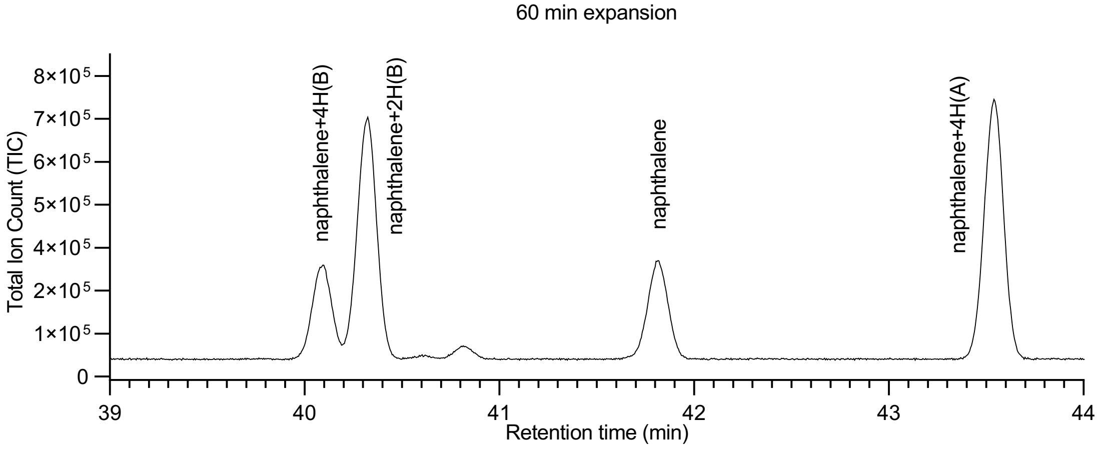

Table S53. Reduction of naphthalene (**8**) under Ar using general method A (Table 1, entry 8).

reaction time	0 min		60 min	
peak	area	retention time (min)	area	retention time (min)
naphthalene	17978418	41.84	2007020	41.81
naphthalene+2H(A)				
naphthalene+2H(B)			4681174	40.32
naphthalene+4H(A)			5086271	43.54
naphthalene+4H(B)			1850501	40.08
internal standard	2468777	49.70	1982174	49.71

Spectrum S8. Mass spectrums of naphthalene (**8**) and reduction products from Chromatogram S29.

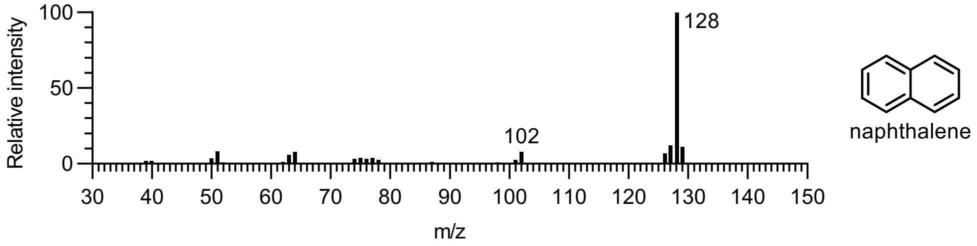

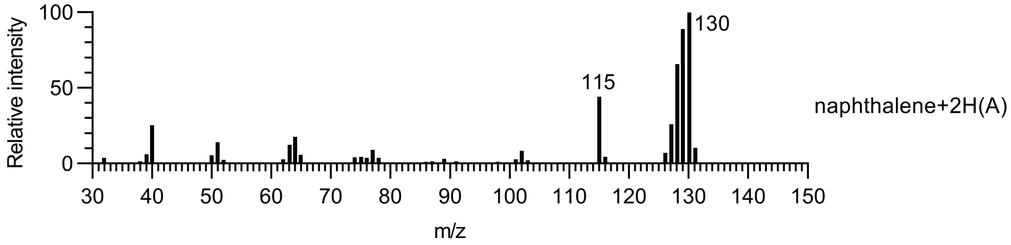

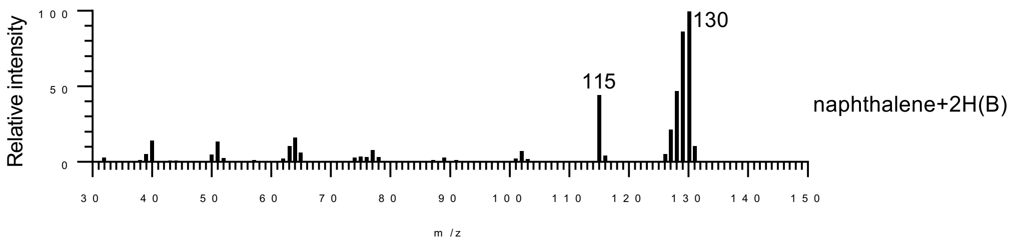

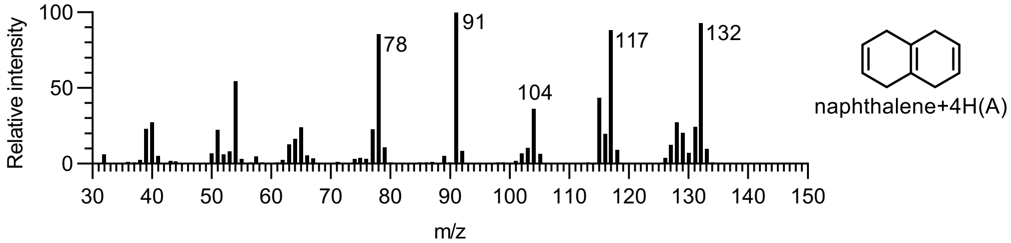

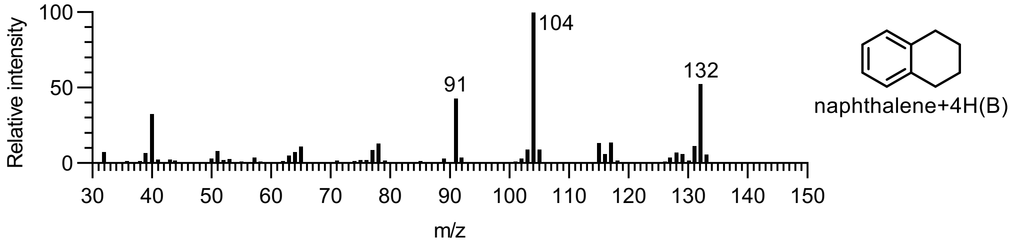

GC-MS method A

Chromatogram S30. Reduction of naphthalene (**8**) under N_2 using general method A (Table 1, entry 8).

0 min

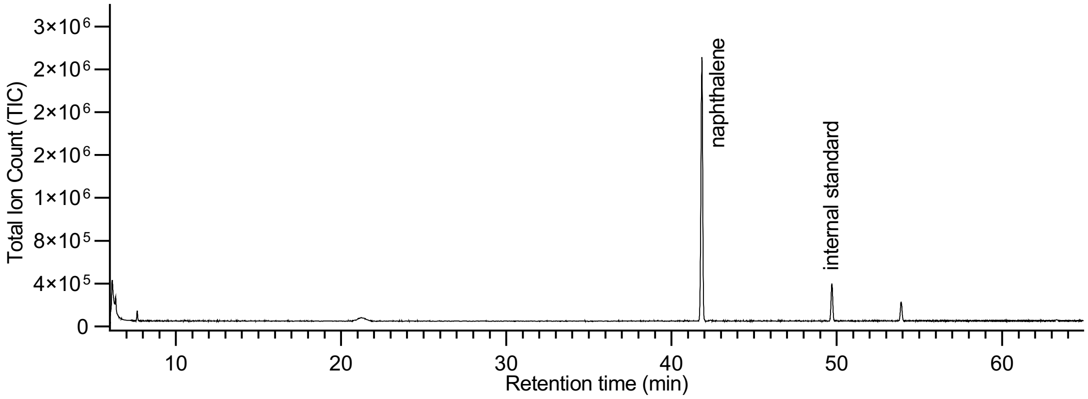

60 min

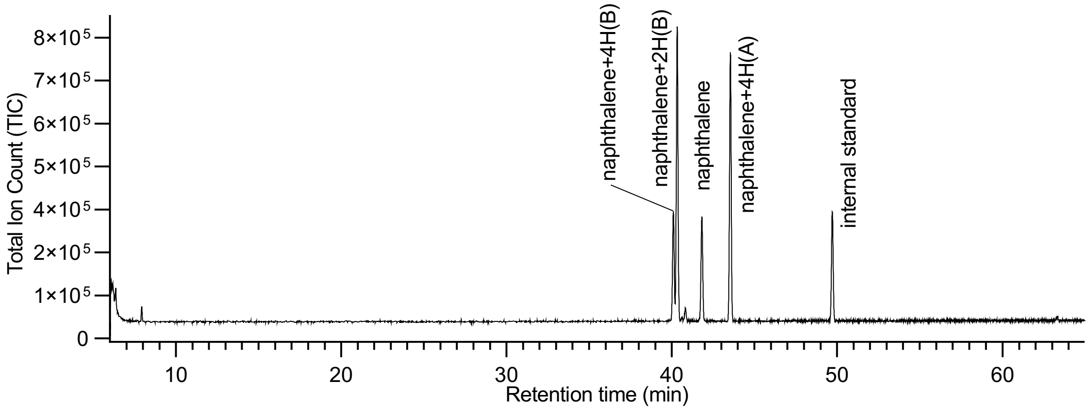

60 min expansion

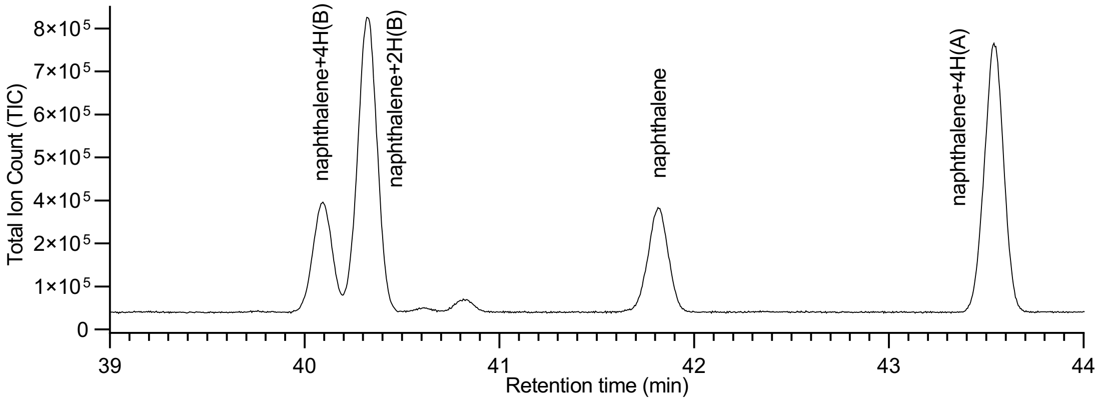

Table S54. Reduction of naphthalene (**8**) under N_2 using general method A (Table 1, entry 8).

reaction time	0 min		60 min	
peak	area	retention time (min)	area	retention time (min)
naphthalene	17978418	41.84	2151641	41.82
naphthalene+2H(A)				
naphthalene+2H(B)			5765844	40.33
naphthalene+4H(A)			5222765	43.55
naphthalene+4H(B)			2130053	40.10
internal standard	2468777	49.70	2155888	49.71

GC-MS method A

Chromatogram S31. Reduction of naphthalene (**8**) under air using general method B (Table 1, entry 8).

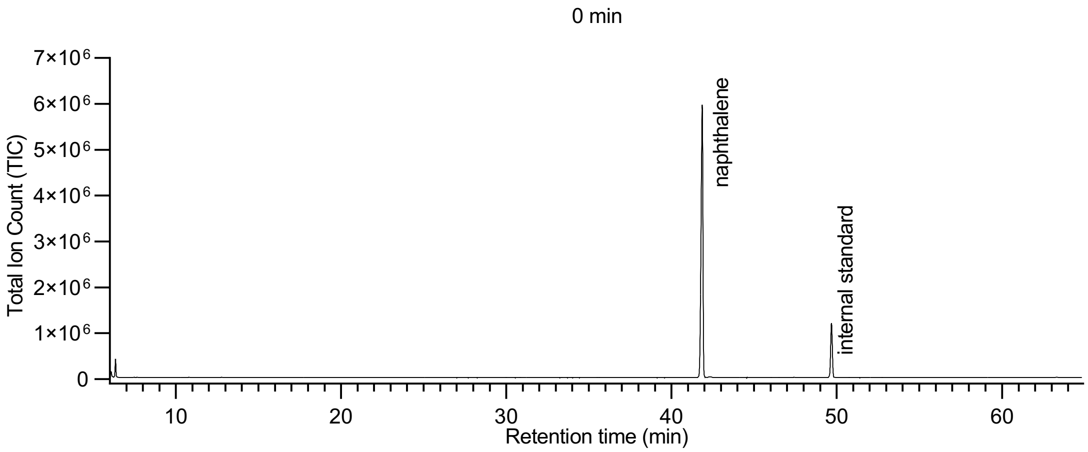

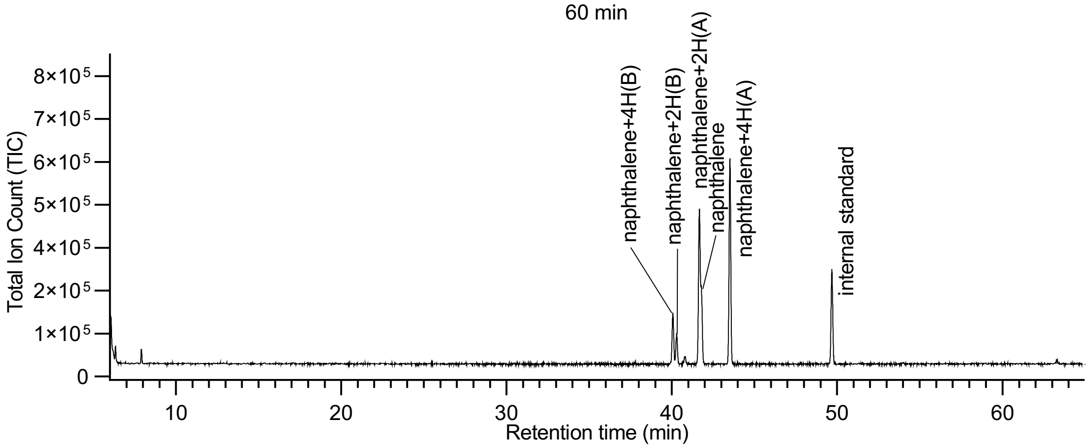

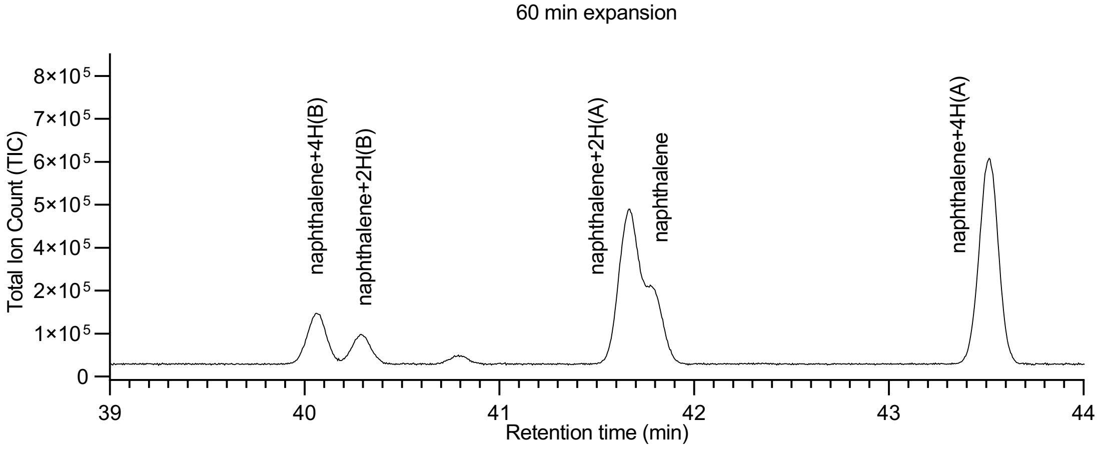

Table S55. Reduction of naphthalene (**8**) under air using general method B (Table 1, entry 8).

reaction time	0 min		60 min	
peak	area	retention time (min)	area	retention time (min)
naphthalene	46857486	41.85	*n.d.*	*n.d.*
naphthalene+2H(A)			*n.d.*	*n.d.*
naphthalene+2H(B)			549590	40.29
naphthalene+4H(A)			3992696	43.51
naphthalene+4H(B)			1003189	40.07
internal standard	8449975	49.68	1872933	49.68

n.d. = not determined

GC-MS method A

Chromatogram S32. Reduction of naphthalene (**8**) under O_2 using general method B (Table 1, entry 8).

0 min

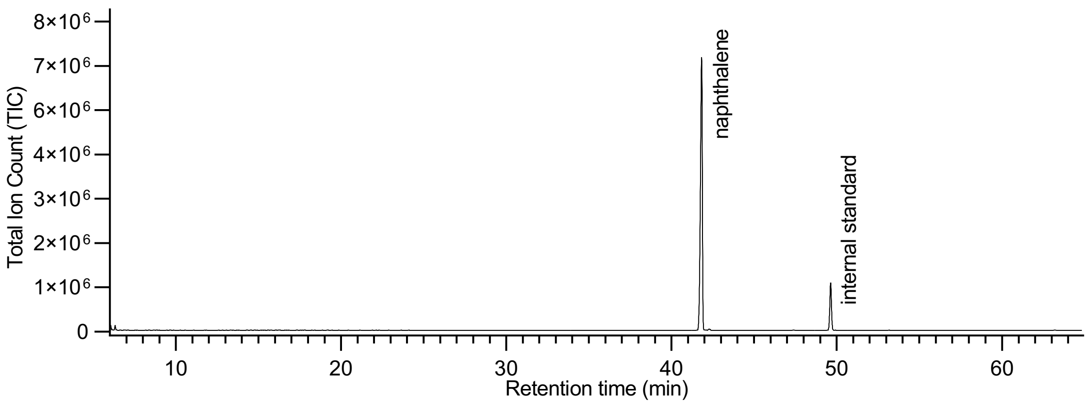

60 min

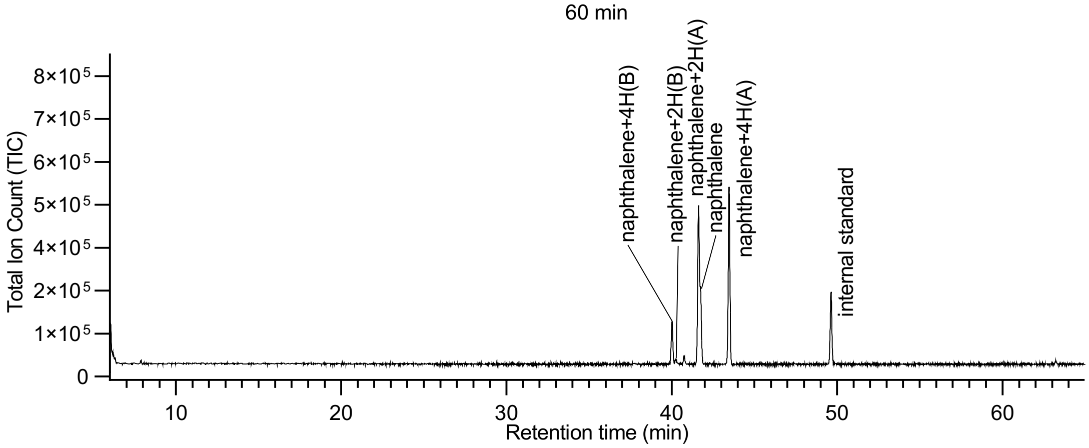

60 min expansion

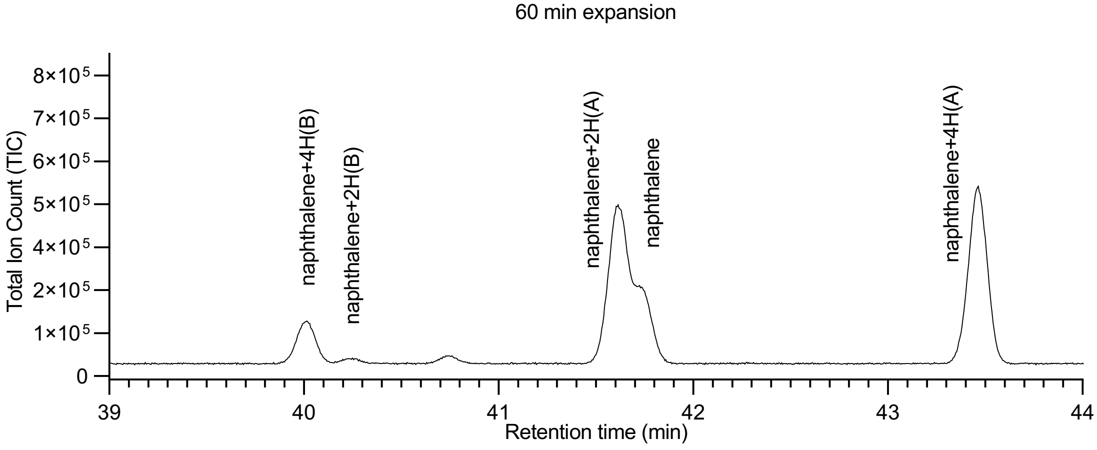

Table S56. Reduction of naphthalene (**8**) under O_2 using general method B (Table 1, entry 8).

reaction time	0 min		60 min	
peak	area	retention time (min)	area	retention time (min)
naphthalene	54558337	41.82	*n.d.*	*n.d.*
naphthalene+2H(A)			*n.d.*	*n.d.*
naphthalene+2H(B)			91343	40.24
naphthalene+4H(A)			3384593	43.47
naphthalene+4H(B)			847953	40.01
internal standard	8335388	49.62	1409449	49.63

n.d. = not determined

GC-MS method B

Chromatogram S33. Chromatogram of Table S3, entry 3.

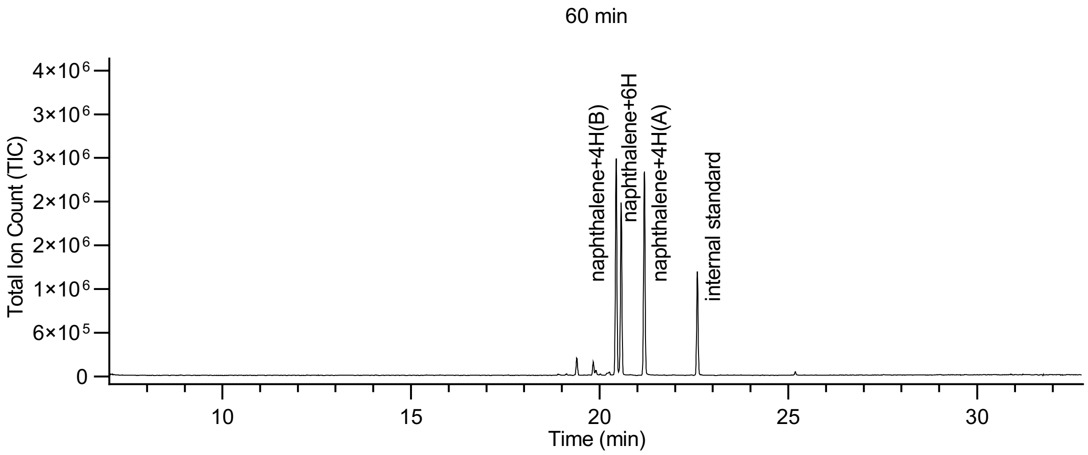

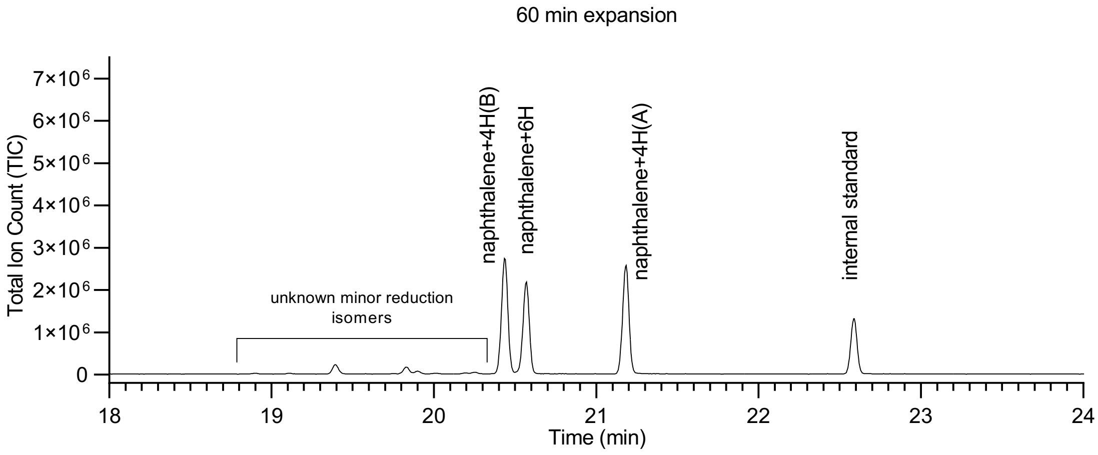

Spectrum S9. Mass spectrum of naphthalene (**8**) over-reduction product from Chromatogram S33 (Table S3, entry 3).

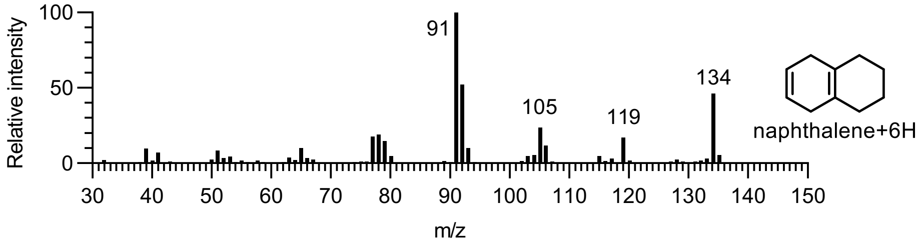

See Spectra S8 for previously shown mass spectra of naphthalene reduction isomers.

GC-MS method B

Chromatogram S34. Chromatogram of Table S3, entry 4.

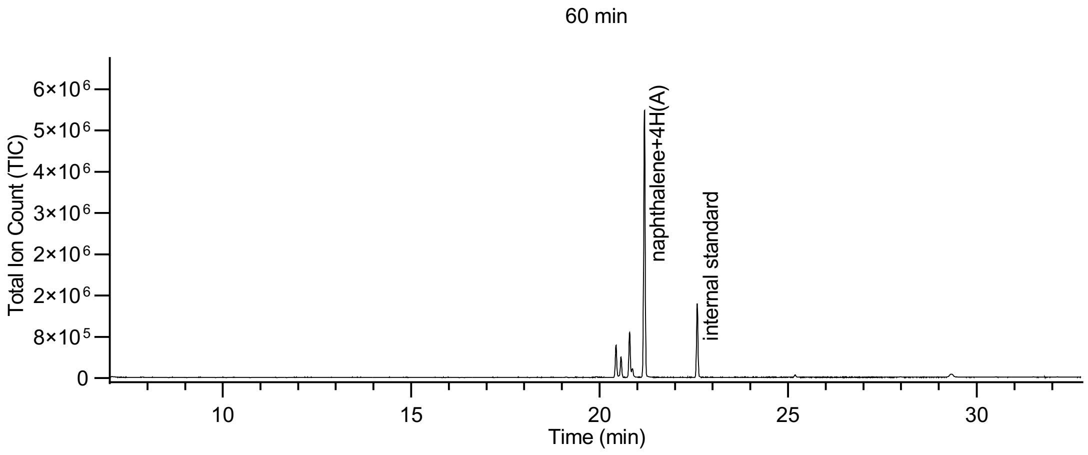

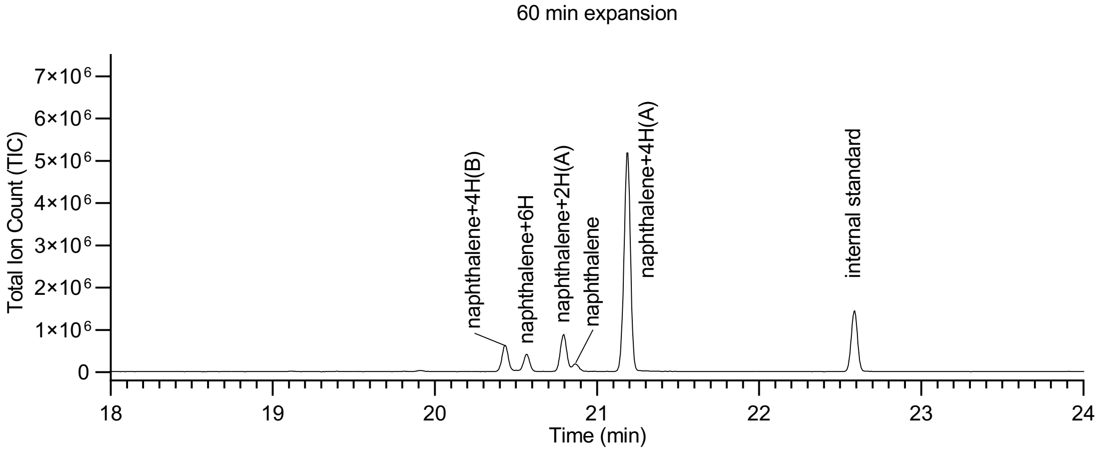

GC-MS method A

Chromatogram S35. Chromatogram of Table S5, entry 1.

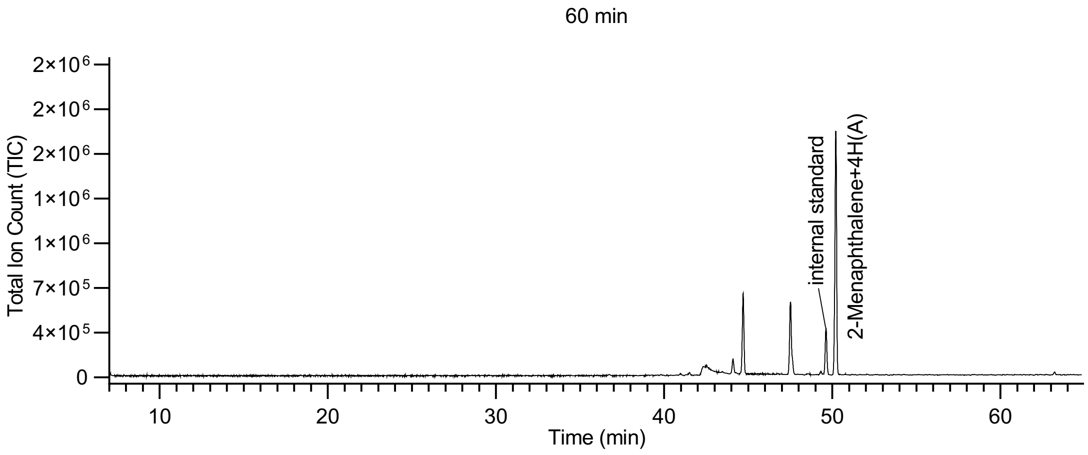

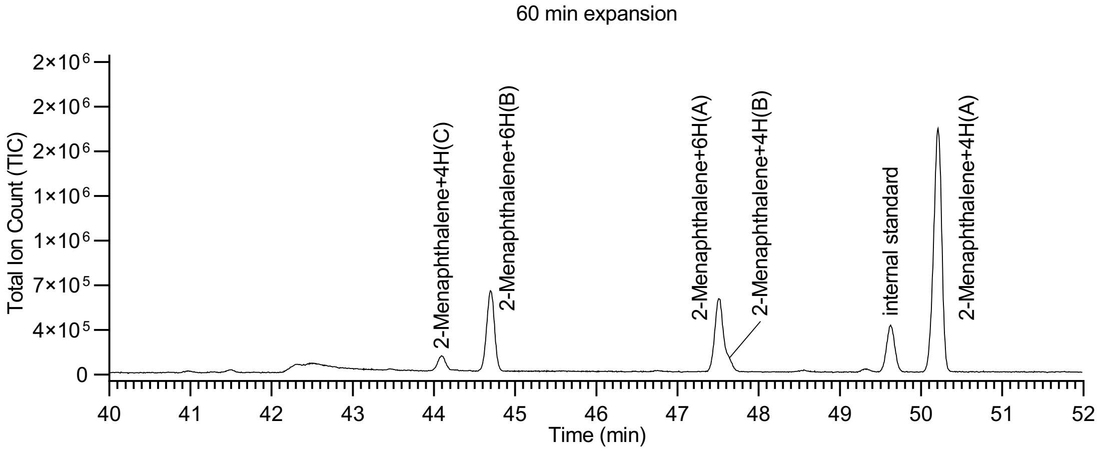

Spectrum S10. Mass spectra of 2-methylnaphthalene reduction products from Chromatogram S35 (Table S5, entry 1).

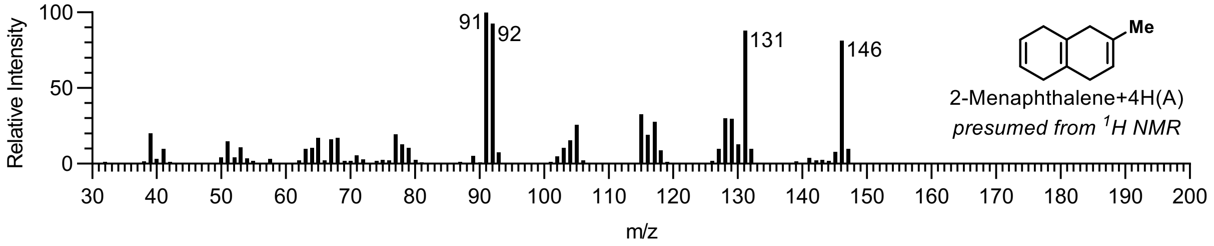

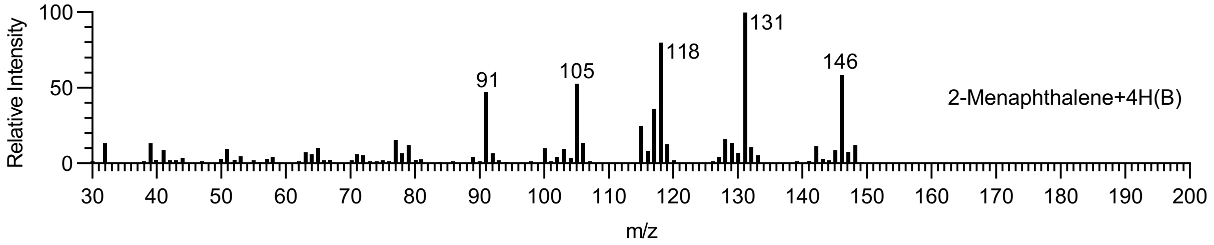

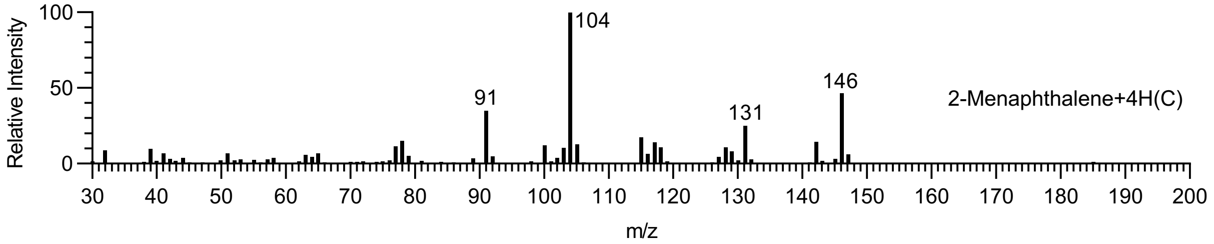

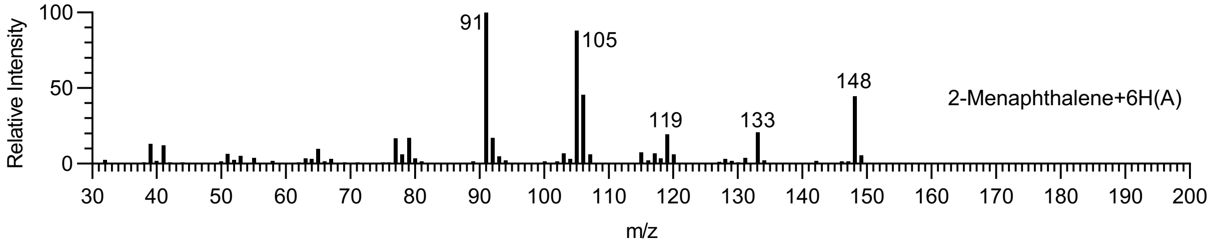

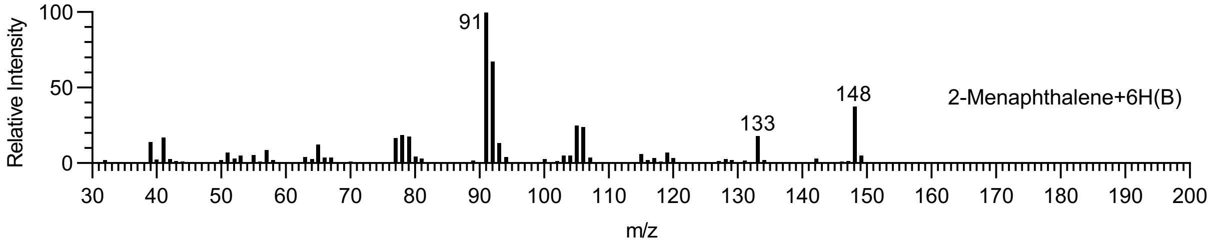

GC-MS method A

Chromatogram S36. Chromatogram of Table S5, entry 2.

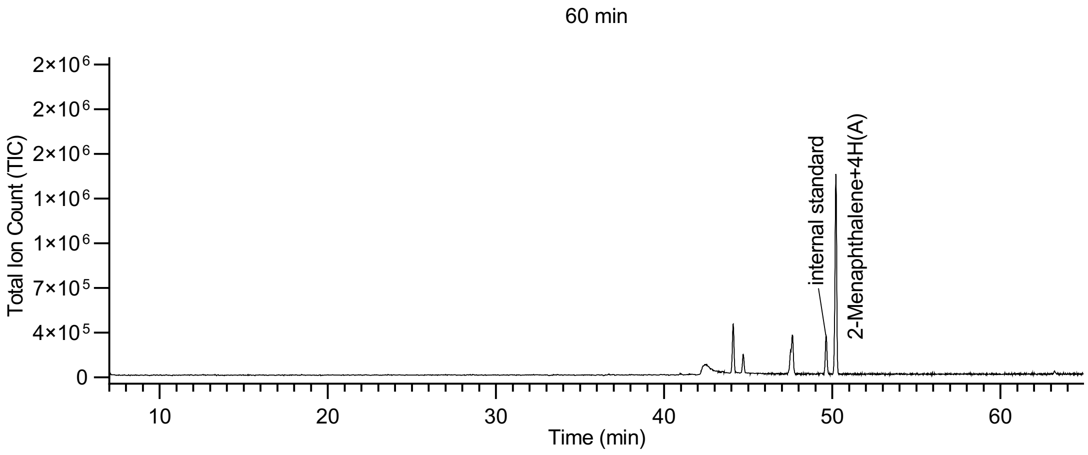

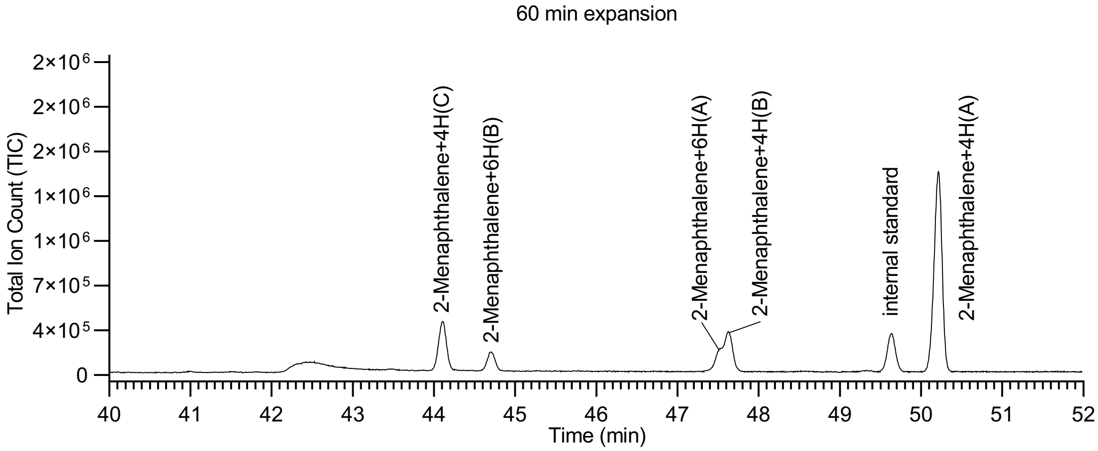

GC-MS method A

Chromatogram S37. Chromatogram of Table S5, entry 3.

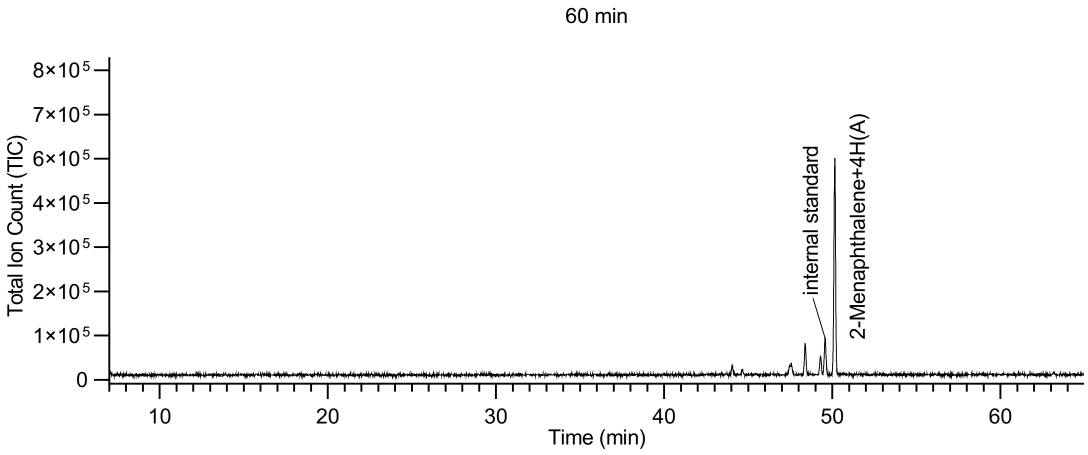

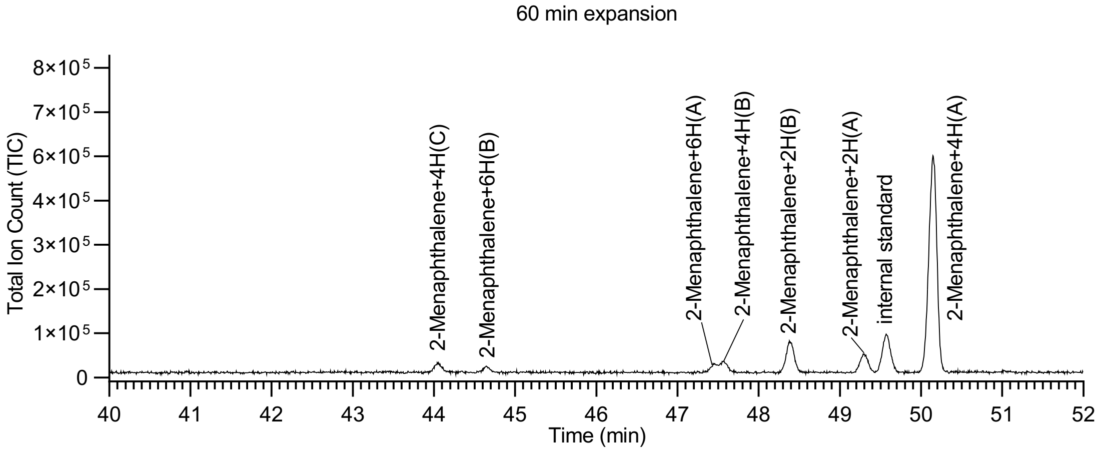

Spectrum S11. Additional mass spectra of 2-methylnaphthalene reduction products from Chromatogram S37 (Table S5, entry 3).

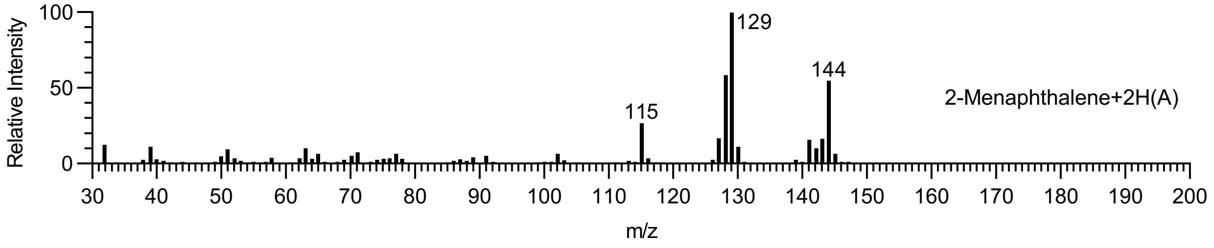

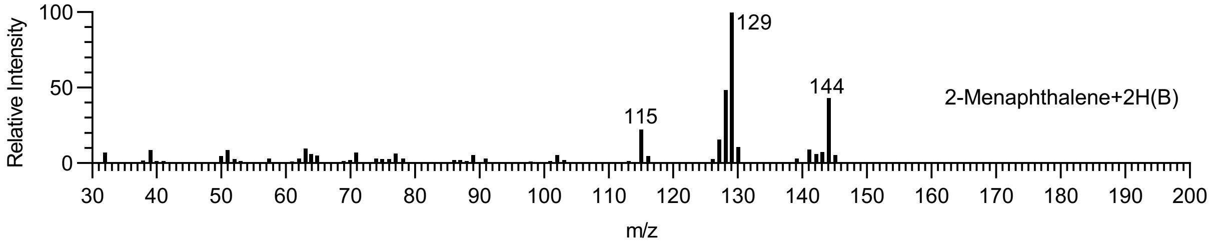

See Spectrum S10 for previously shown mass spectra of 2-methylnaphthalene reduction isomers.

GC-MS method A

Chromatogram S38. Chromatogram of Table S5, entry 4.

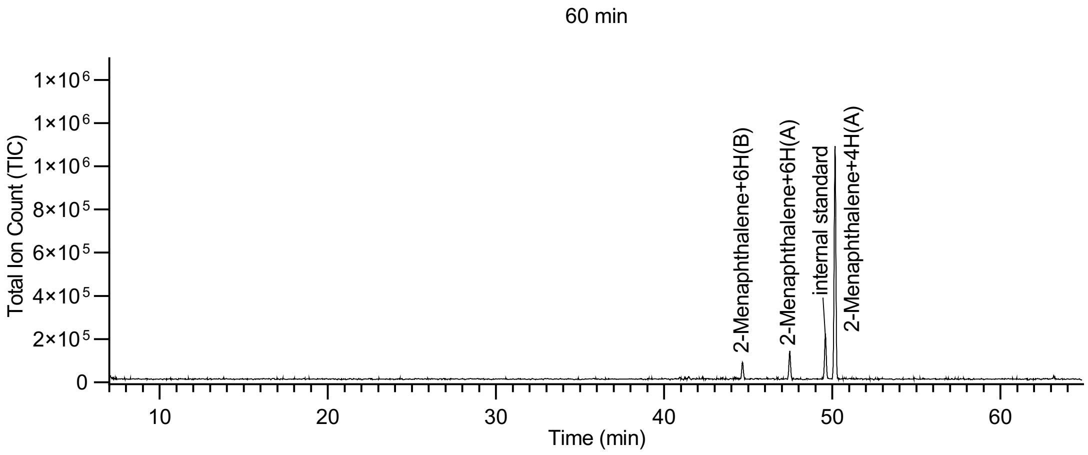

GC-MS method B

Chromatogram S39. Chromatogram of Table S5, entry 5.

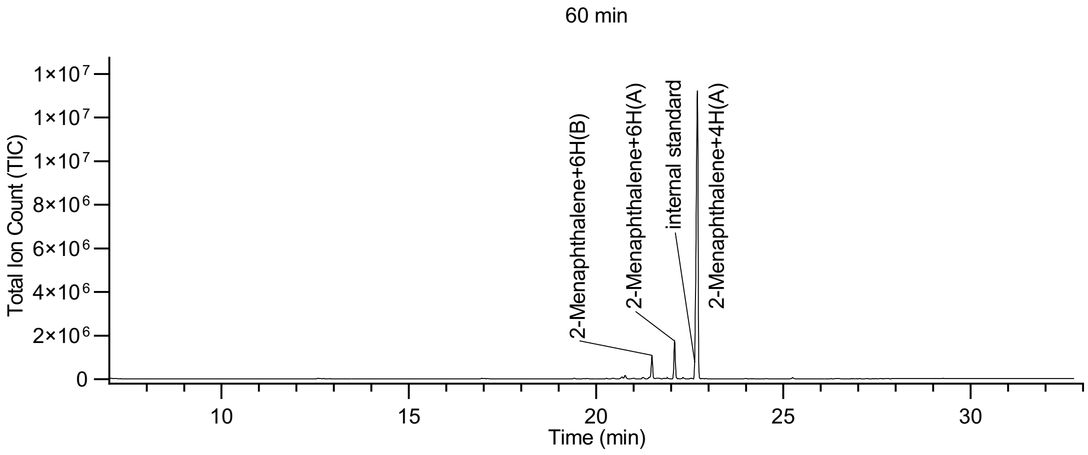

GC-MS method B

Chromatogram S40. Chromatogram of Table S5, entry 6.

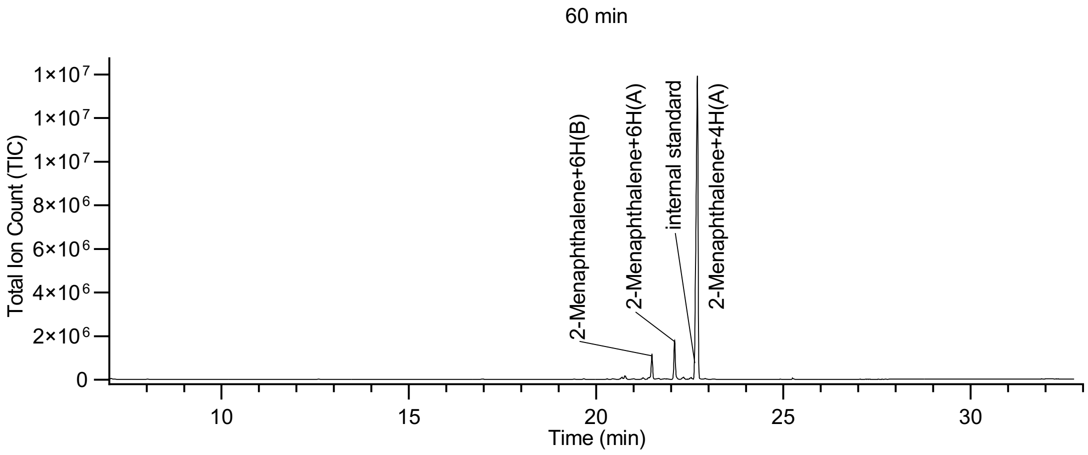

GC-MS method A

Chromatogram S41. Chromatogram of Table S6, entry 1.

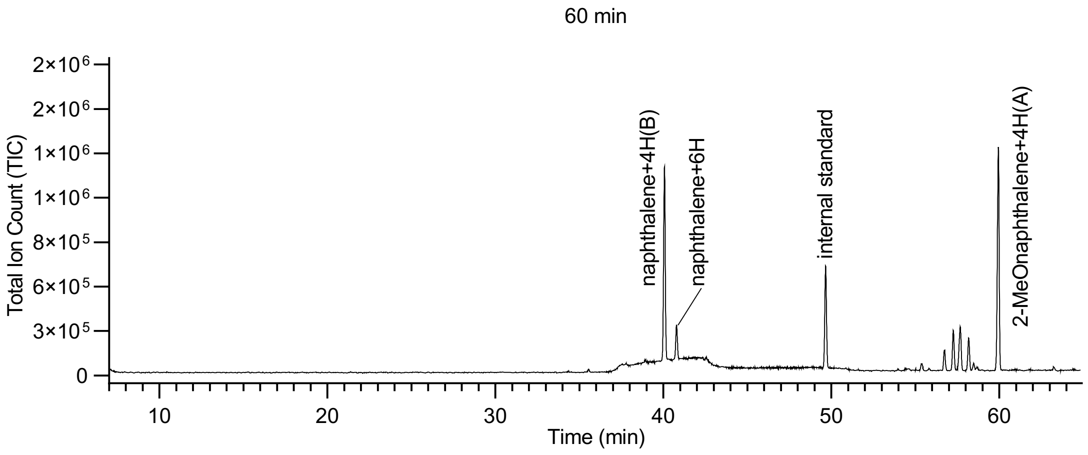

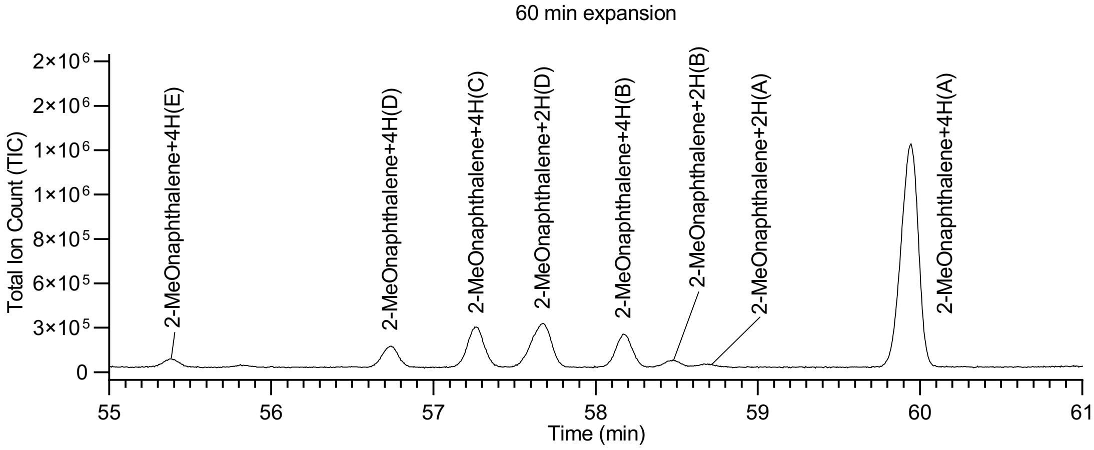

Spectrum S12. Mass spectra of 2-methoxynaphthalene reduction products from Chromatogram S41 (Table S6, entry 1).

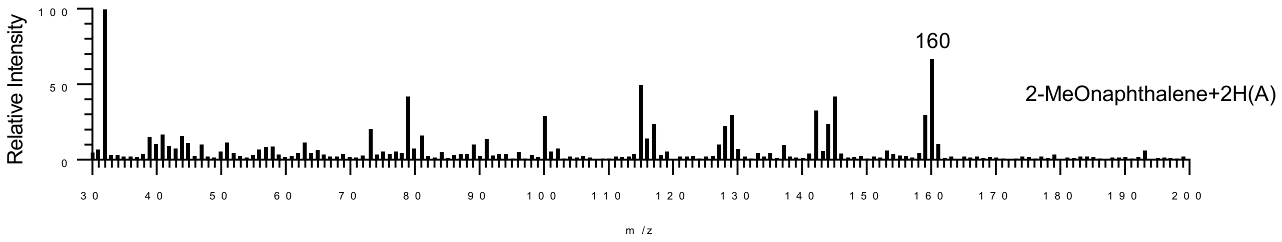

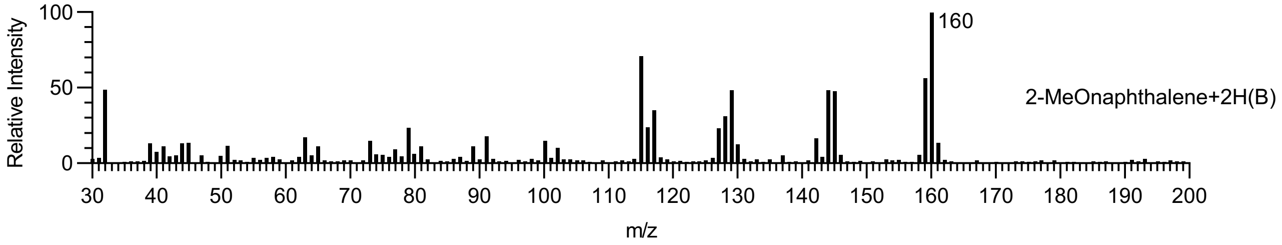

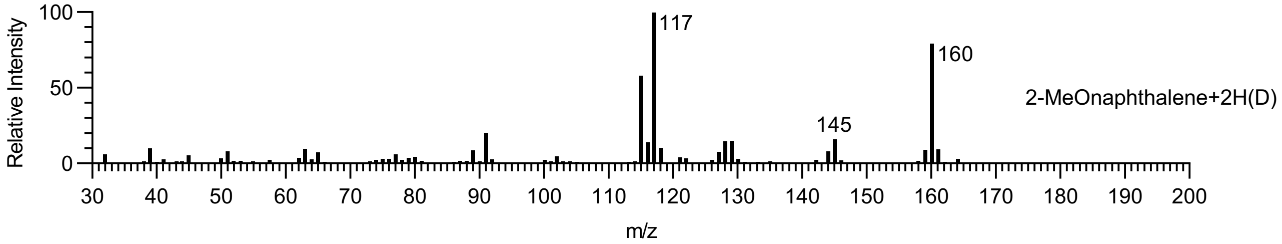

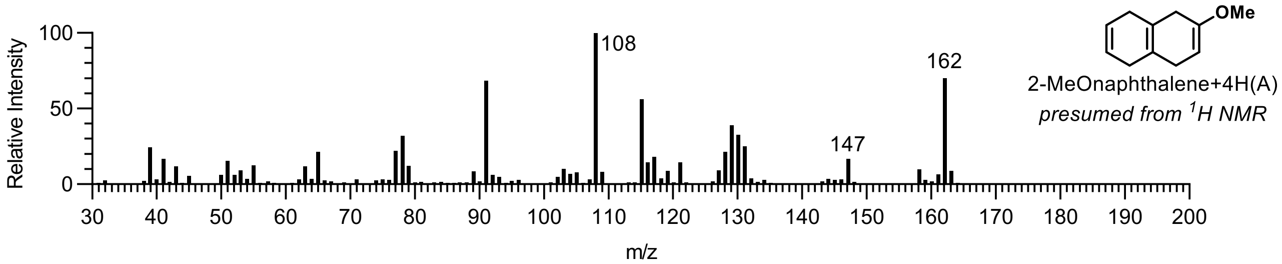

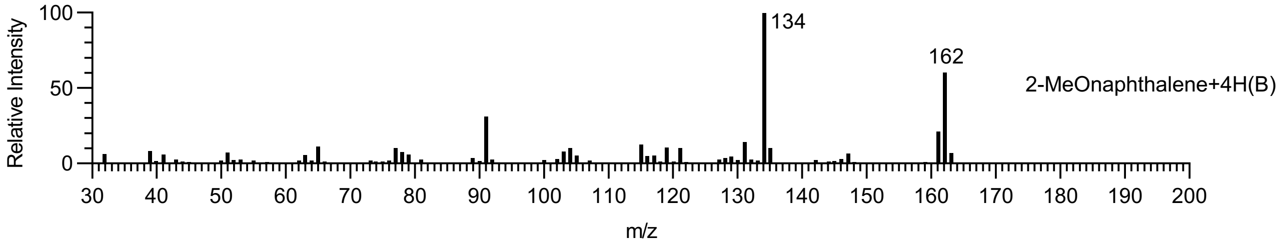

Continuation of Spectrum S12.

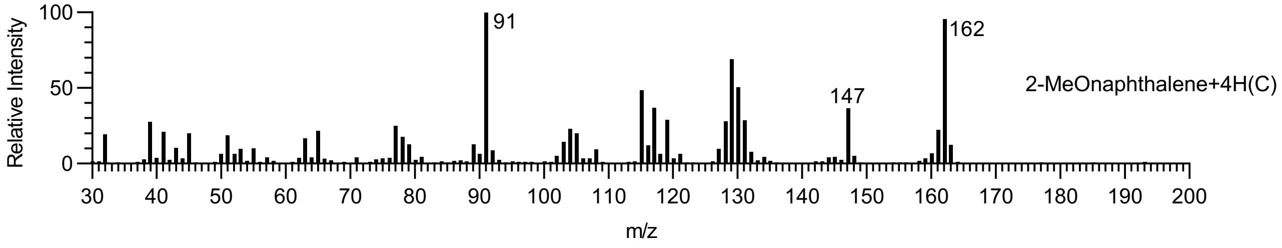

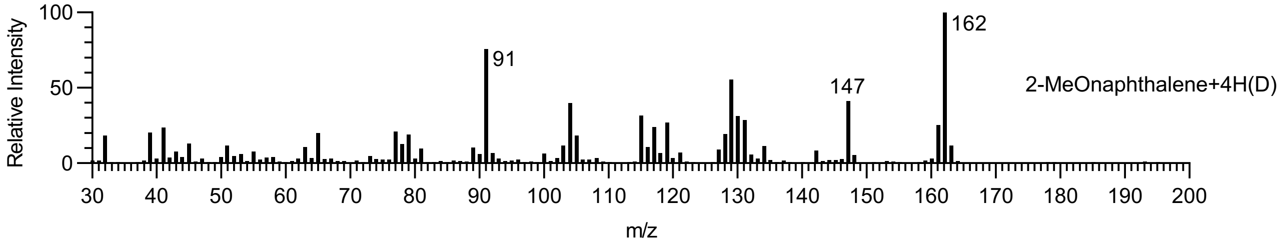

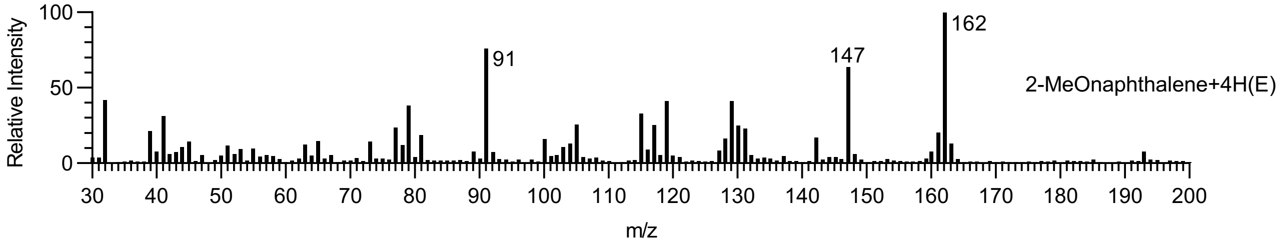

See Spectrum S8 for previously shown mass spectra of naphthalene reduction isomers.

GC-MS method A

Chromatogram S42. Chromatogram of Table S6, entry 2.

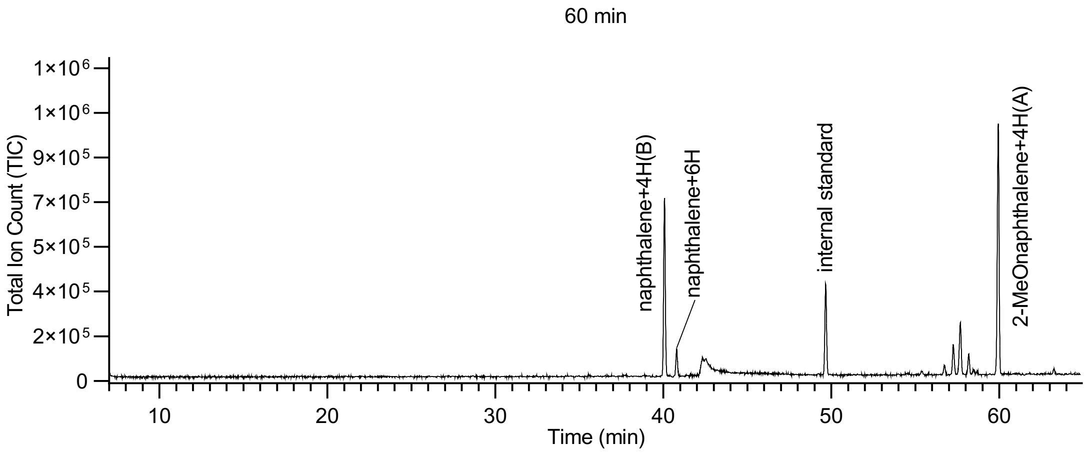

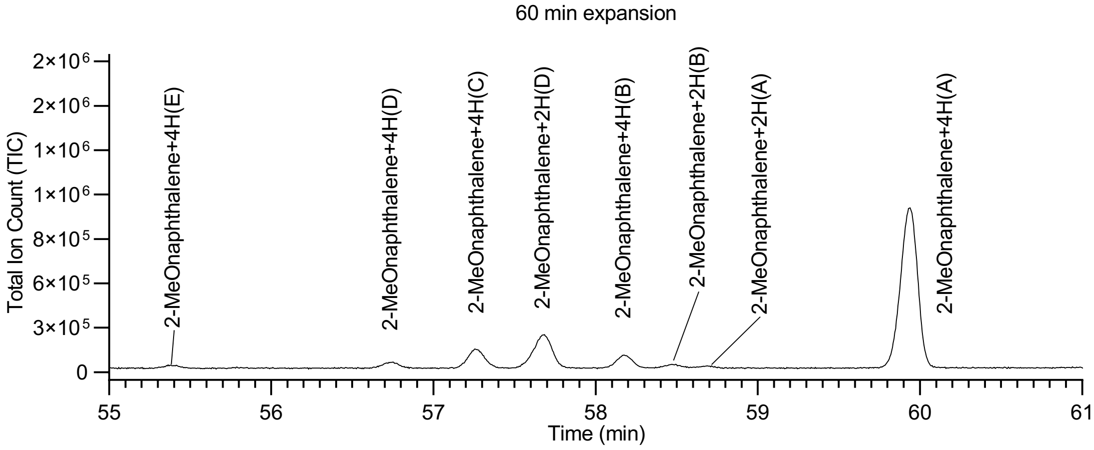

GC-MS method A

Chromatogram S43. Chromatogram of Table S6, entry 3.

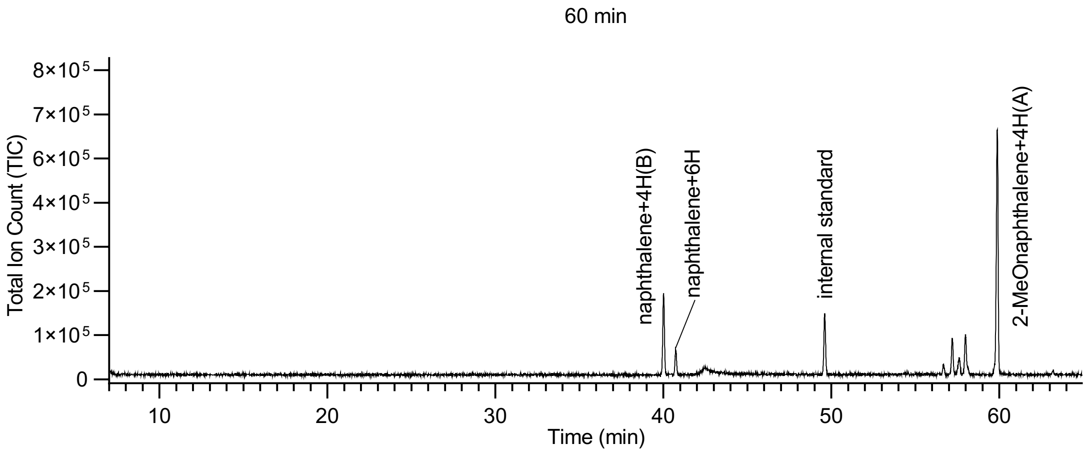

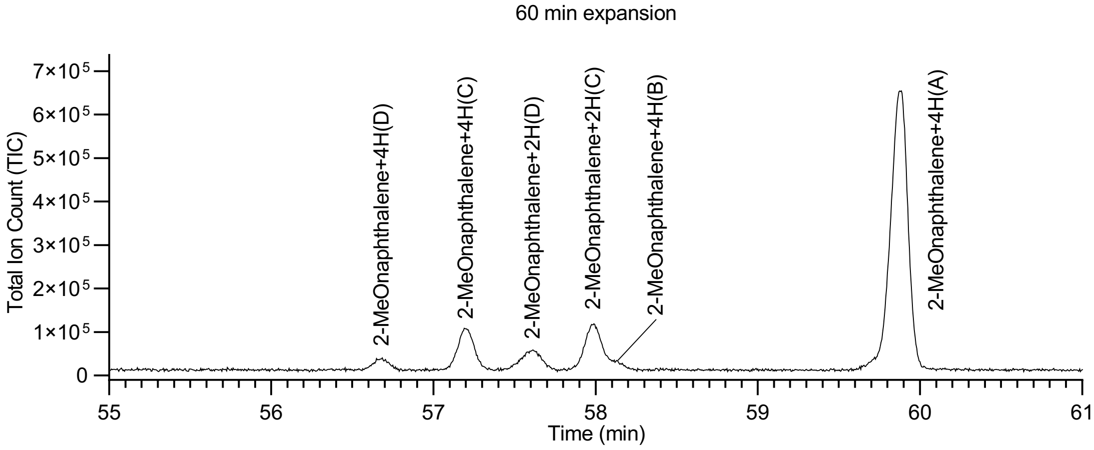

Spectrum S13. Additional mass spectra of 2-methoxynaphthalene reduction products from Chromatogram S43 (Table S6, entry 3).

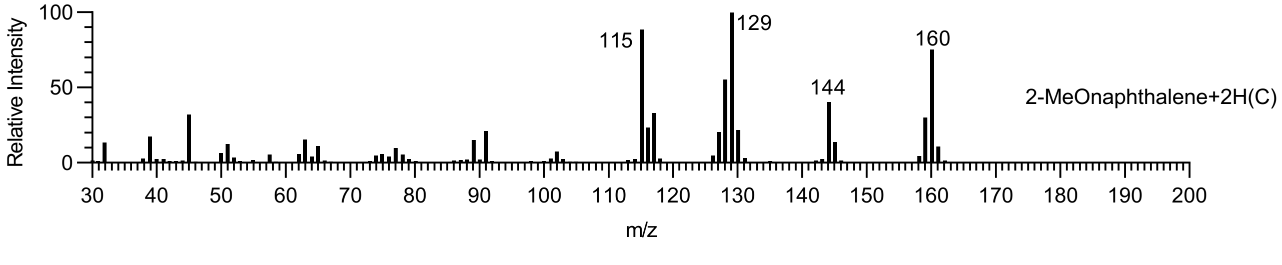

See Spectrum S12 for previously shown mass spectra of 2-methoxynaphthalene reduction isomers.

GC-MS method B

Chromatogram S44. Chromatogram of Table S6, entry 4.

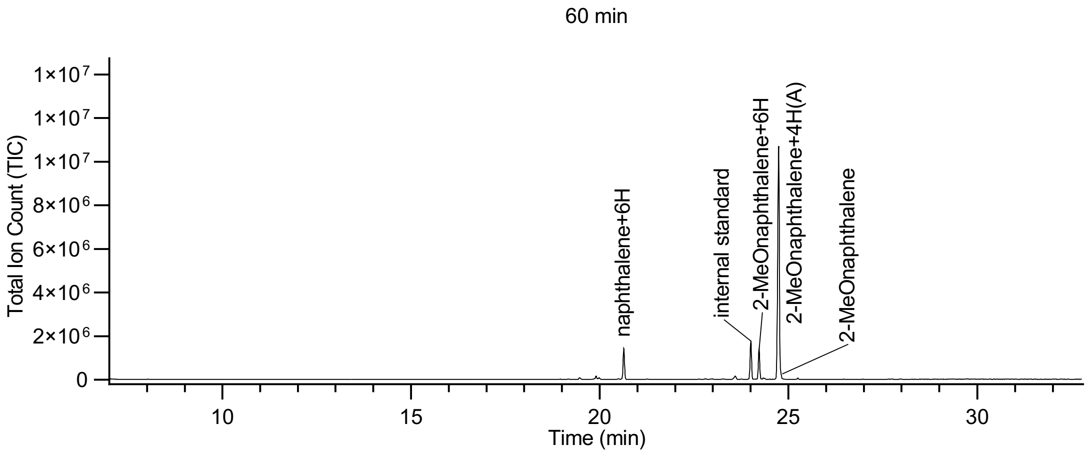

Spectrum S14. Additional mass spectra of 2-methoxynaphthalene reduction products from Chromatogram S44 (Table S6, entry 4).

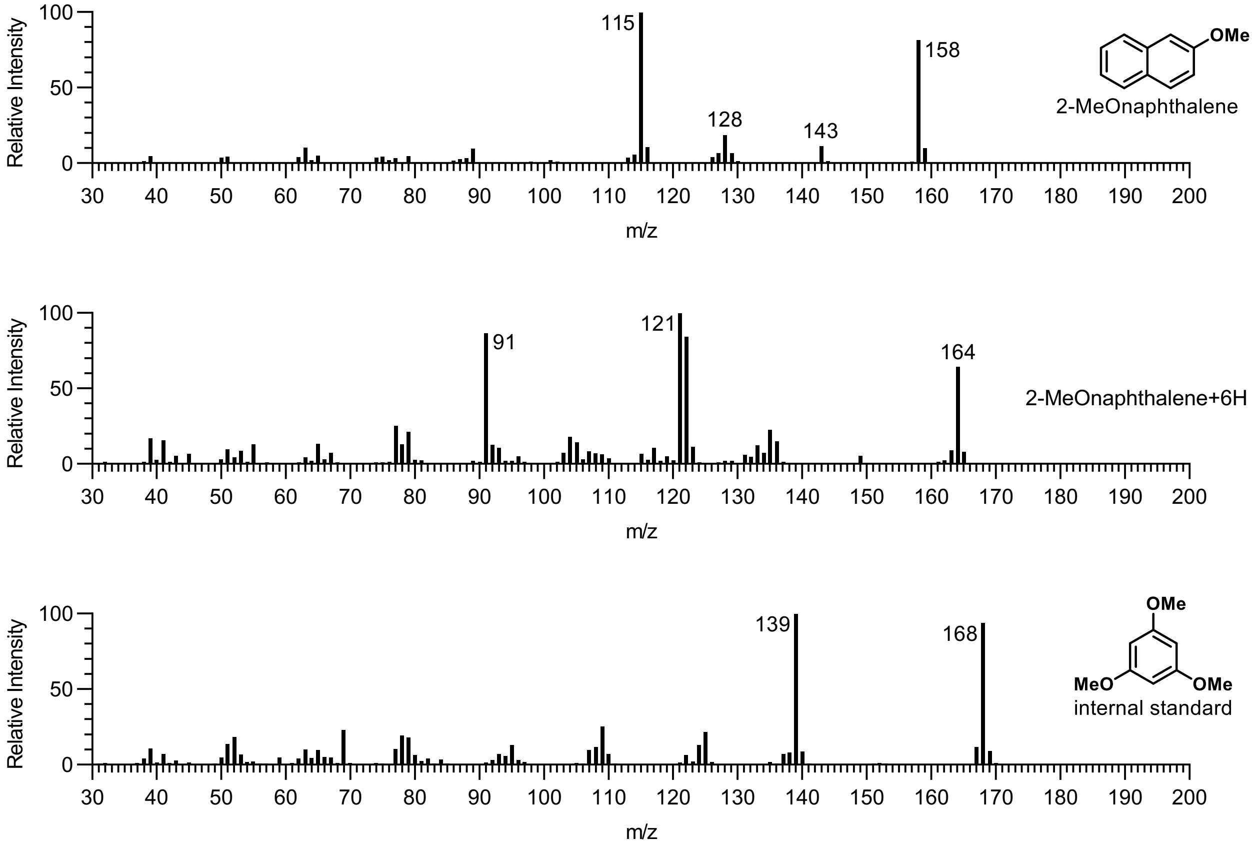

See Spectrum S8 and S12 for previously shown mass spectra of naphthalene and 2-methoxynaphthalene reduction isomers, respectively.

GC-MS method B

Chromatogram S45. Chromatogram of Table S6, entry 5.

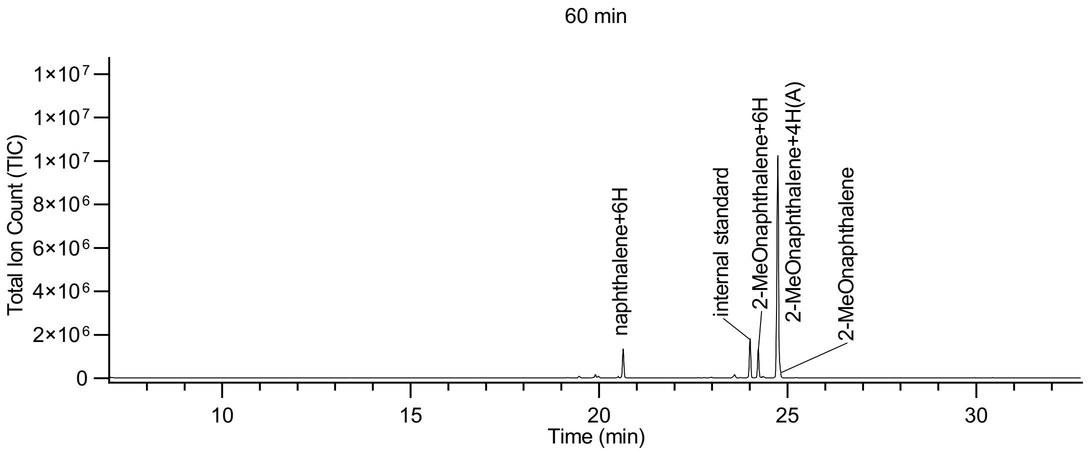

GC-MS method B

Chromatogram S46. Chromatogram of Table S6, entry 6.

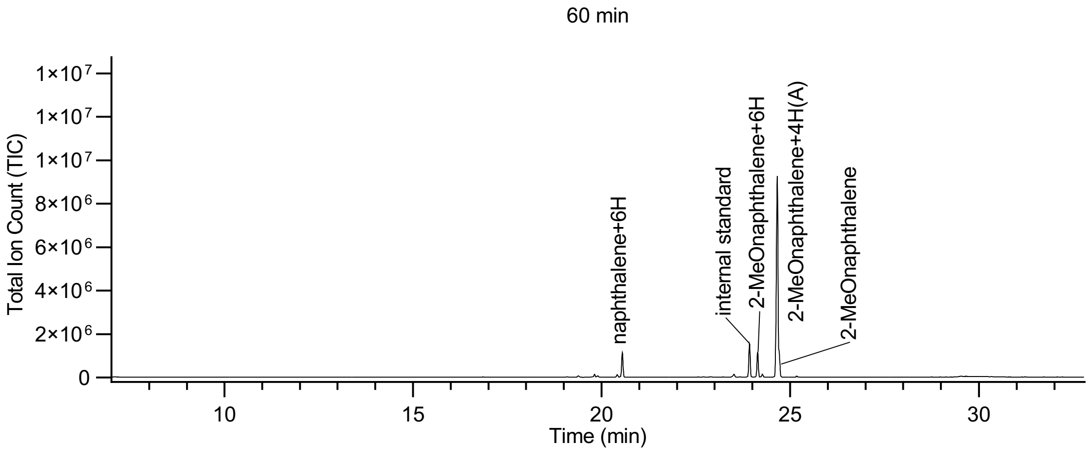

GC-MS method B

Chromatogram S47. Chromatogram of Table S7, entry 1.

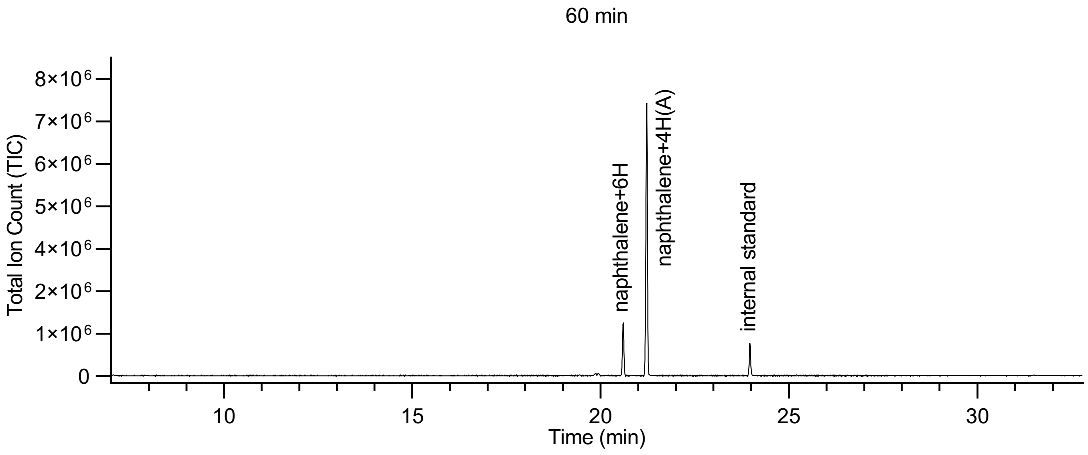

See Spectrum S8 and S9 for previously shown mass spectra of naphthalene reduction isomers.

GC-MS method B

Chromatogram S48. Chromatogram of Table S7, entry 2.

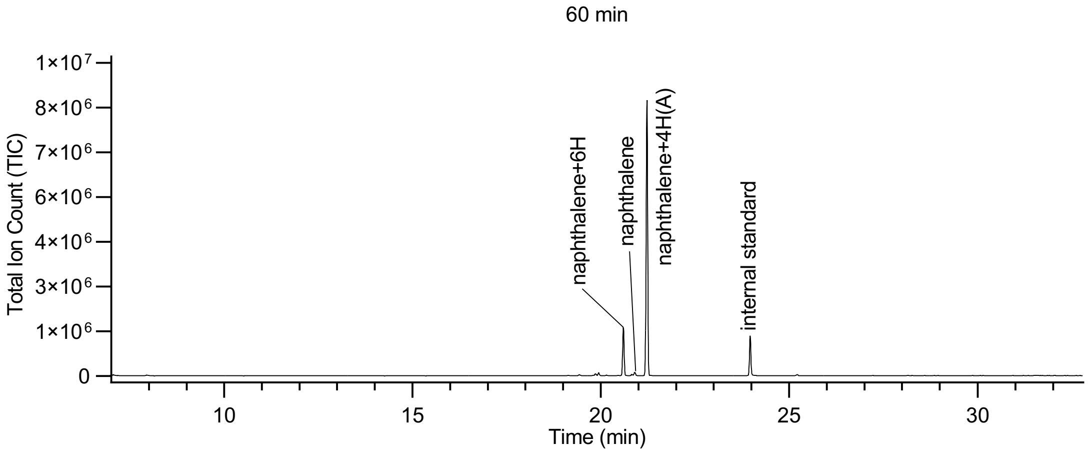

GC-MS method B

Chromatogram S49. Chromatogram of Table S7, entry 3.

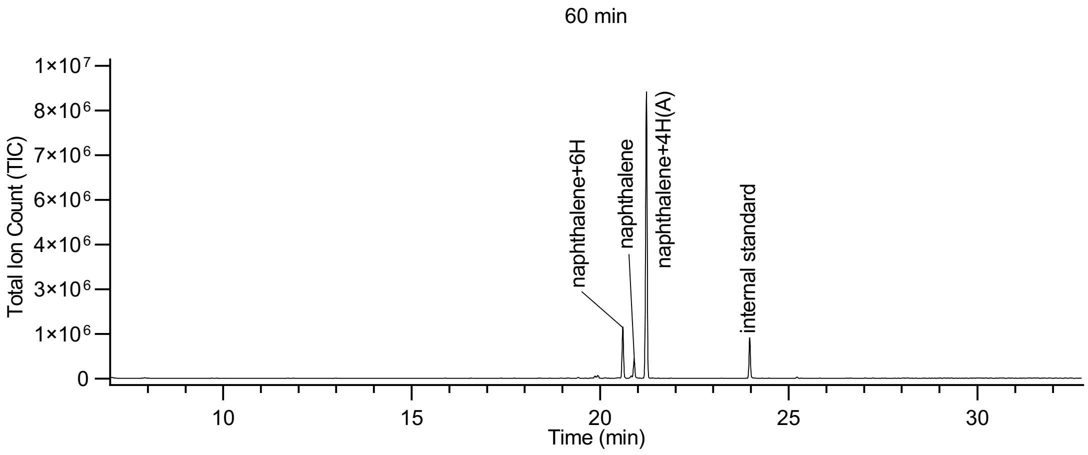

GC-MS method B

Chromatogram S50. Chromatogram of Table S9, entry 1.

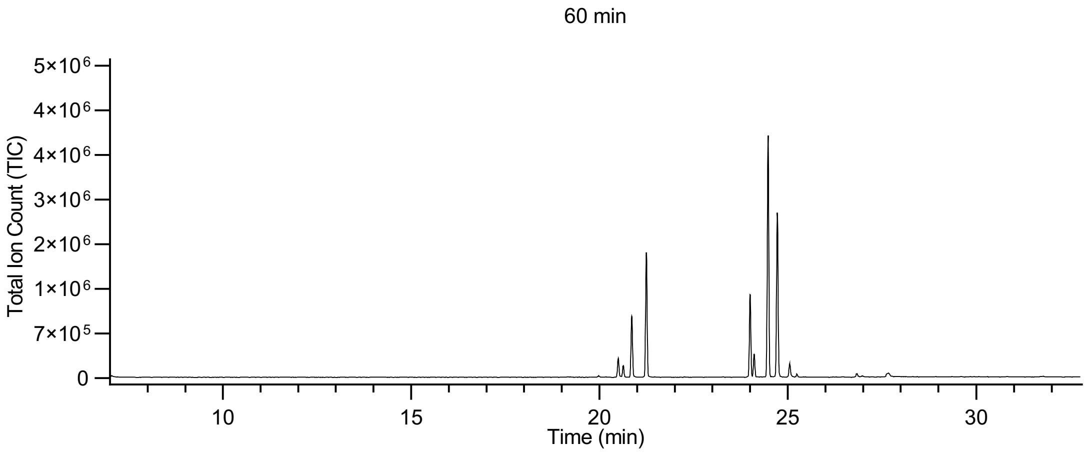

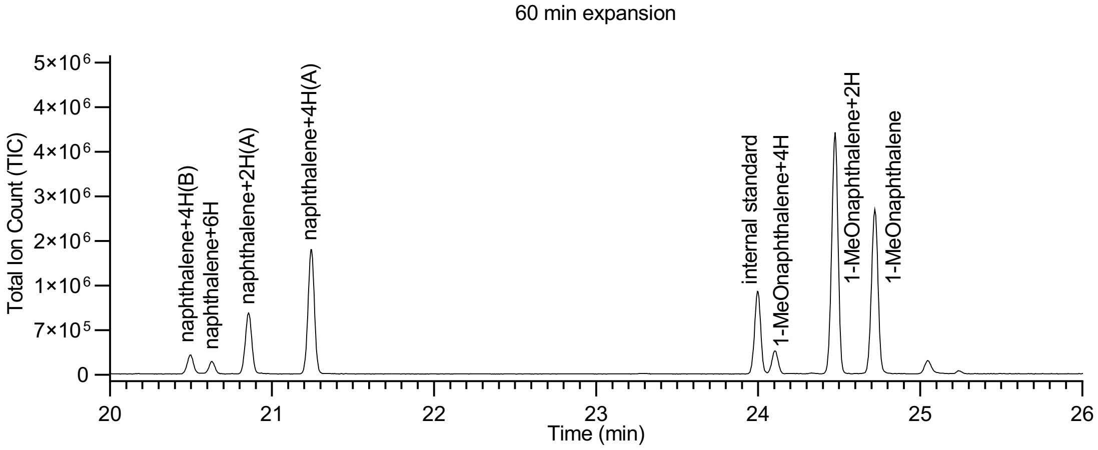

Spectrum S15. Mass spectra of 1-methoxynaphthalene reduction products from Chromatogram S50 (Table S9, entry 1).

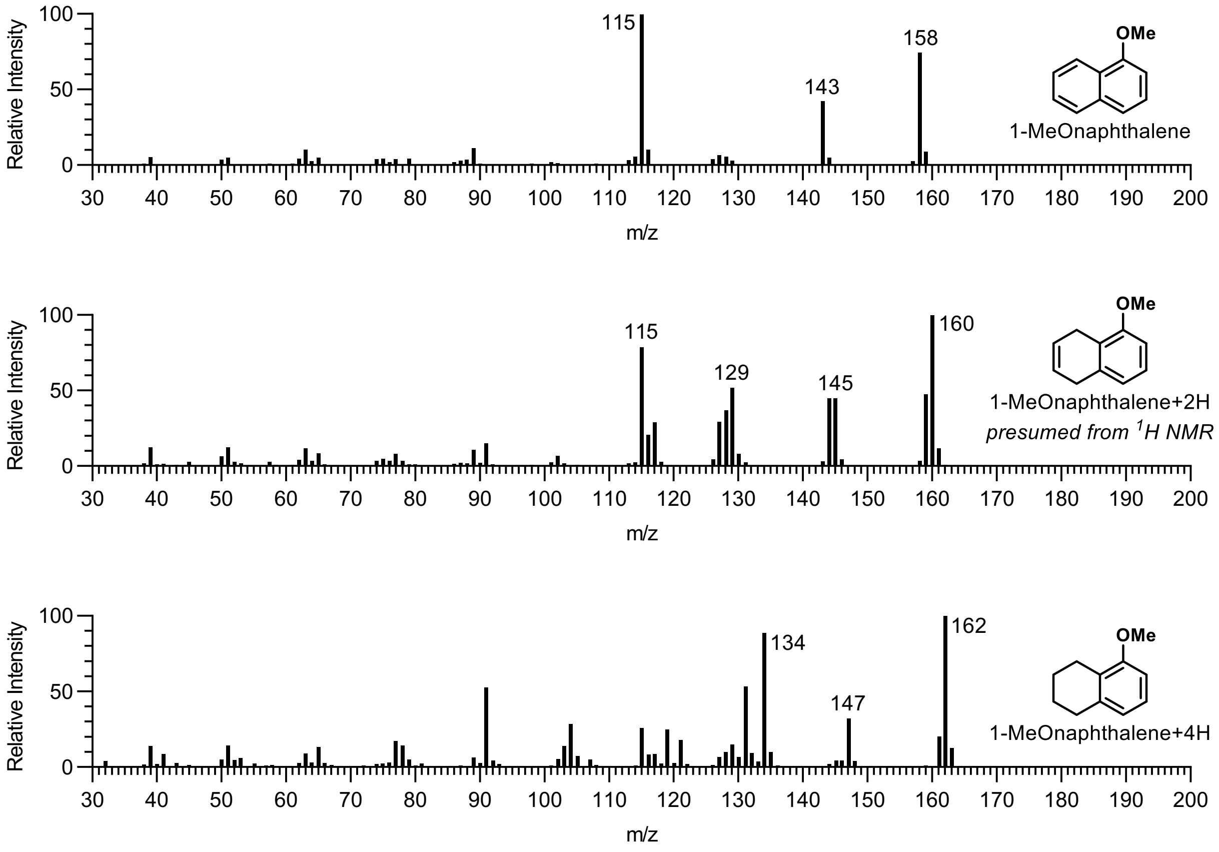

See Spectrum S8 and S9 for previously shown mass spectra of naphthalene reduction isomers.

GC-MS method B

Chromatogram S51. Chromatogram of Table S9, entry 2.

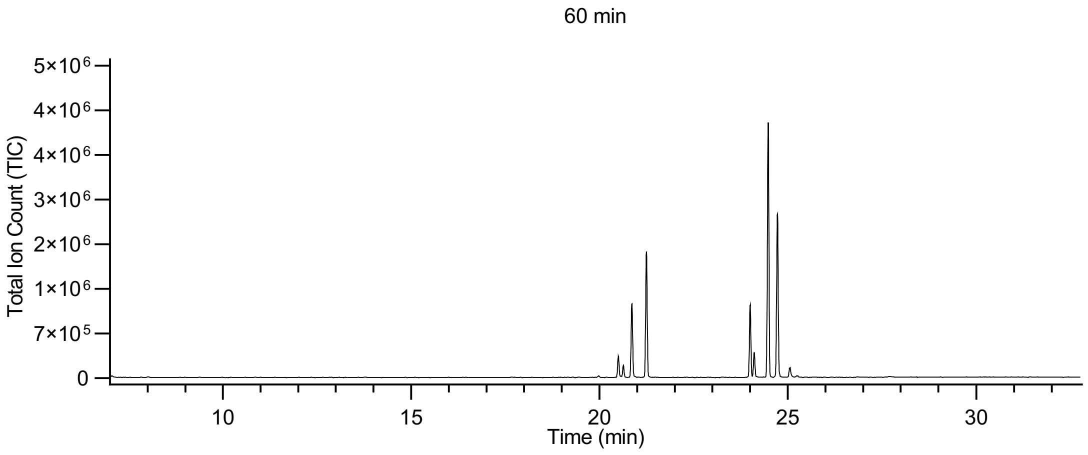

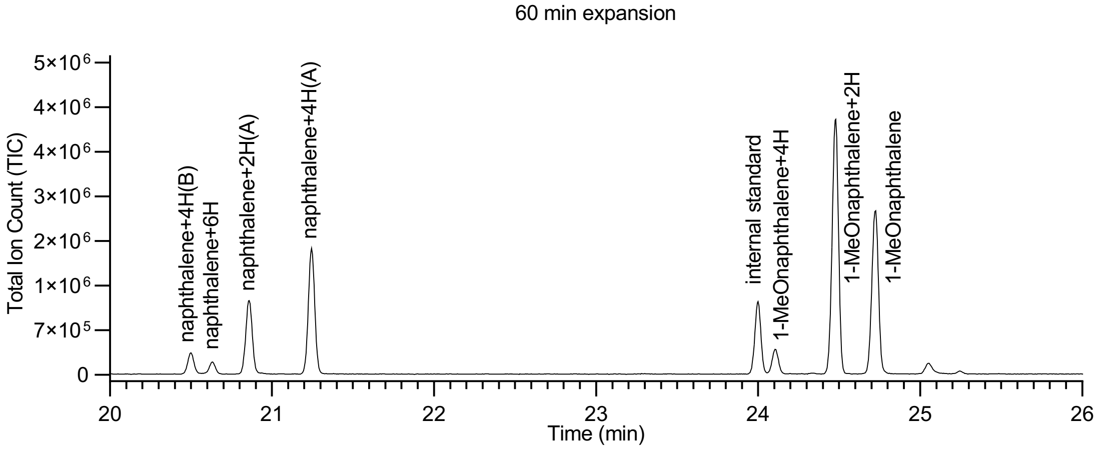

GC-MS method B

Chromatogram S52. Chromatogram of Table S9, entry 3.

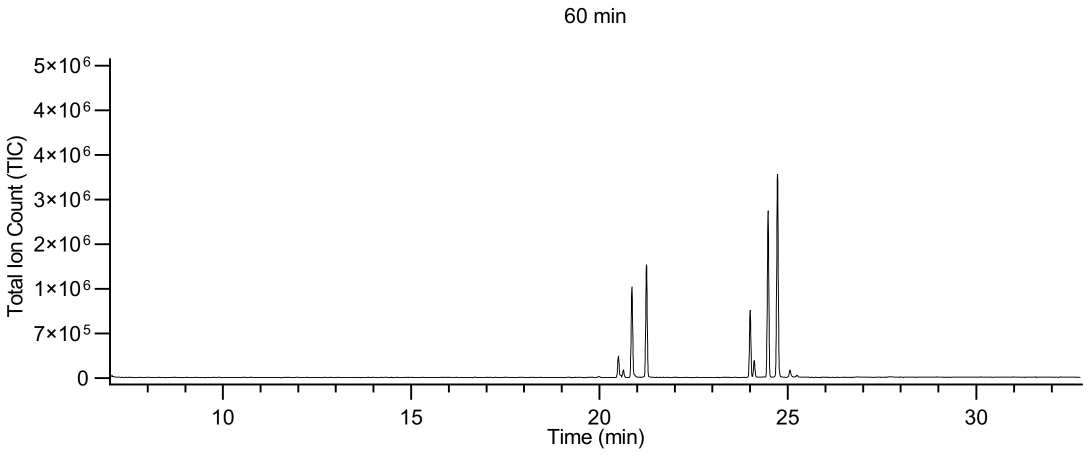

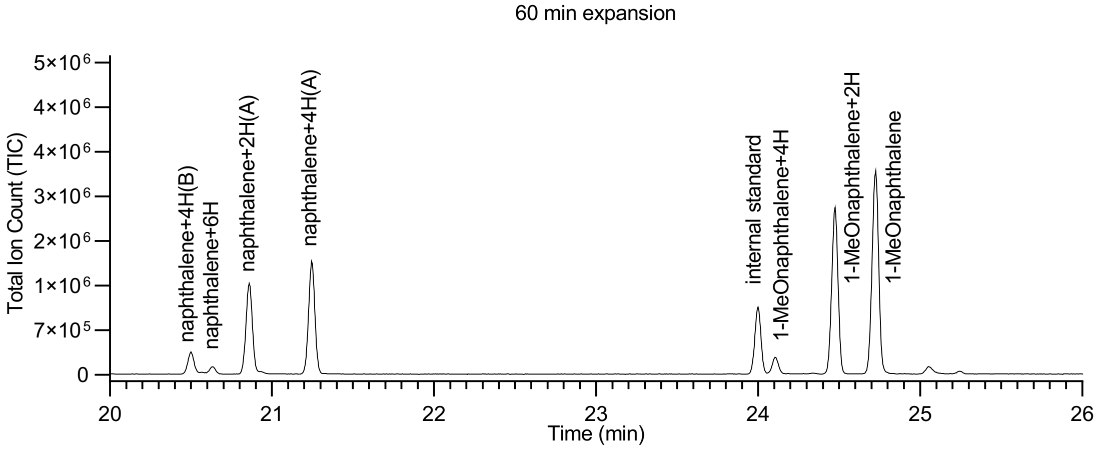

GC-MS method B

Chromatogram S53. Chromatogram of Table S10, entry 1.

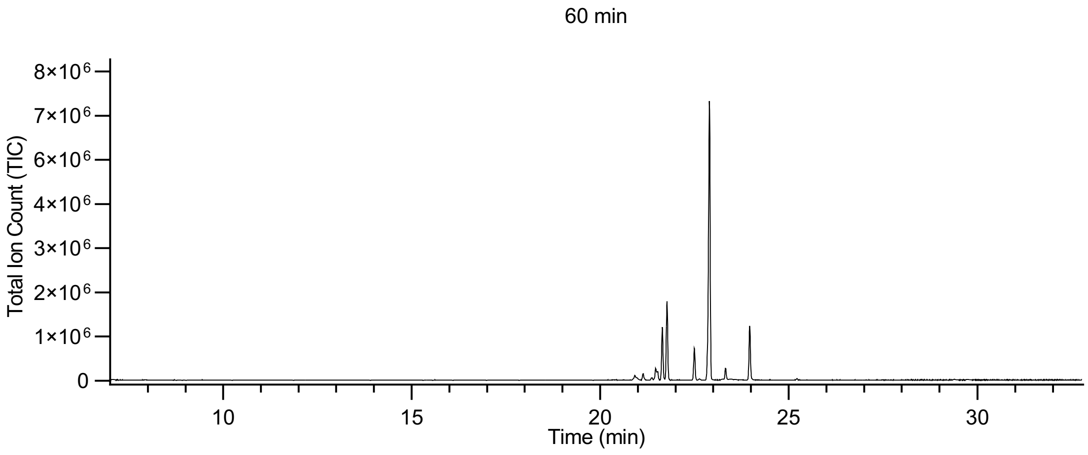

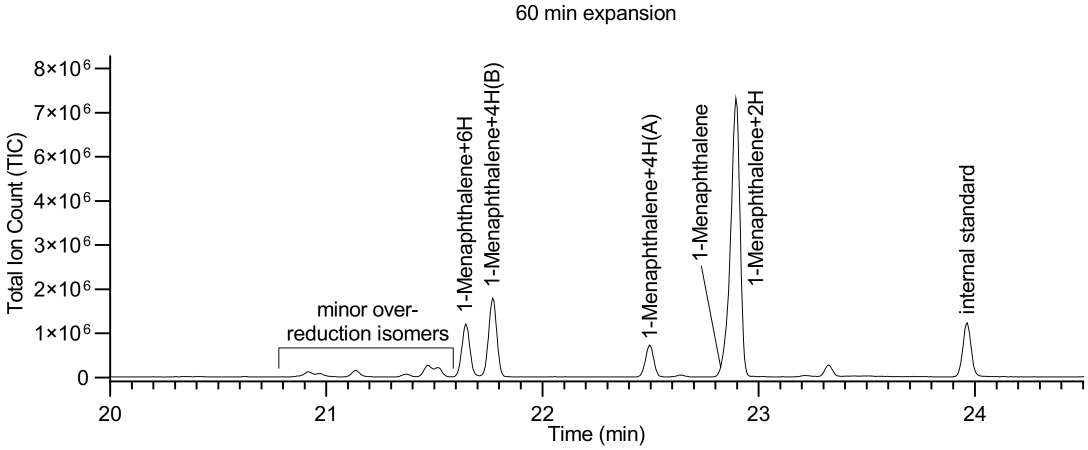

Spectrum S16. Mass spectra of 1-methylnaphthalene reduction products from Chromatogram S53 (Table S10, entry 1).

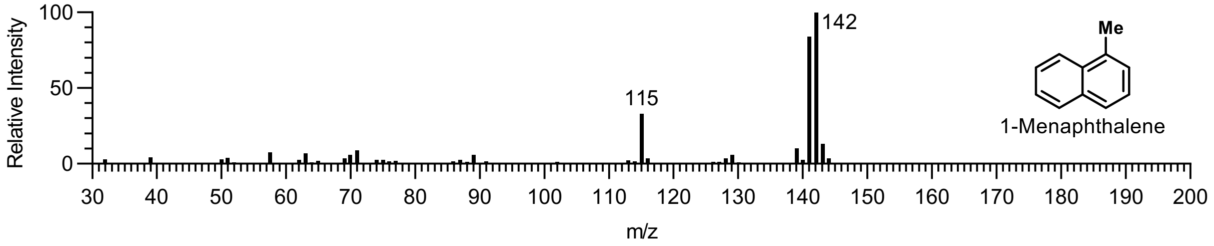

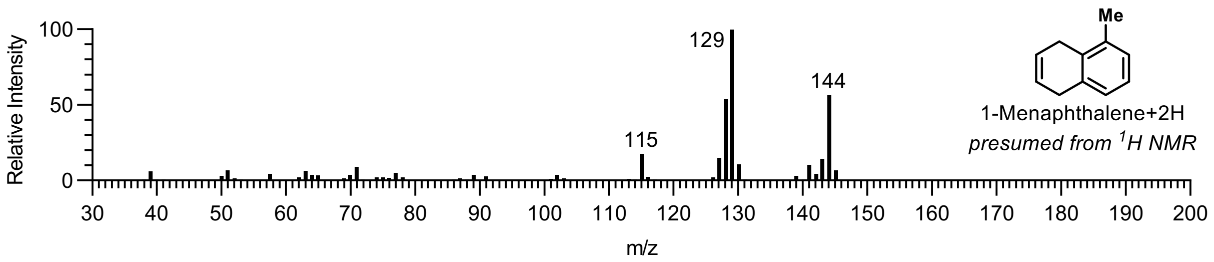

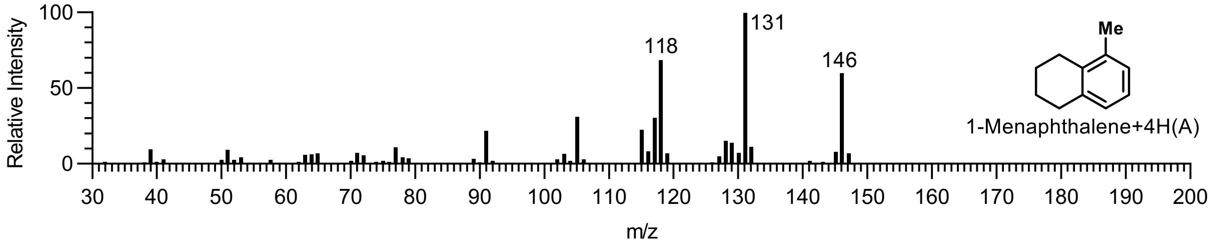

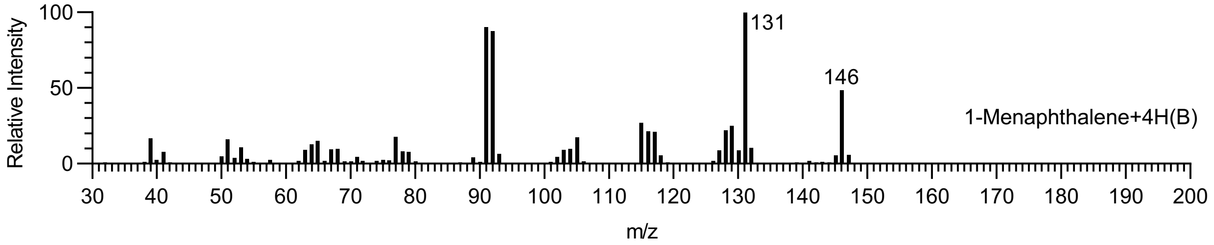

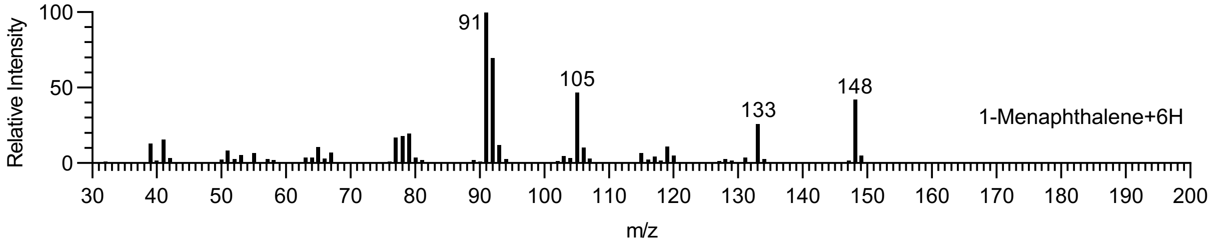

GC-MS method B

Chromatogram S54. Chromatogram of Table S10, entry 2.

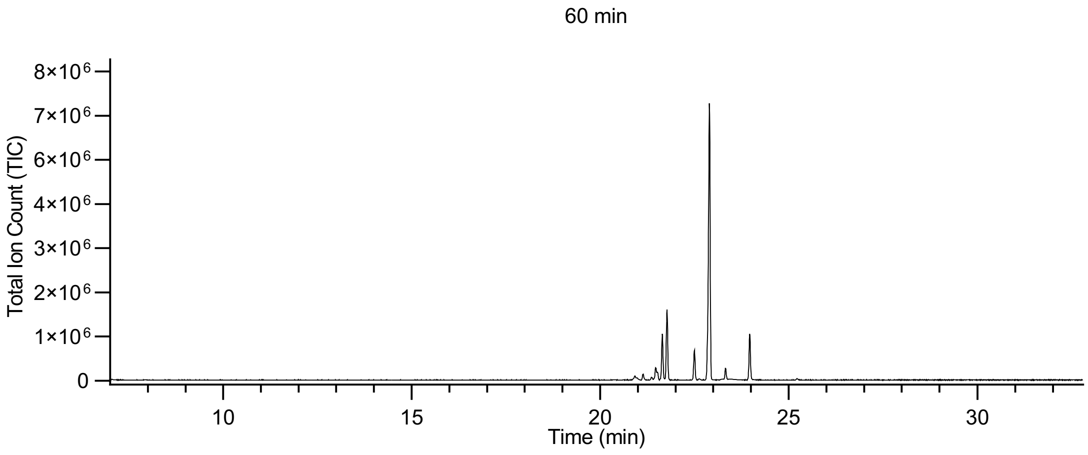

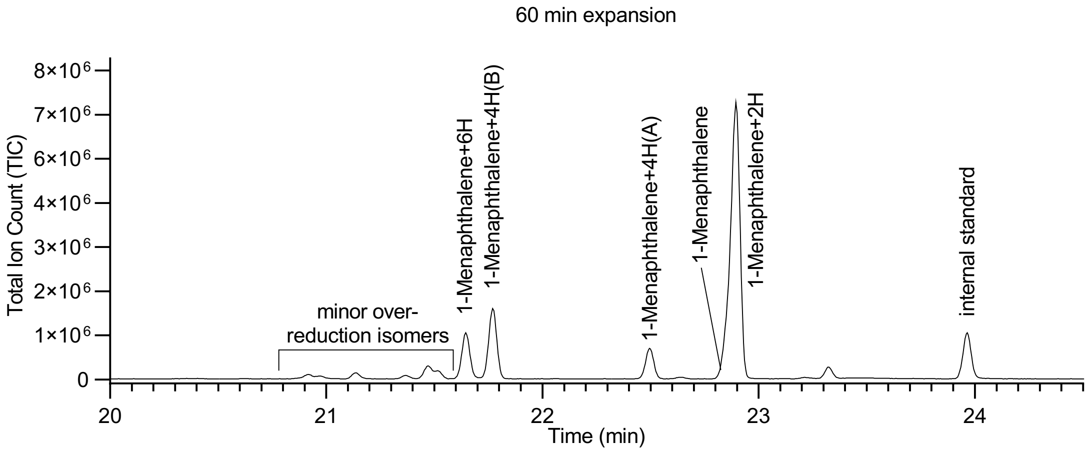

GC-MS method B

Chromatogram S55. Chromatogram of Table S10, entry 3.

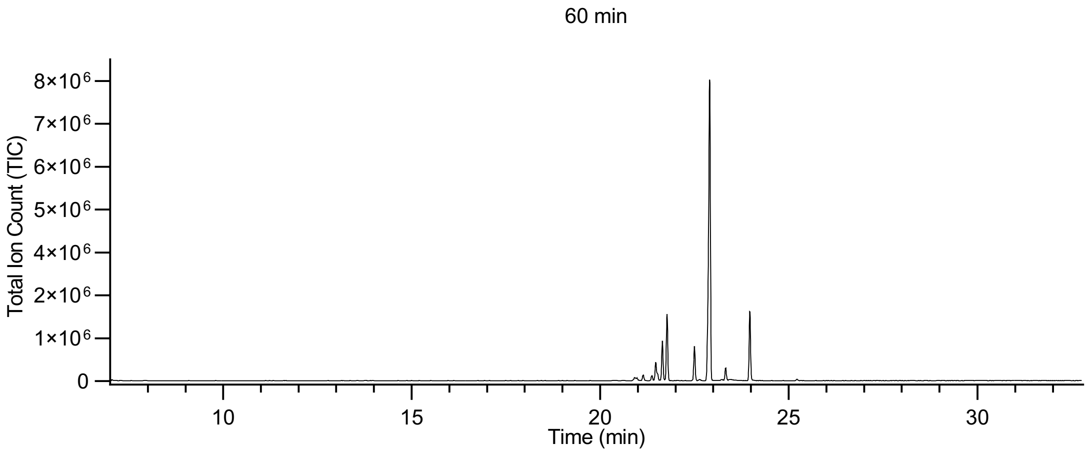

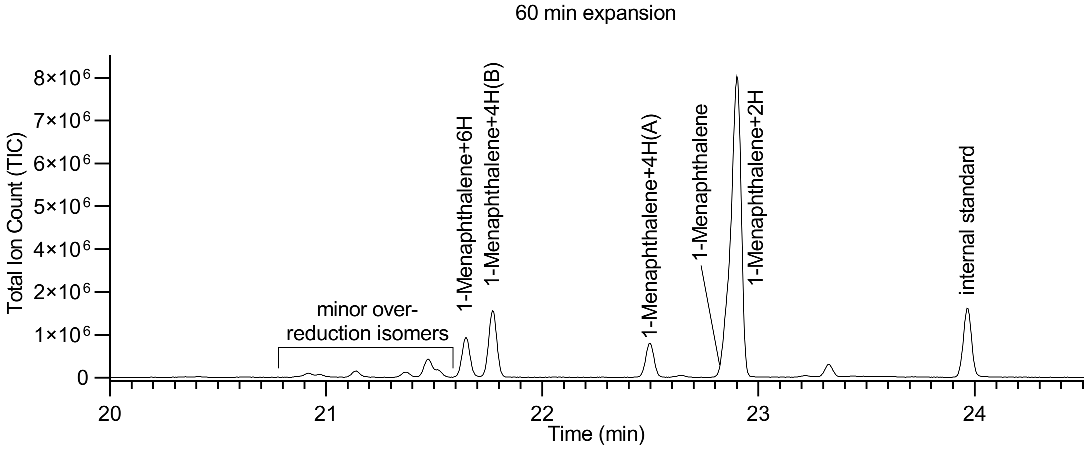

GC-MS method B

Chromatogram S56. Chromatogram of Table S13, entry 1.

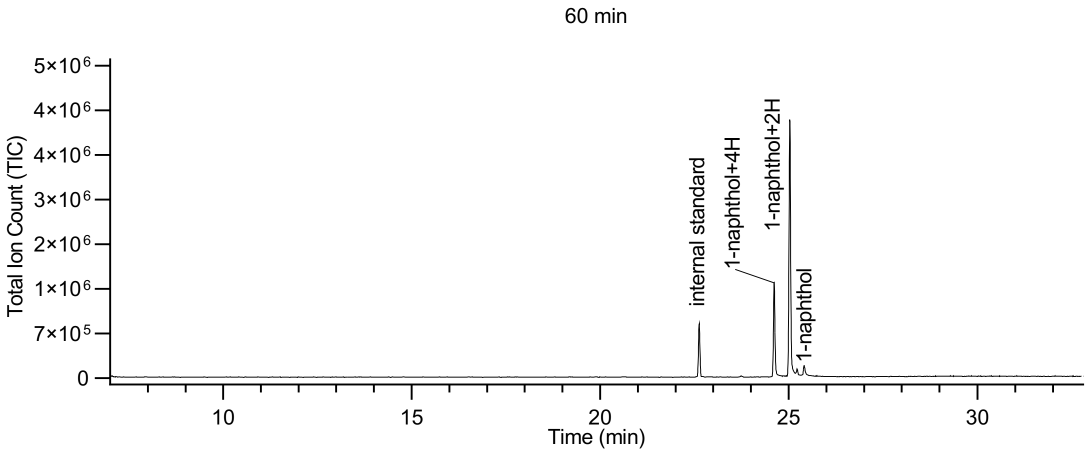

Spectrum S17. Mass spectra of 1-naphthol reduction products from Chromatogram S56 (Table S13, entry 1).

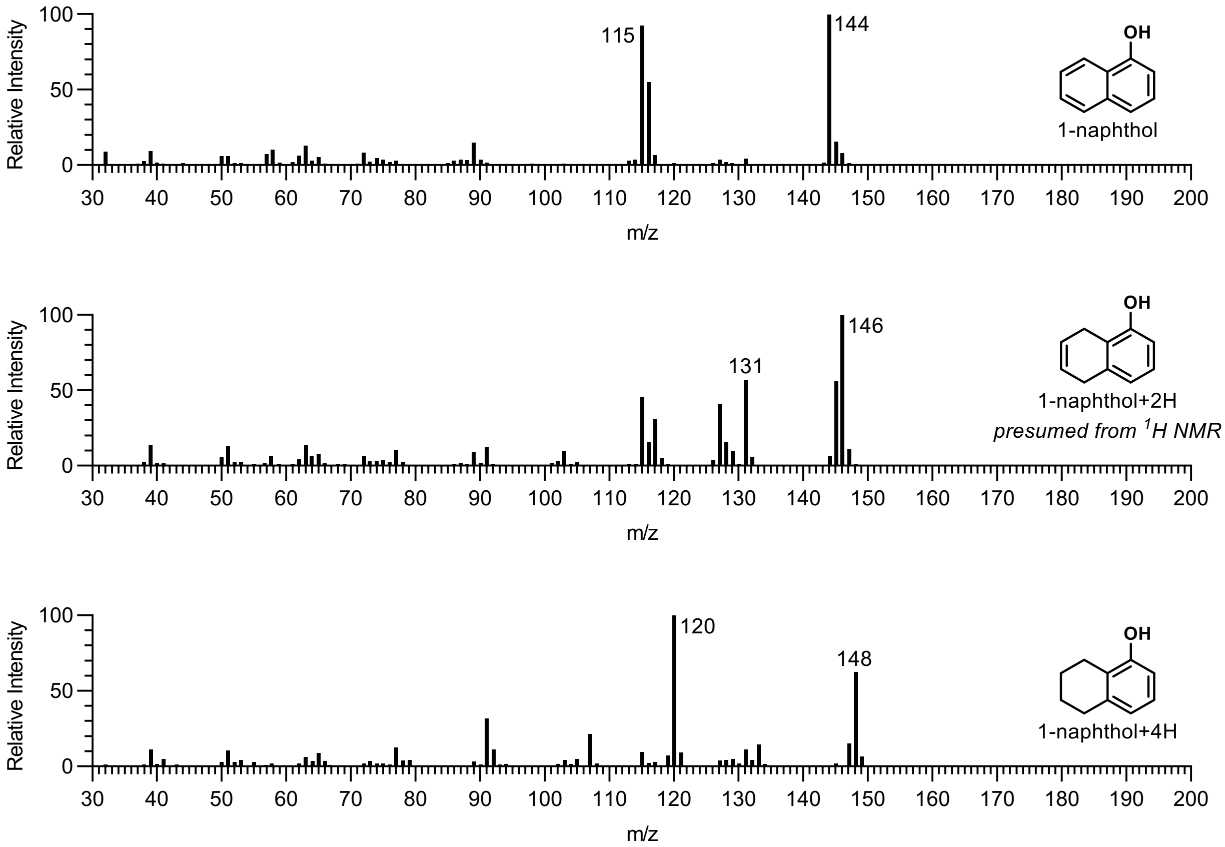

GC-MS method B

Chromatogram S57. Chromatogram of Table S13, entry 2.

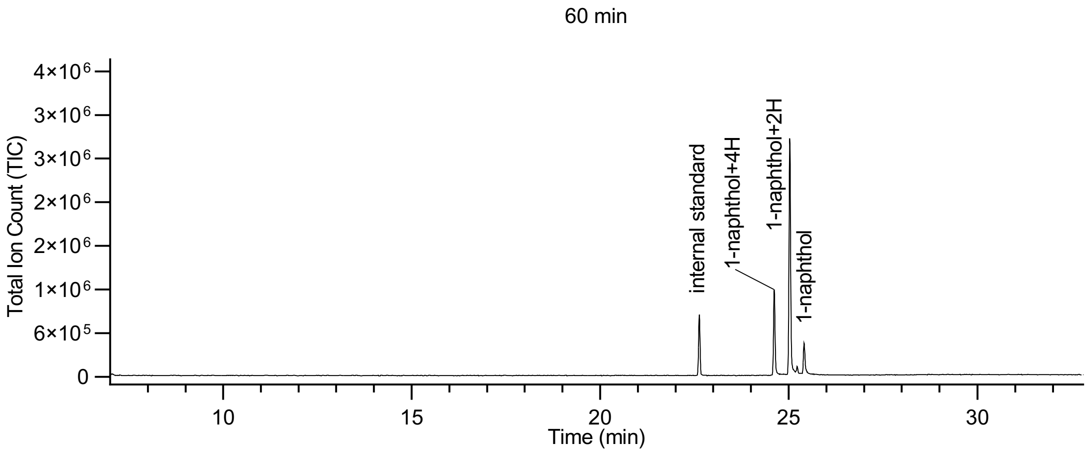

GC-MS method B

Chromatogram S58. Chromatogram of Table S13, entry 3.

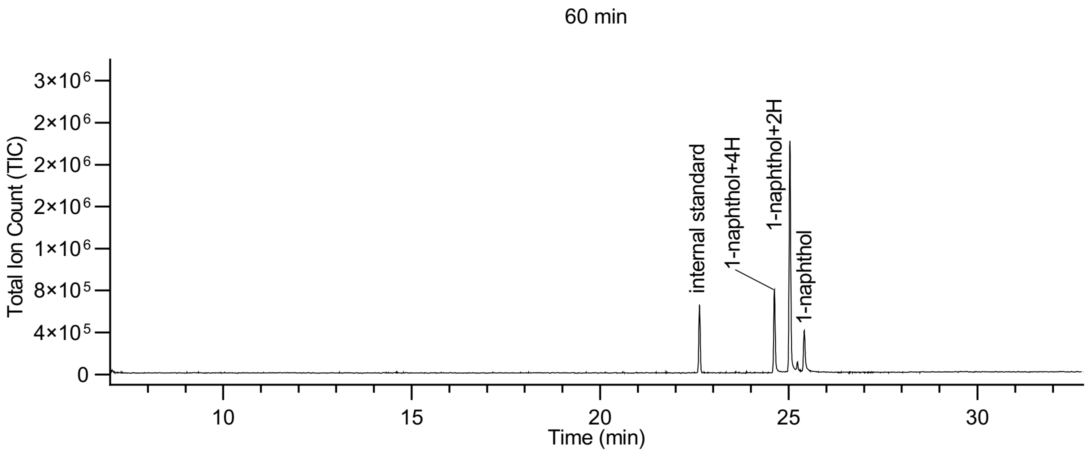

GC-MS method B

Chromatogram S59. Chromatogram of Table S14, entry 1.

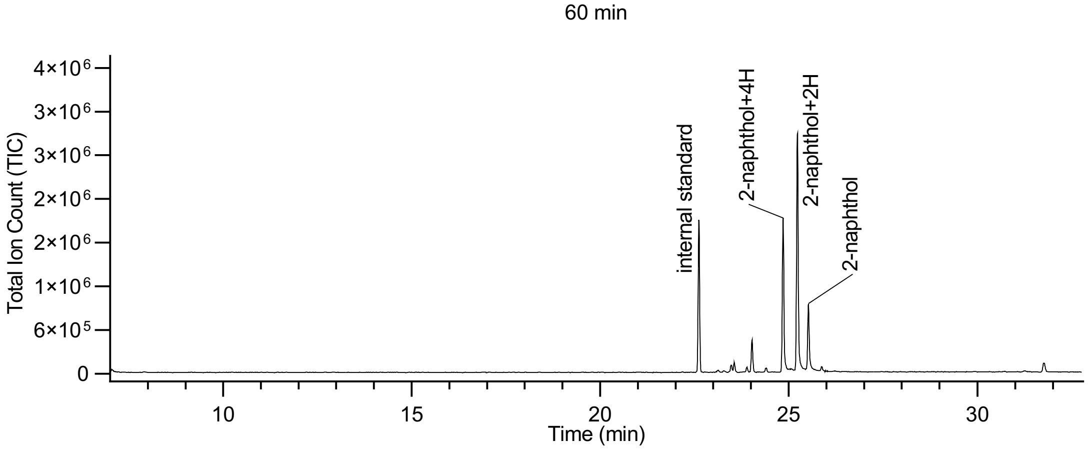

Spectrum S18. Mass spectra of 2-naphthol reduction products from Chromatogram S59 (Table S14, entry 1).

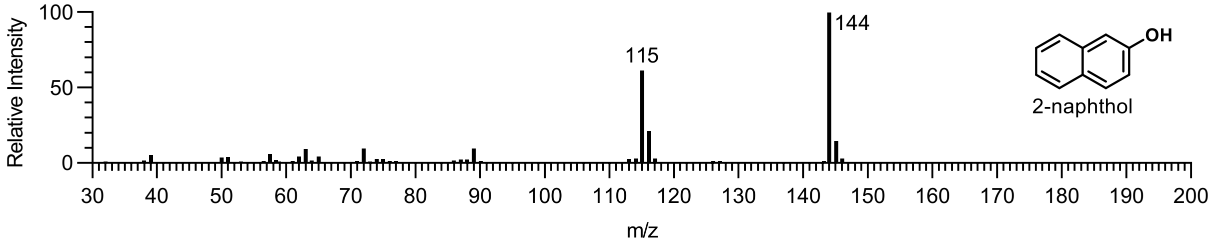

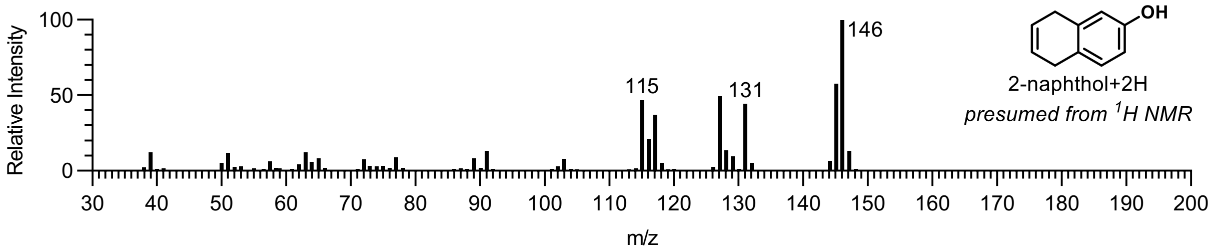

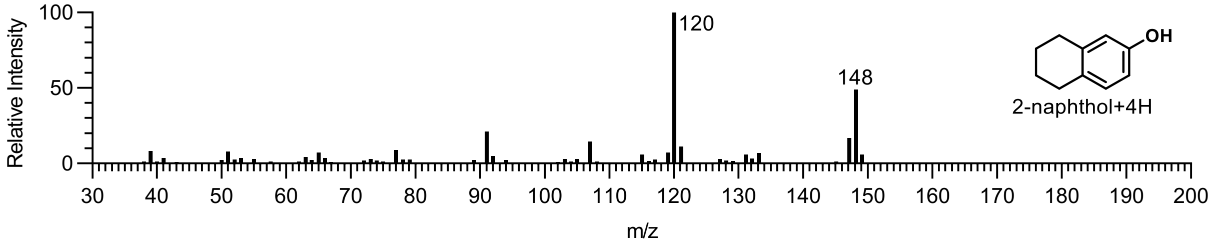

GC-MS method B

Chromatogram S60. Chromatogram of Table S14, entry 2.

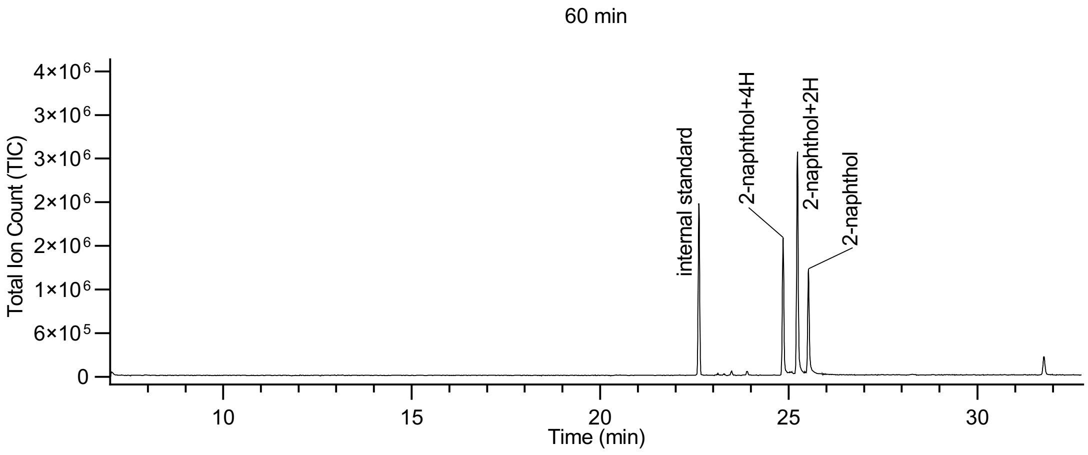

GC-MS method B

Chromatogram S61. Chromatogram of Table S14, entry 3.

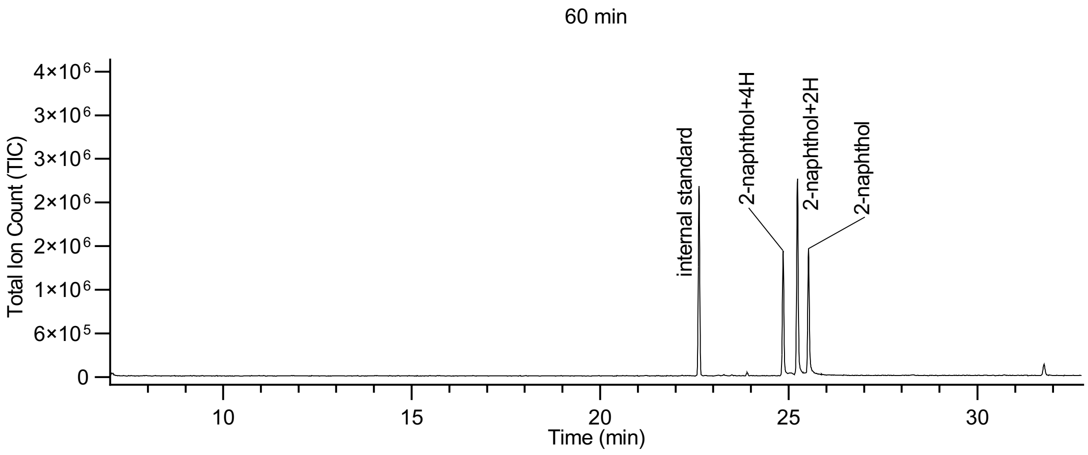

GC-MS method B

Chromatogram S62. Chromatogram of Table S15, entry 1.

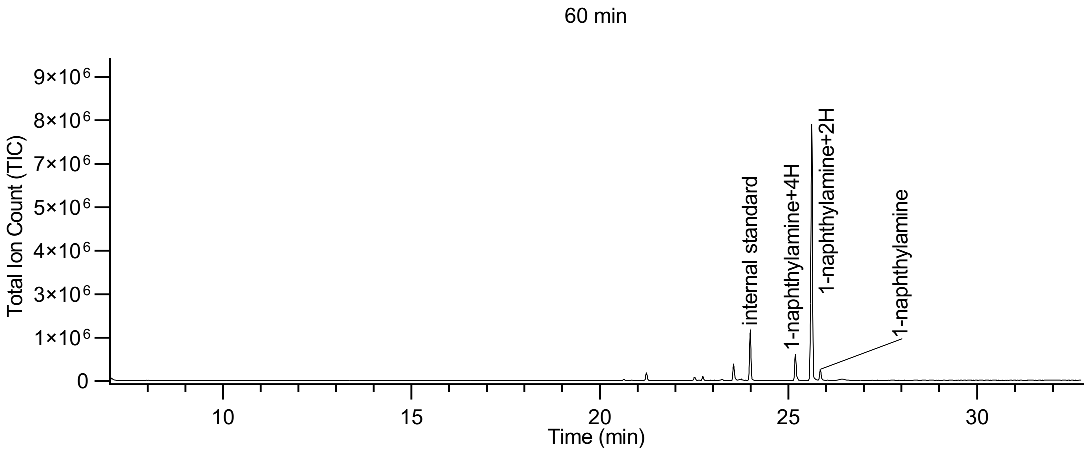

Spectrum S19. Mass spectra of 1-aminonaphthalene reduction products from Chromatogram S62 (Table S15, entry 1).

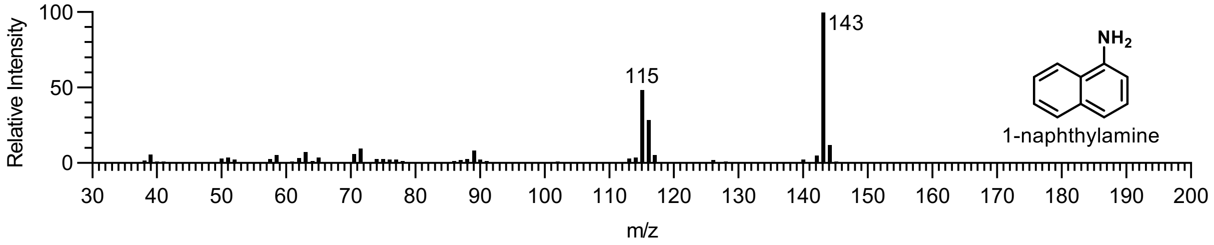

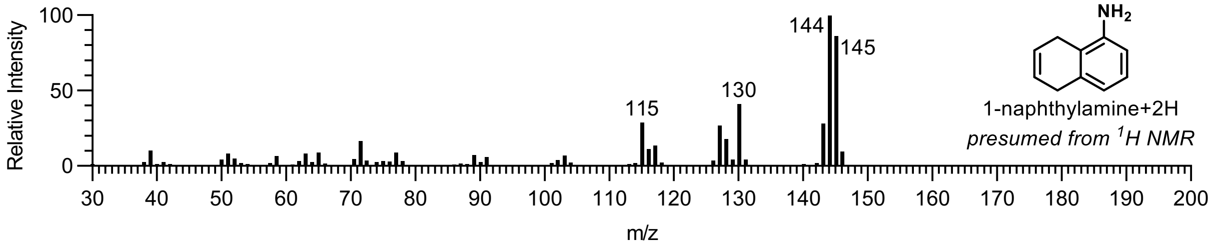

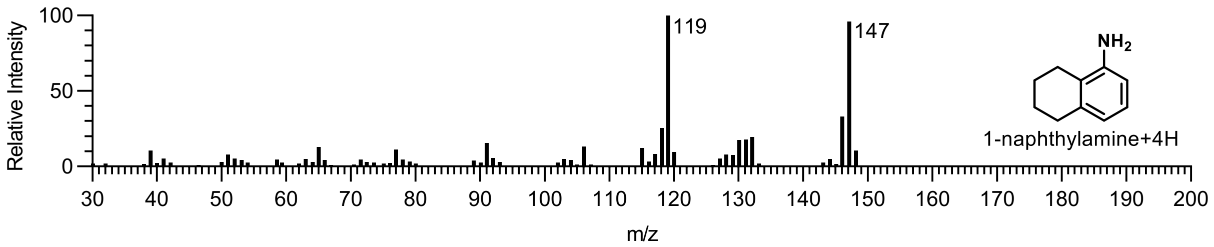

GC-MS method B

Chromatogram S63. Chromatogram of Table S15, entry 2.

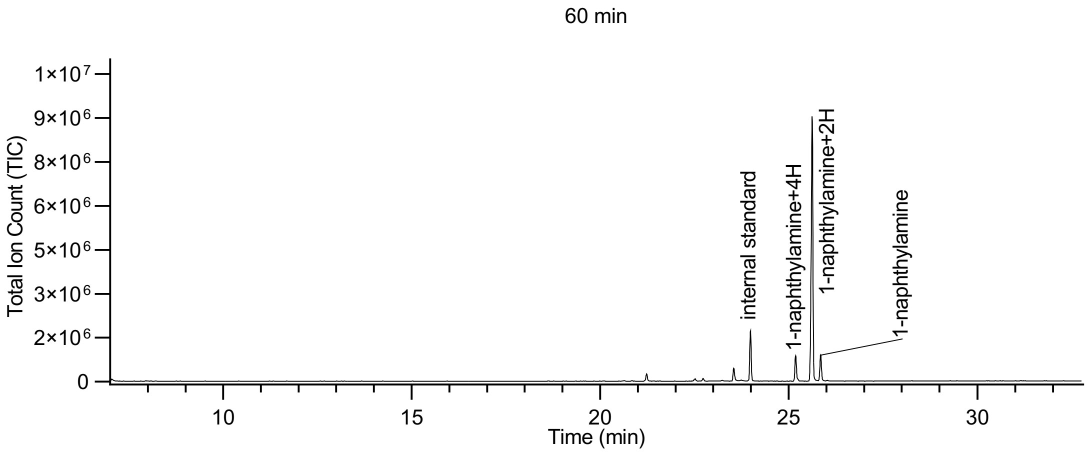

GC-MS method B

Chromatogram S64. Chromatogram of Table S15, entry 3.

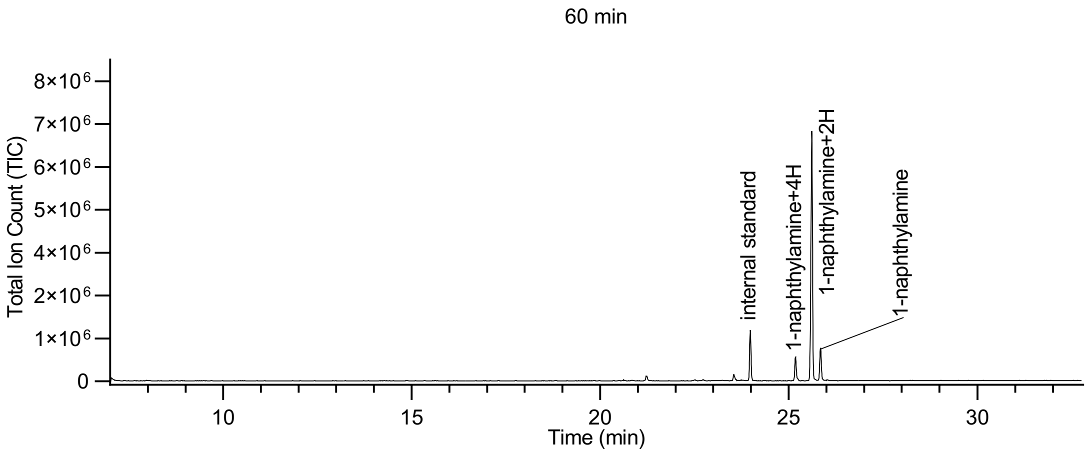

GC-MS method C

Chromatogram S65. Chromatogram of Table S17, entry 1.

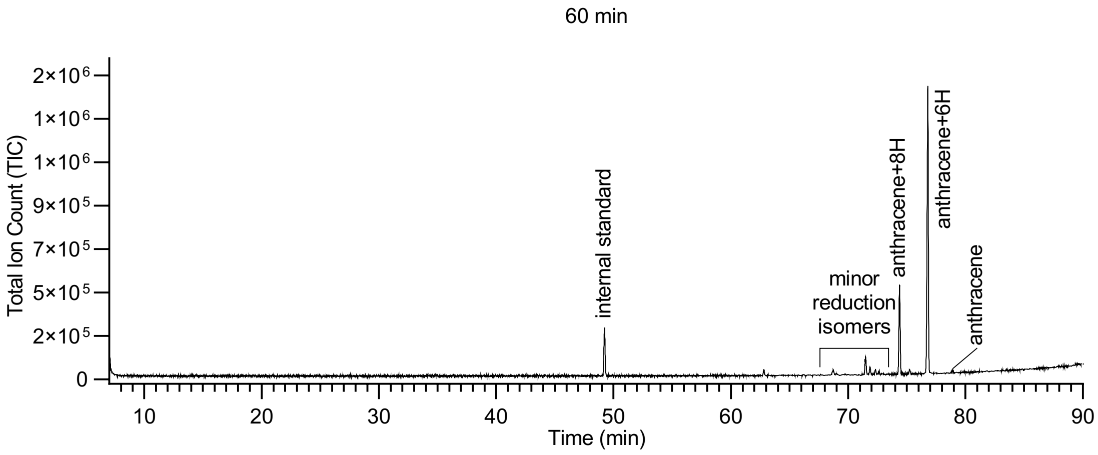

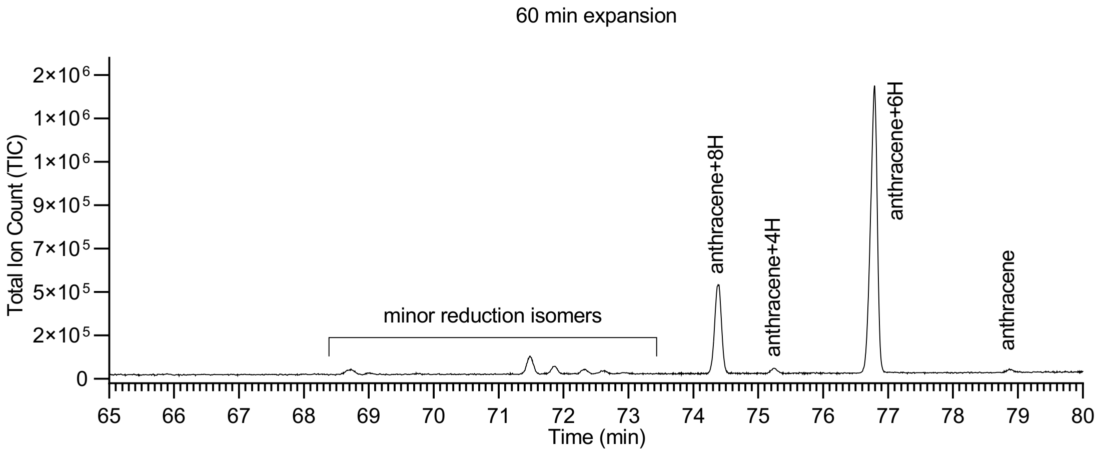

Spectrum S20. Mass spectra of anthracene reduction products from Chromatogram S65 (Table S17, entry 1).

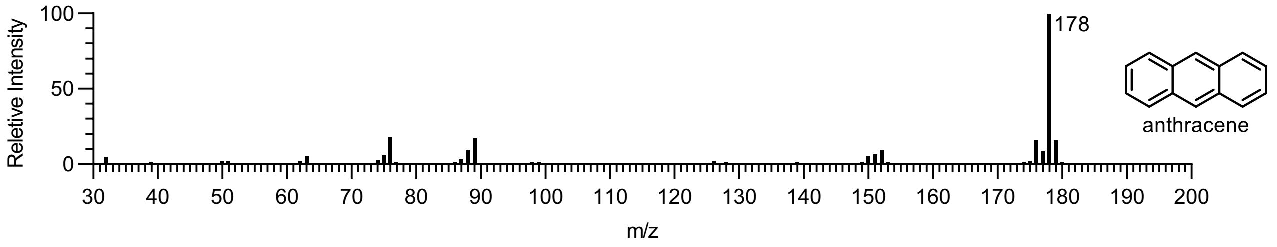

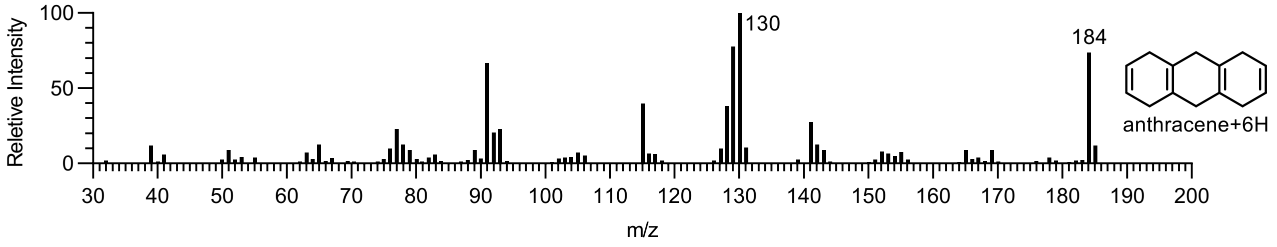

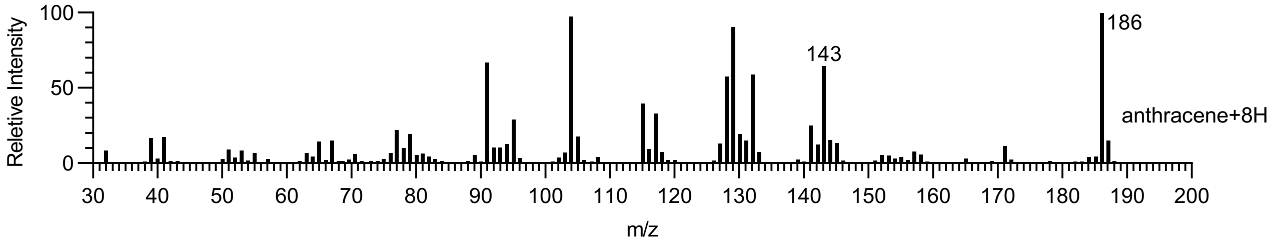

GC-MS method C

Chromatogram S66. Chromatogram of Table S17, entry 2.

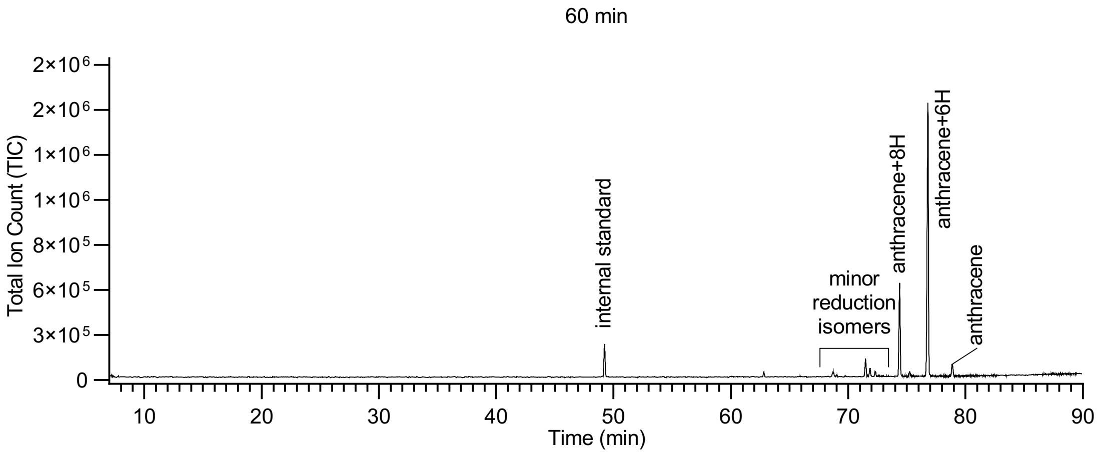

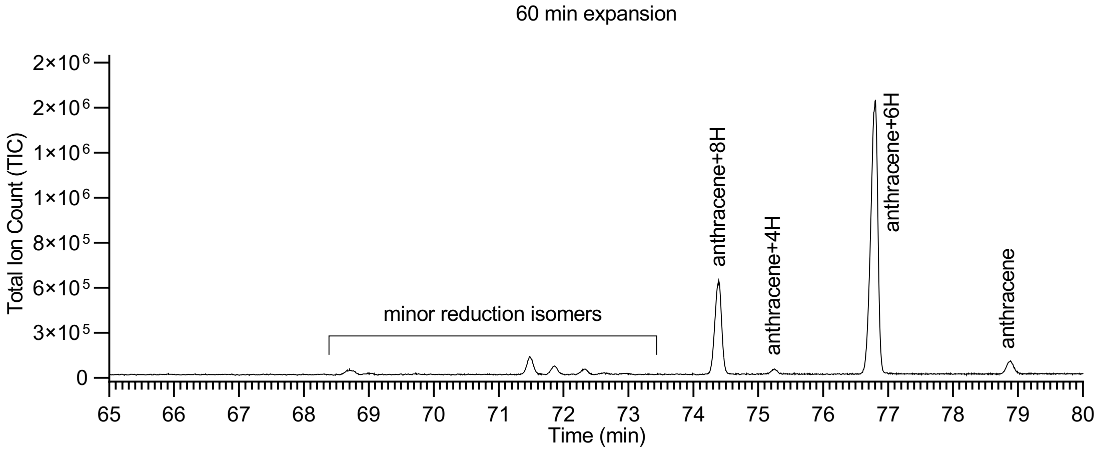

GC-MS method C

Chromatogram S67. Chromatogram of Table S17, entry 3.

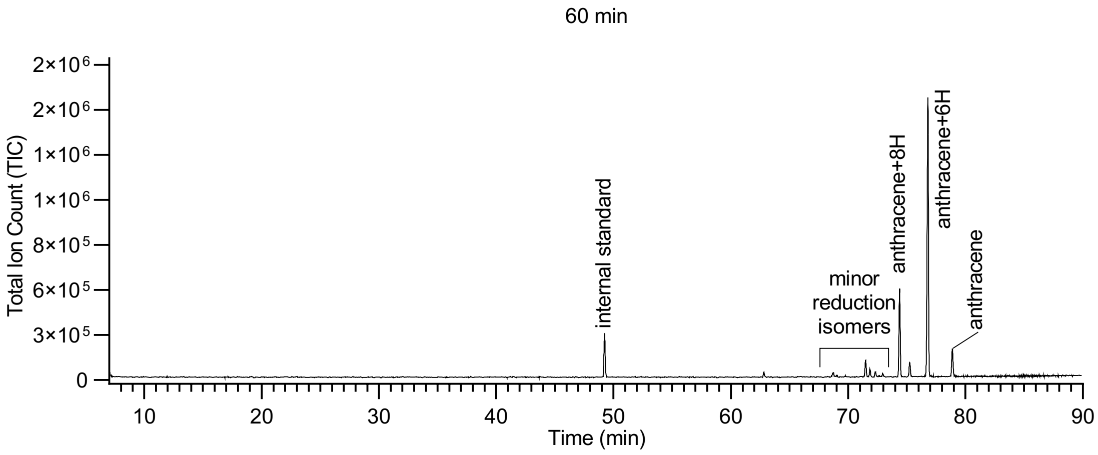

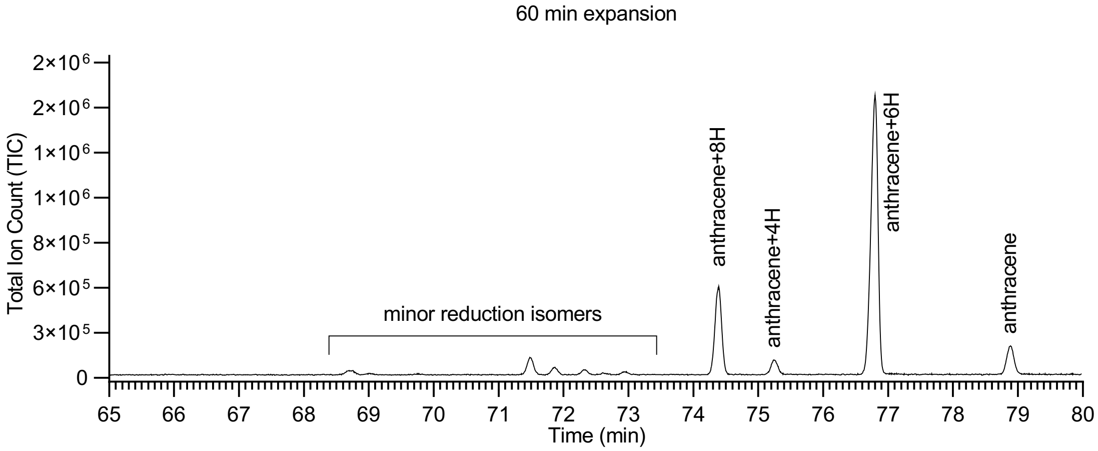

GC-MS method B

Chromatogram S68. Chromatogram of Table S18, entry 1.

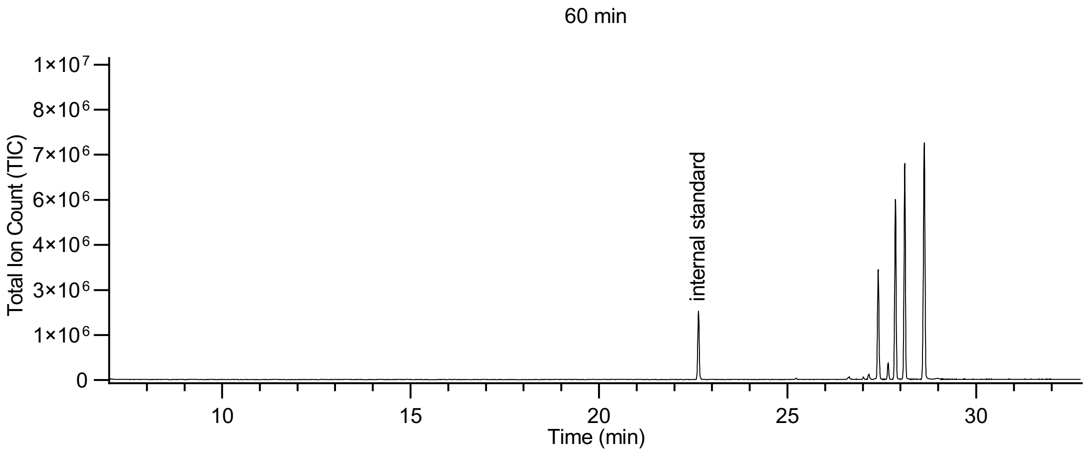

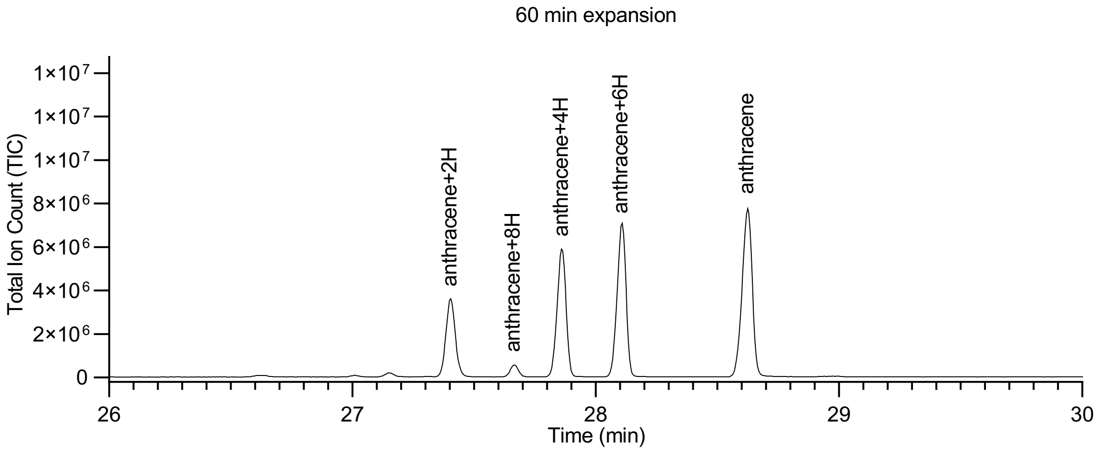

Spectrum S21. Mass spectra of anthracene reduction products from Chromatogram S68 (Table S18, entry 1).

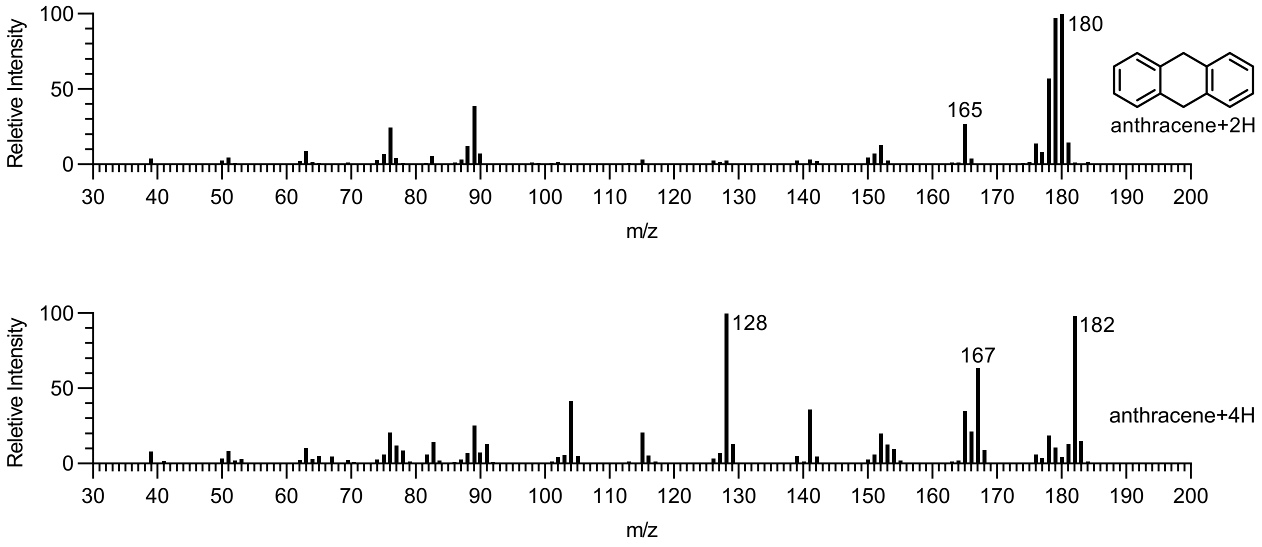

See Spectrum S20 for previously shown mass spectra of anthracene reduction isomers.

GC-MS method B

Chromatogram S69. Chromatogram of Table S18, entry 2.

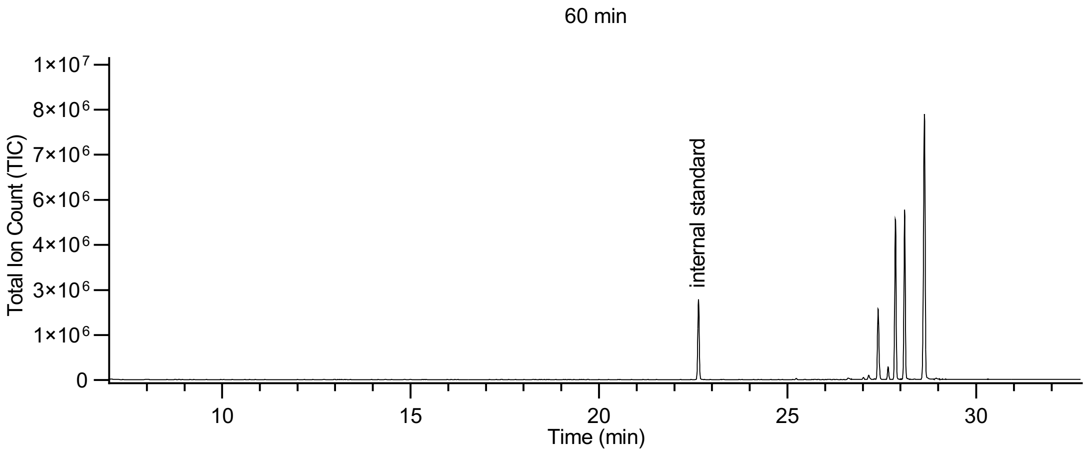

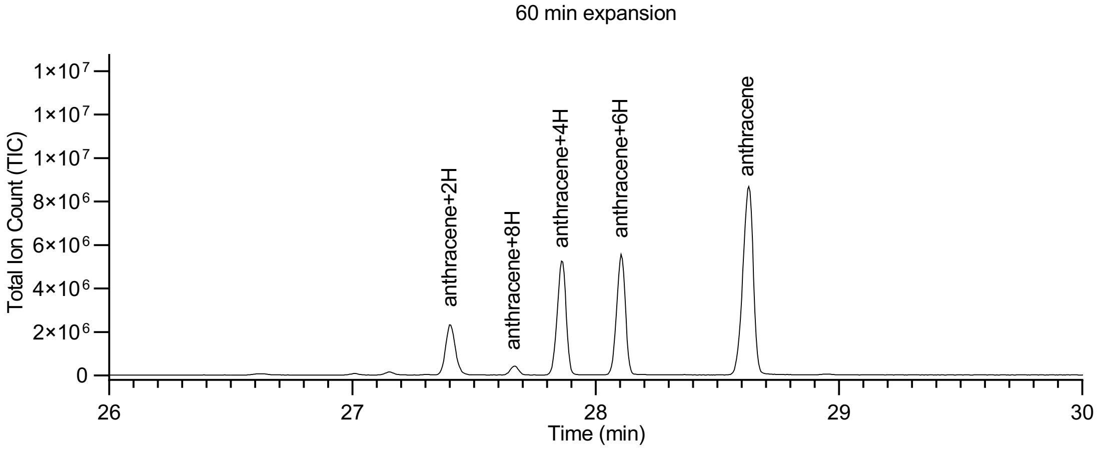

GC-MS method B

Chromatogram S70. Chromatogram of Table S18, entry 3.

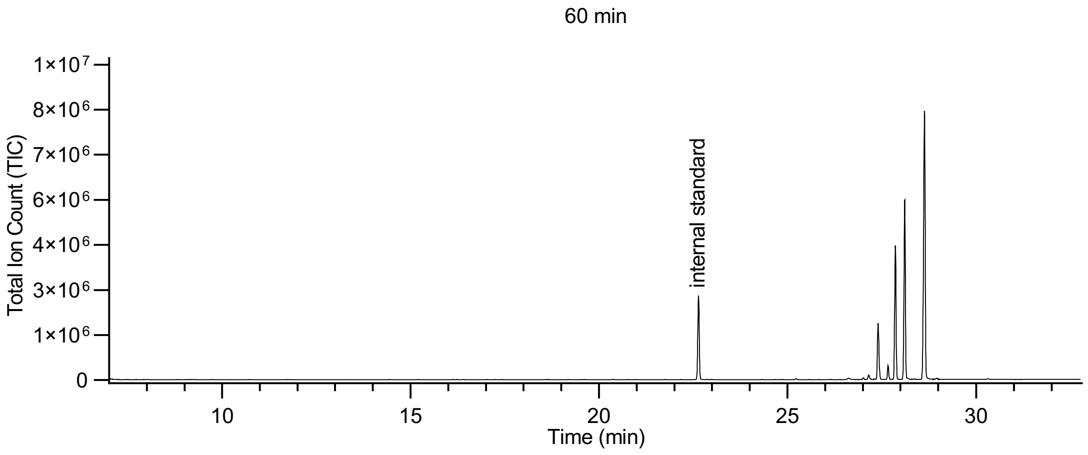

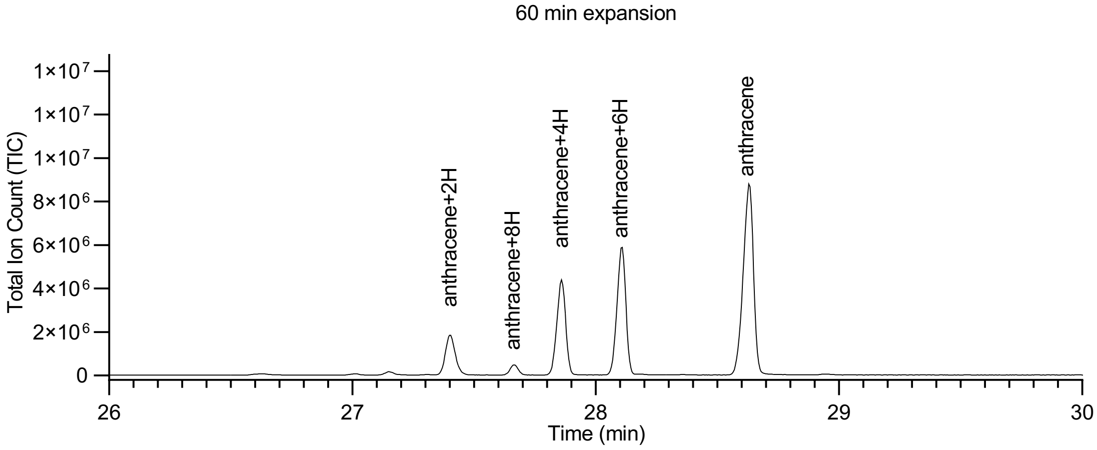

GC-MS method B

Chromatogram S71. Chromatogram of Table S19, entry 1.

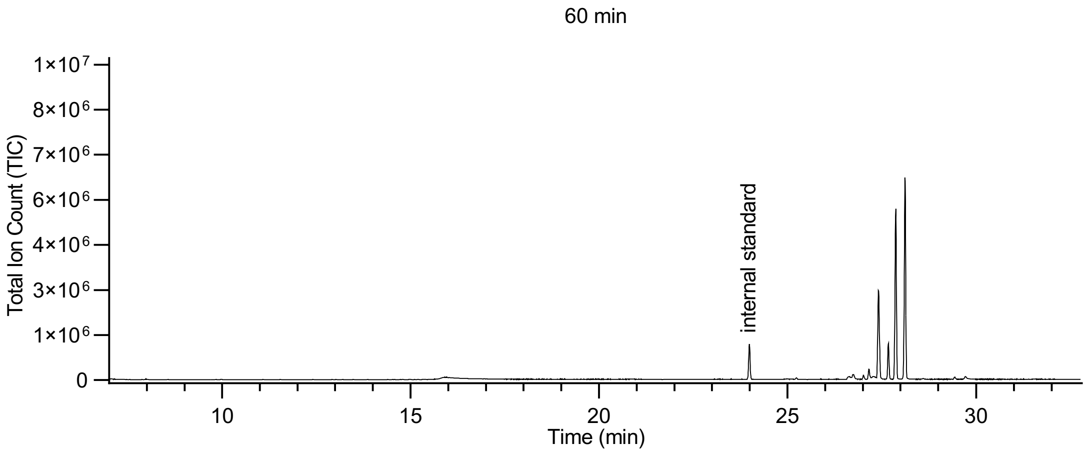

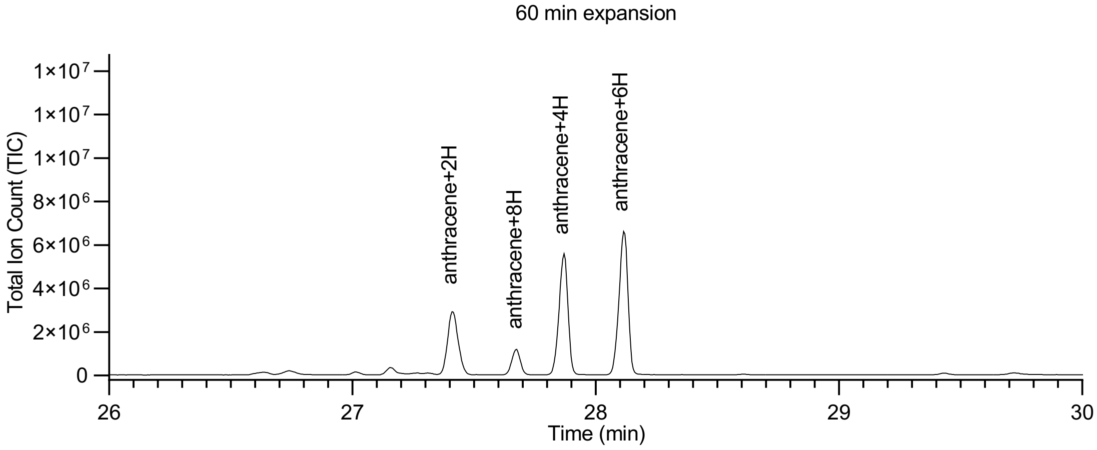

GC-MS method B

Chromatogram S72. Chromatogram of Table S19, entry 2.

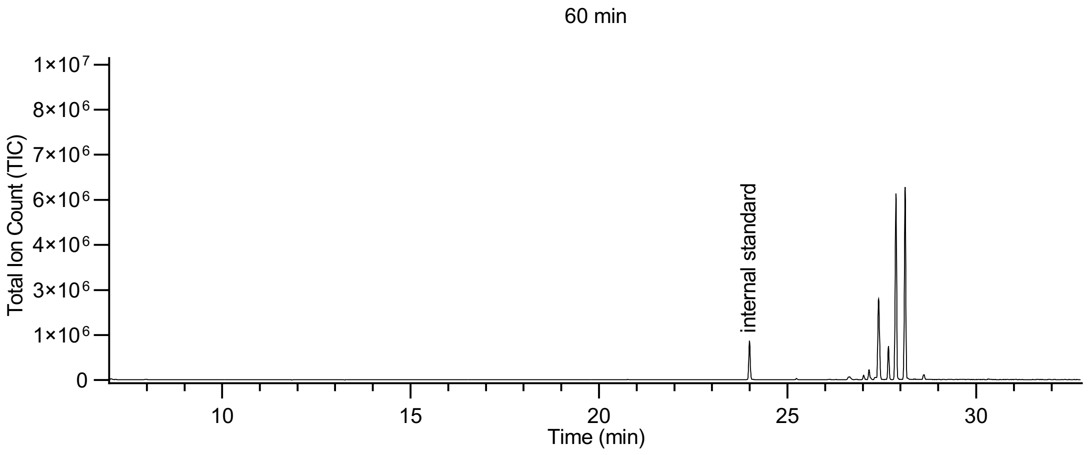

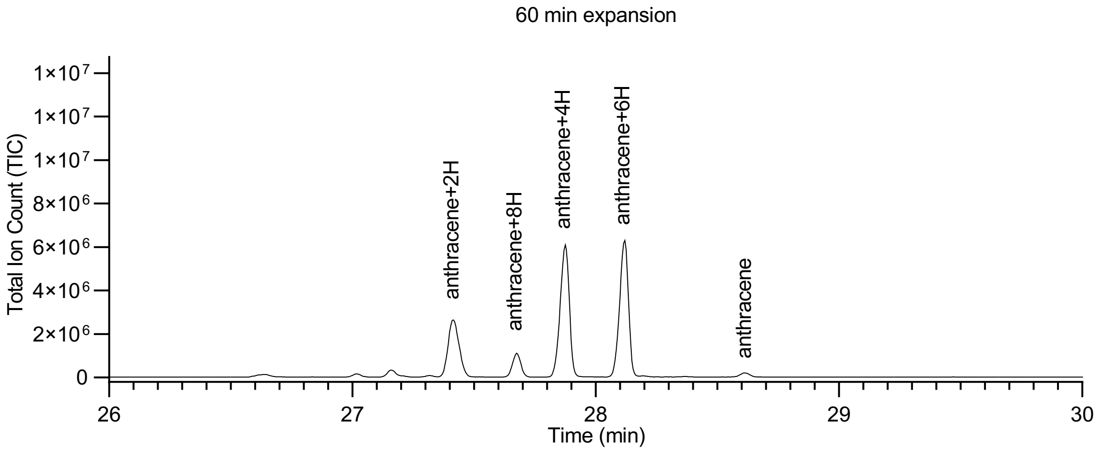

GC-MS method B

Chromatogram S73. Chromatogram of Table S19, entry 3.

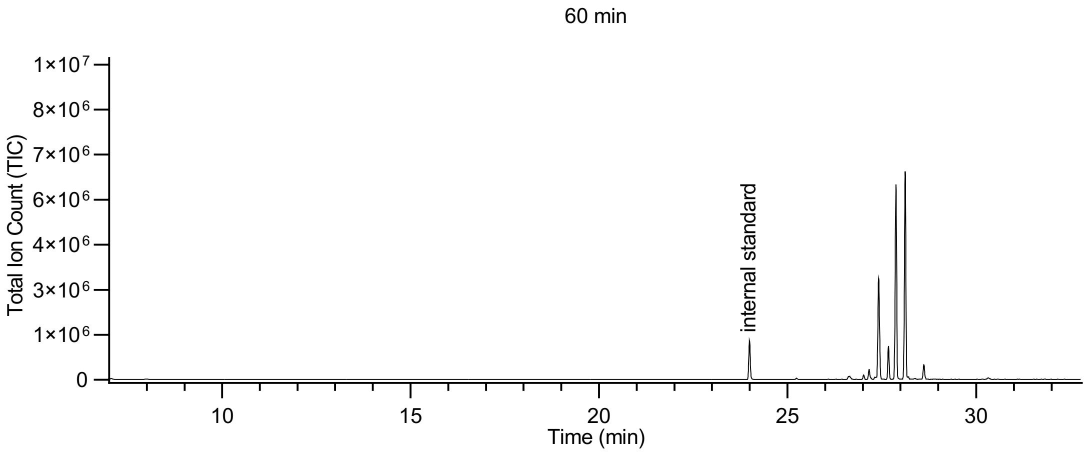

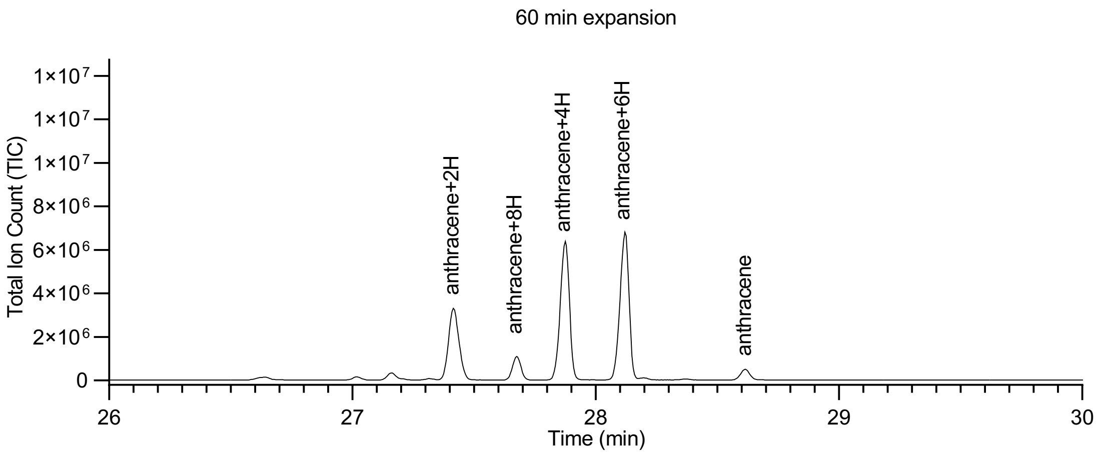

GC-MS method B

Chromatogram S74. Chromatogram of Table S20, entry 1.

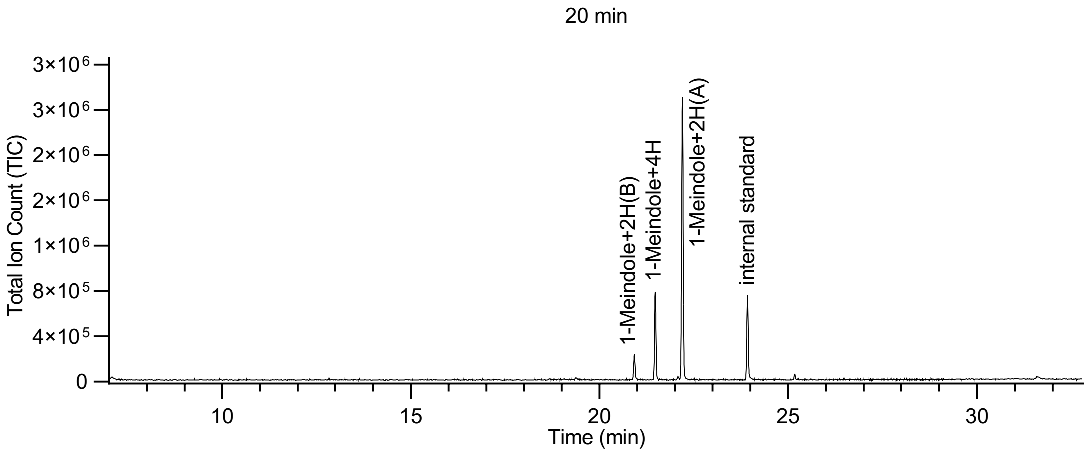

Spectrum S22. Mass spectra of 1-methylindole reduction products from Chromatogram S74 (Table S20, entry 1).

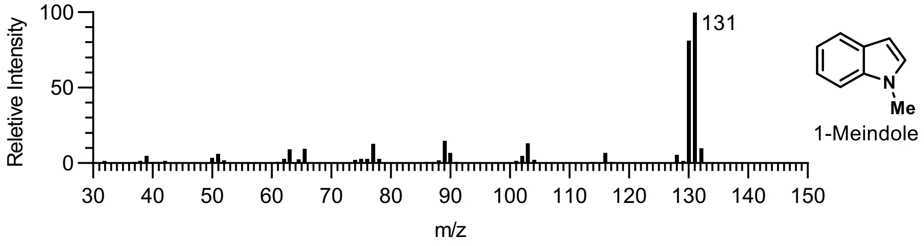

1-Meindole

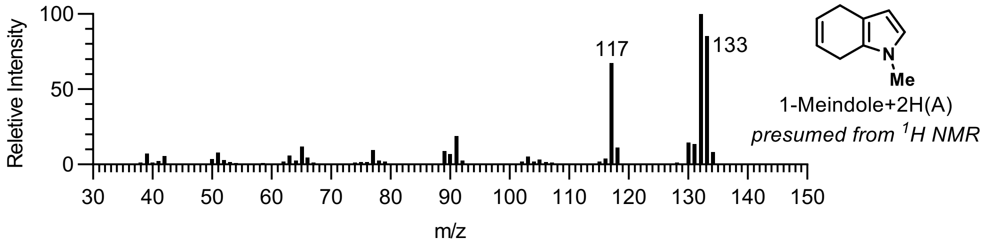

1-Meindole+2H(A)
presumed from ^{1}*H NMR*

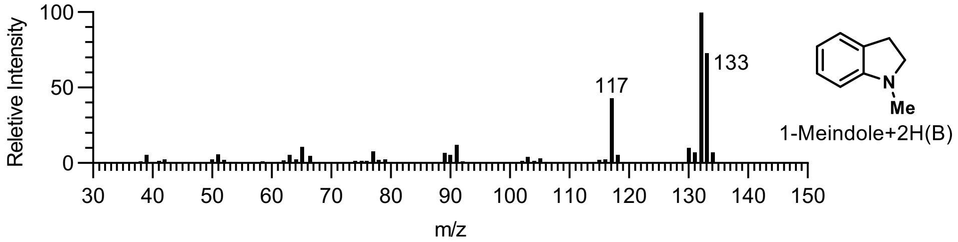

1-Meindole+2H(B)

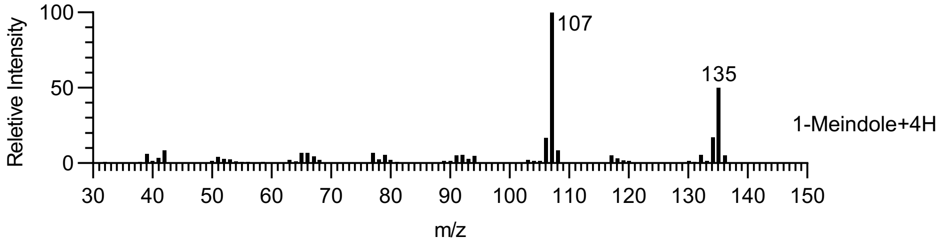

1-Meindole+4H

GC-MS method B

Chromatogram S75. Chromatogram of Table S20, entry 2.

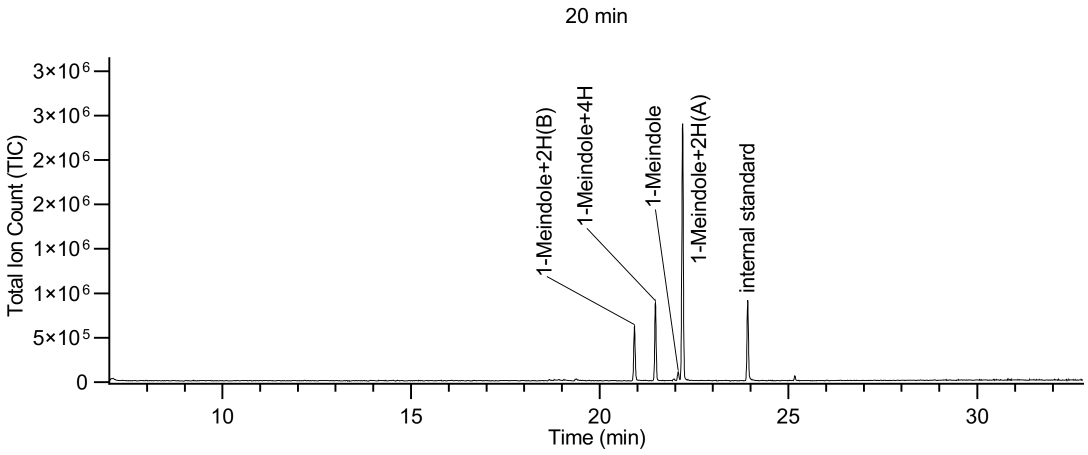

GC-MS method B

Chromatogram S76. Chromatogram of Table S20, entry 3.

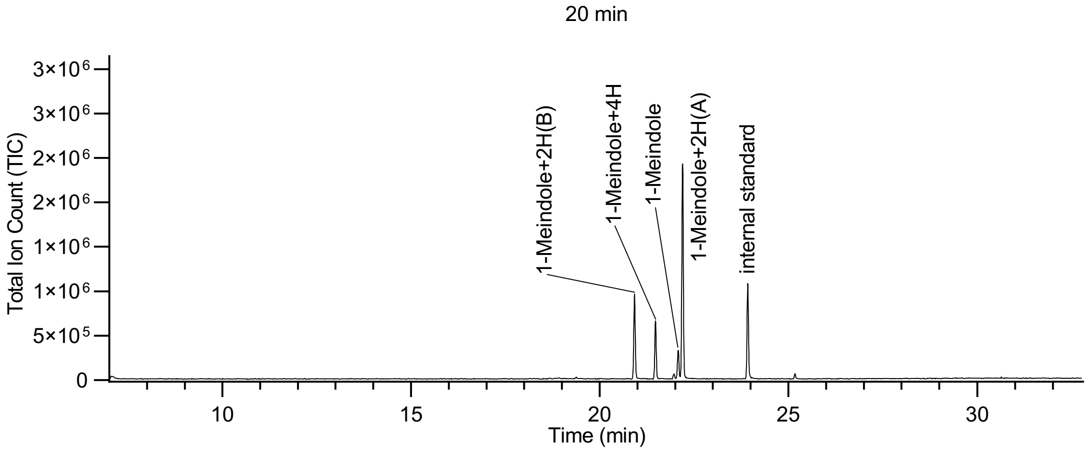

GC-MS method B

Chromatogram S77. Chromatogram of Table S21, entry 1.

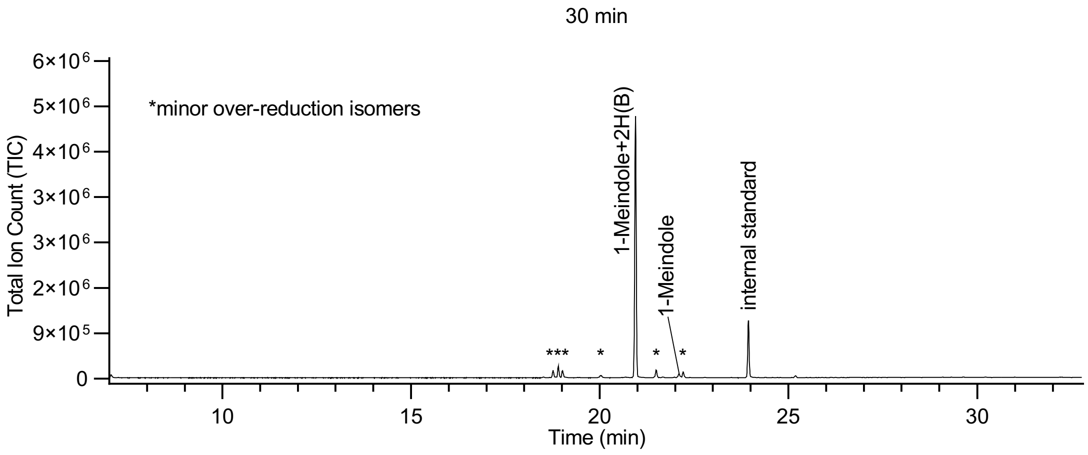

GC-MS method B

Chromatogram S78. Chromatogram of Table S21, entry 2.

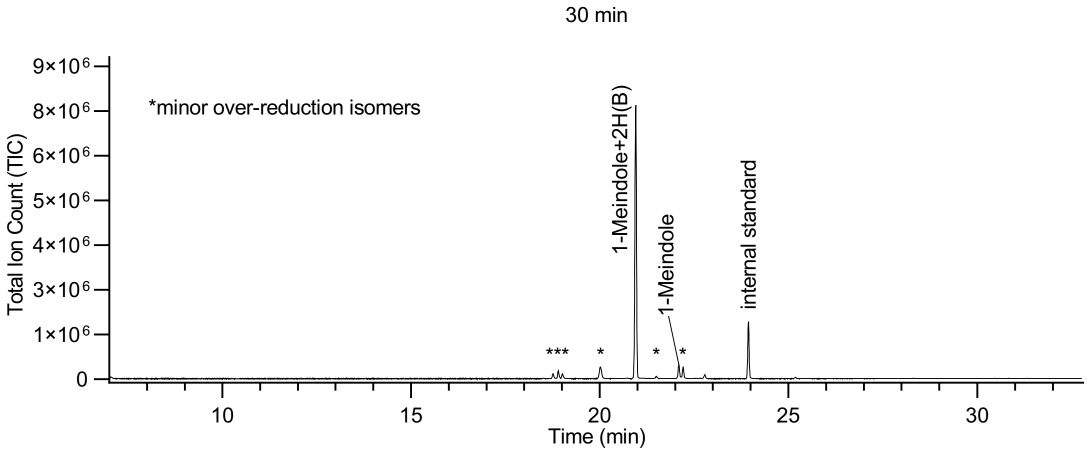

GC-MS method B

Chromatogram S79. Chromatogram of Table S21, entry 3.

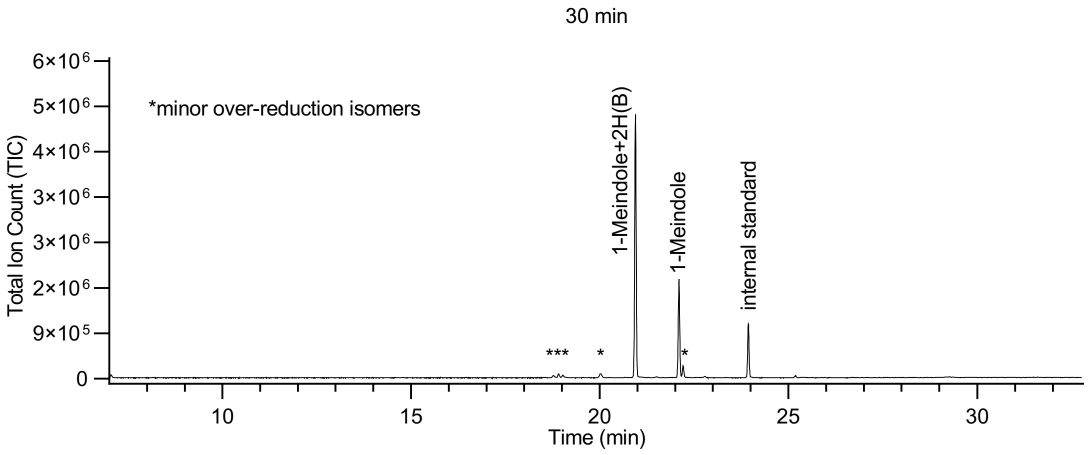

GC-MS method B

Chromatogram S80. Chromatogram of Table S22, entry 1.

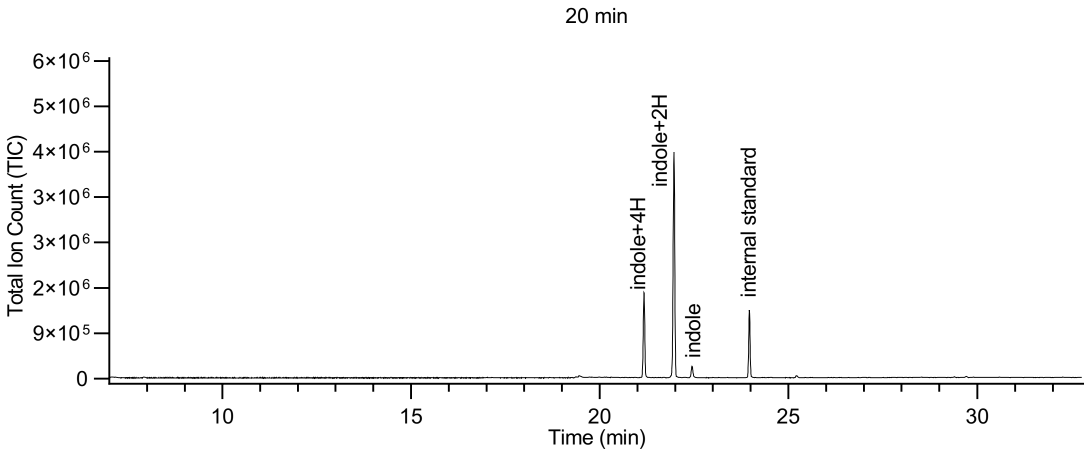

Spectrum S23. Mass spectra of indole reduction products from Chromatogram S80 (Table S22, entry 1).

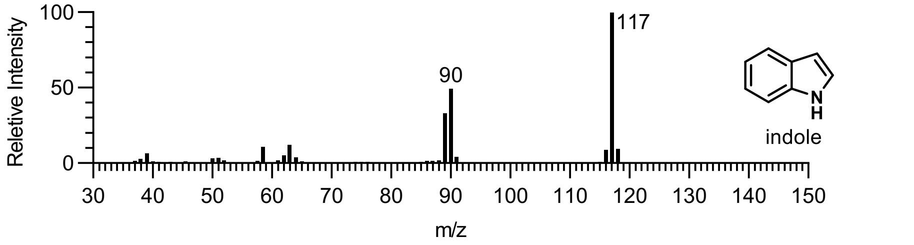

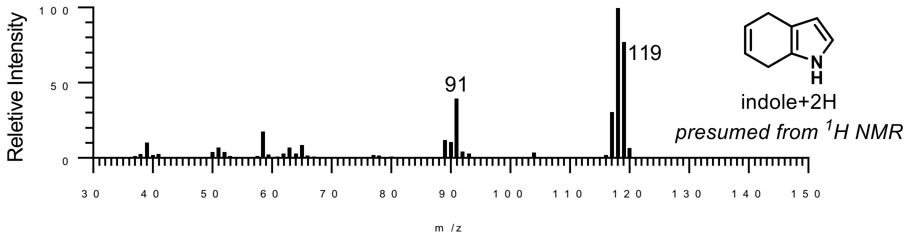

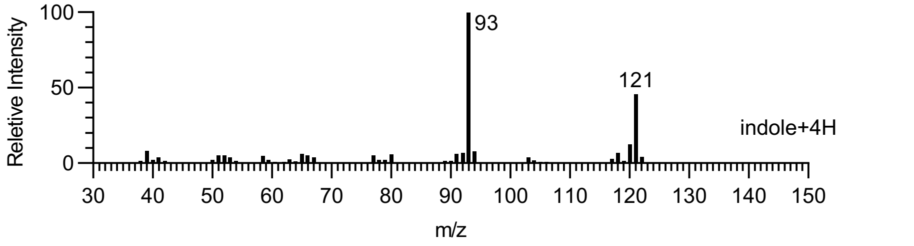

GC-MS method B

Chromatogram S81. Chromatogram of Table S22, entry 2.

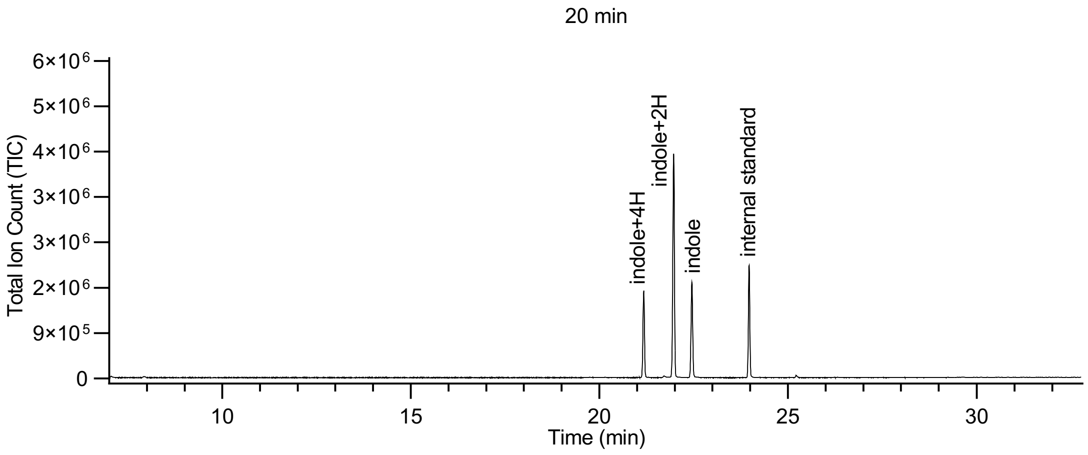

GC-MS method B

Chromatogram S82. Chromatogram of Table S22, entry 3.

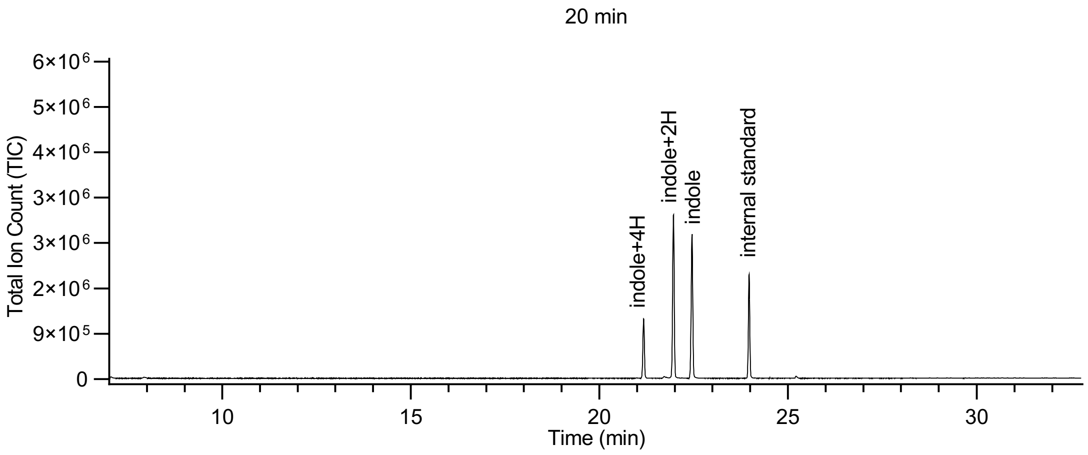

GC-MS method B

Chromatogram S83. Chromatogram of Table S23, entry 1.

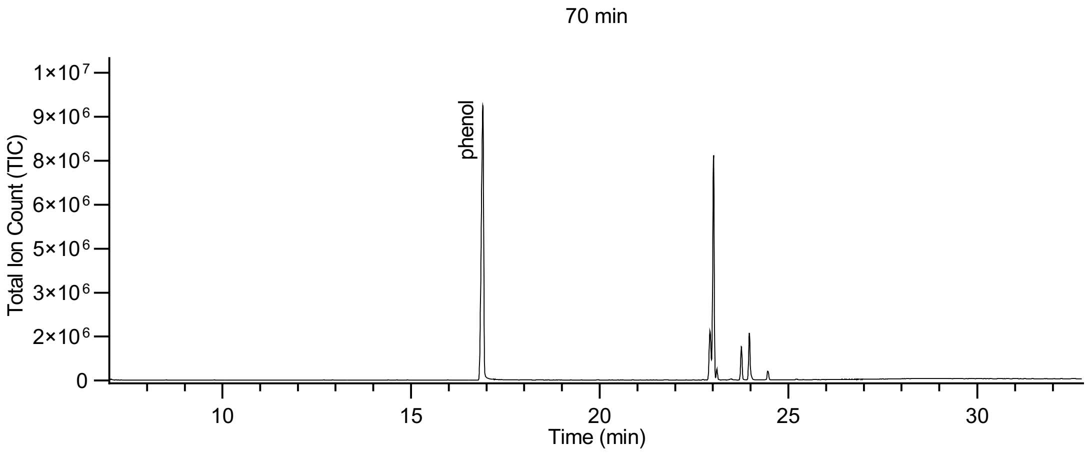

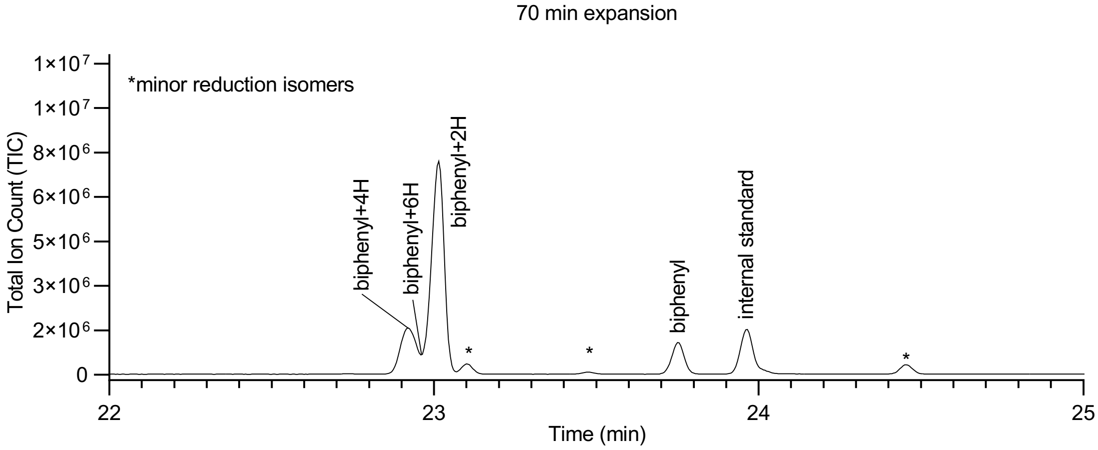

Spectrum S24. Mass spectra of biphenyl reduction products from Chromatogram S83 (Table S23, entry 1).

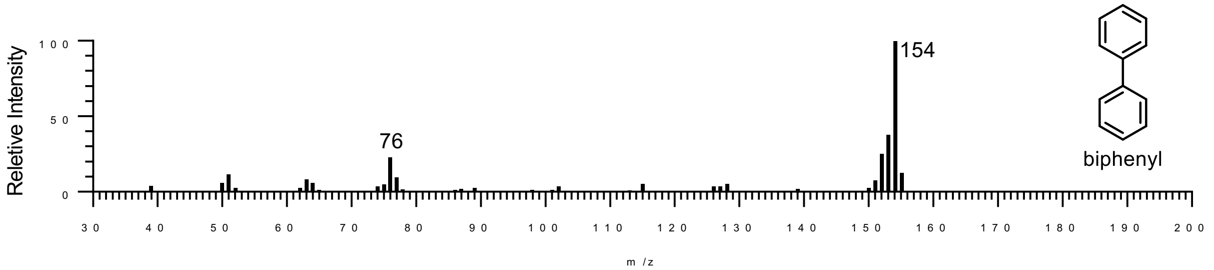

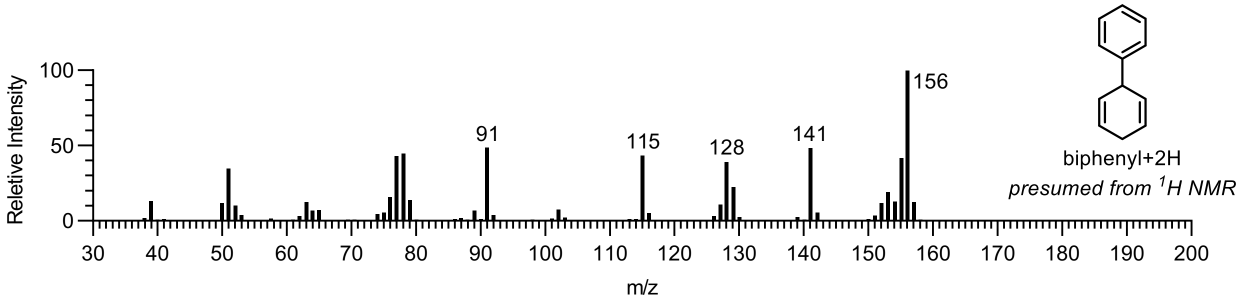

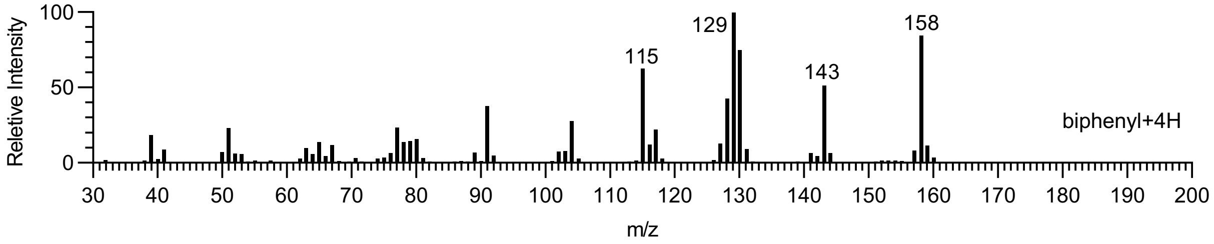

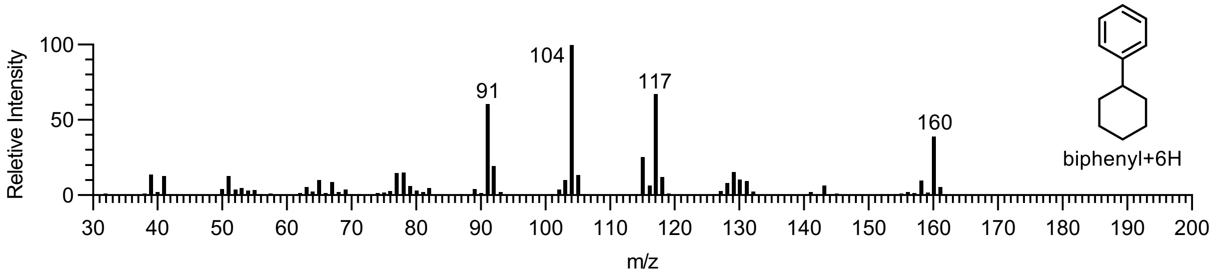

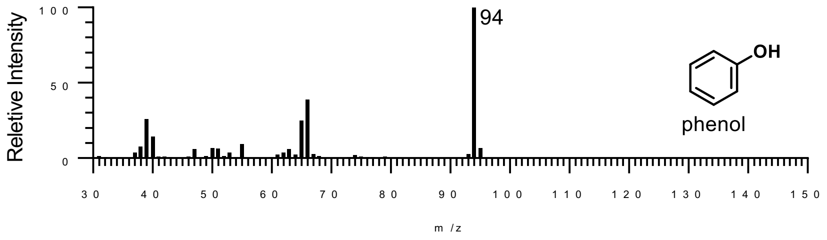

GC-MS method B

Chromatogram S84. Chromatogram of Table S23, entry 2.

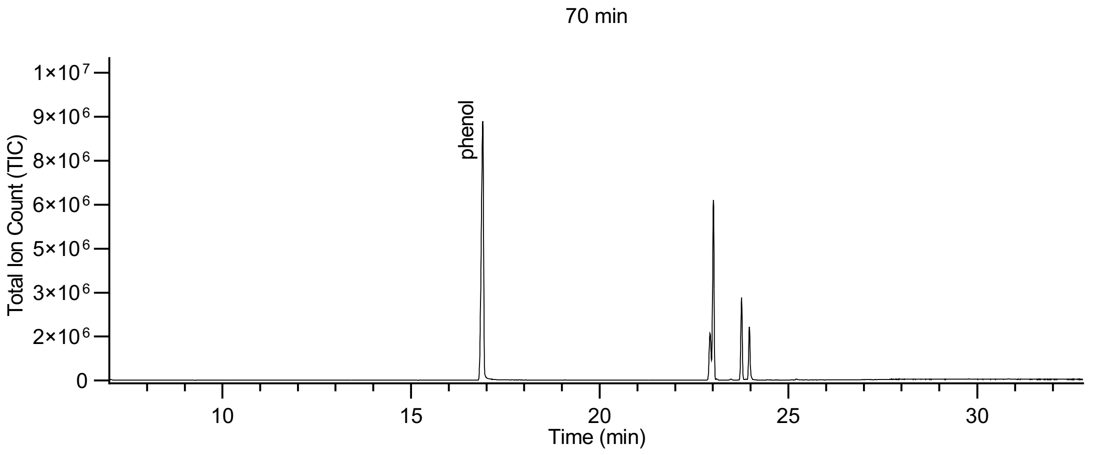

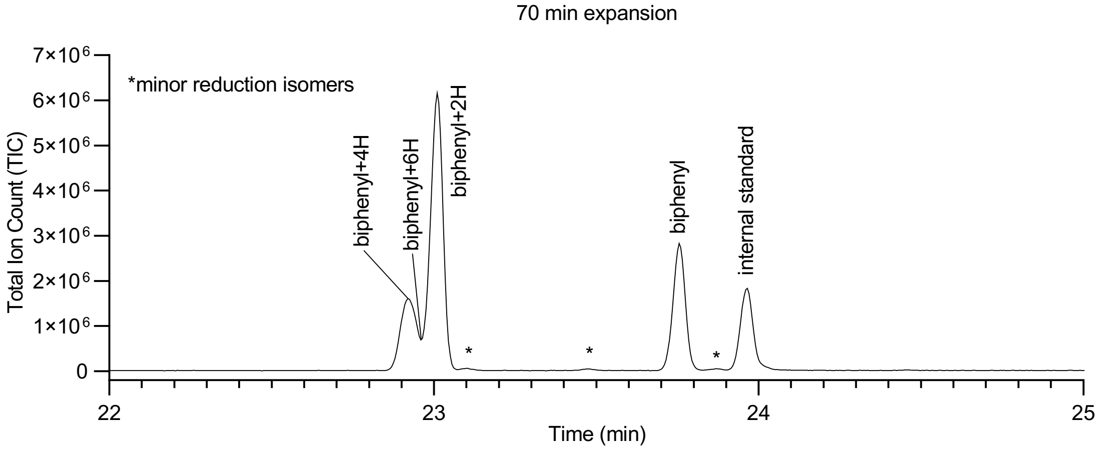

GC-MS method B

Chromatogram S85. Chromatogram of Table S23, entry 3.

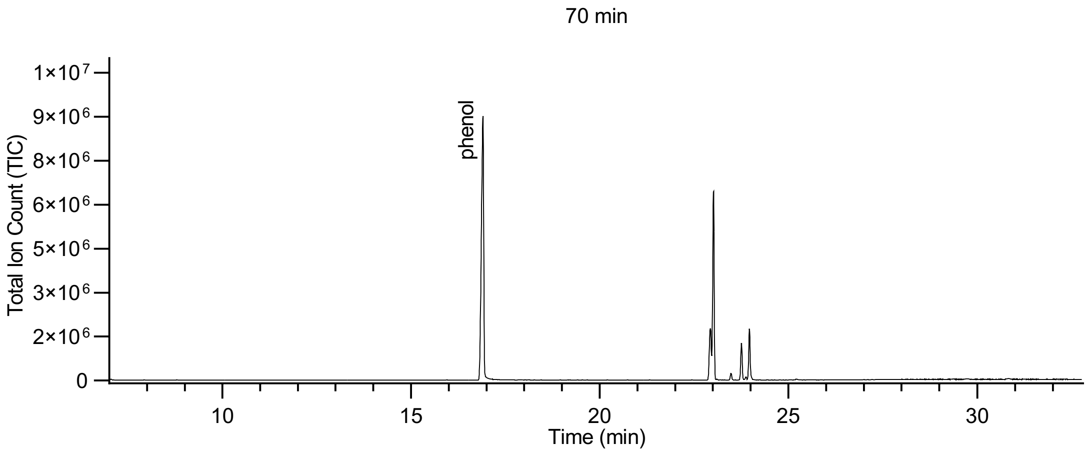

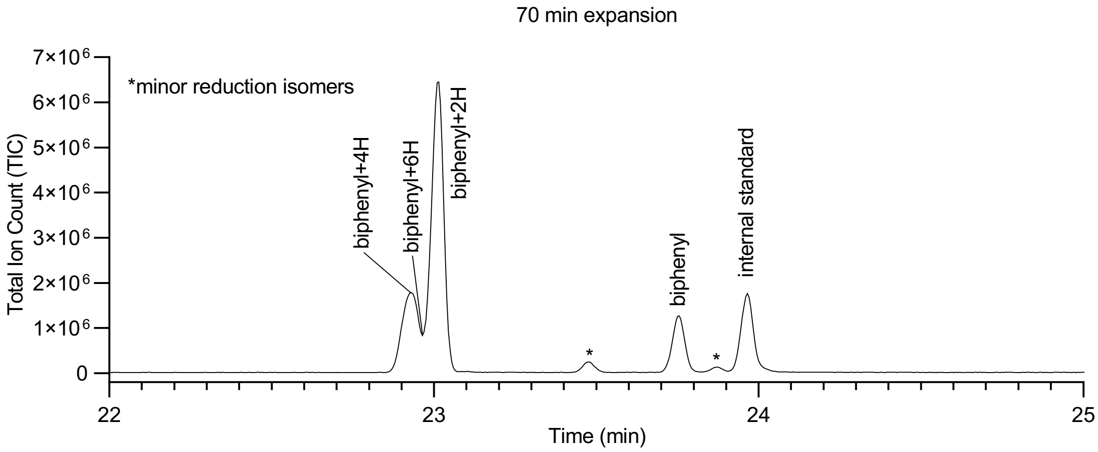

GC-MS method B

Chromatogram S86. Chromatogram of Table S24, entry 1.

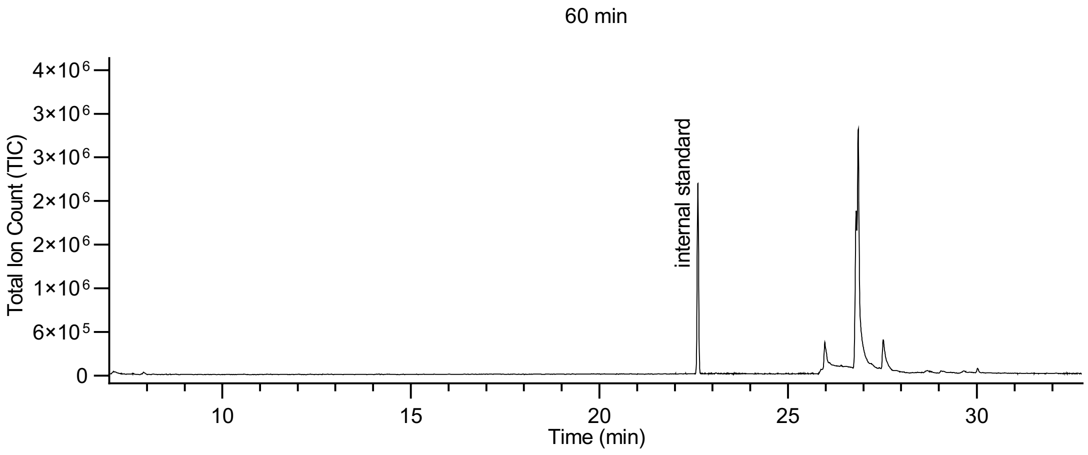

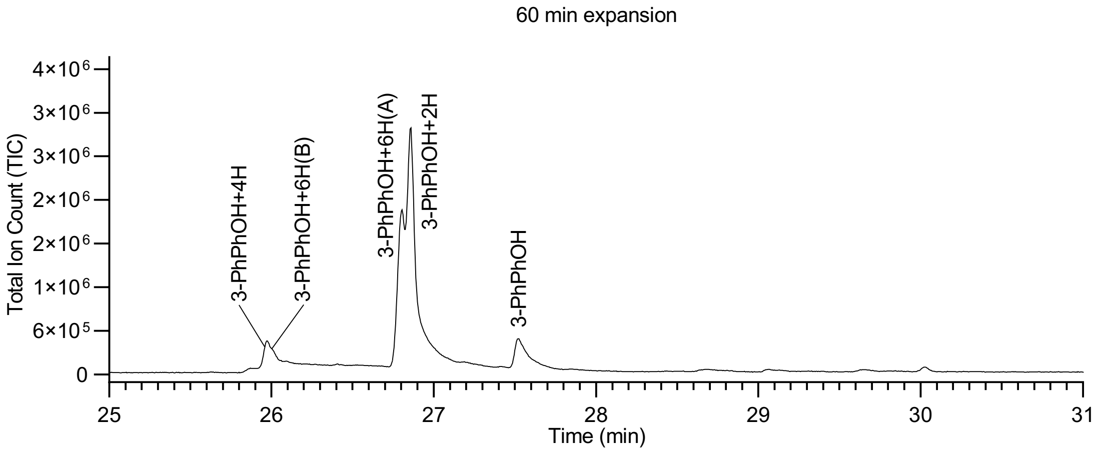

Spectrum S25. Mass spectra of 3-phenylphenol reduction products from Chromatogram S86 (Table S24, entry 1).

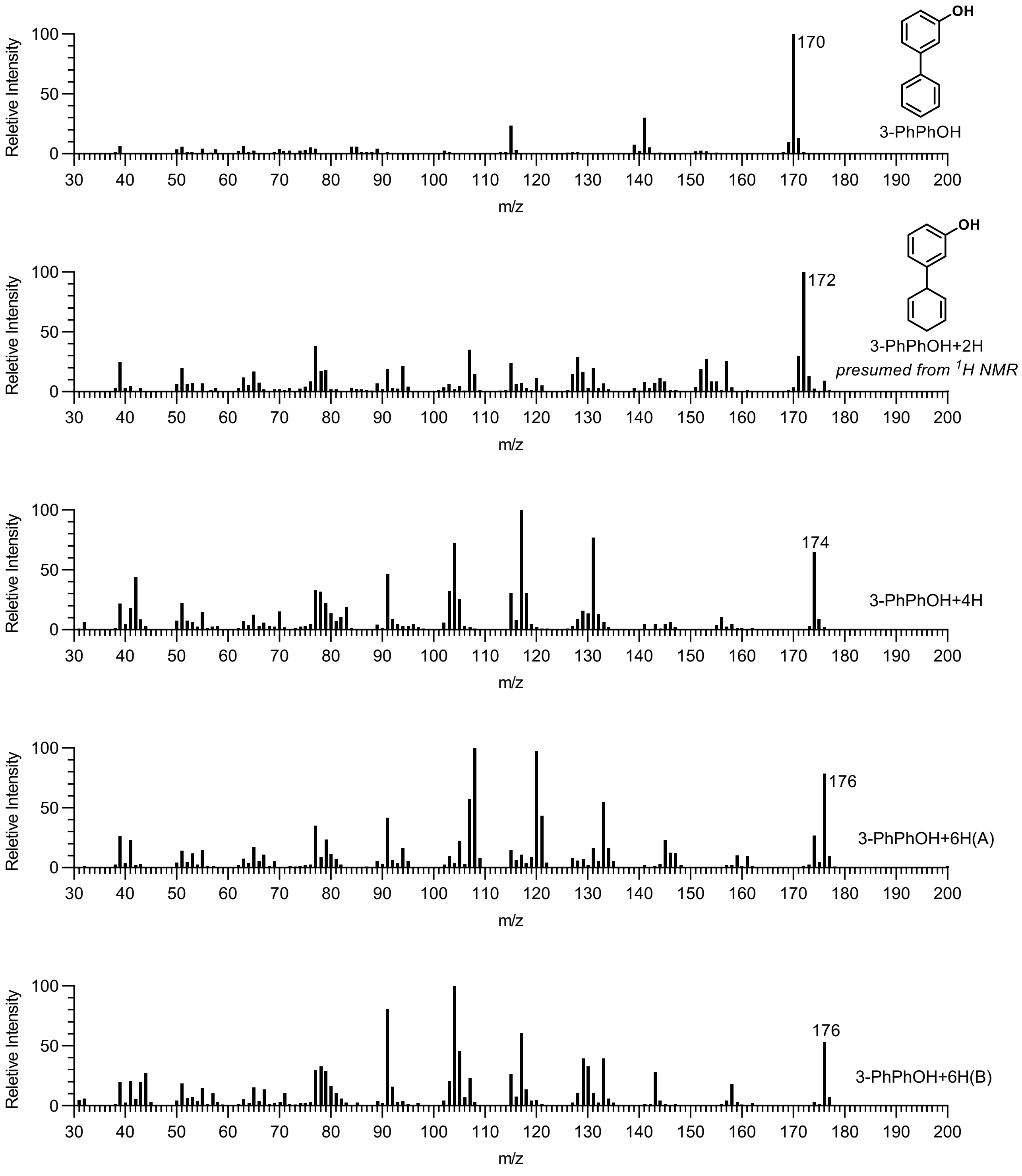

GC-MS method B

Chromatogram S87. Chromatogram of Table S24, entry 2.

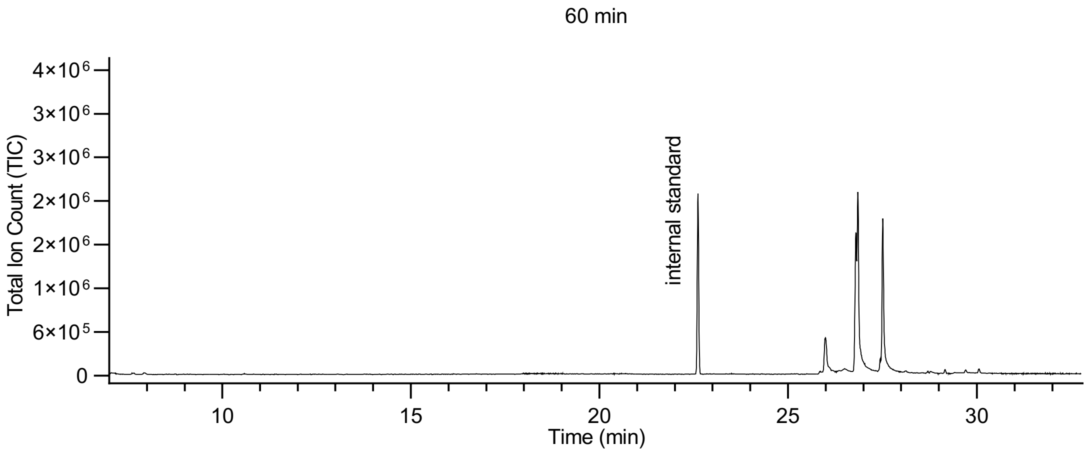

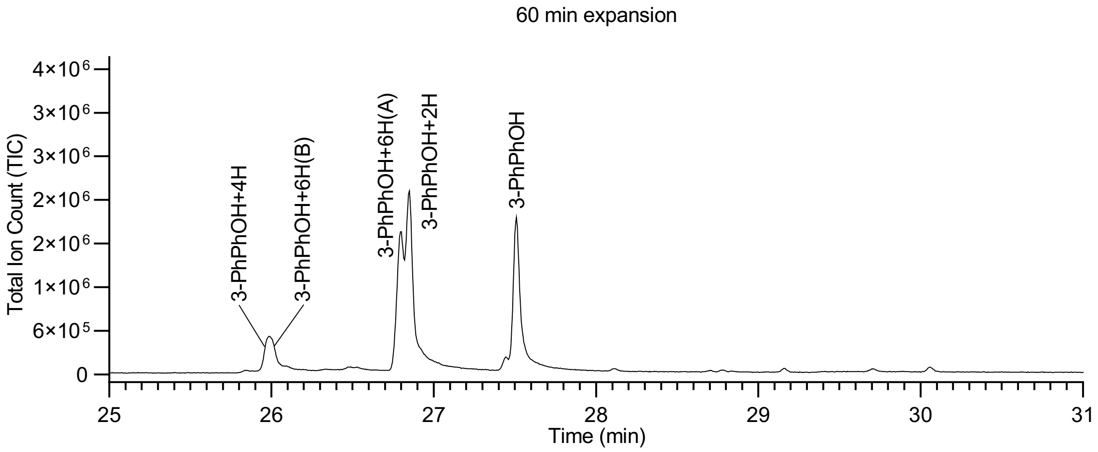

GC-MS method B

Chromatogram S88. Chromatogram of Table S24, entry 3.

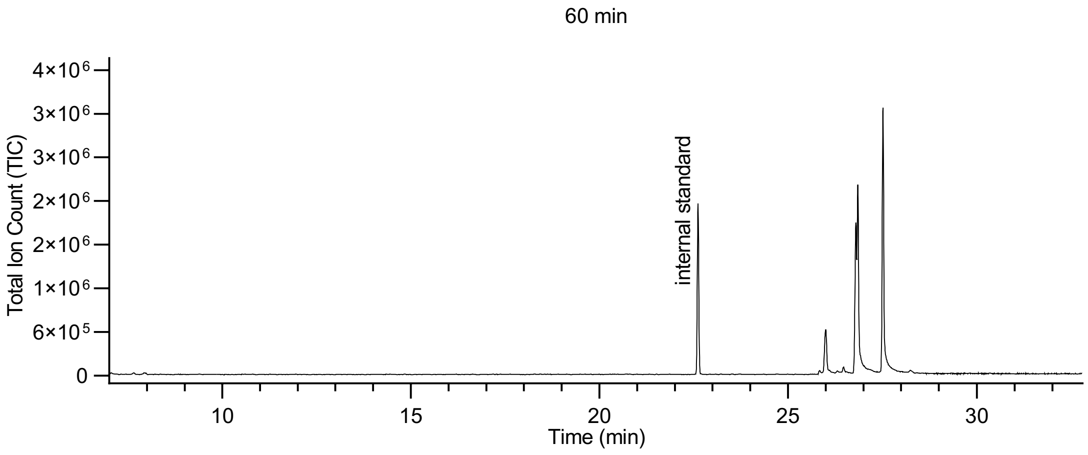

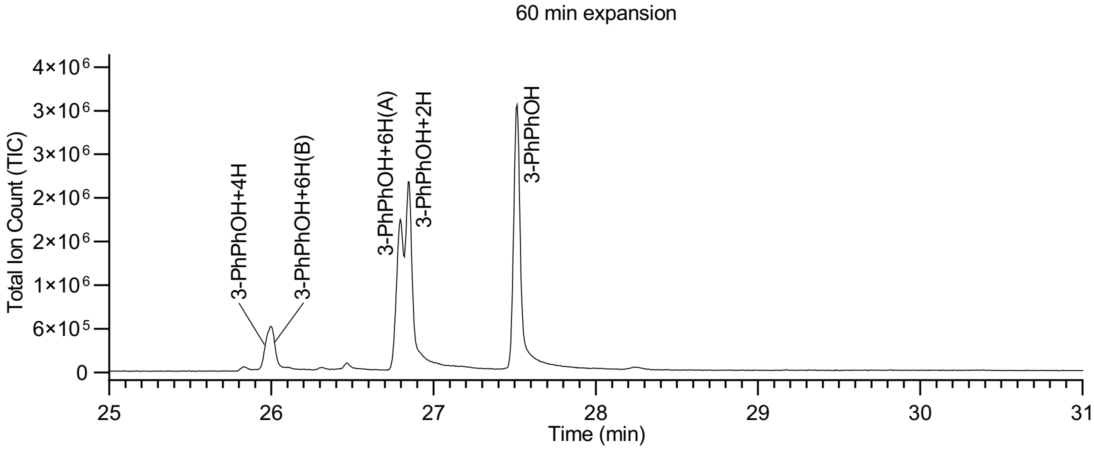

NMR Spectra

Spectrum S26. ^{1}H NMR spectrum of 1-methoxyadamantane (300 MHz, $CDCl_3$, 298 K).

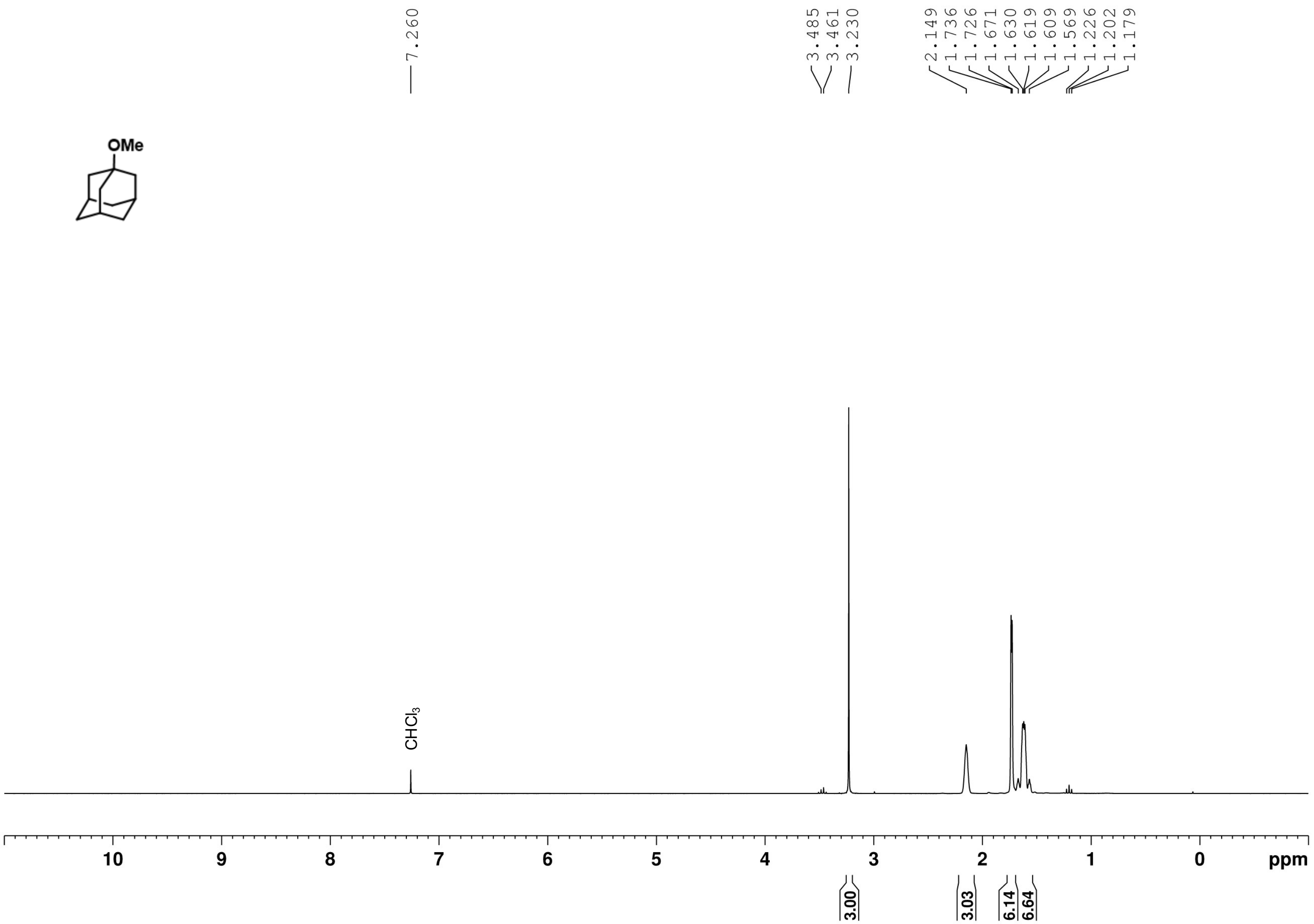

Spectrum S27. ^{1}H NMR spectrum of *t*-butyldimethyl(*p*-tolyloxy)silane (**6**) (400 MHz, $CDCl_3$, 298 K).

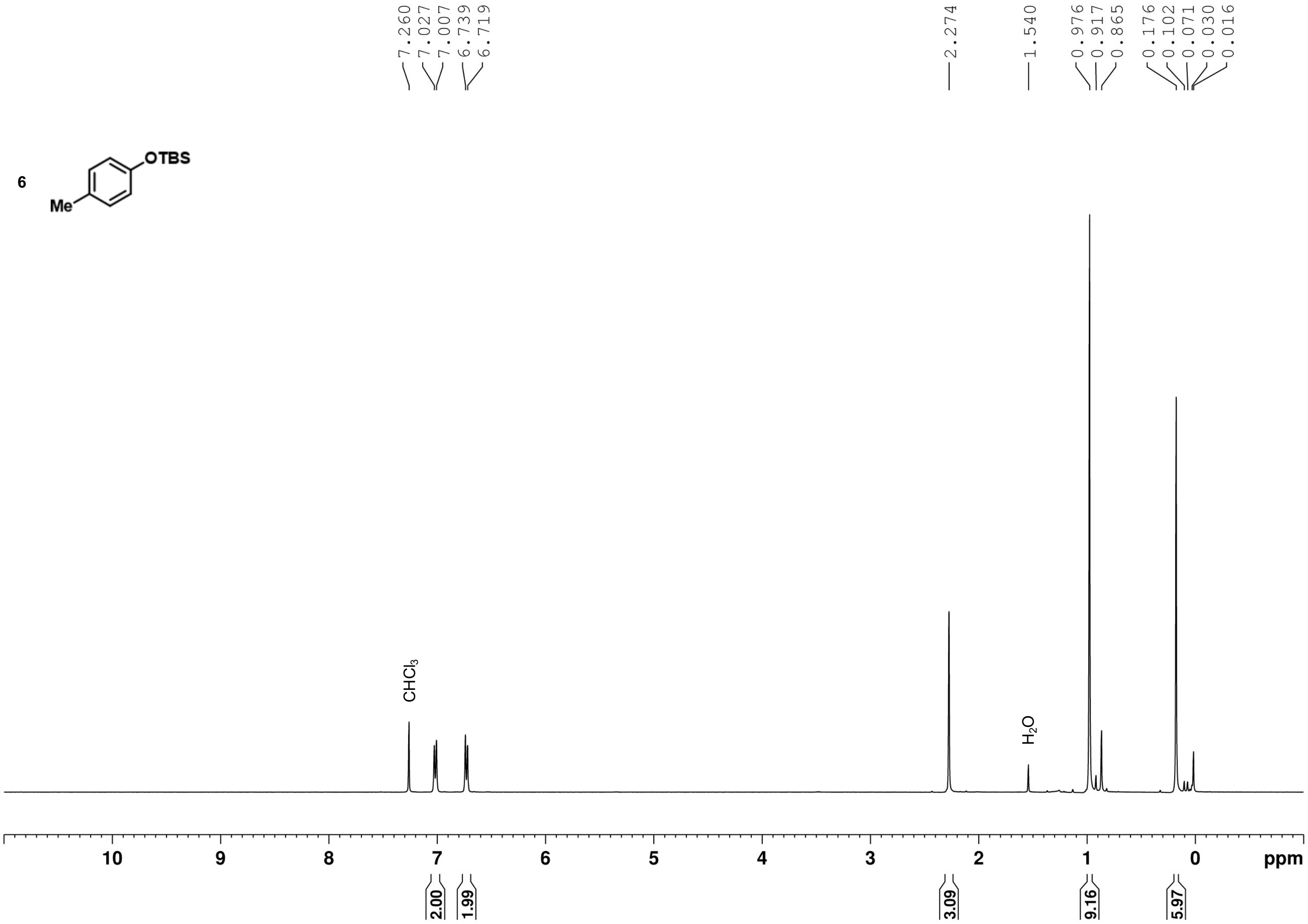

Spectrum S28. ^{1}H NMR spectrum of the reduction of naphthalene (**8**) under Ar using general method A, Table 2 entry 1 (400 MHz, $CDCl_3$, 298 K).

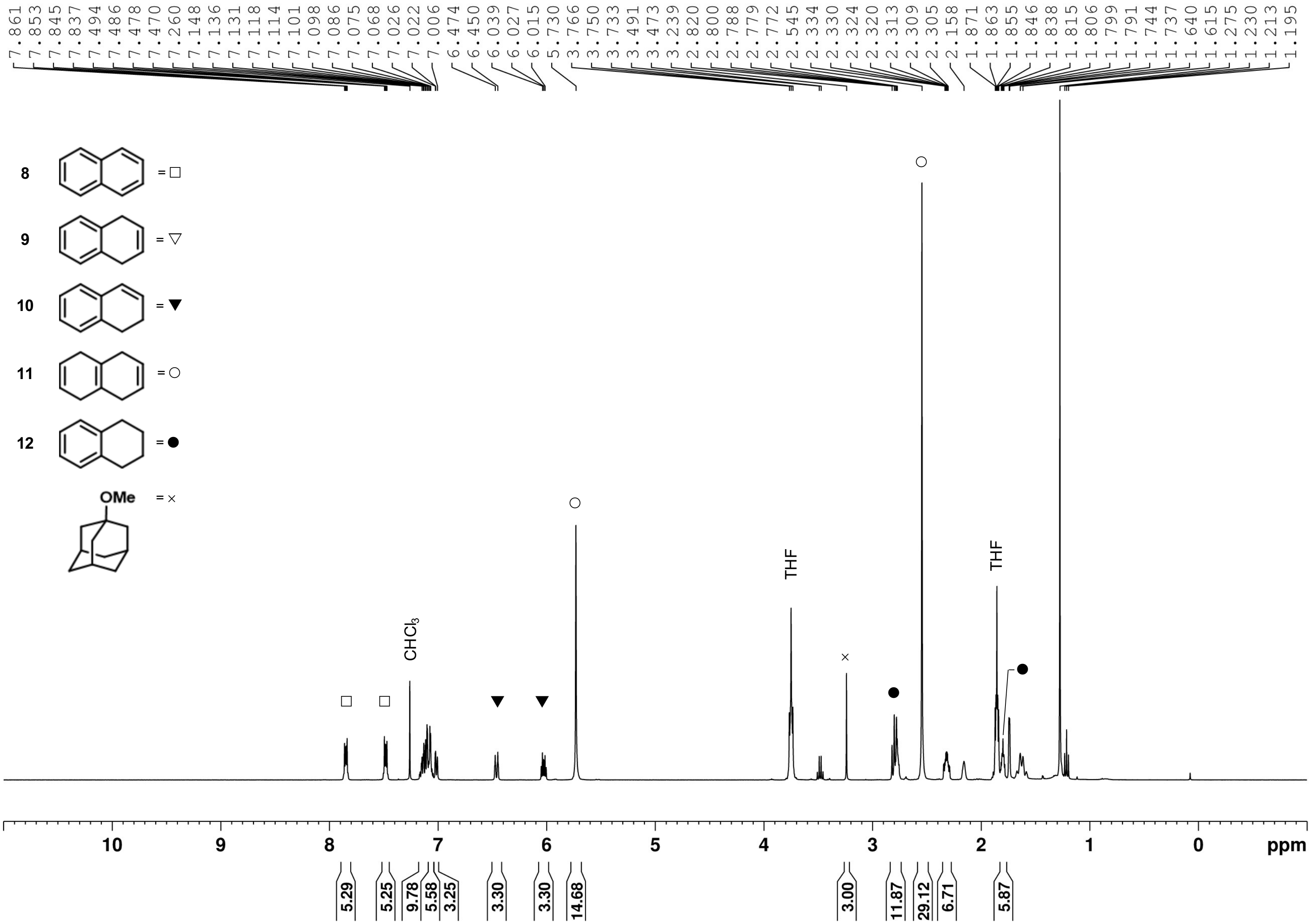

Continuation of Spectrum S28.

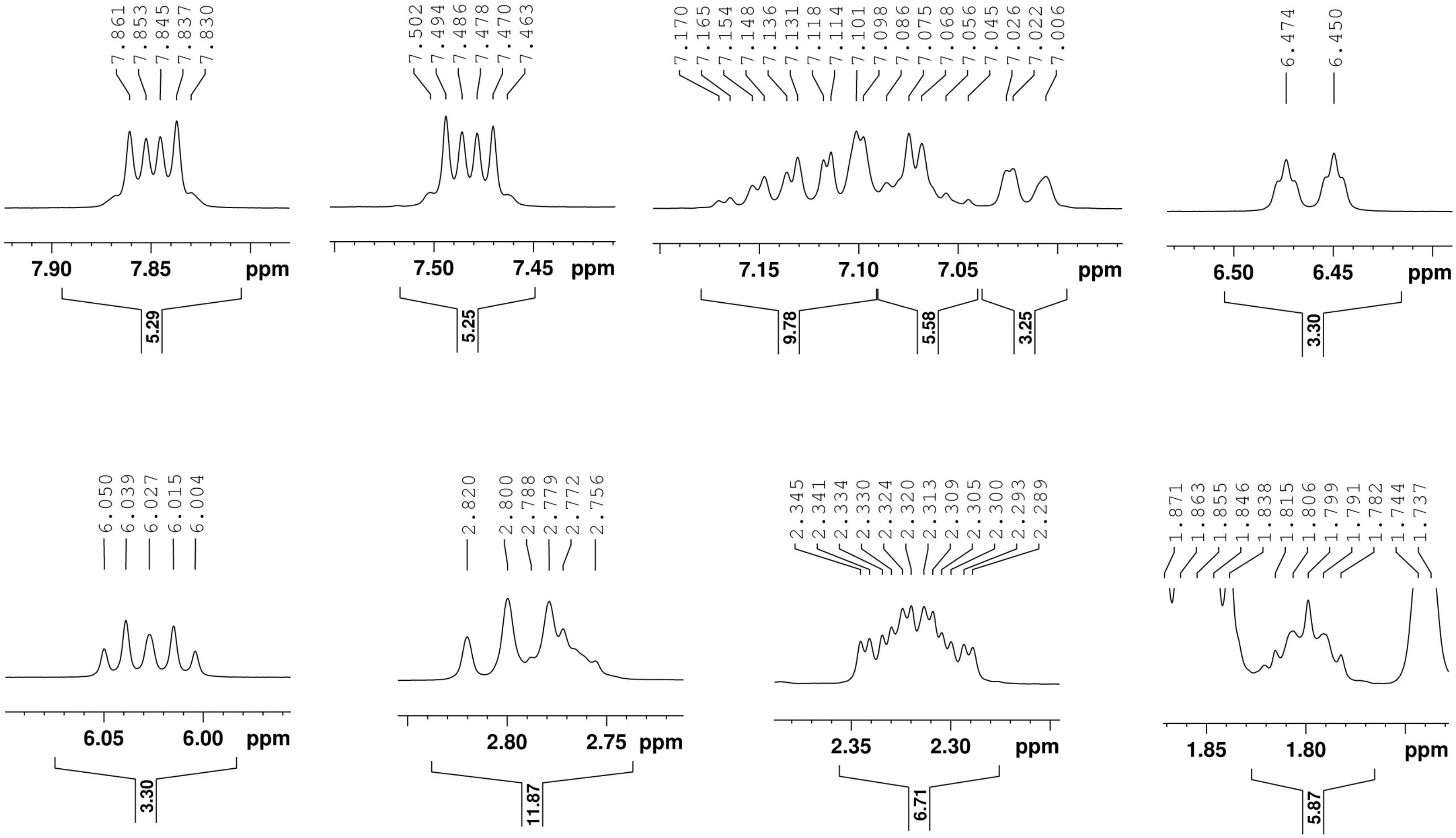

Spectrum S29. ^{1}H NMR spectrum of the reduction of naphthalene (**8**) under air using general method A, Table 2 entry 2 (400 MHz, $CDCl_3$, 298 K).

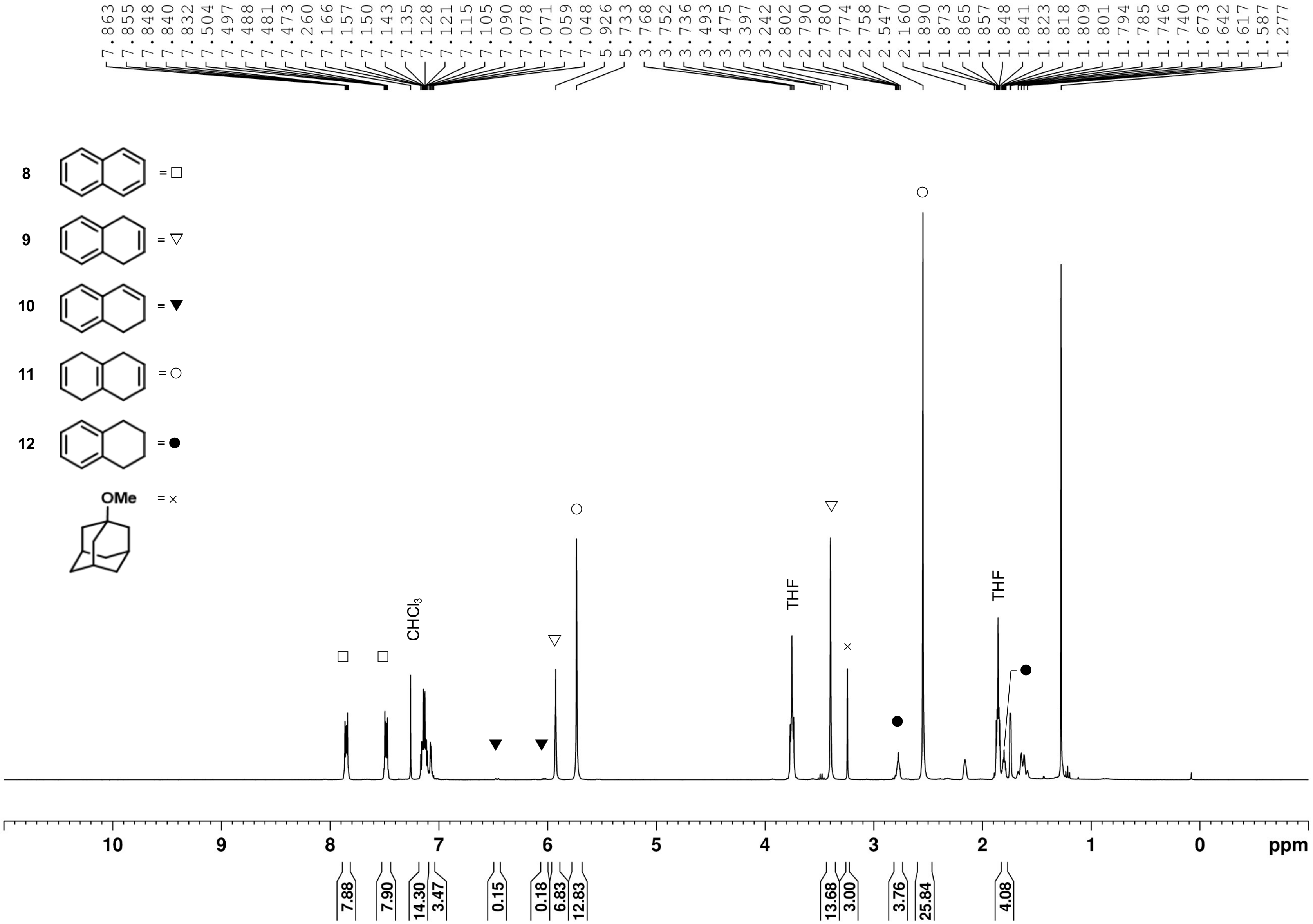

Continuation of Spectrum S29.

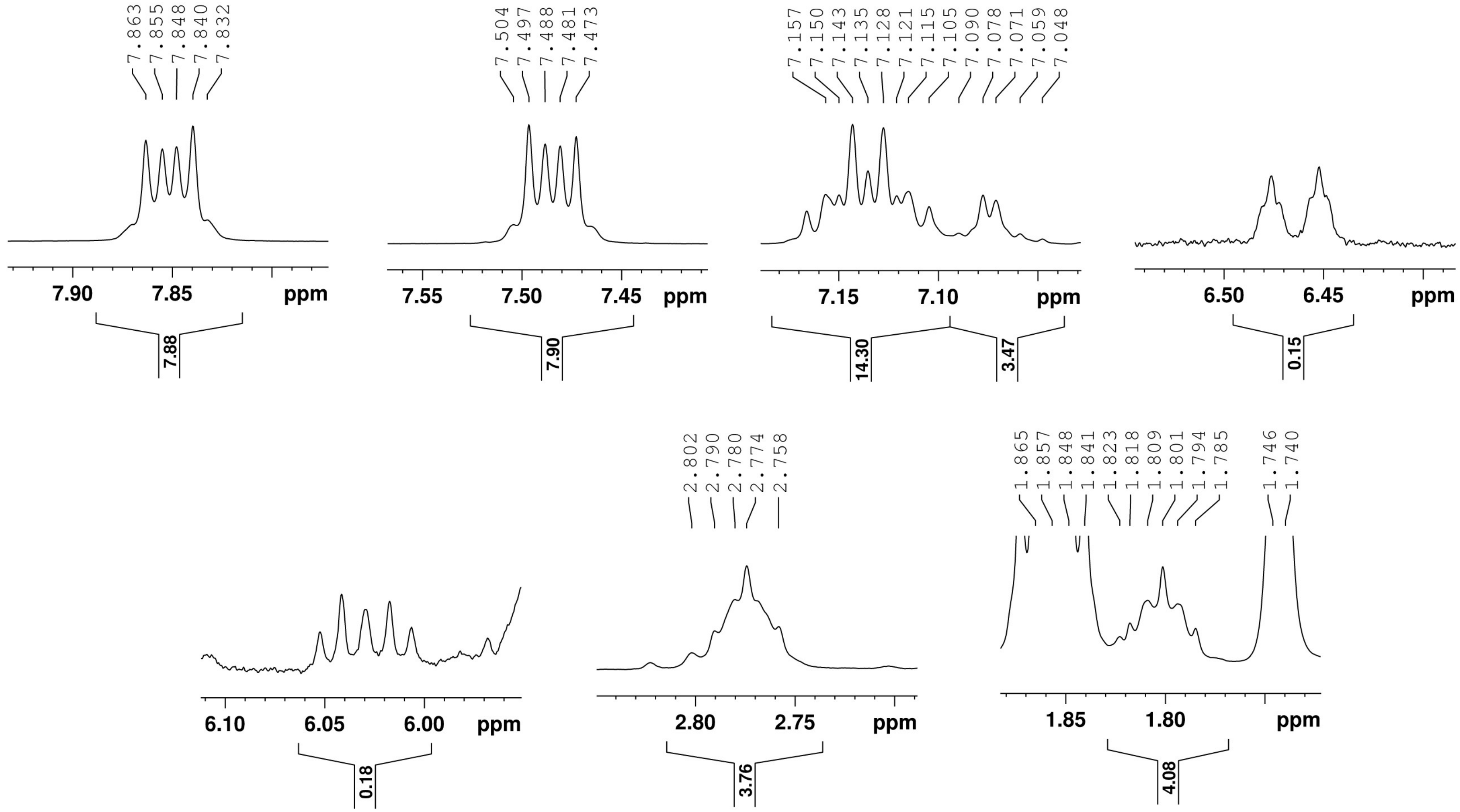

Spectrum S30. ^{1}H NMR spectrum of the reduction of naphthalene (**8**) under O_2 using general method B, Table 2 entry 3 (500 MHz, $CDCl_3$, 298 K).

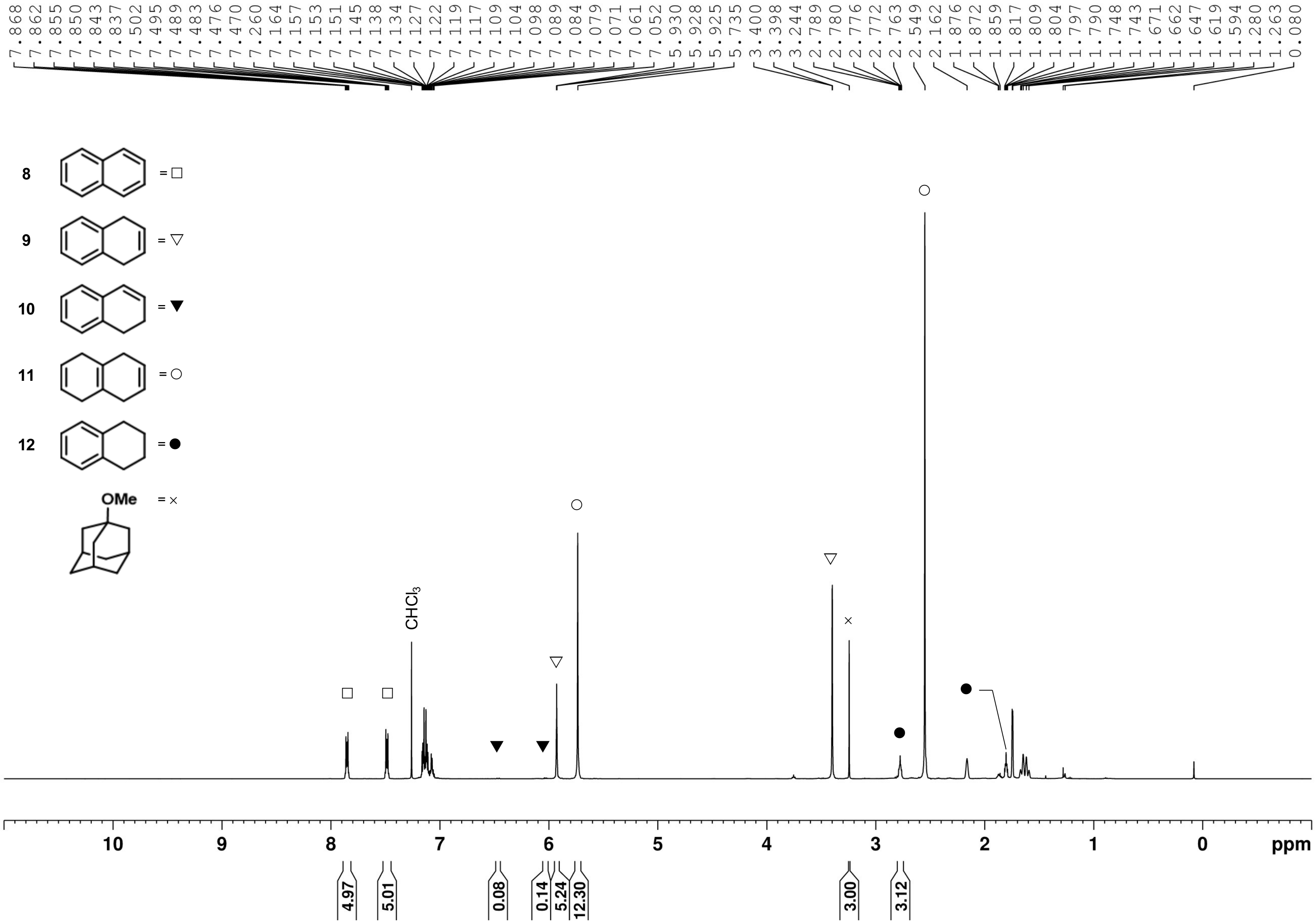

Spectrum S31. ^{1}H NMR spectrums of the reduction of naphthalene (**8**) under Ar using the general kinetic method, Figure 2a (400 MHz, $CDCl_3$, 298 K).

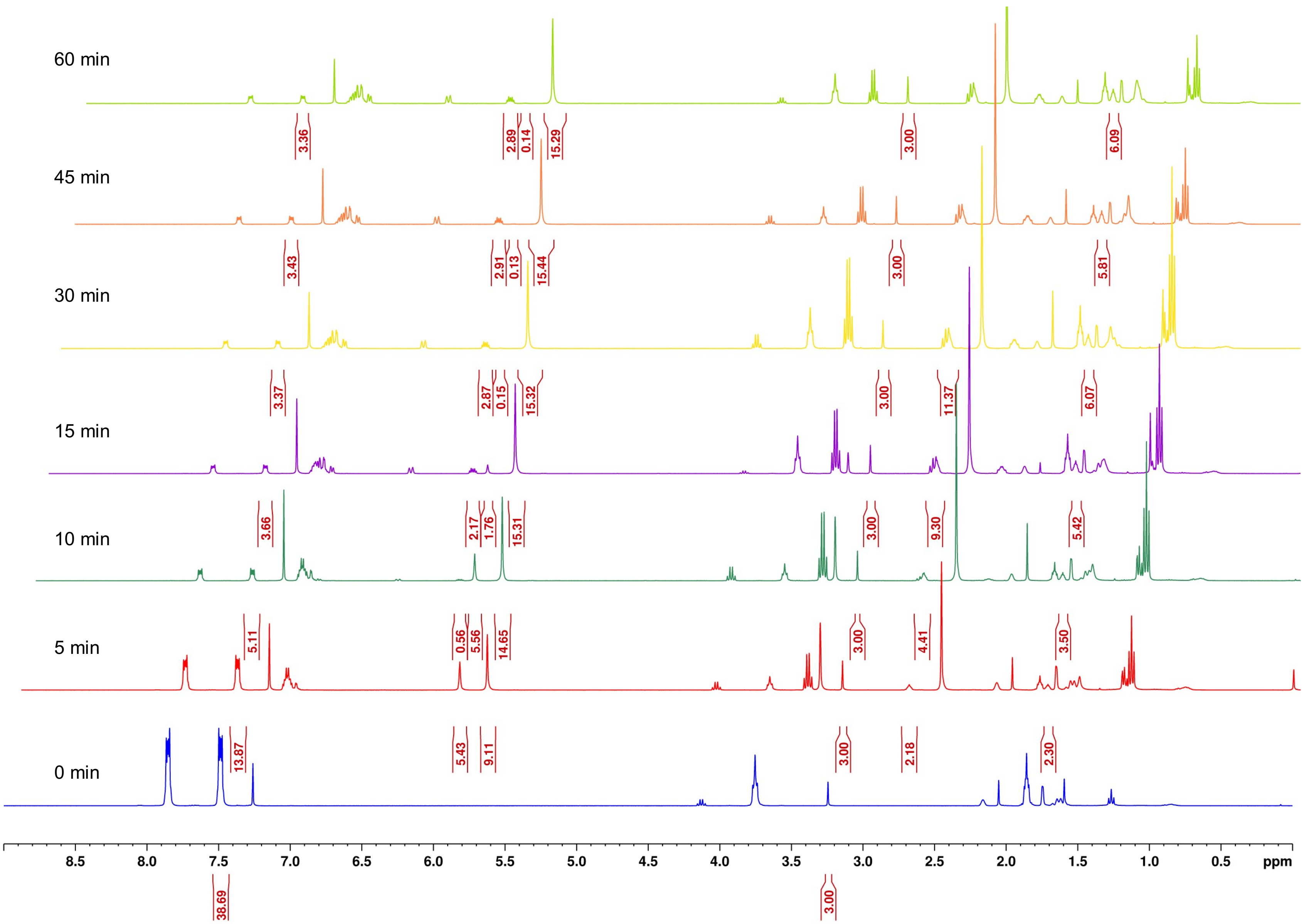

Spectrum S32. ^{1}H NMR spectrums of the reduction of naphthalene (**8**) under O_2 using the general kinetic method, Figure 2b (400 MHz, $CDCl_3$, 298 K).

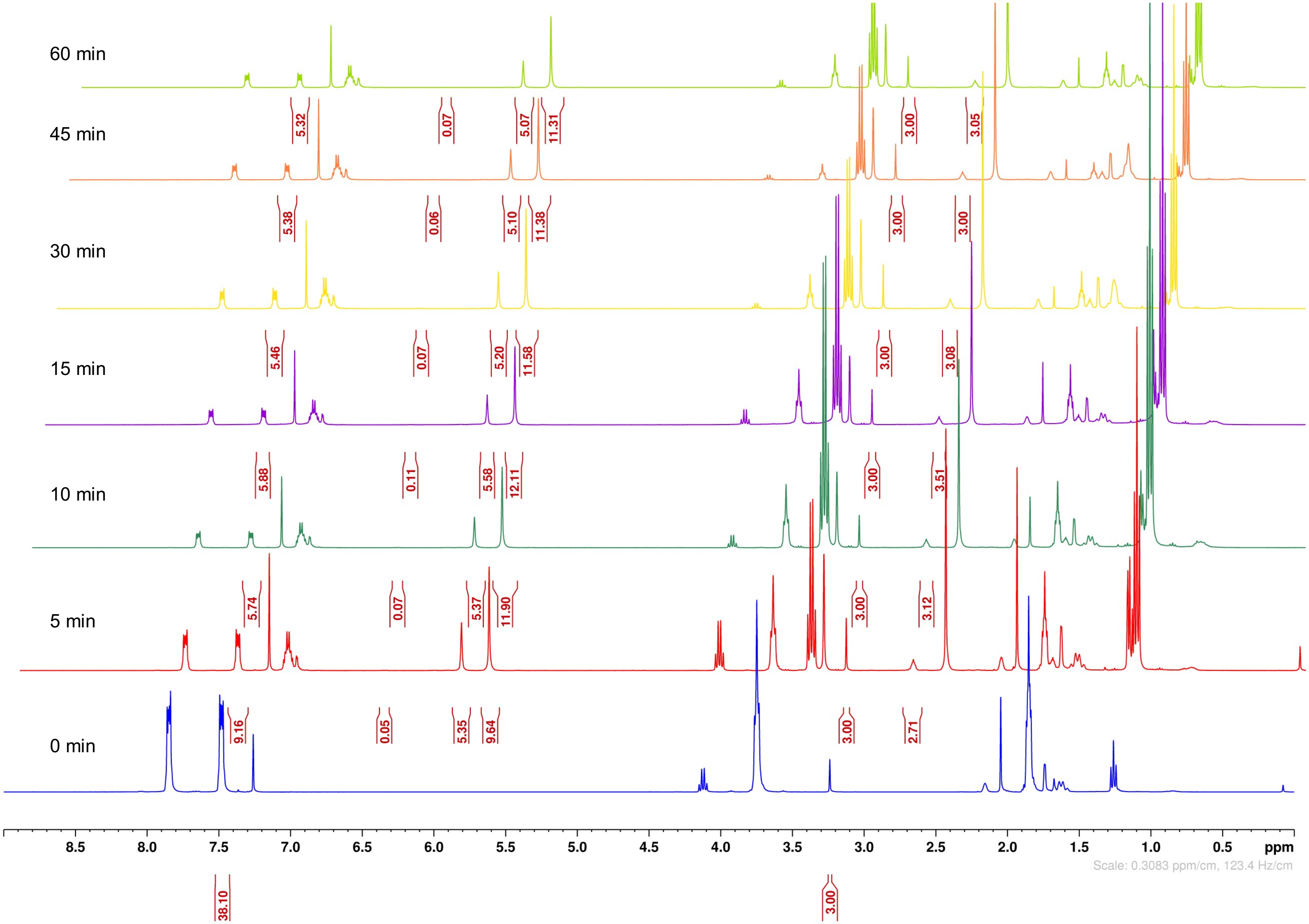

Spectrum S33. ^{1}H NMR spectrum of Table 3, entry 1 (300 MHz, $CDCl_3$, 298 K).

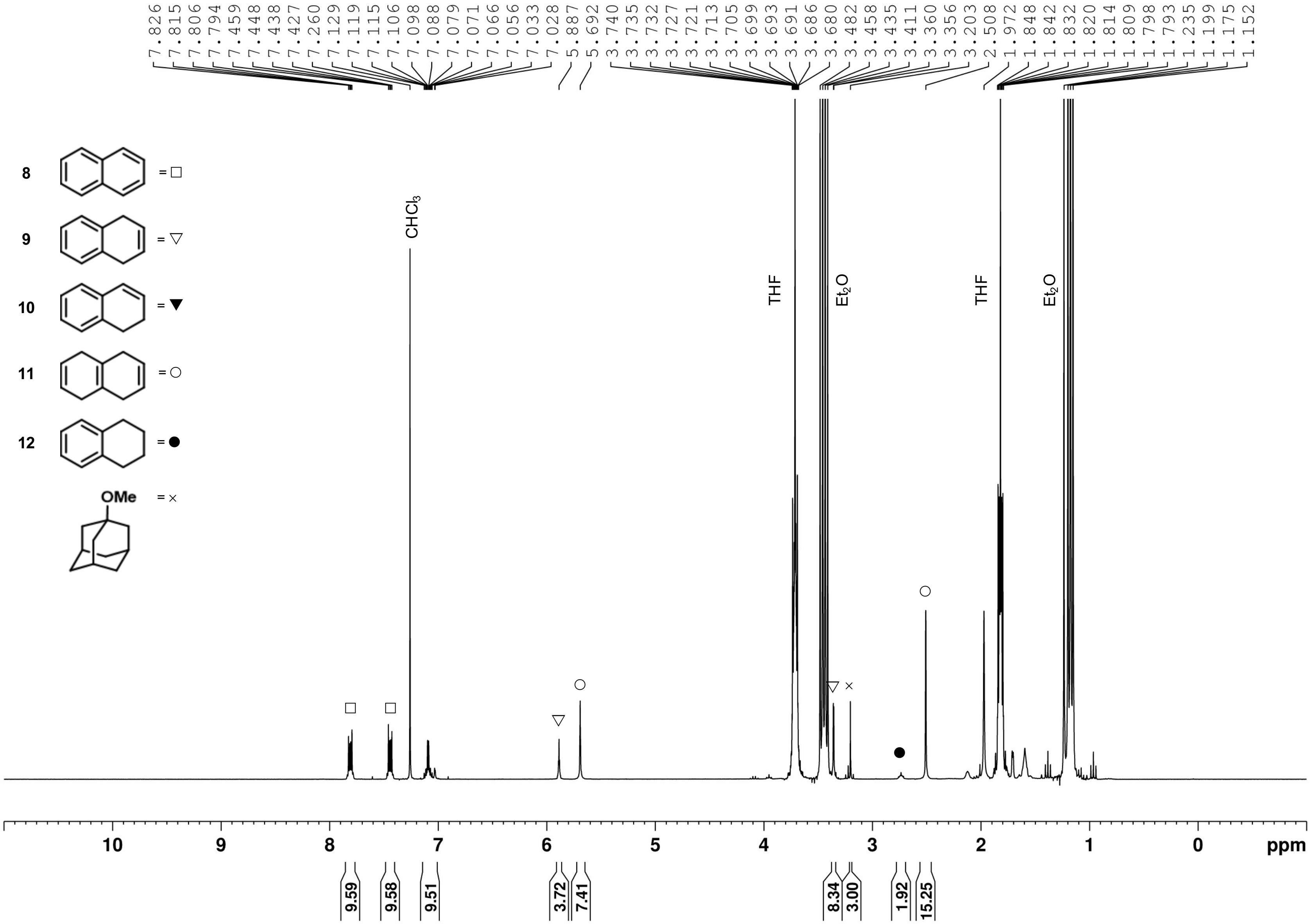

Spectrum S34. ^{1}H NMR spectrum of Table 3, entry 2 (300 MHz, $CDCl_3$, 298 K).

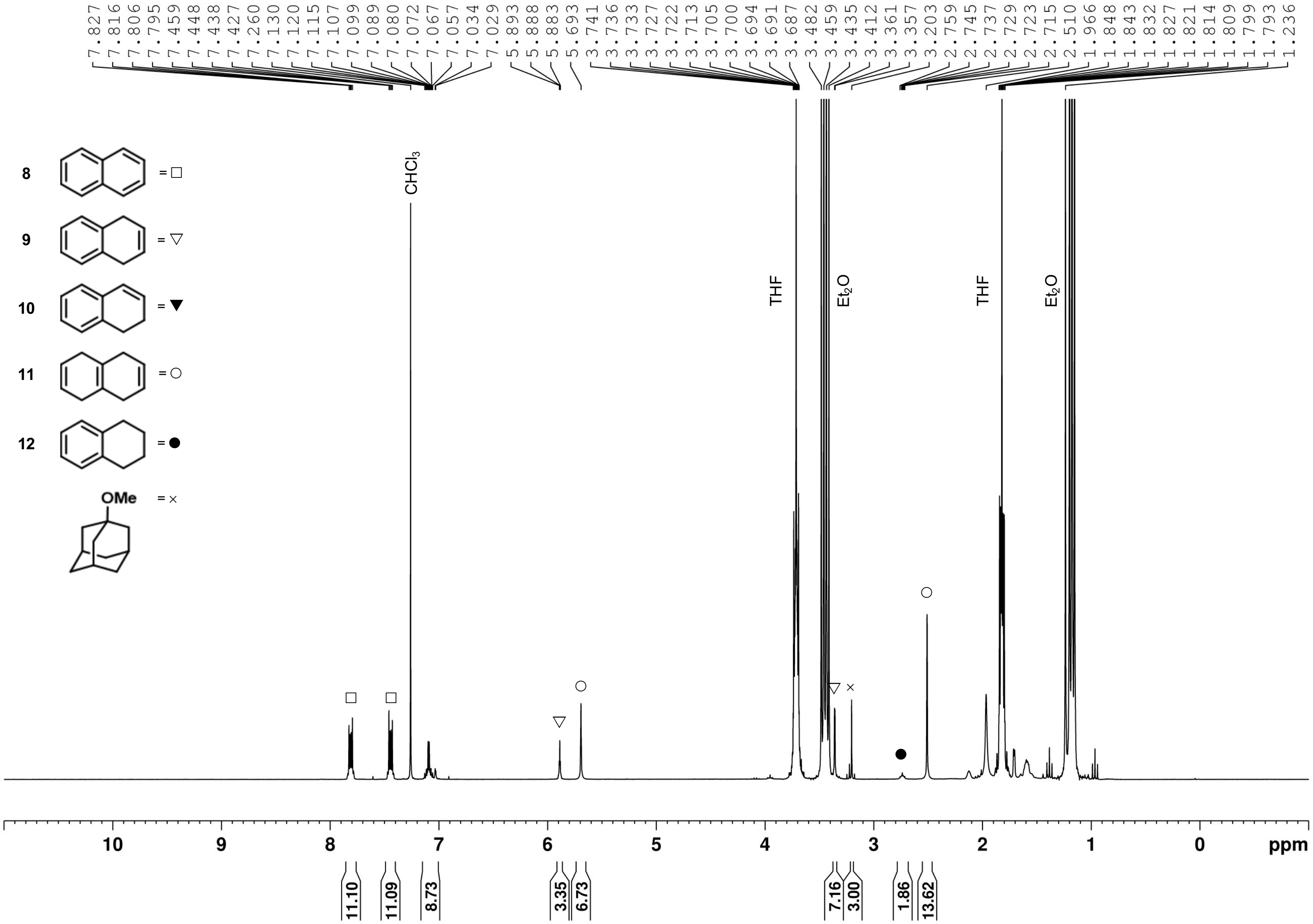

Spectrum S35. ^{1}H NMR spectrum of Table 3, entry 3 (300 MHz, $CDCl_3$, 298 K).

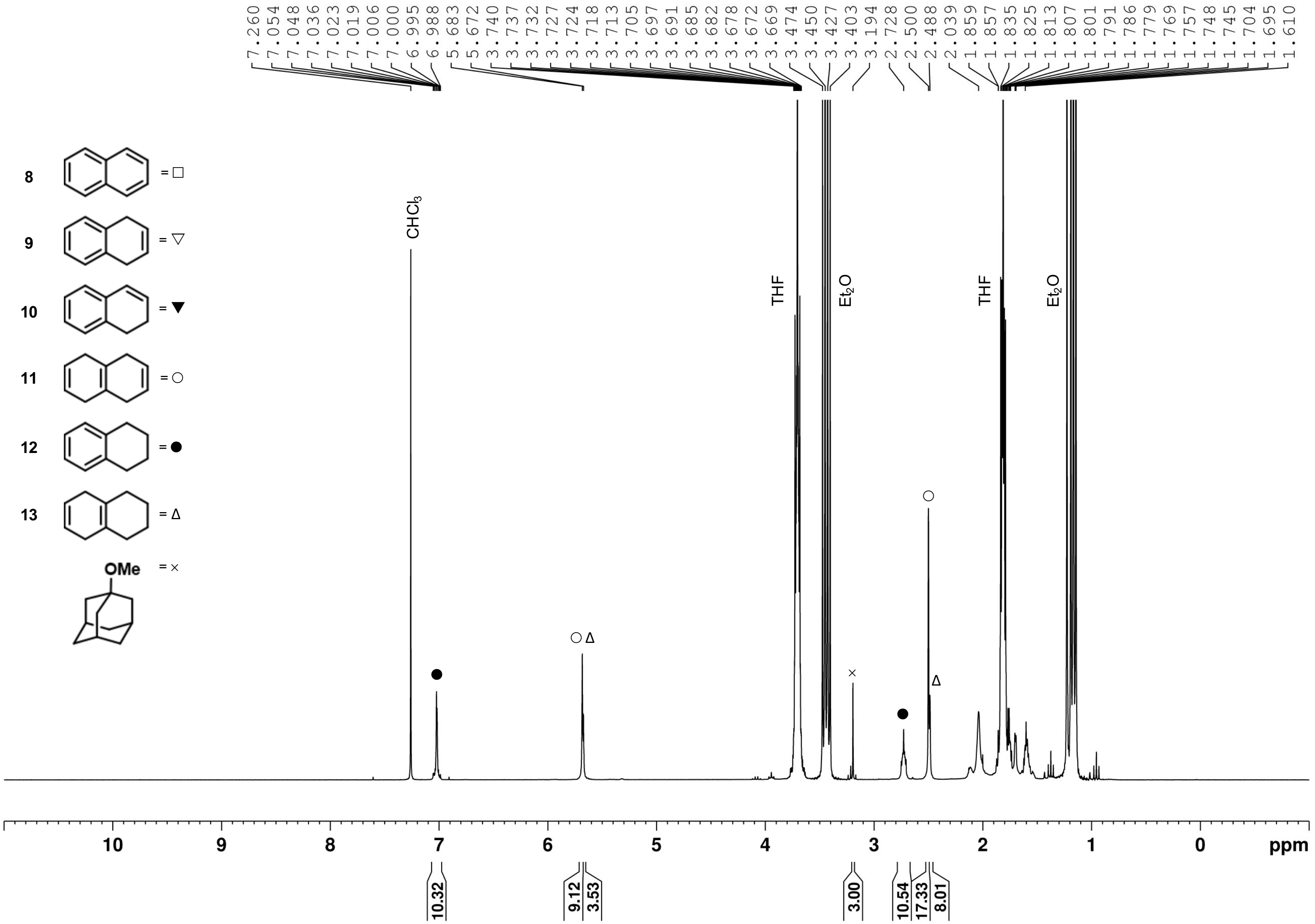

Continuation of Spectrum S35.

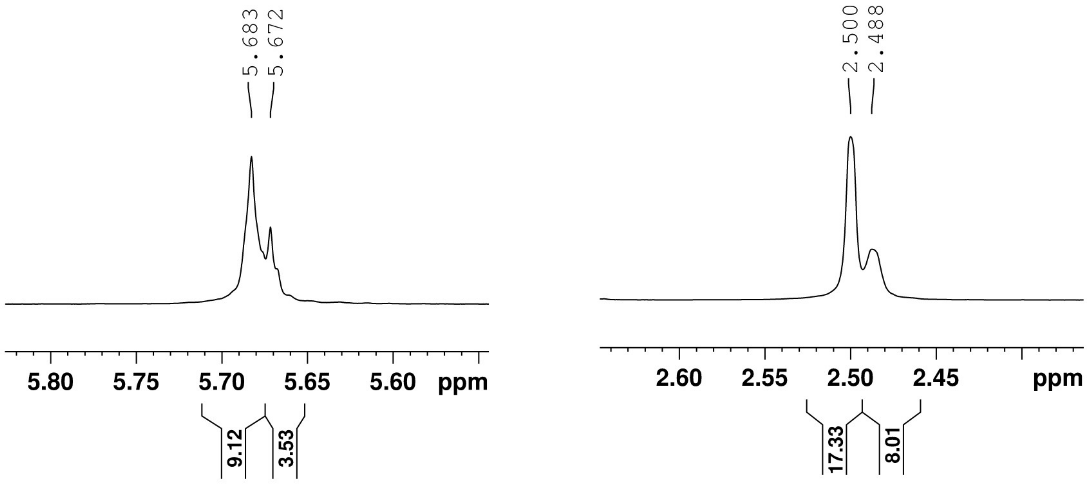

Spectrum S36. ^{1}H NMR spectrum of Table 3, entry 4 (300 MHz, $CDCl_3$, 298 K).

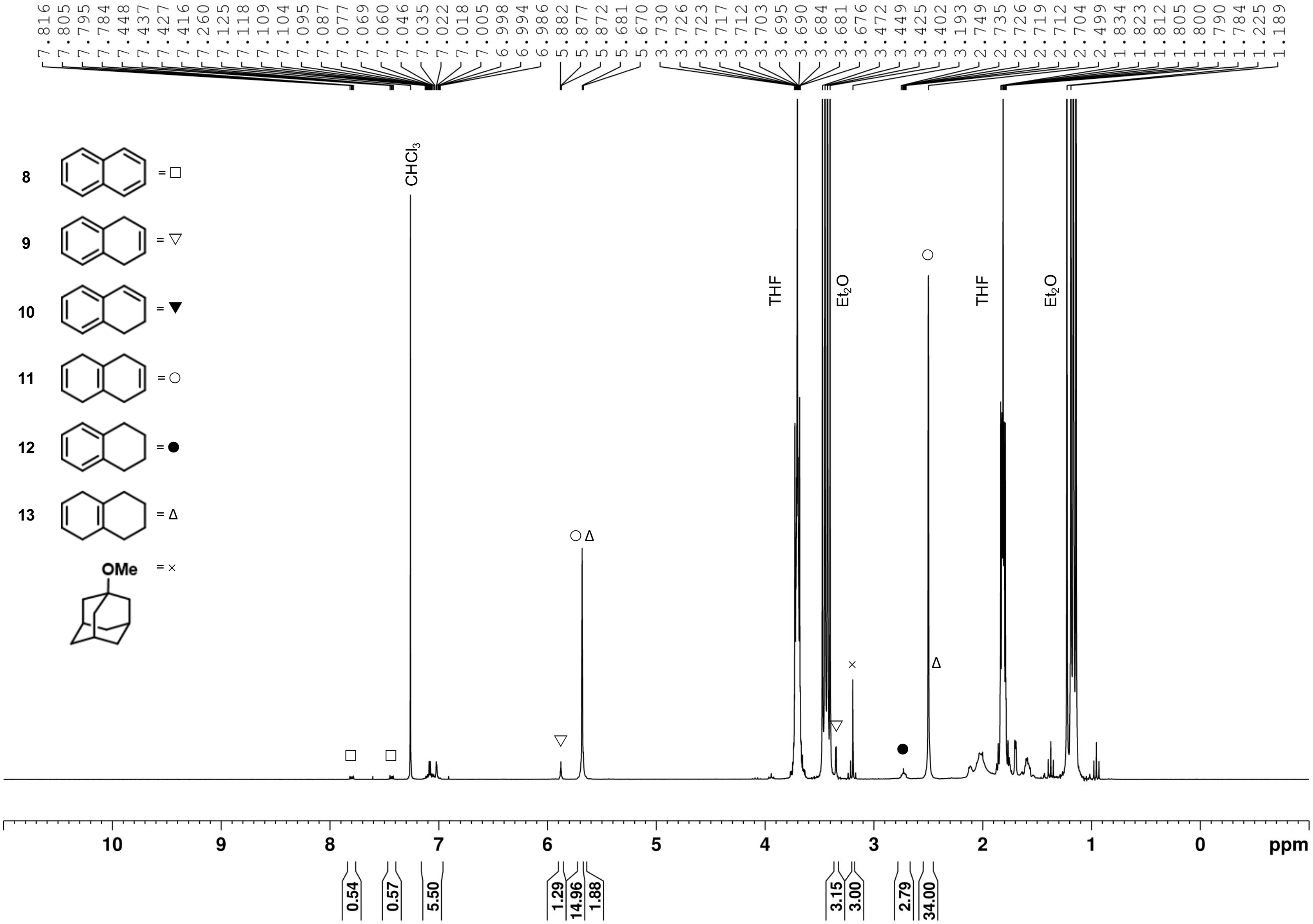

Continuation of Spectrum S36.

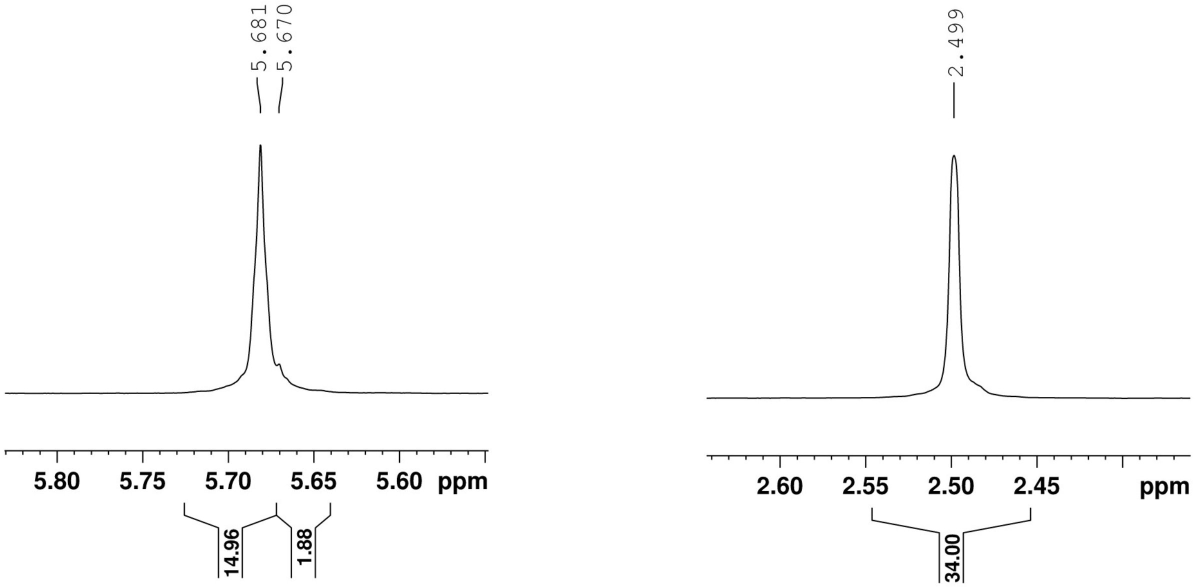

Spectrum S37. ^{1}H NMR spectrum of Table 4, entry 1 (300 MHz, $CDCl_3$, 298 K).

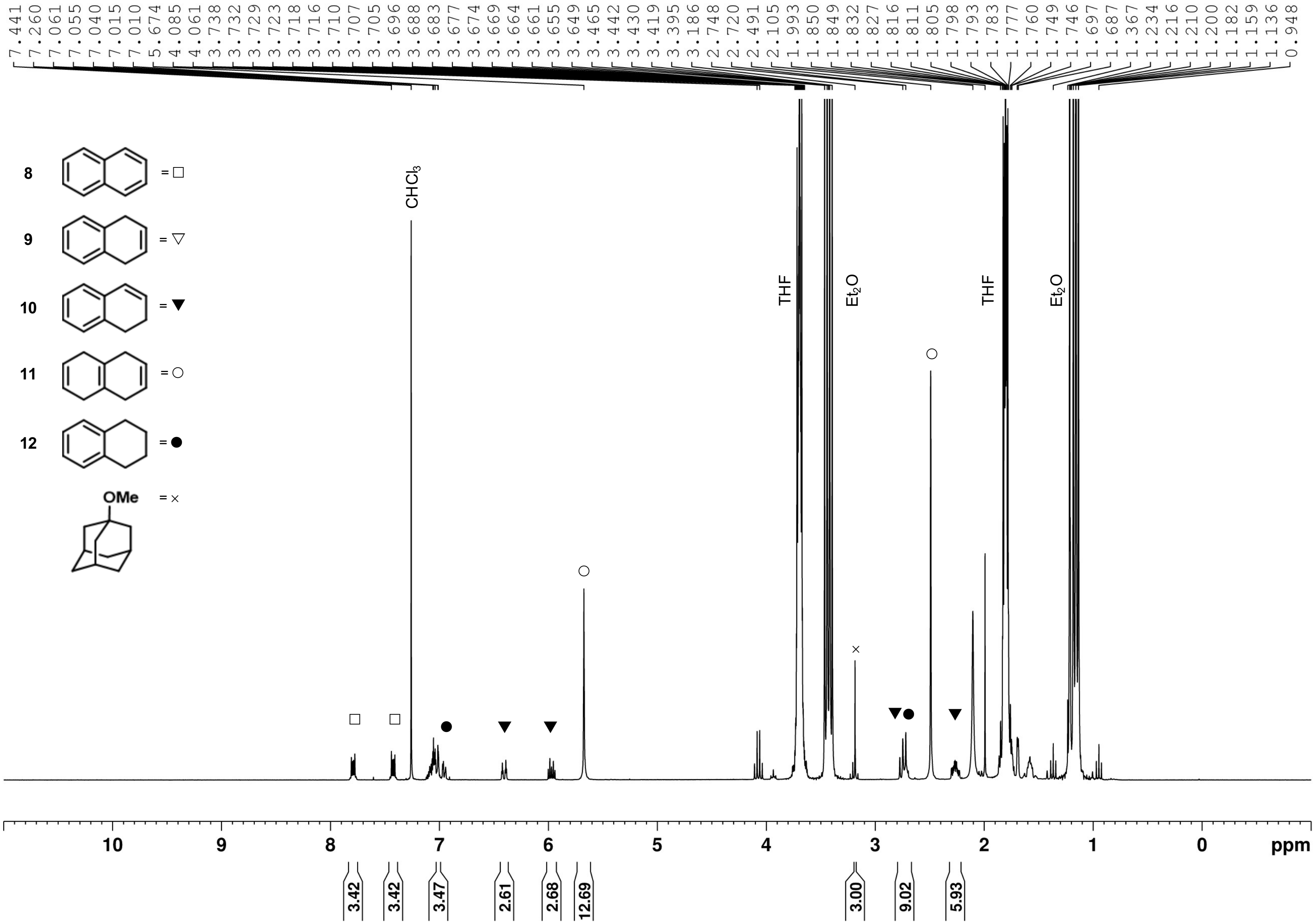

Spectrum S38. ^{1}H NMR spectrum of Table 4, entry 2 (300 MHz, $CDCl_3$, 298 K).

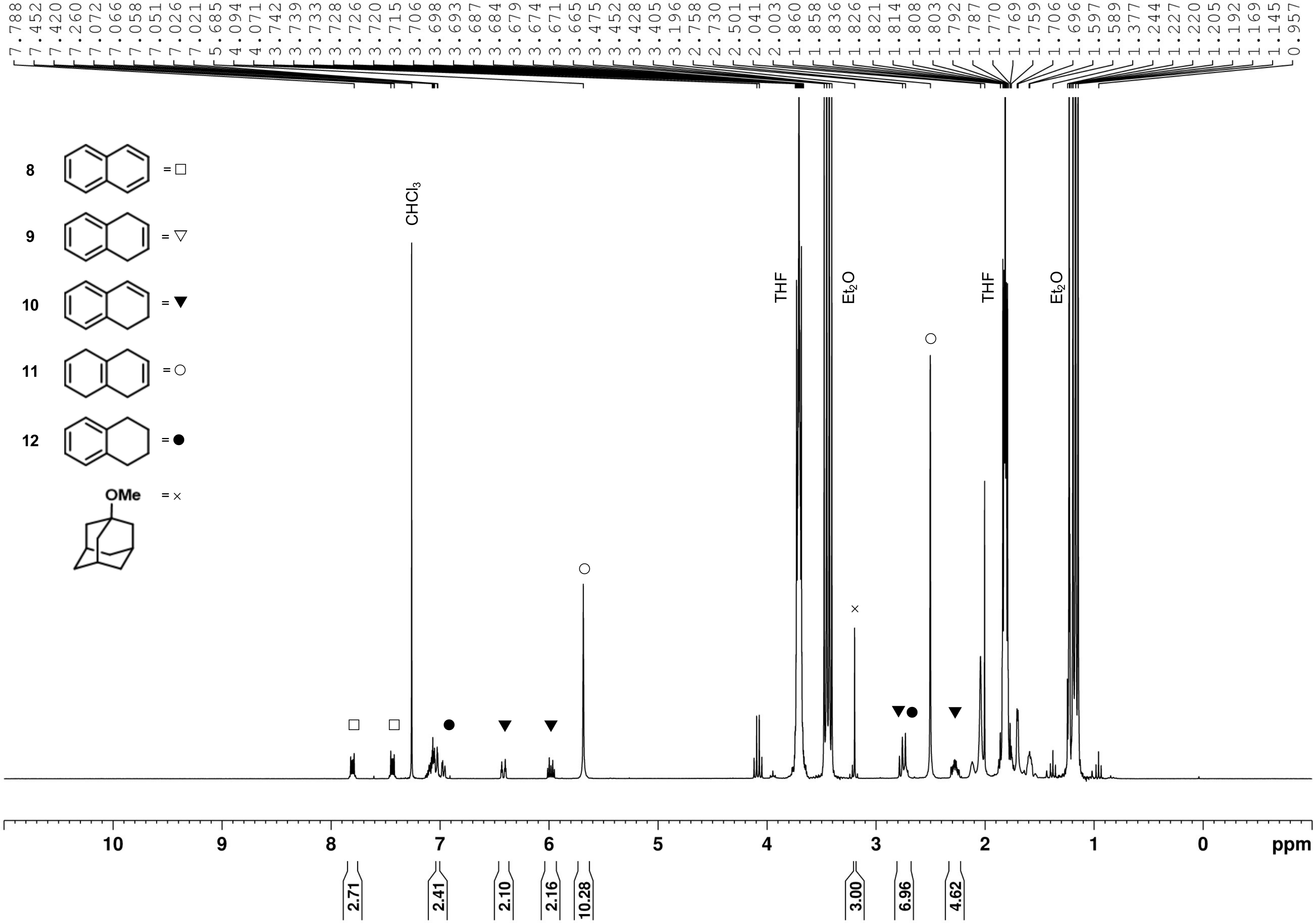

Spectrum S39. ^{1}H NMR spectrum of Table 4, entry 3 (300 MHz, $CDCl_3$, 298 K).

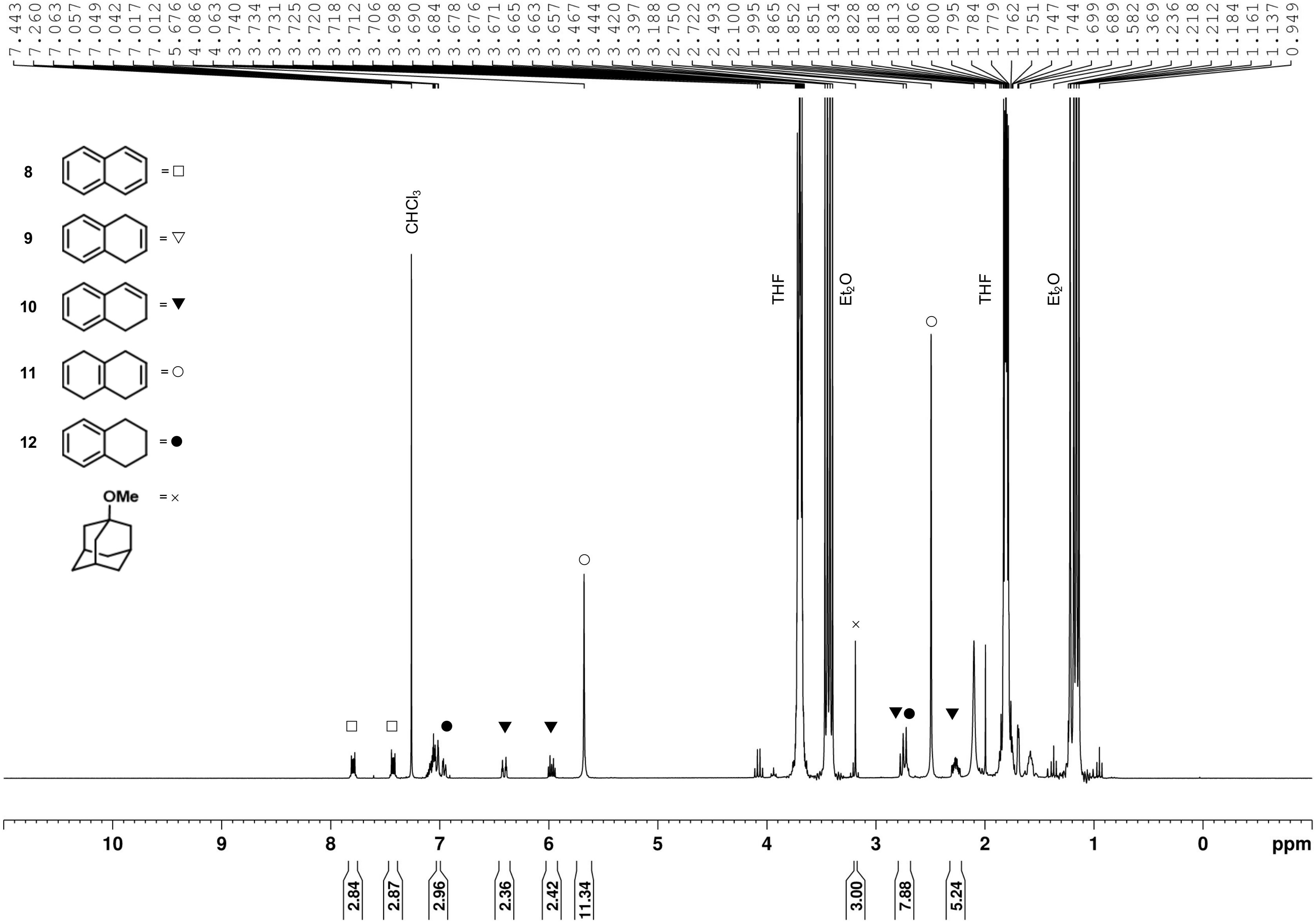

Spectrum S40. ^{1}H NMR spectrum of Table 4, entry 4 (300 MHz, $CDCl_3$, 298 K).

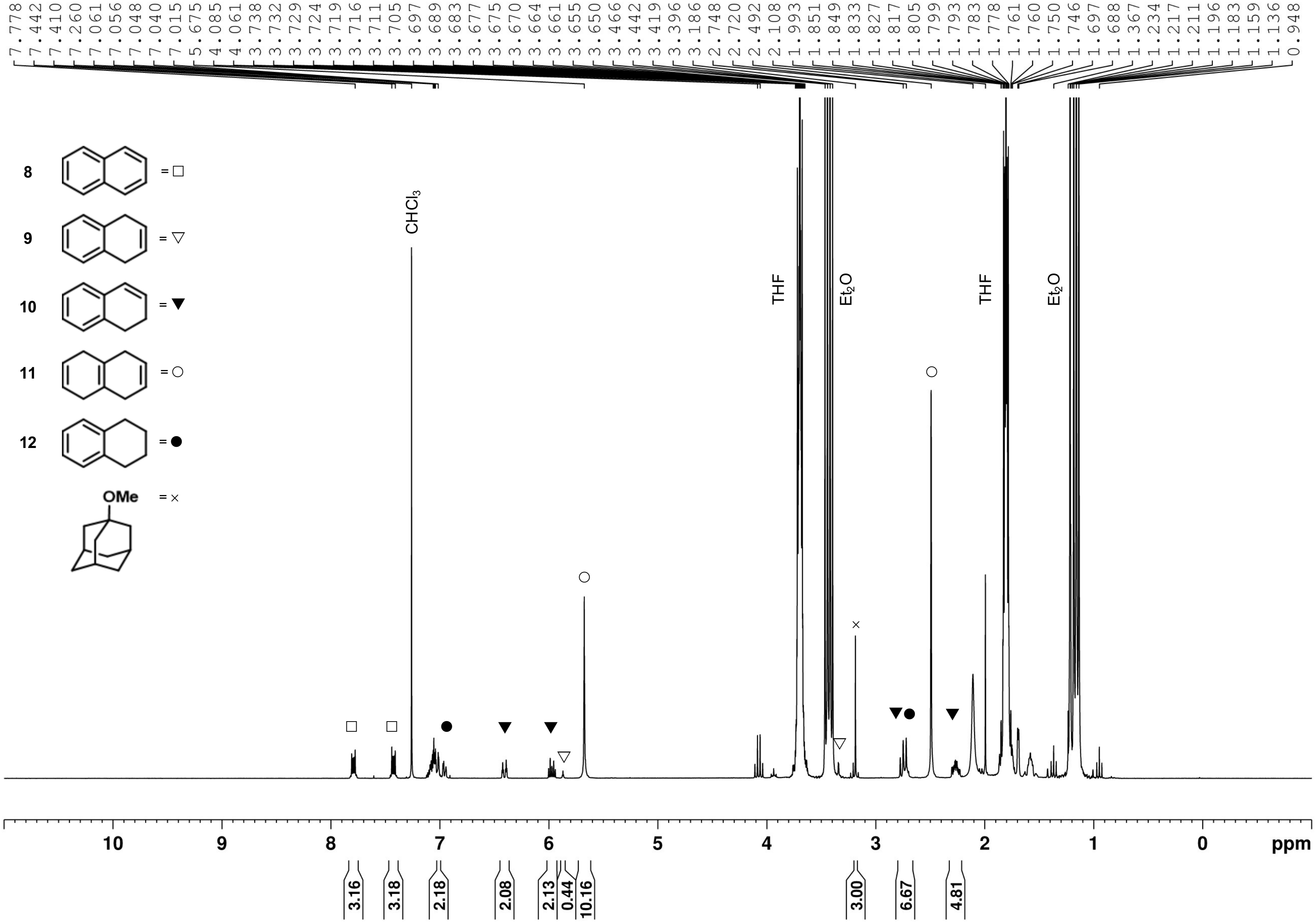

Spectrum S41. ^{1}H NMR spectrum of Table 4, entry 5 (300 MHz, $CDCl_3$, 298 K).

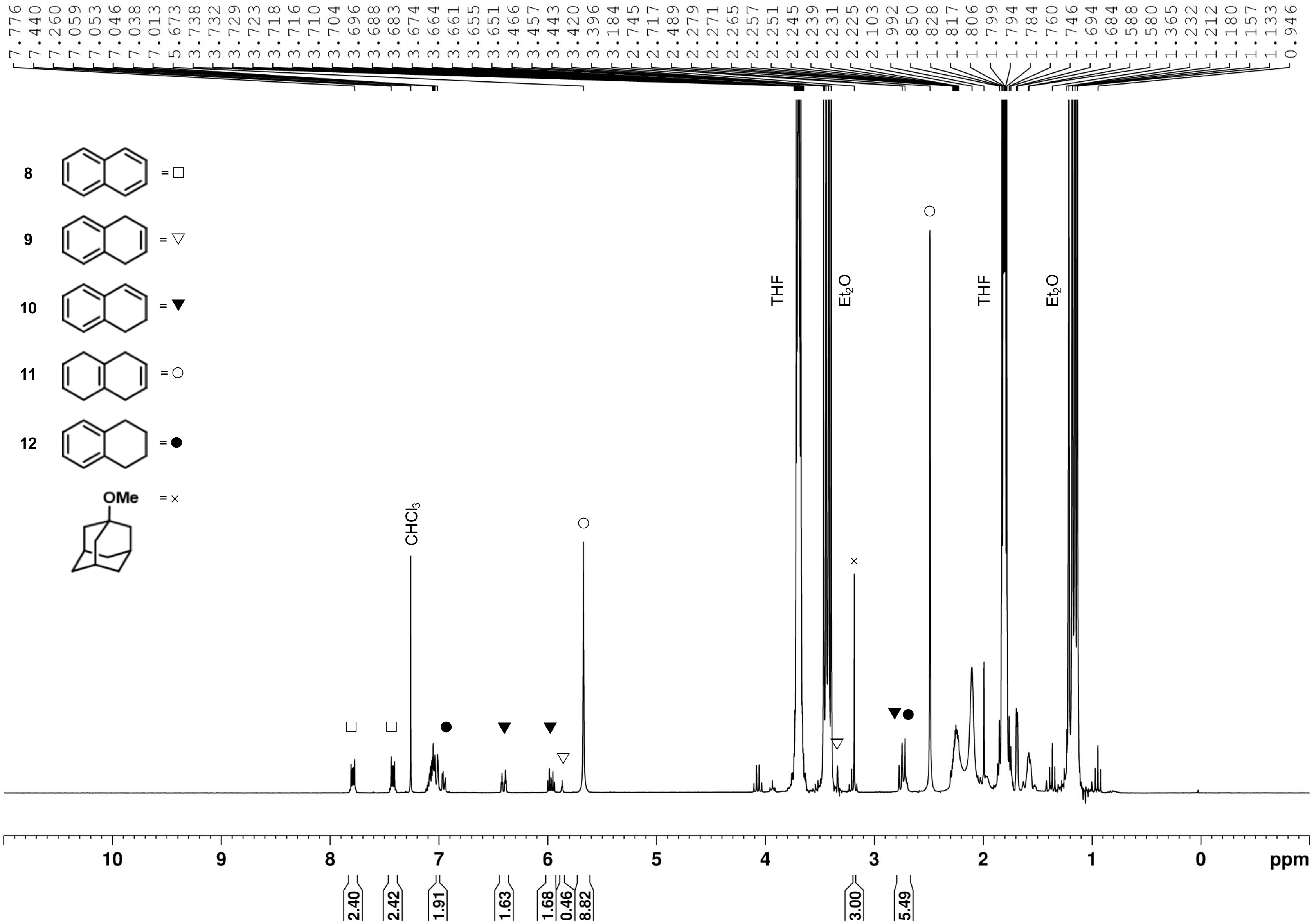

Spectrum S42. ^{1}H NMR spectrum of Table 4, entry 6 (300 MHz, $CDCl_3$, 298 K).

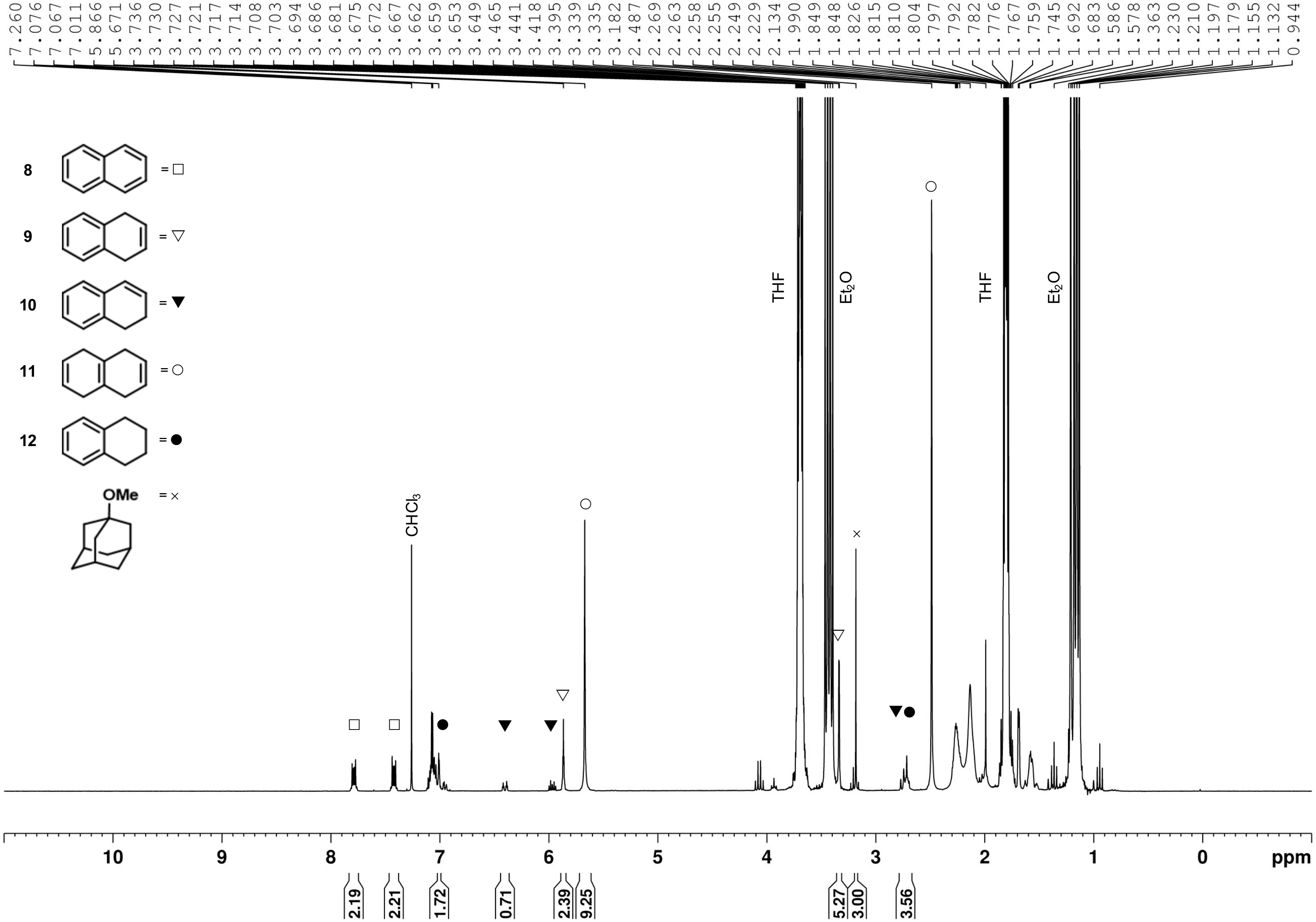

Spectrum S43. ^{1}H NMR spectrum of Table 4, entry 7 (300 MHz, $CDCl_3$, 298 K).

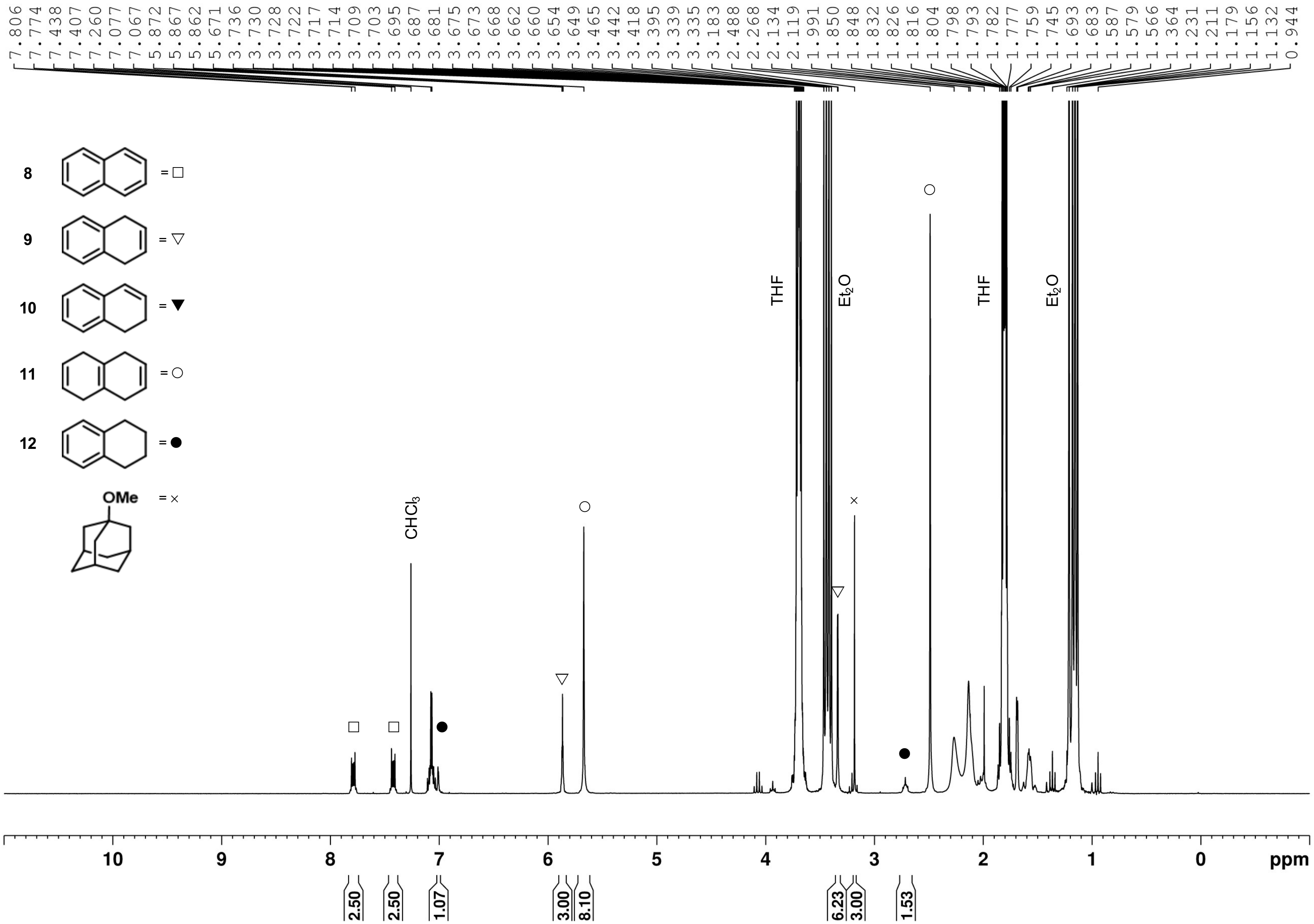

Spectrum S44. ^{1}H NMR spectrums of the reaction of 1,4-dihydronaphthalene (**9**) with KO_2 (300 MHz, $CDCl_3$, 298 K).

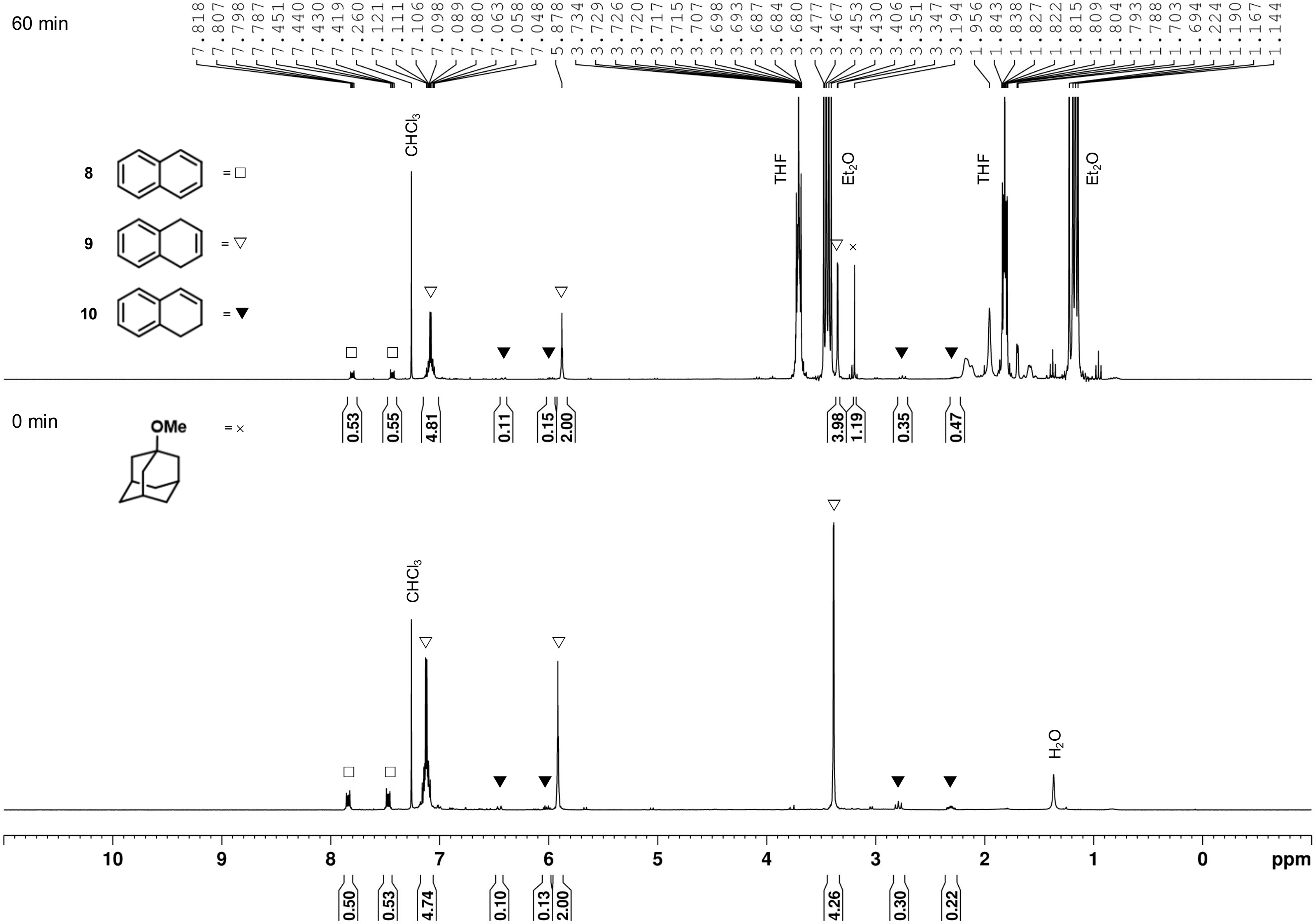

Spectrum S45. ^{1}H NMR spectrums of the reaction of 1,2-dihydronaphthalene (**10**) with KO_2 (300 MHz, $CDCl_3$, 298 K).

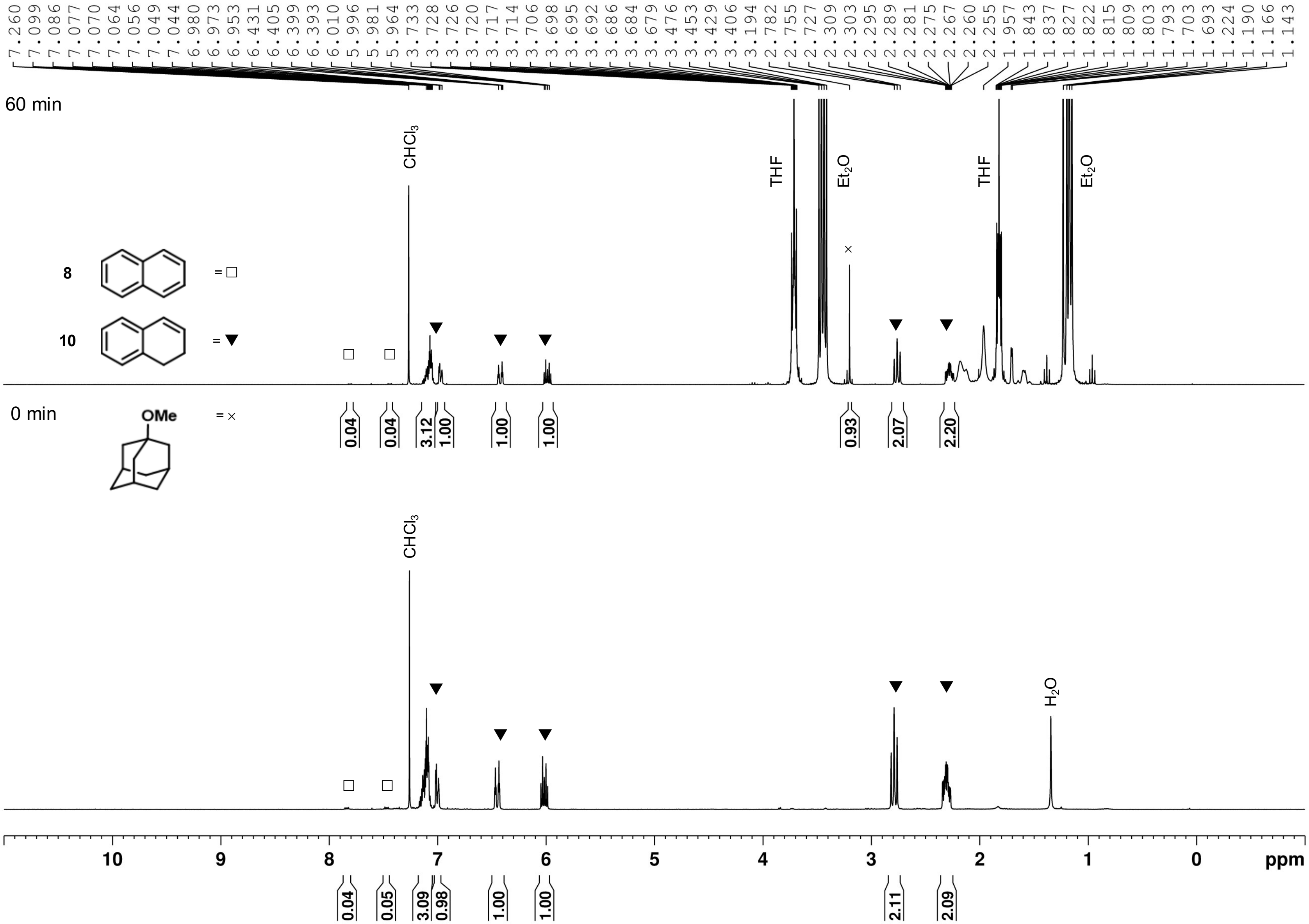

Spectrum S46. ^{1}H NMR spectrum of Table S5, entry 1 (300 MHz, $CDCl_3$, 298 K).

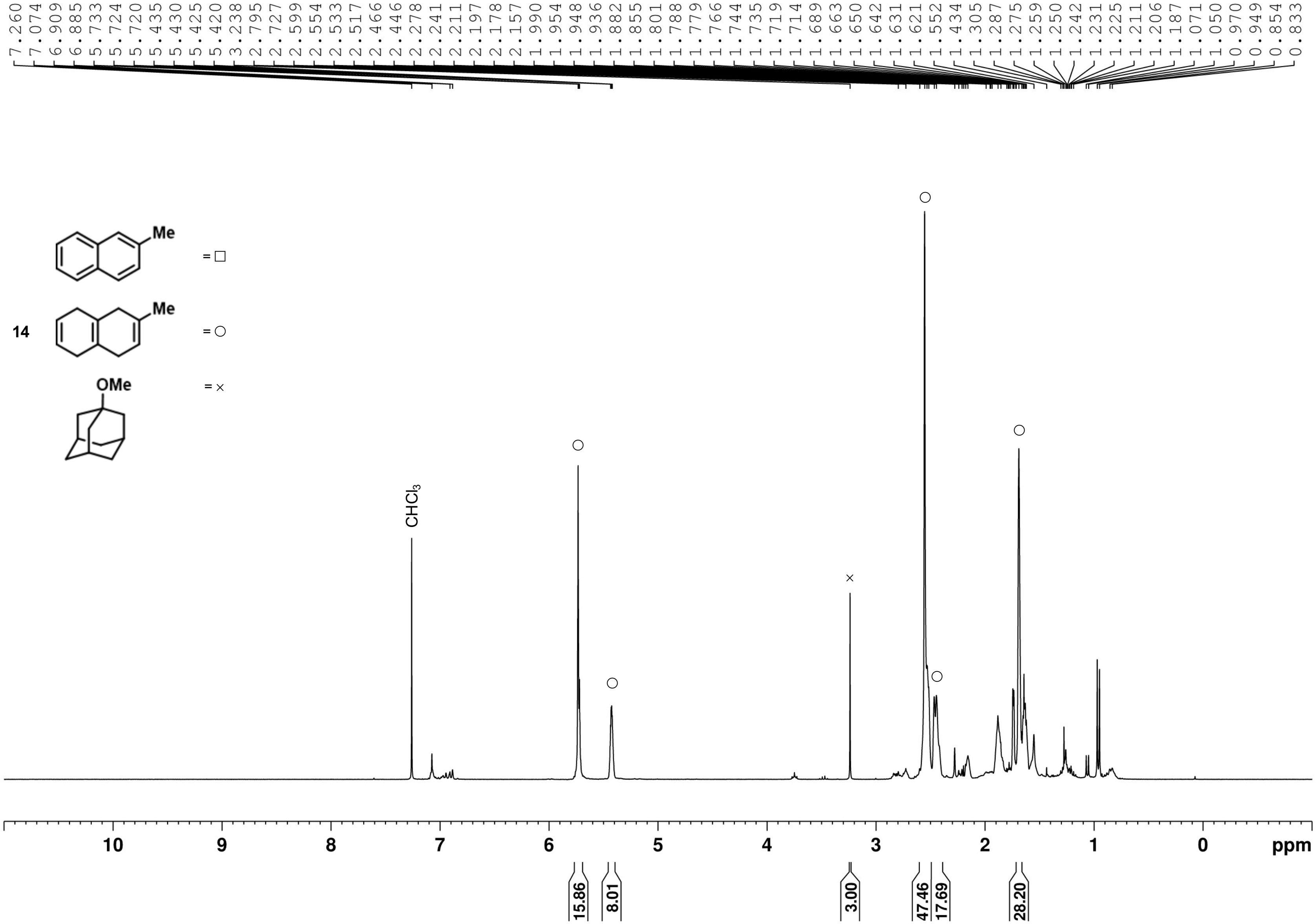

Spectrum S47. ^{1}H NMR spectrum of Table S5, entry 2 (300 MHz, $CDCl_3$, 298 K).

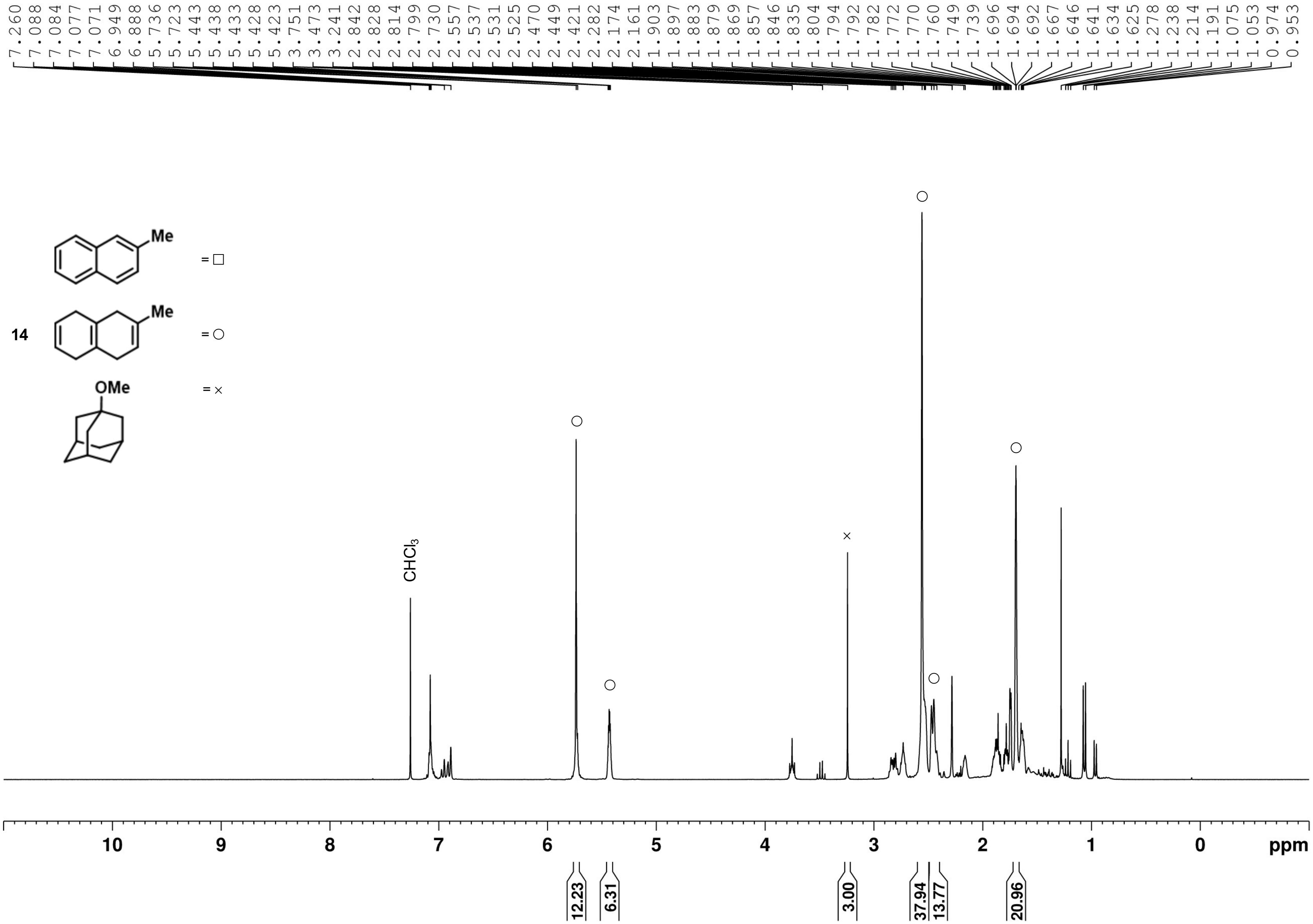

Spectrum S48. ^{1}H NMR spectrum of Table S5, entry 3 (300 MHz, $CDCl_3$, 298 K).

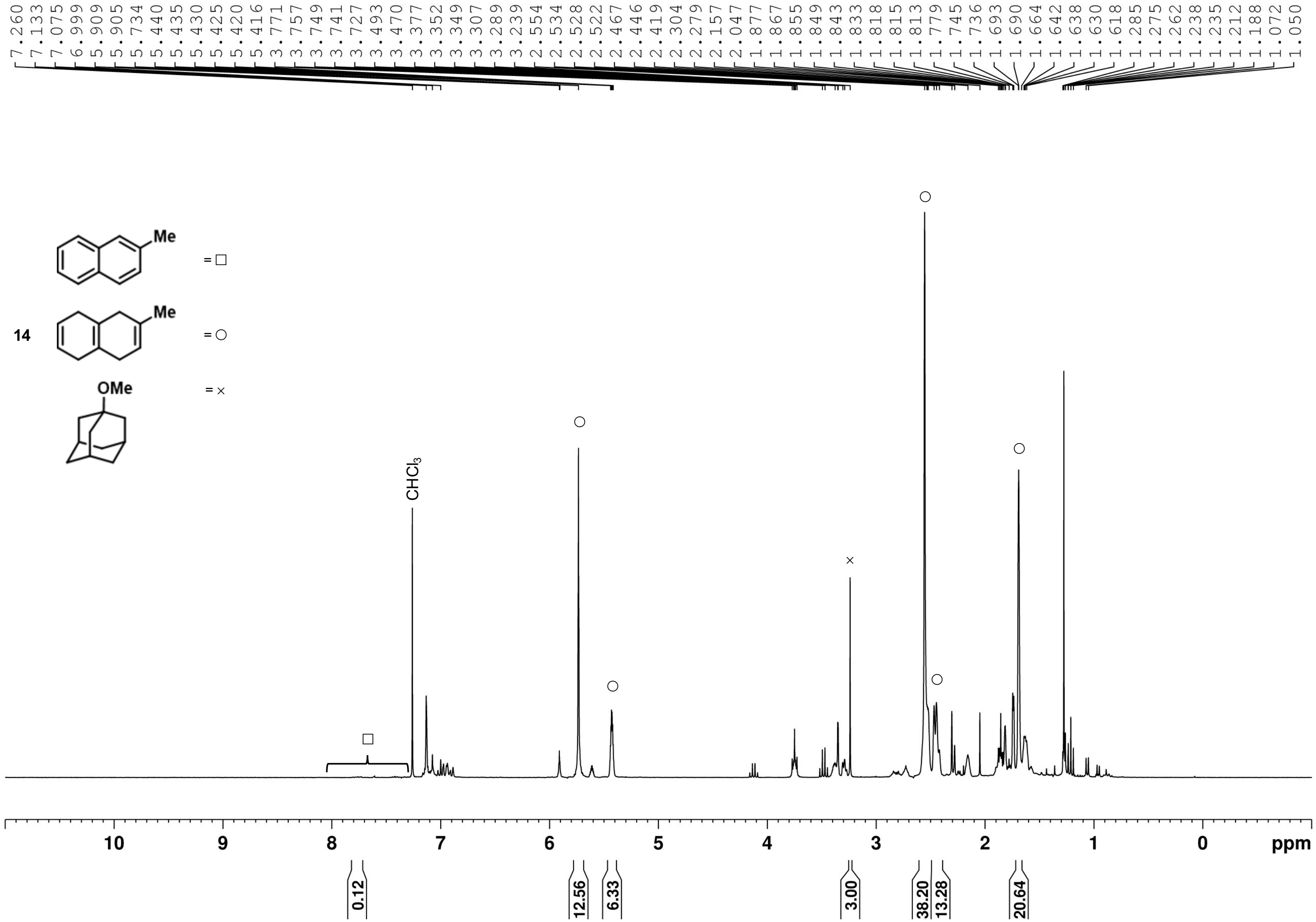

Spectrum S49. ^{1}H NMR spectrum of Table S5, entry 4 (300 MHz, $CDCl_3$, 298 K).

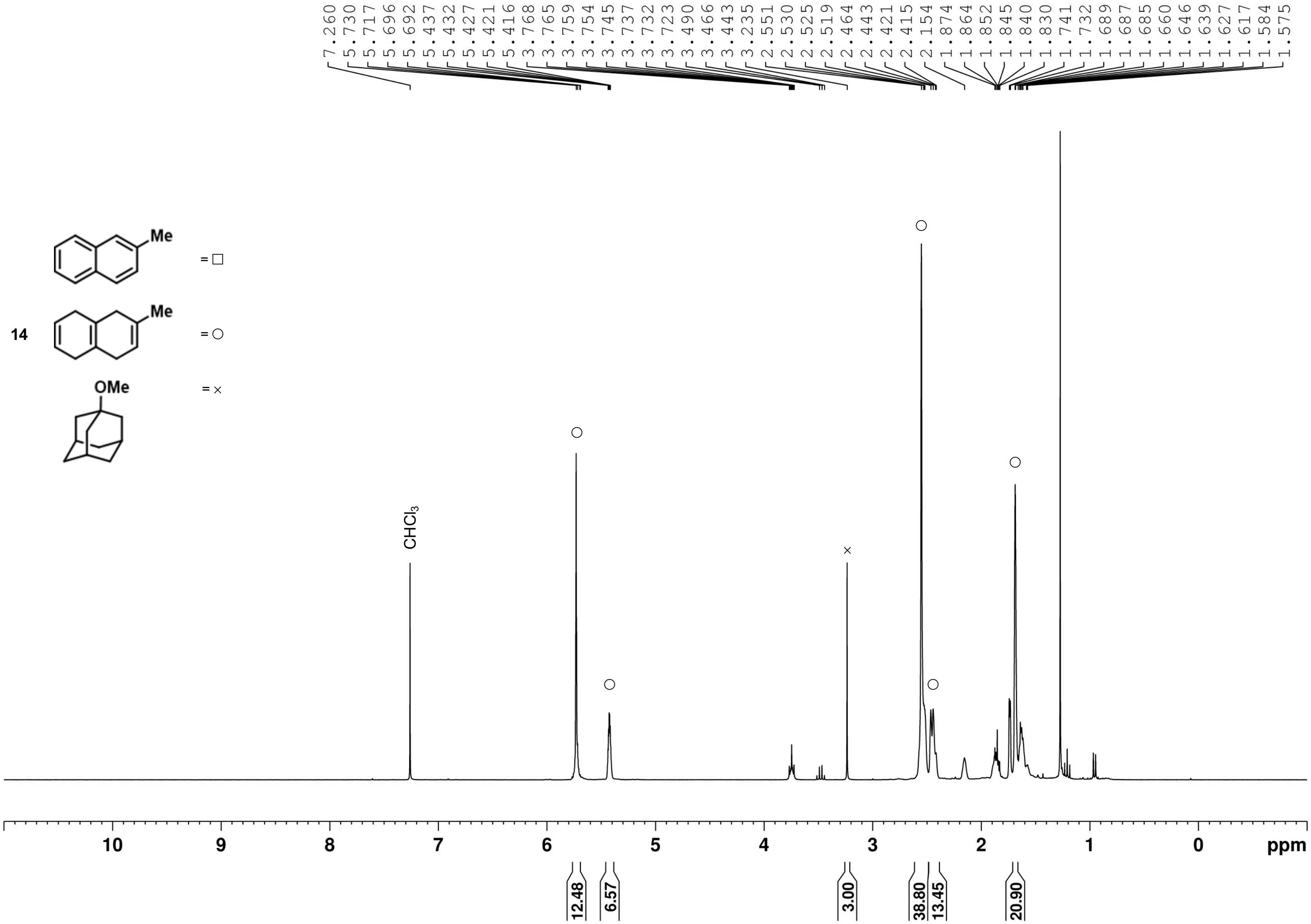

Spectrum S50. ^{1}H NMR spectrum of Table S5, entry 5 (300 MHz, $CDCl_3$, 298 K).

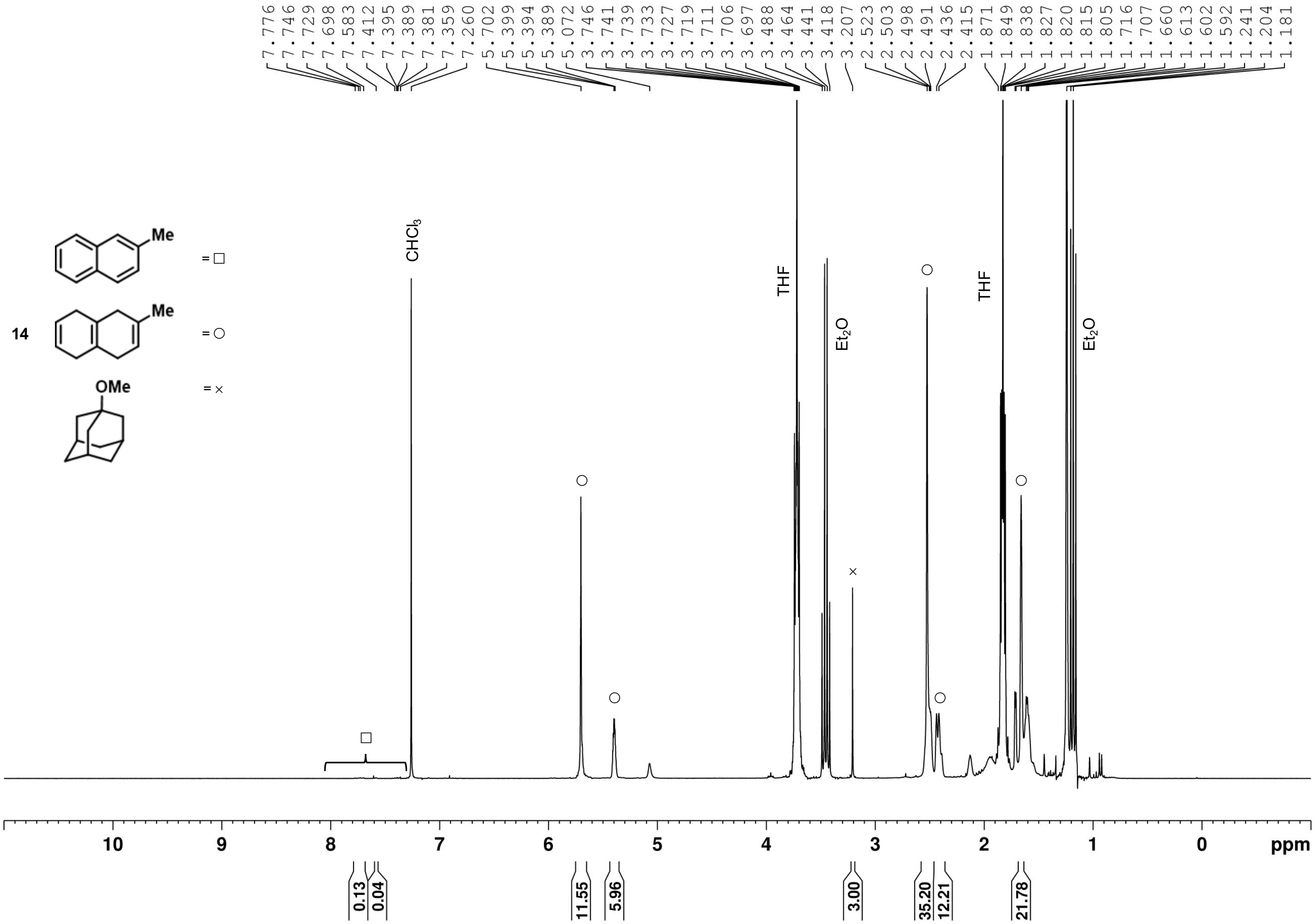

Spectrum S51. ^{1}H NMR spectrum of Table S5, entry 6 (300 MHz, $CDCl_3$, 298 K).

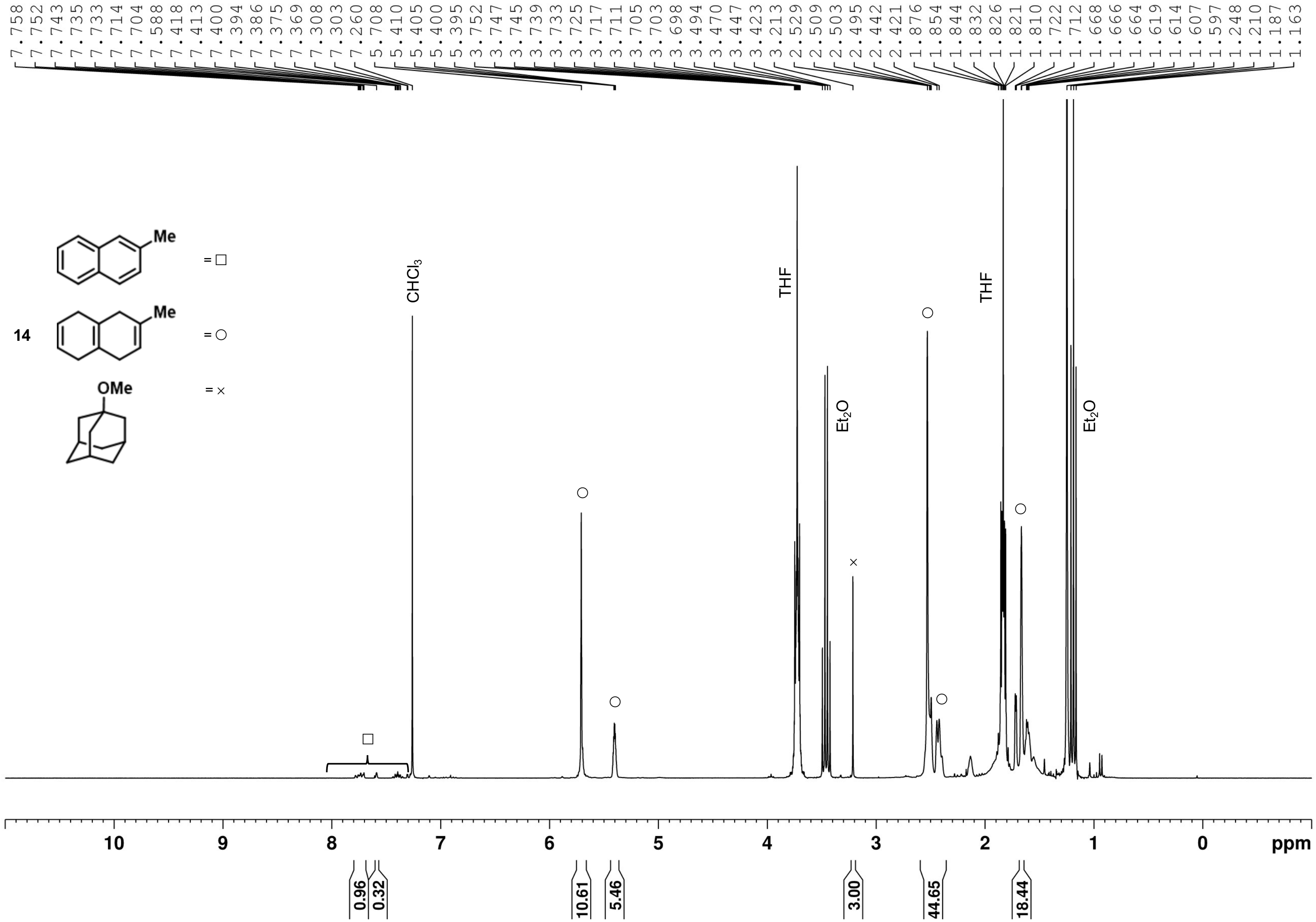

Spectrum S52. ^{1}H NMR spectrum of Table S6, entry 1 (400 MHz, $CDCl_3$, 298 K).

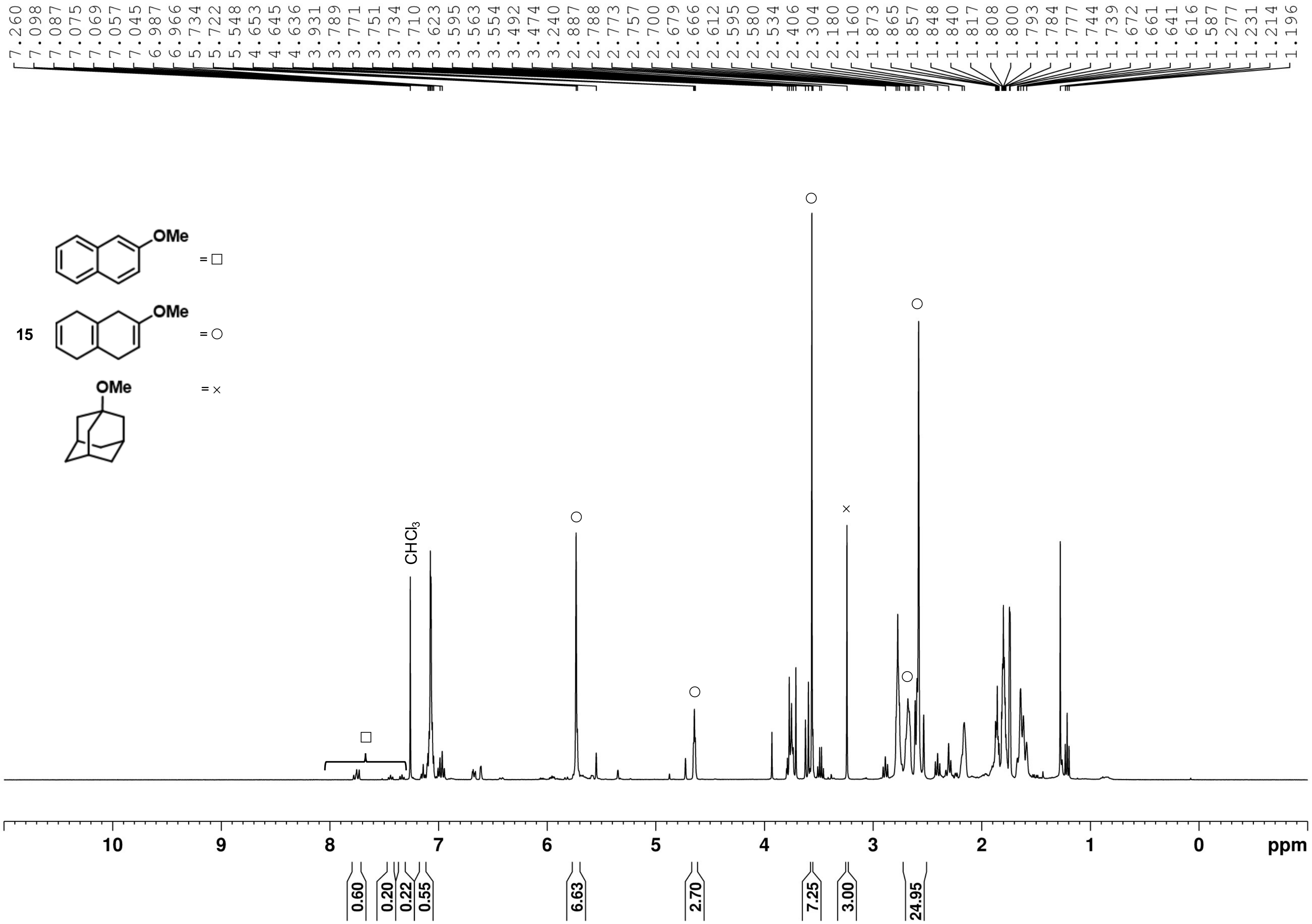

Spectrum S53. ^1H NMR spectrum of Table S6, entry 2 (400 MHz, $CDCl_3$, 298 K).

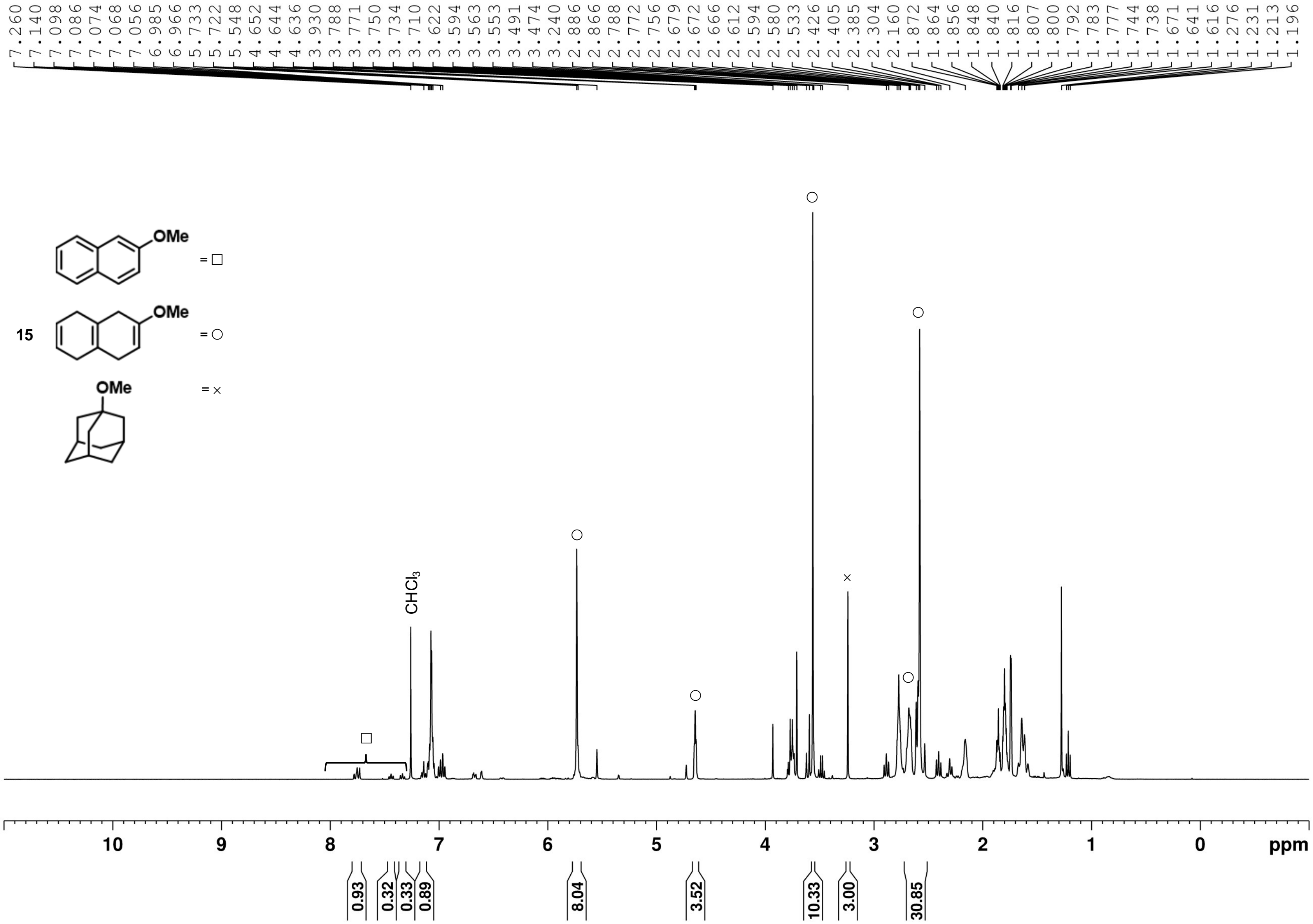

Spectrum S54. ^{1}H NMR spectrum of Table S6, entry 3 (300 MHz, $CDCl_3$, 298 K).

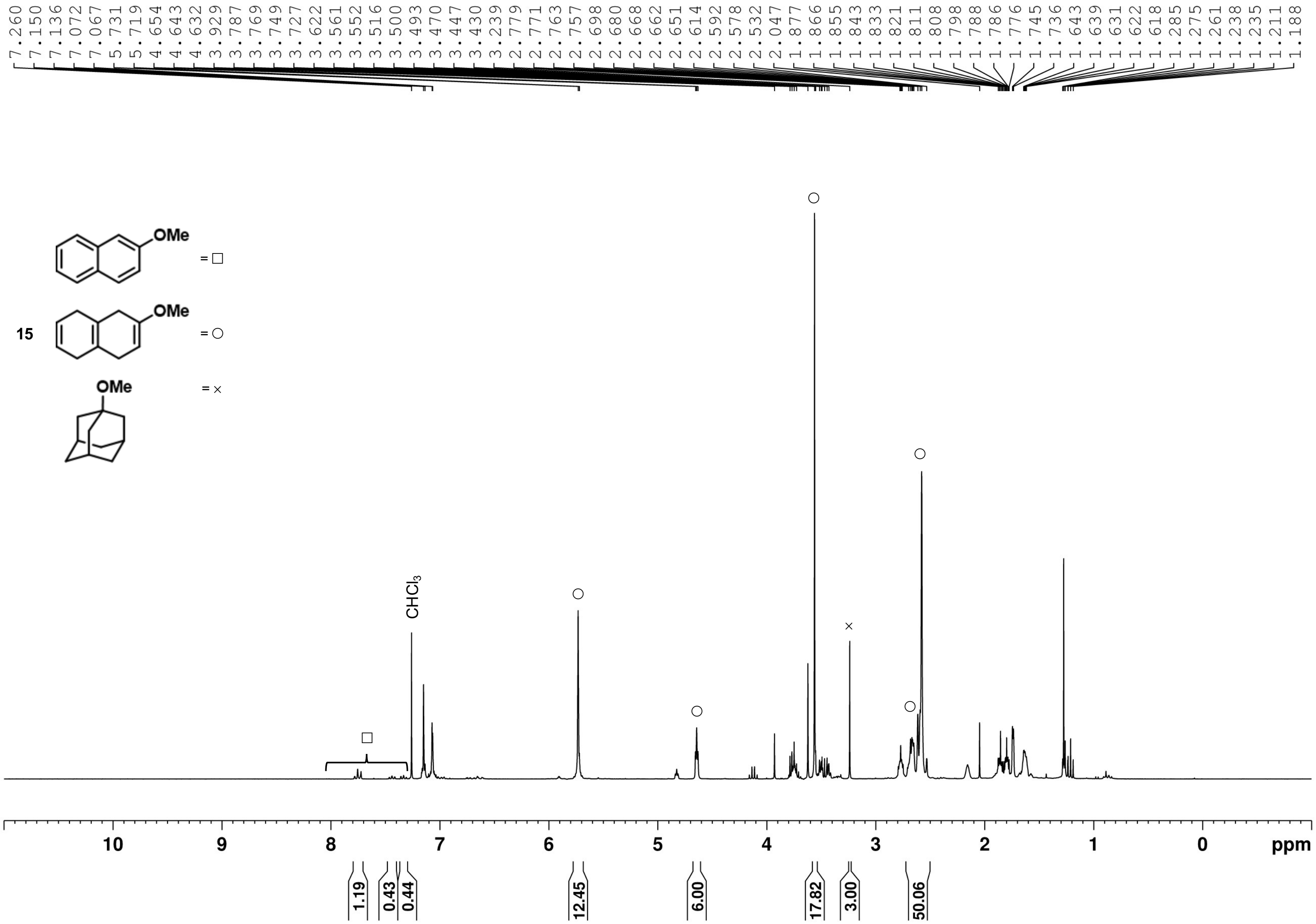

Spectrum S55. ^{1}H NMR spectrum of Table S6, entry 4 (300 MHz, $CDCl_3$, 298 K).

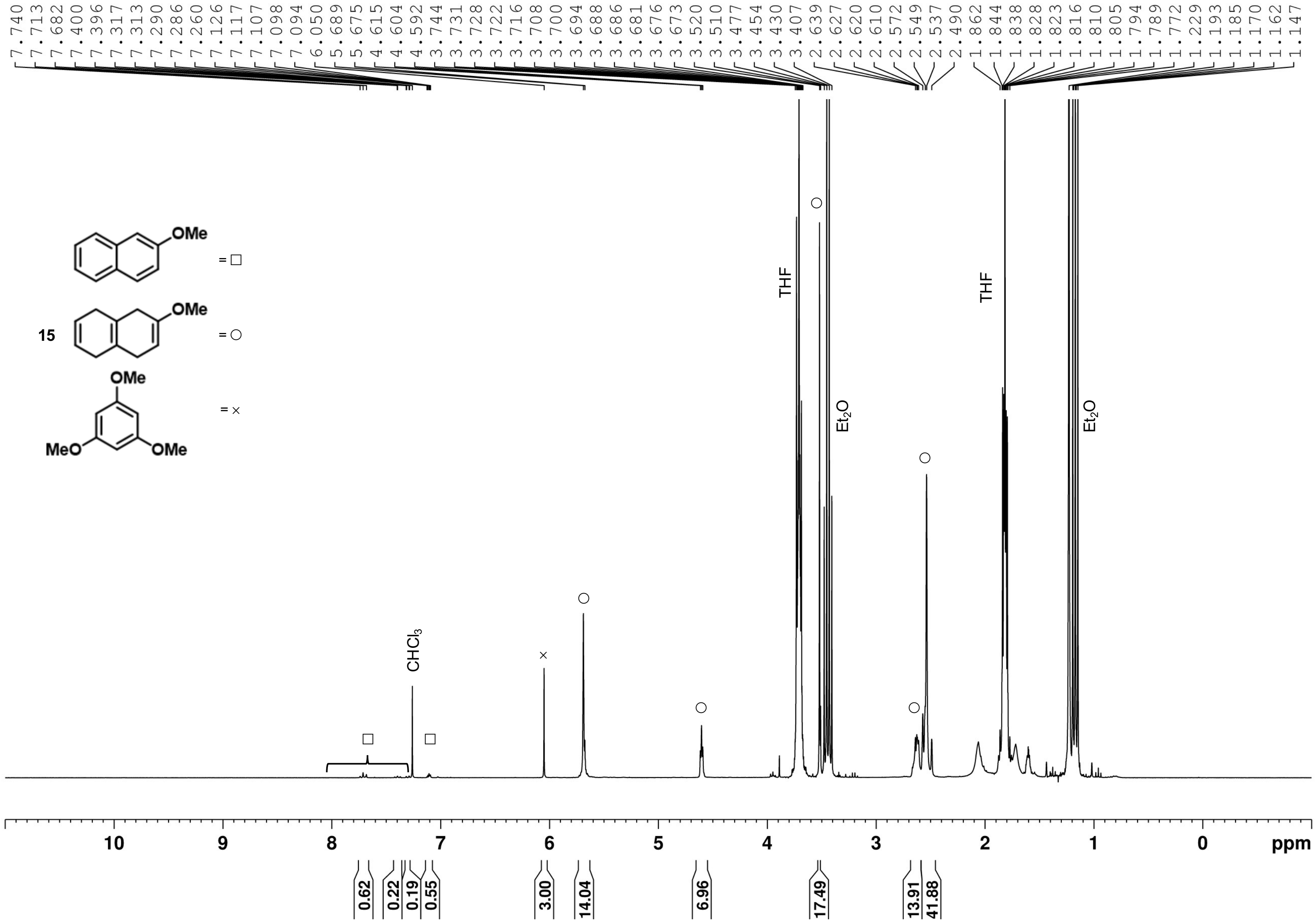

Spectrum S56. ^{1}H NMR spectrum of Table S6, entry 5 (300 MHz, $CDCl_3$, 298 K).

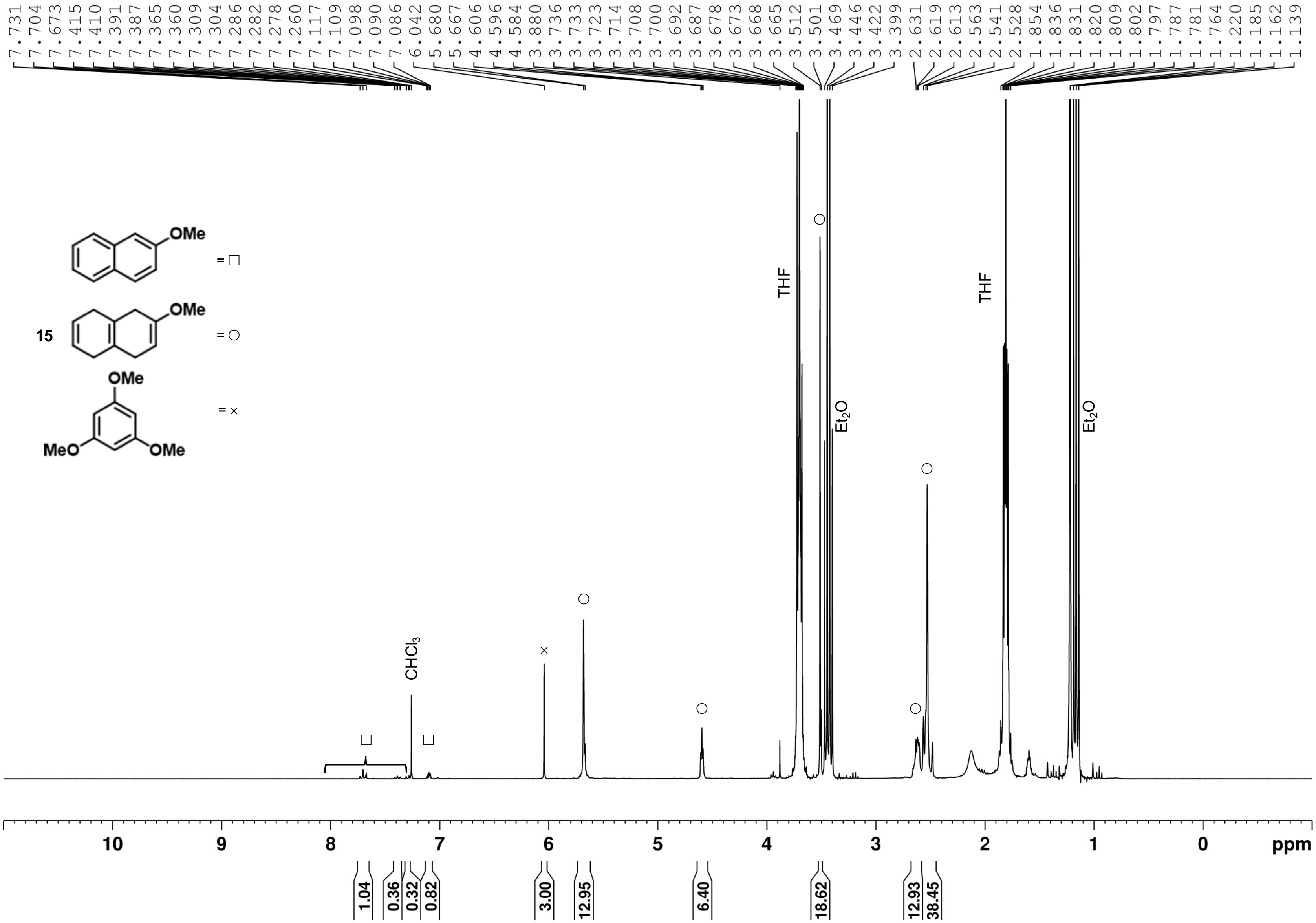

Spectrum S57. ^{1}H NMR spectrum of Table S6, entry 6 (300 MHz, $CDCl_3$, 298 K).

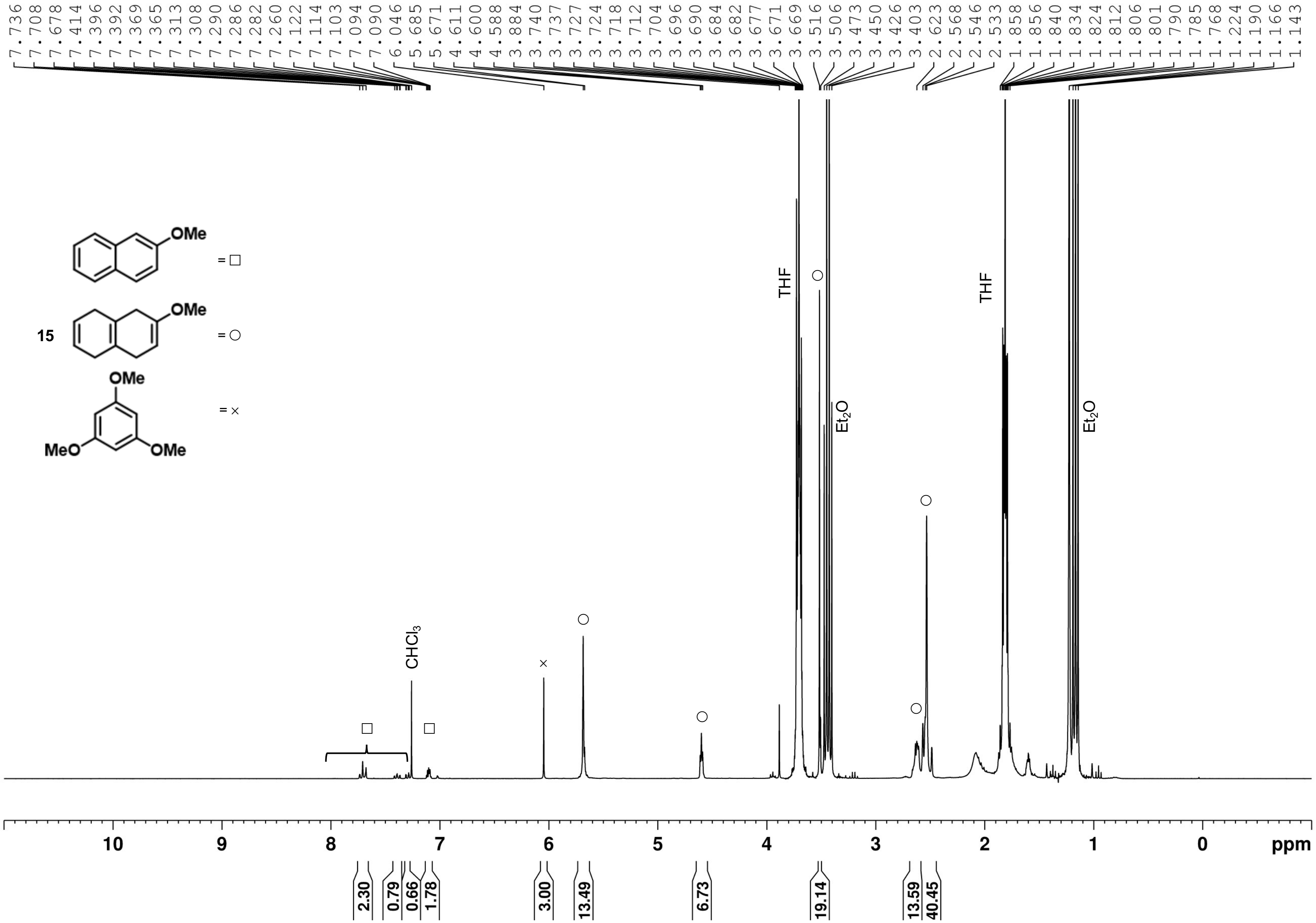

Spectrum S58. ^{1}H NMR spectrum of Table S7, entry 1 (300 MHz, $CDCl_3$, 298 K).

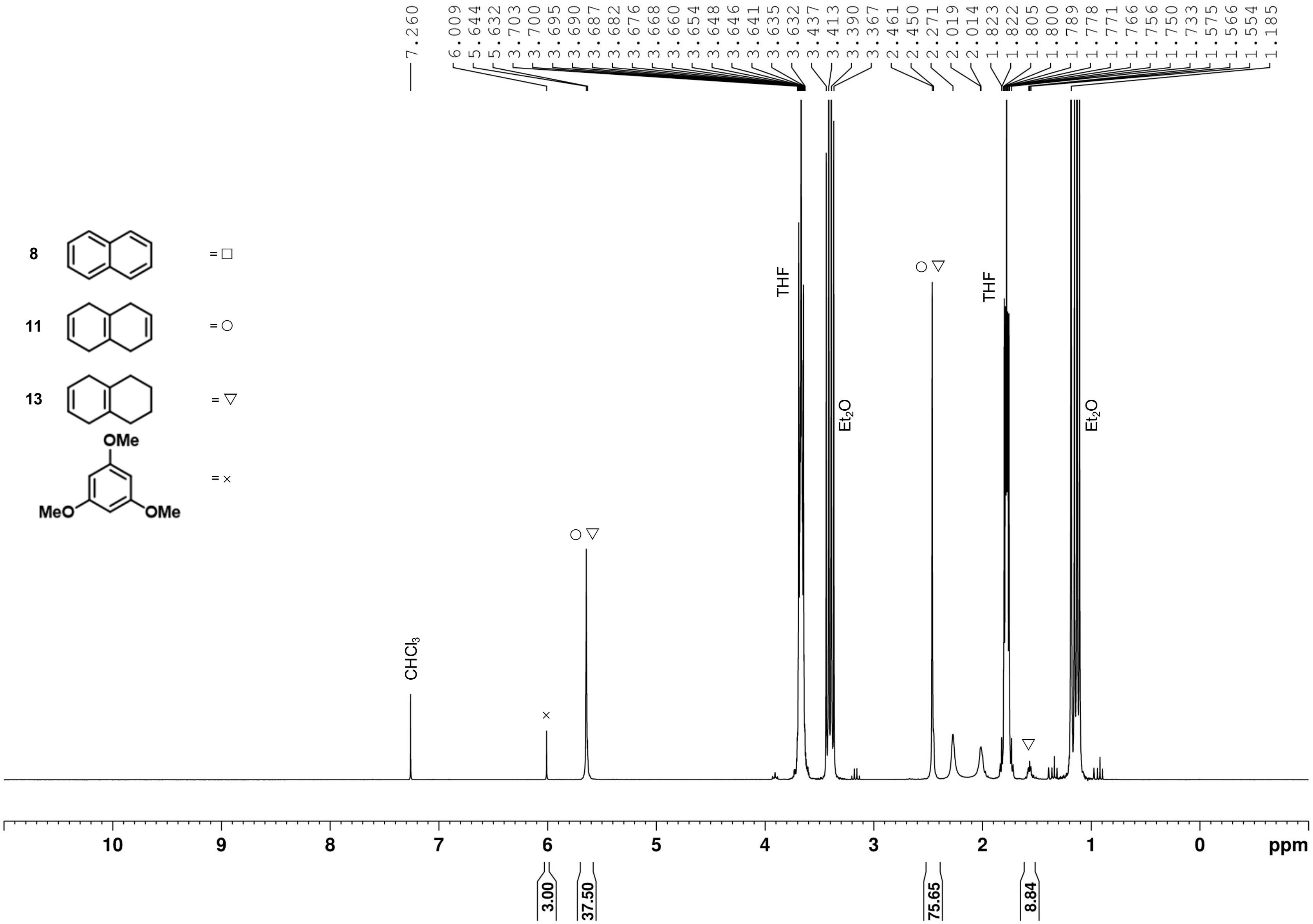

Spectrum S59. ^{1}H NMR spectrum of Table S7, entry 2 (300 MHz, $CDCl_3$, 298 K).

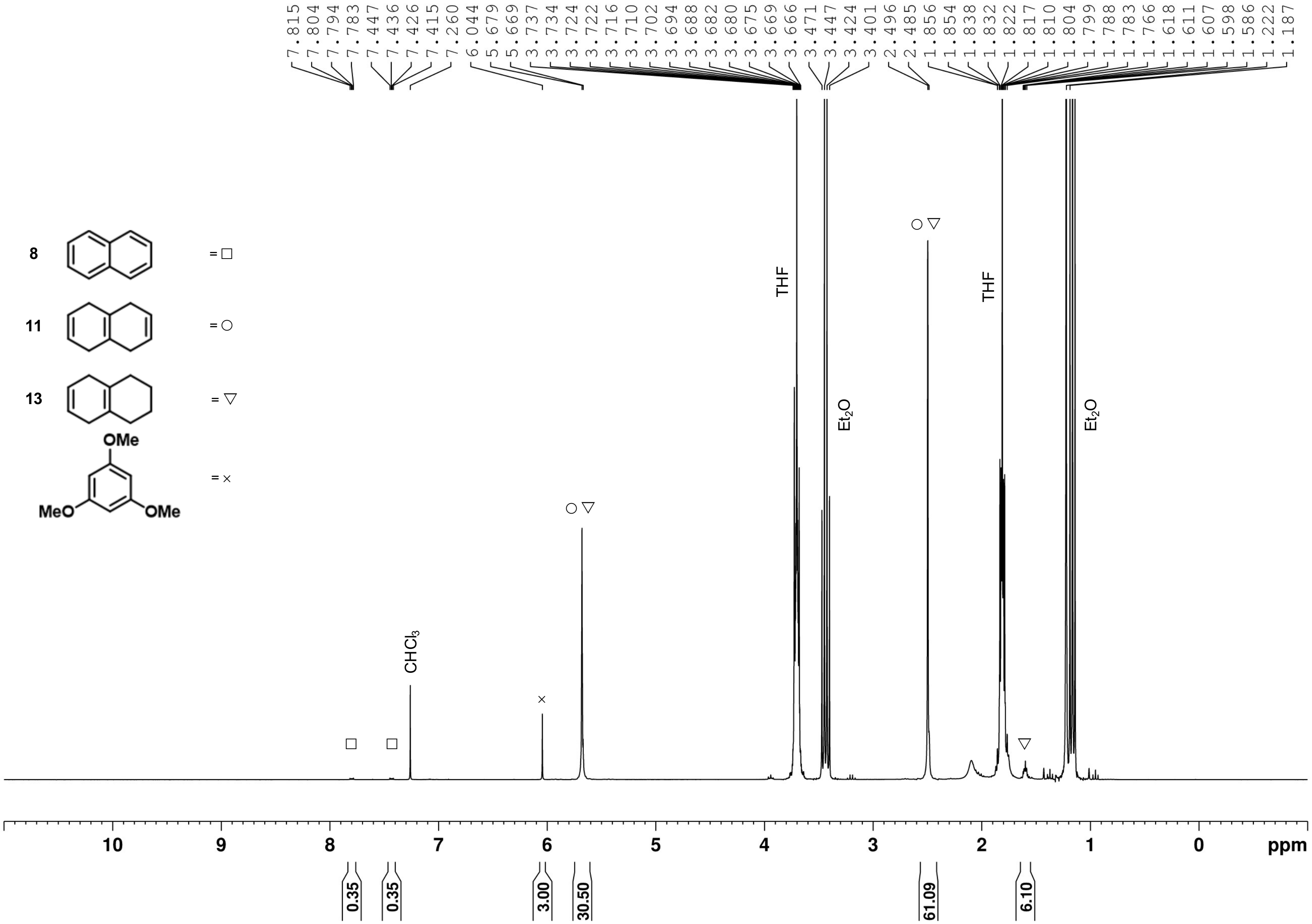

Spectrum S60. ^{1}H NMR spectrum of Table S7, entry 3 (300 MHz, $CDCl_3$, 298 K).

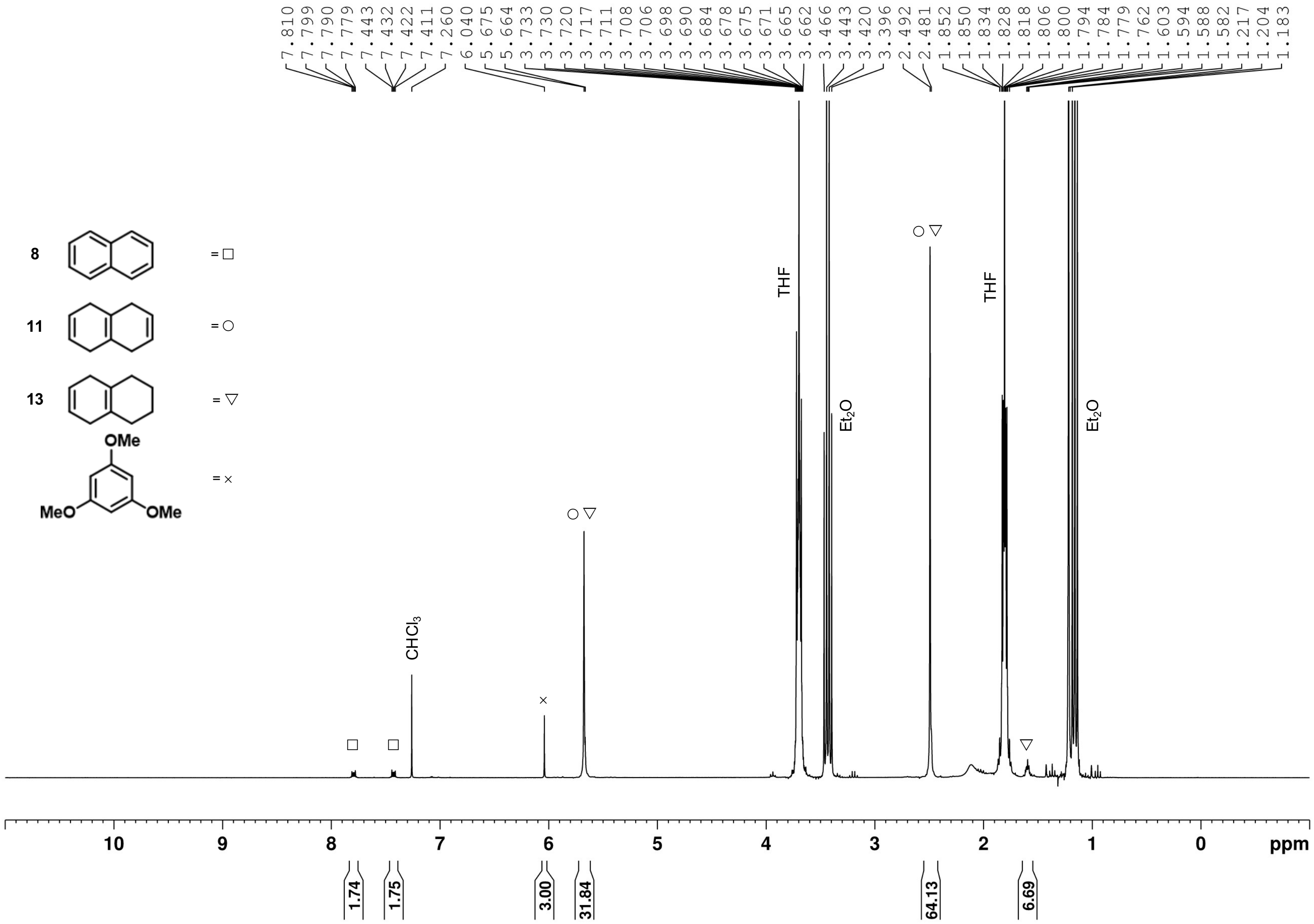

Spectrum S61. ^{1}H NMR spectrum of 2-(1,4,5,8-tetrahydronaphthalen-2-yl)acetic acid (**16**) (300 MHz, $CDCl_3$, 298 K).

16

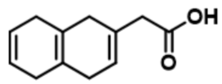

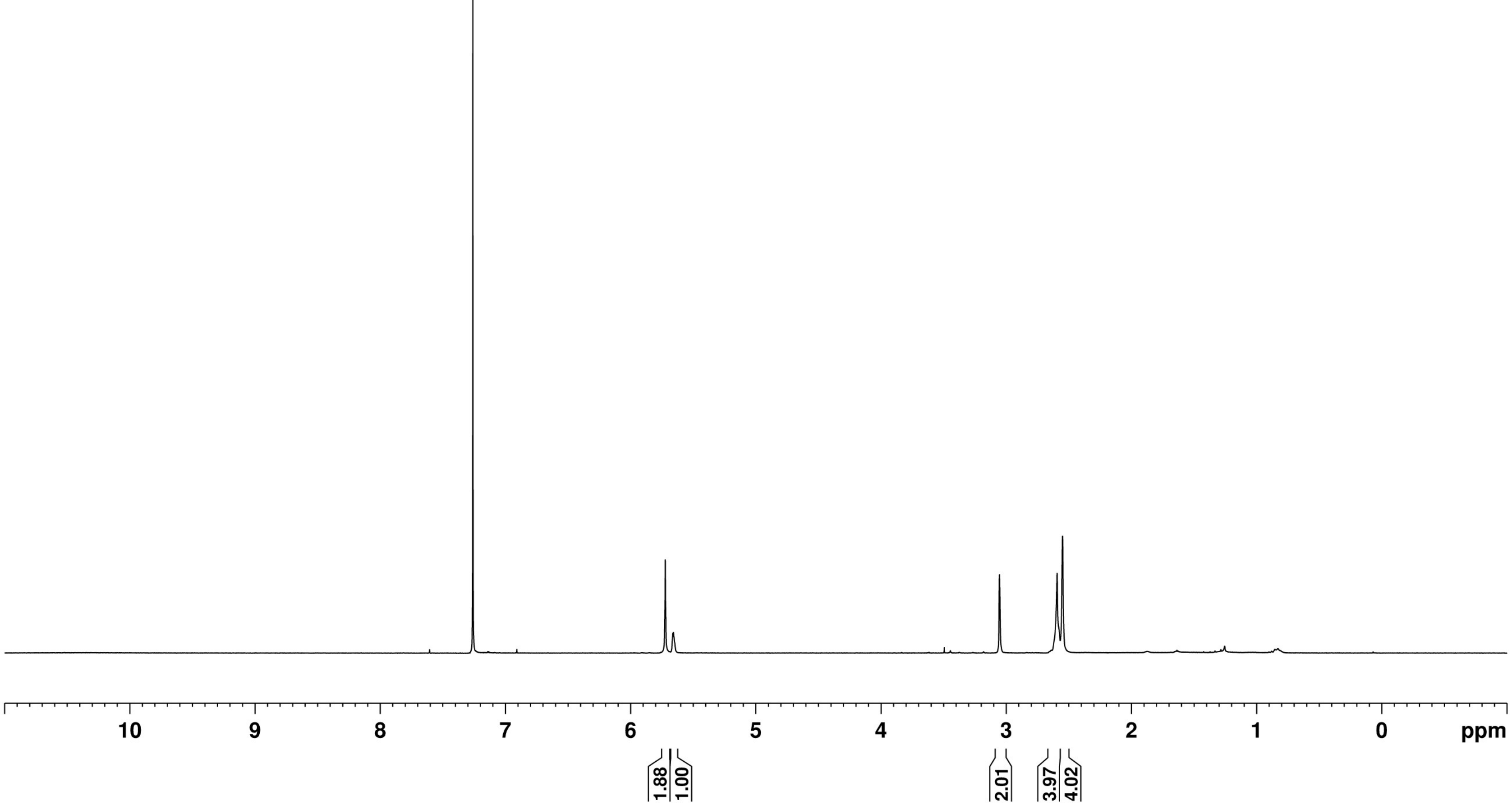

Spectrum S62. ^{13}C NMR spectrum of 2-(1,4,5,8-tetrahydronaphthalen-2-yl)acetic acid (**16**) (300 MHz, DMSO-d_6, 298 K).

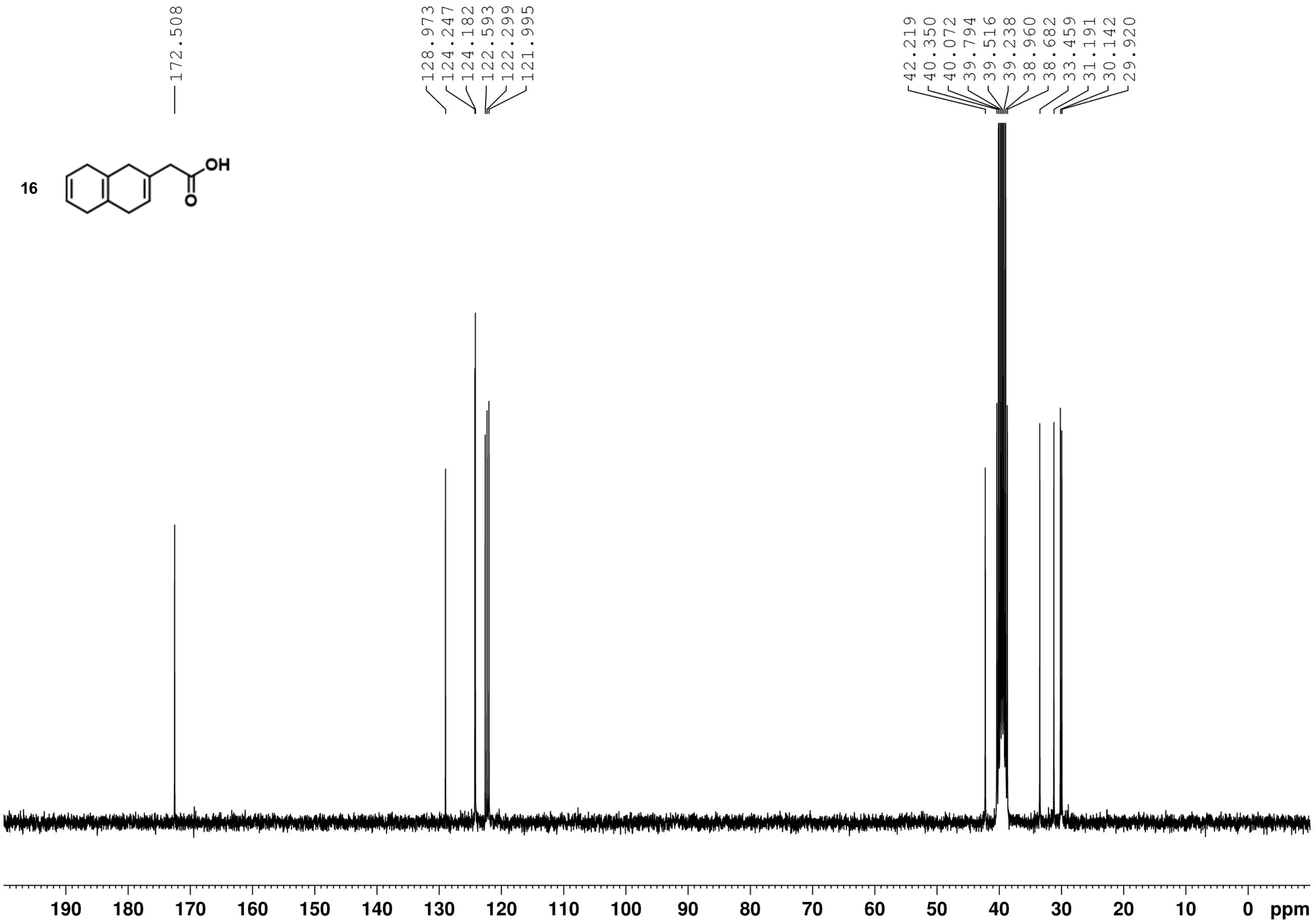

Spectrum S63. ^{1}H NMR spectrum of Table S8, entry 1 (300 MHz, $CDCl_3$, 298 K).

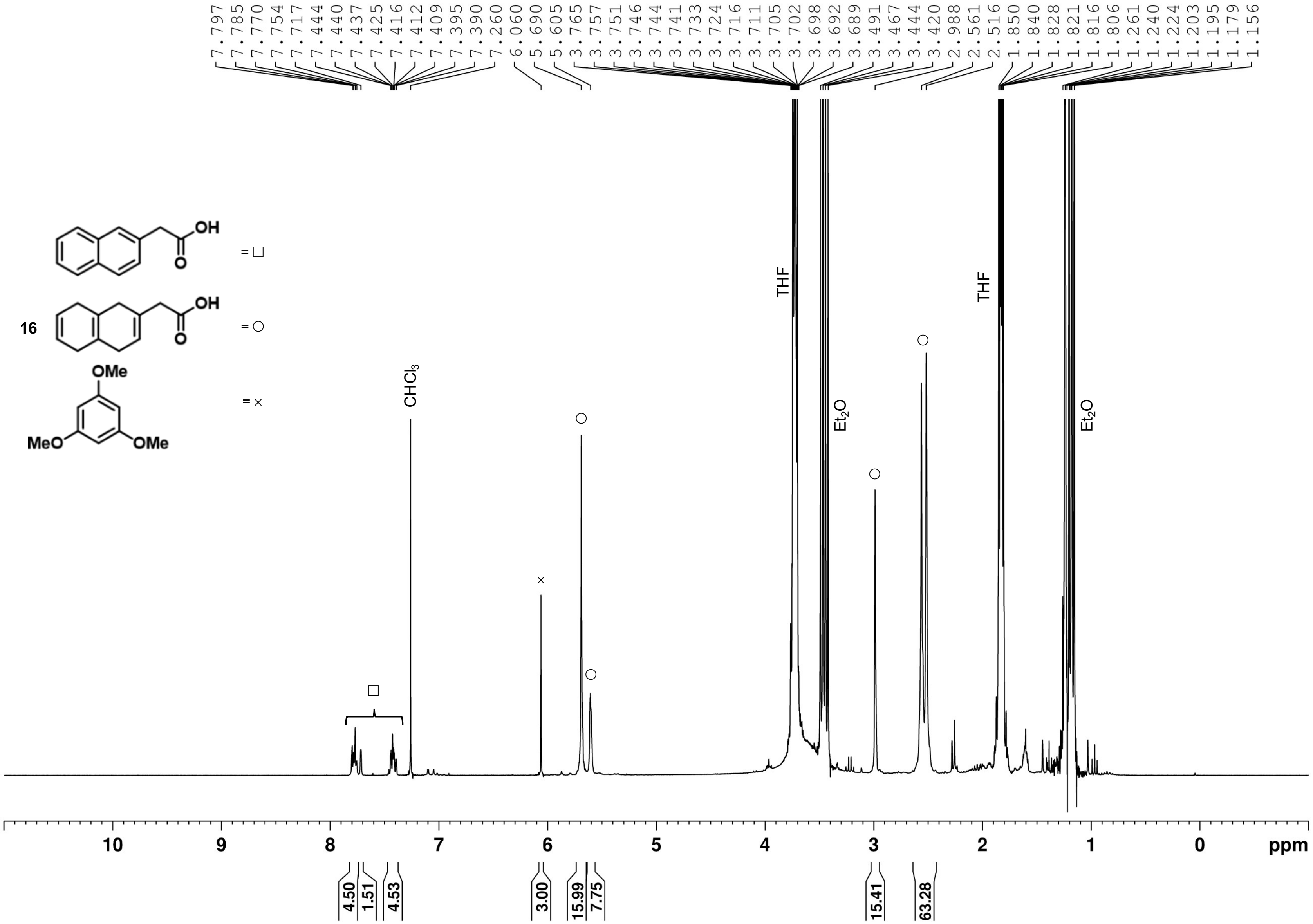

Spectrum S64. ^{1}H NMR spectrum of Table S8, entry 2 (300 MHz, $CDCl_3$, 298 K).

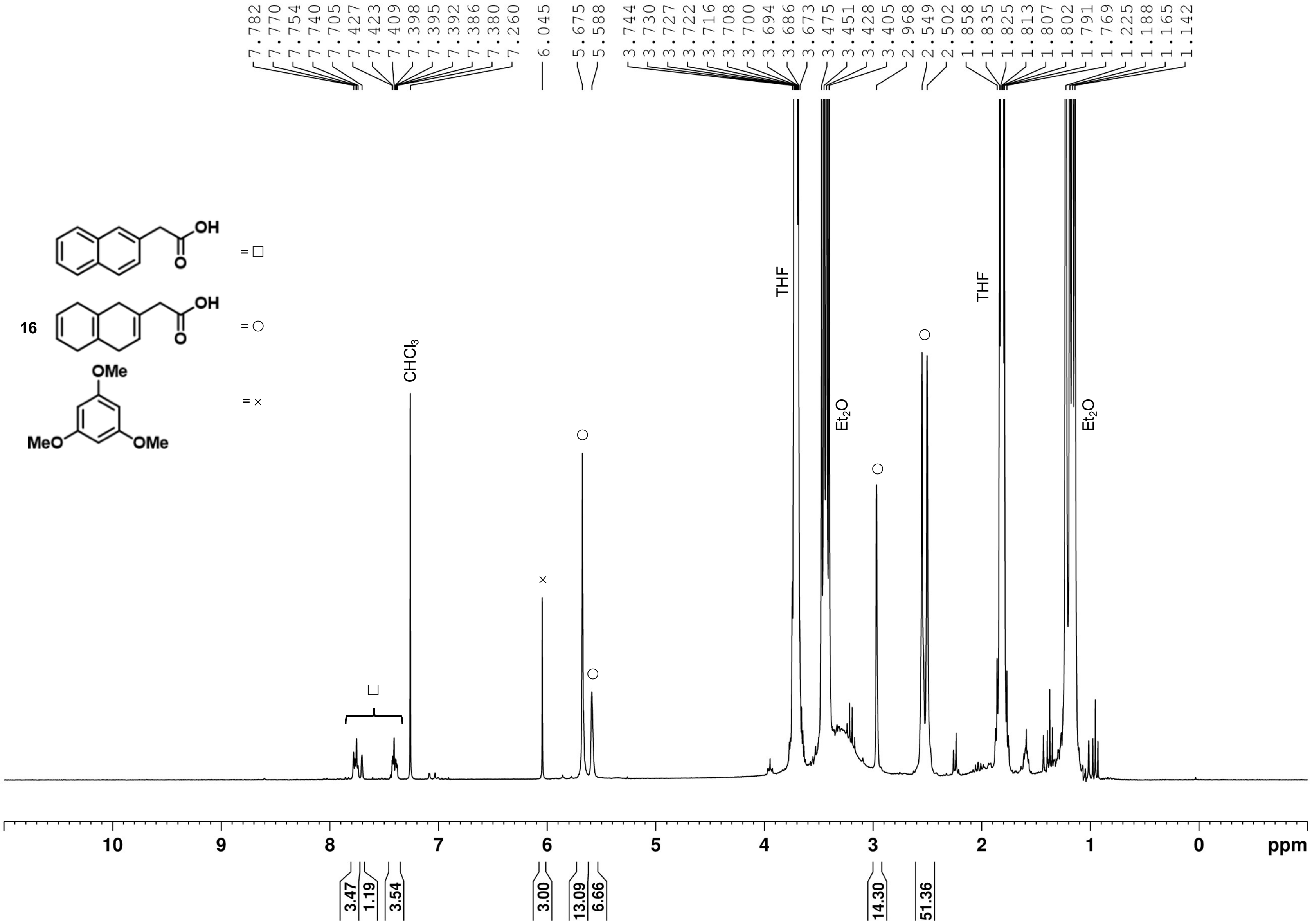

Spectrum S65. ^{1}H NMR spectrum of Table S8, entry 3 (300 MHz, $CDCl_3$, 298 K).

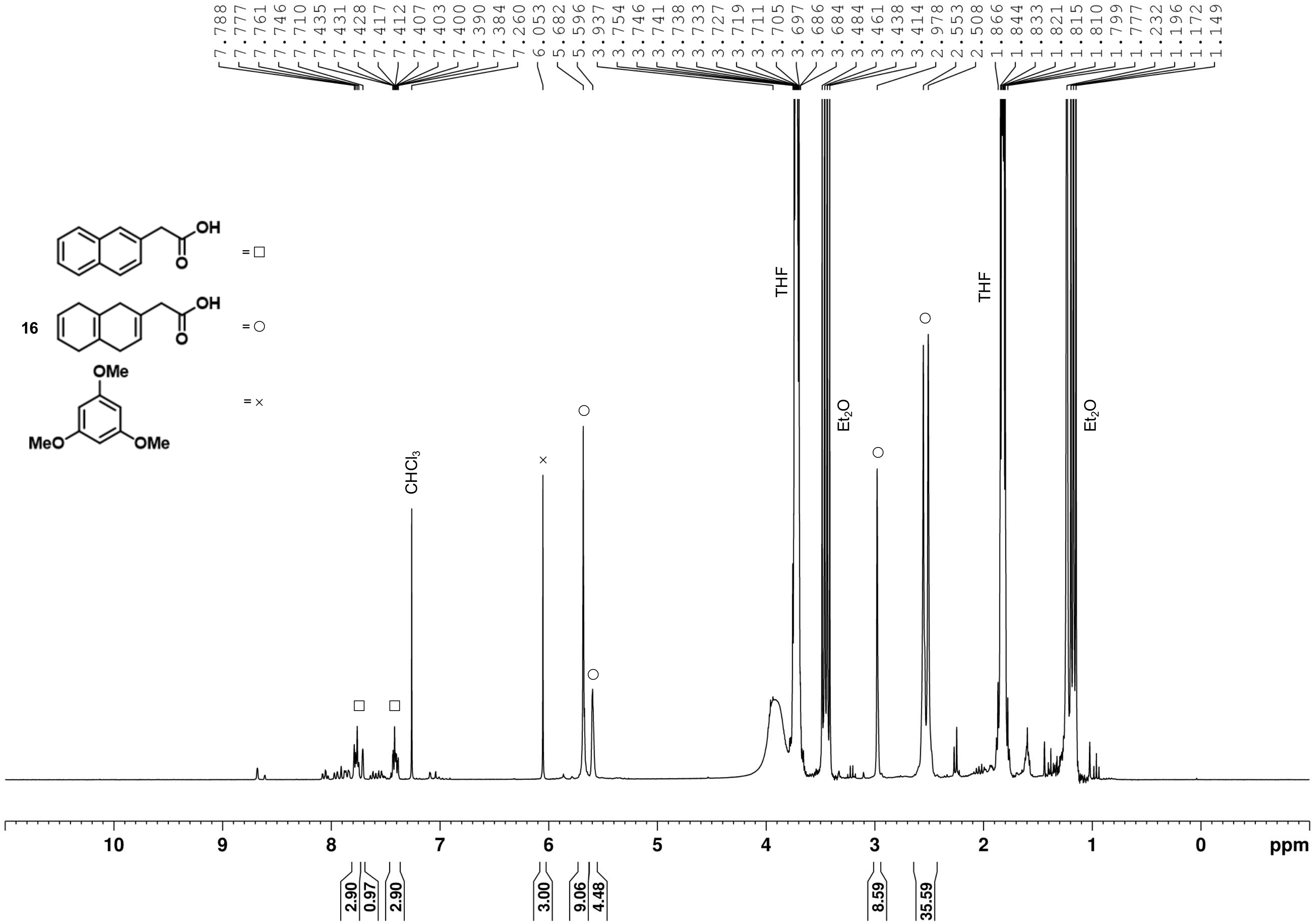

Spectrum S66. ^{1}H NMR spectrum of Table S9, entry 1 (300 MHz, $CDCl_3$, 298 K).

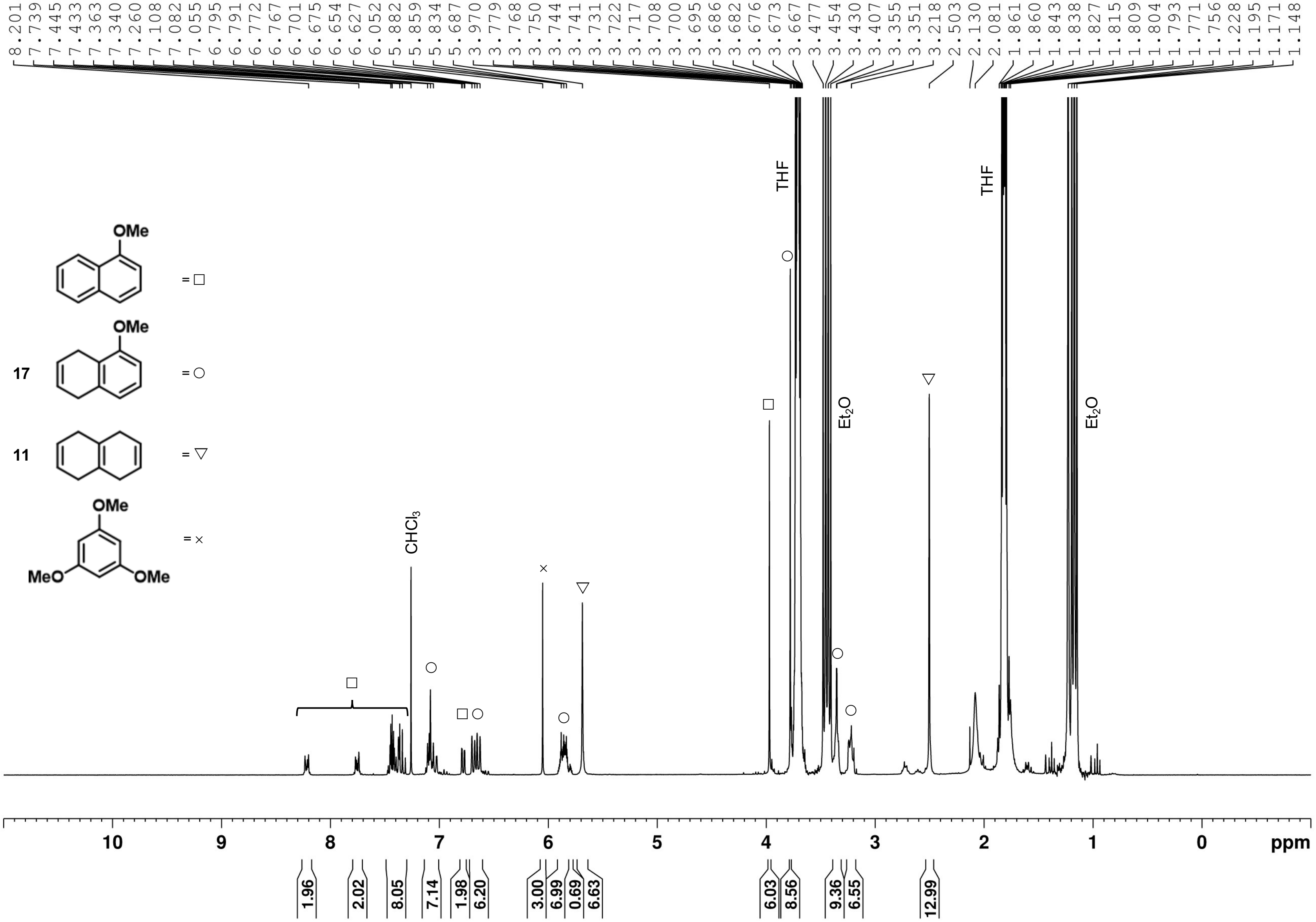

Spectrum S67. ^{1}H NMR spectrum of Table S9, entry 2 (300 MHz, $CDCl_3$, 298 K).

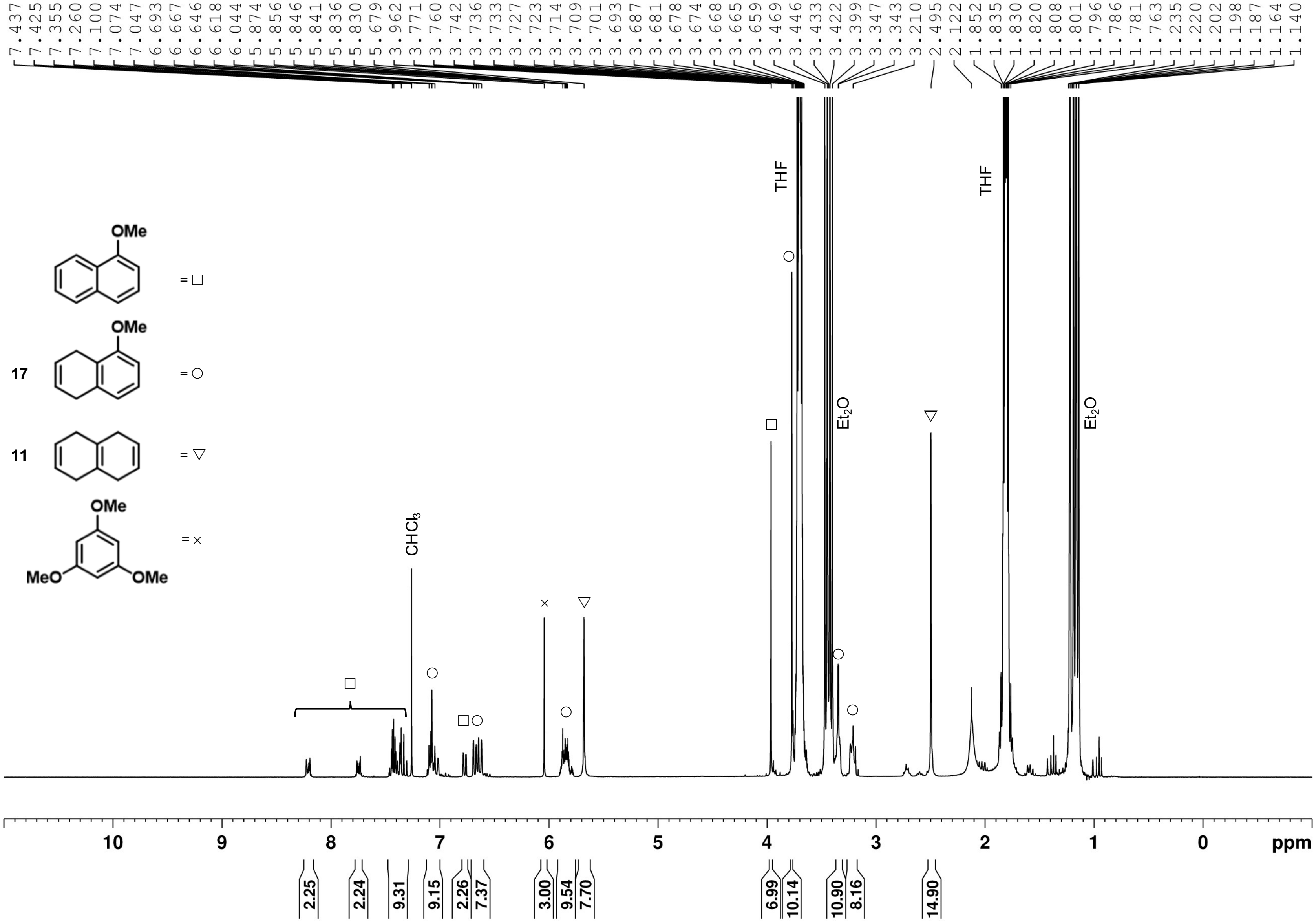

Spectrum S68. ^{1}H NMR spectrum of Table S9, entry 3 (300 MHz, $CDCl_3$, 298 K).

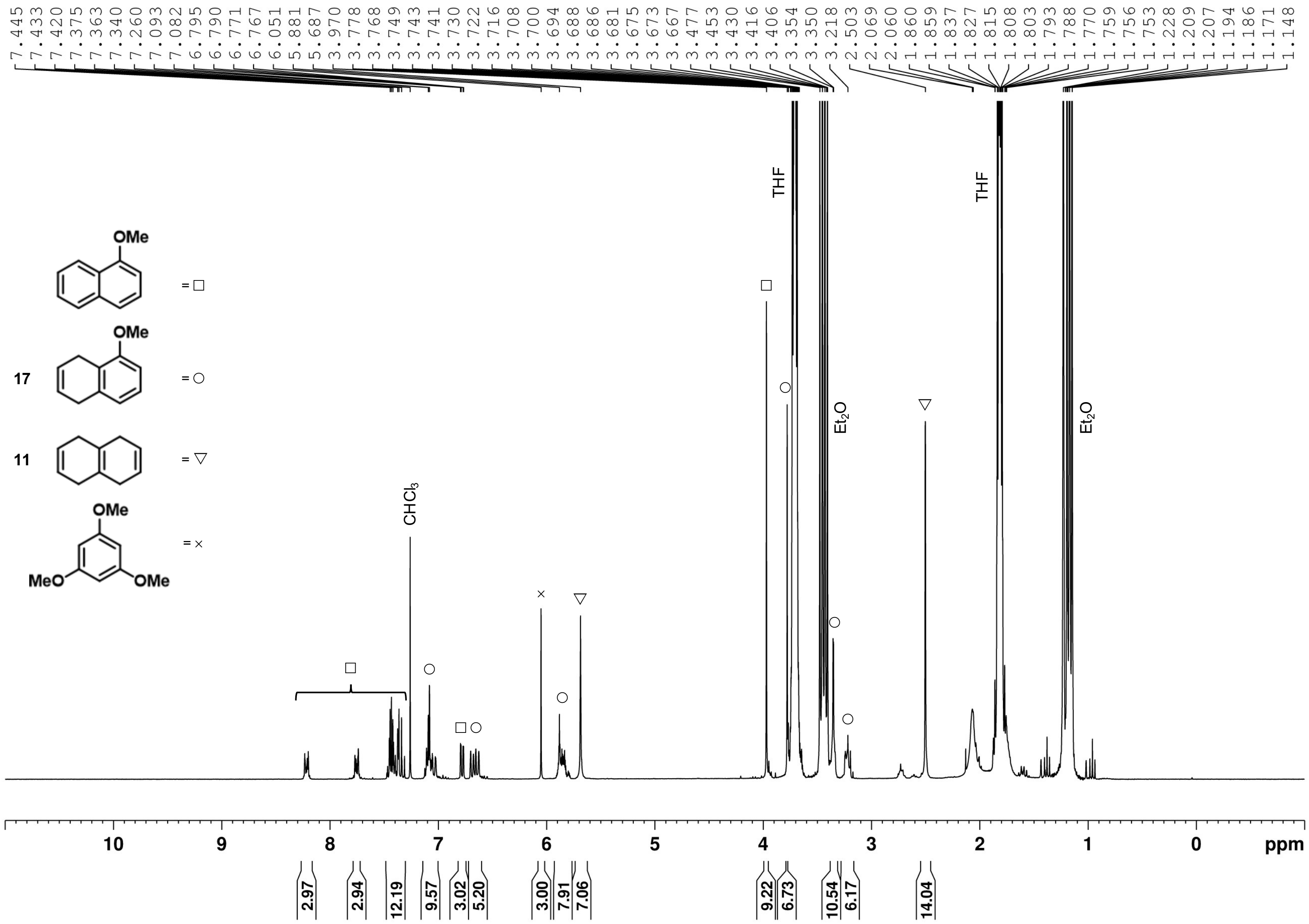

Spectrum S69. ^{1}H NMR spectrum of Table S10, entry 1 (300 MHz, $CDCl_3$, 298 K).

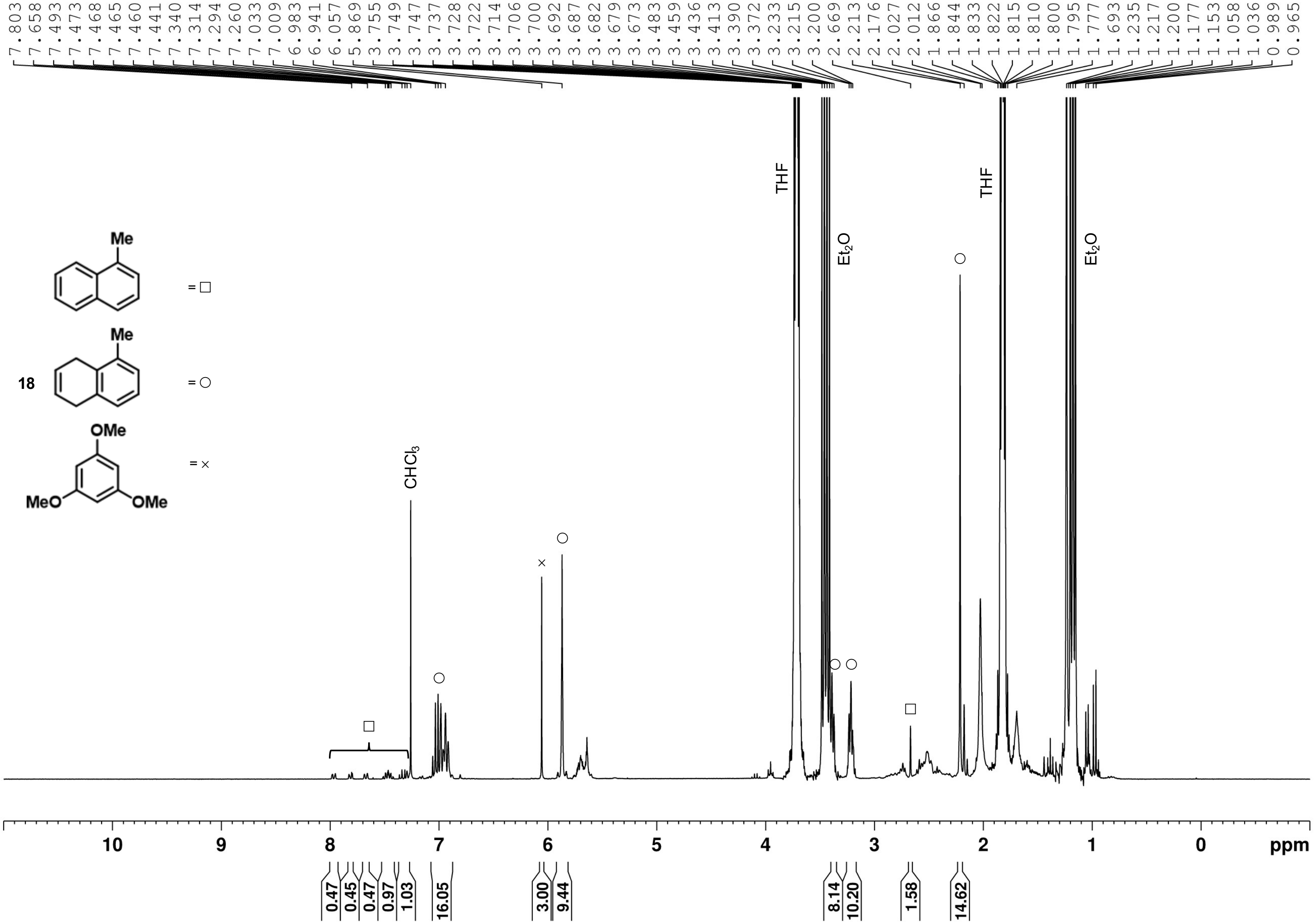

Spectrum S70. ^1H NMR spectrum of Table S10, entry 2 (300 MHz, $CDCl_3$, 298 K).

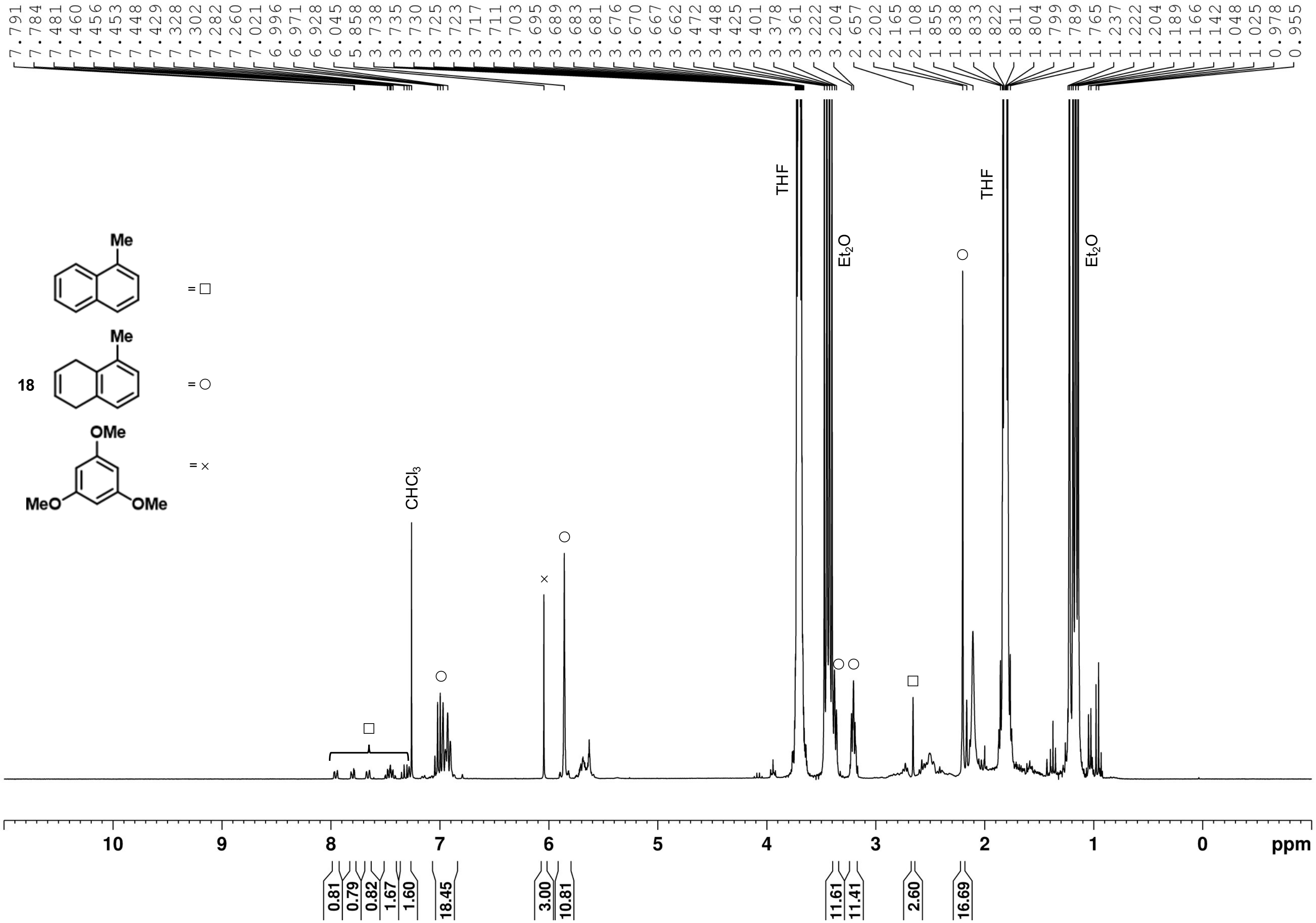

Spectrum S71. ^{1}H NMR spectrum of Table S10, entry 3 (300 MHz, $CDCl_3$, 298 K).

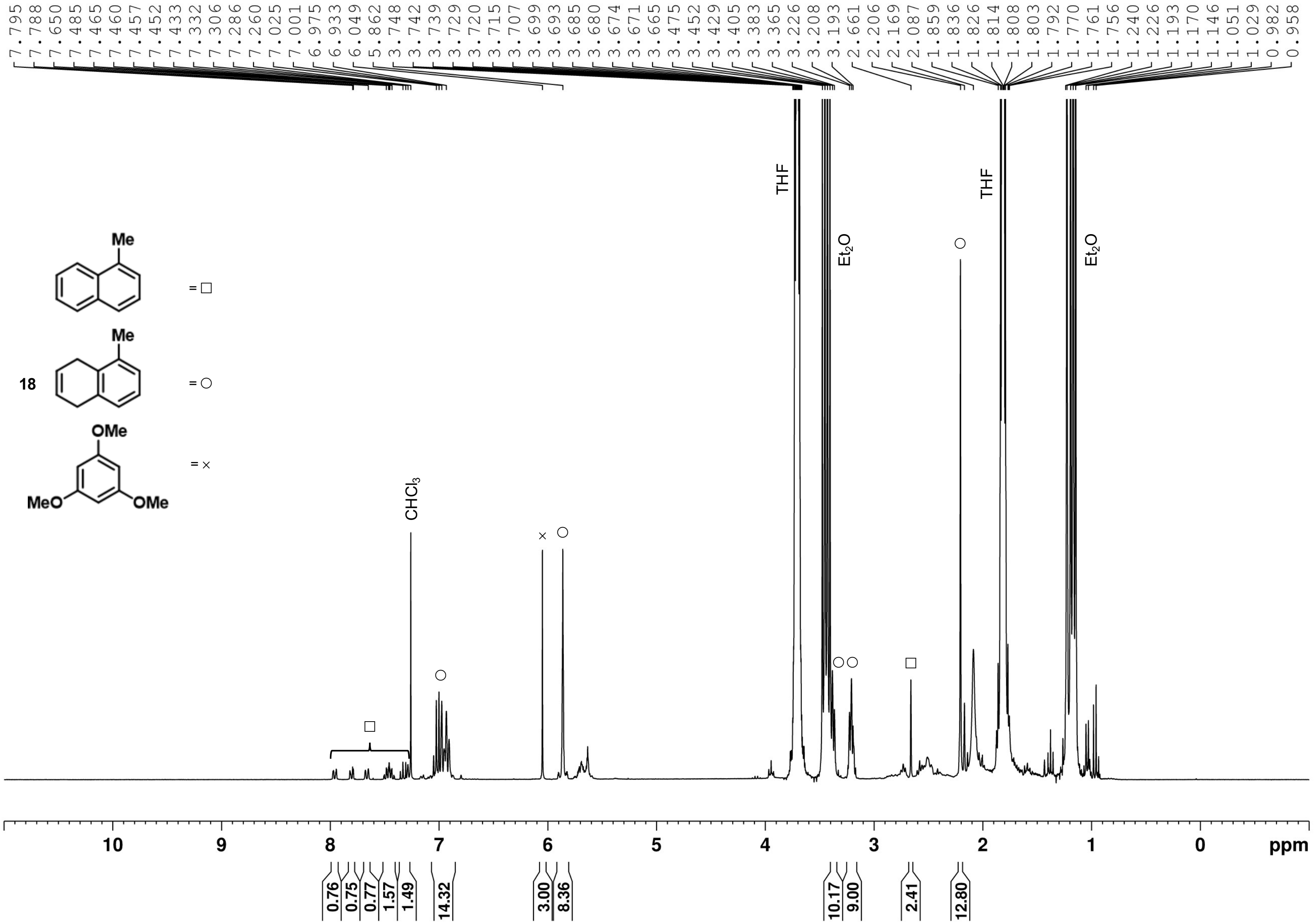

Spectrum S72. ^1H NMR spectrum of Table S11, entry 1 (300 MHz, $CDCl_3$, 298 K).

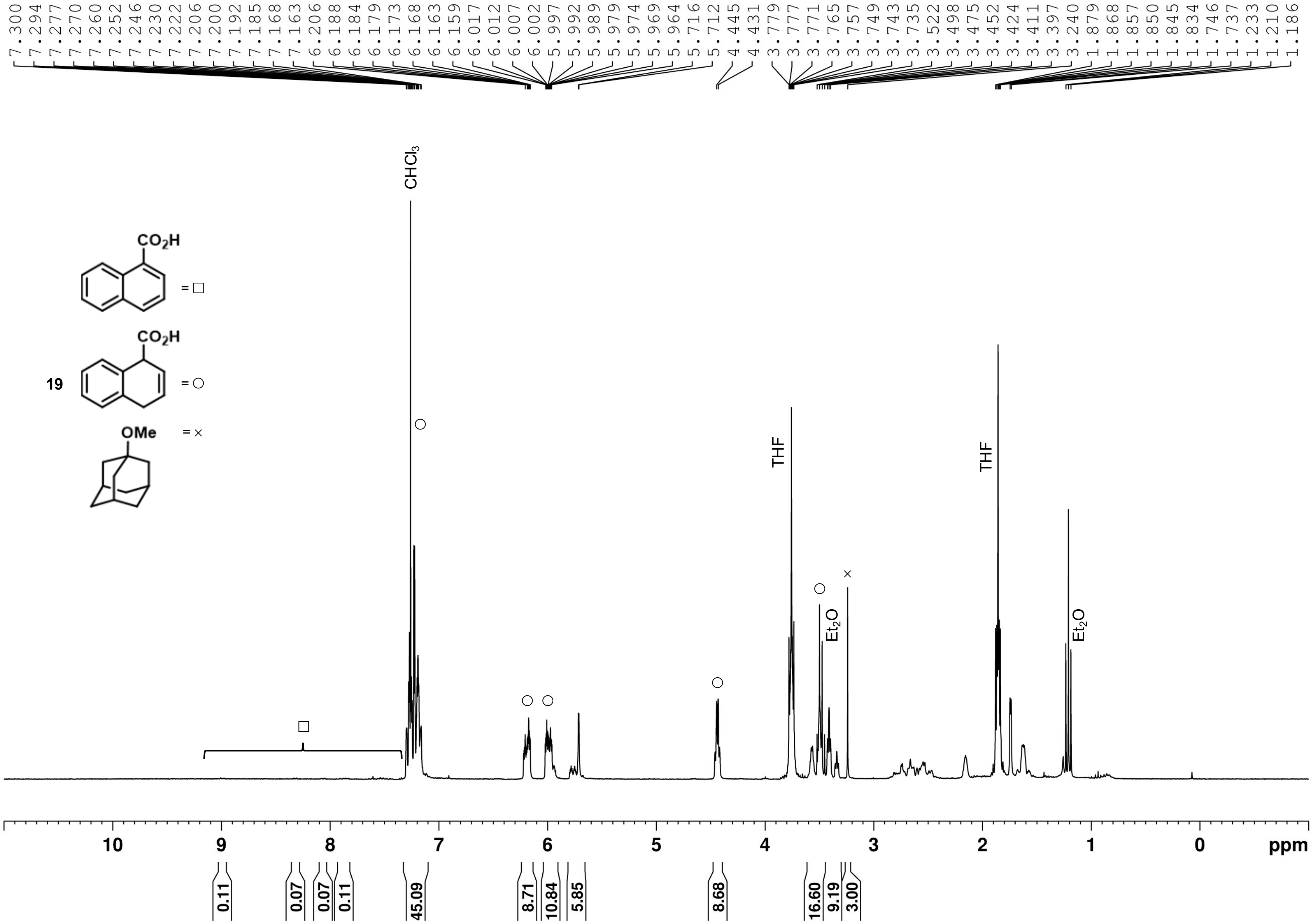

Spectrum S73. ^{1}H NMR spectrum of Table S11, entry 2 (300 MHz, $CDCl_3$, 298 K).

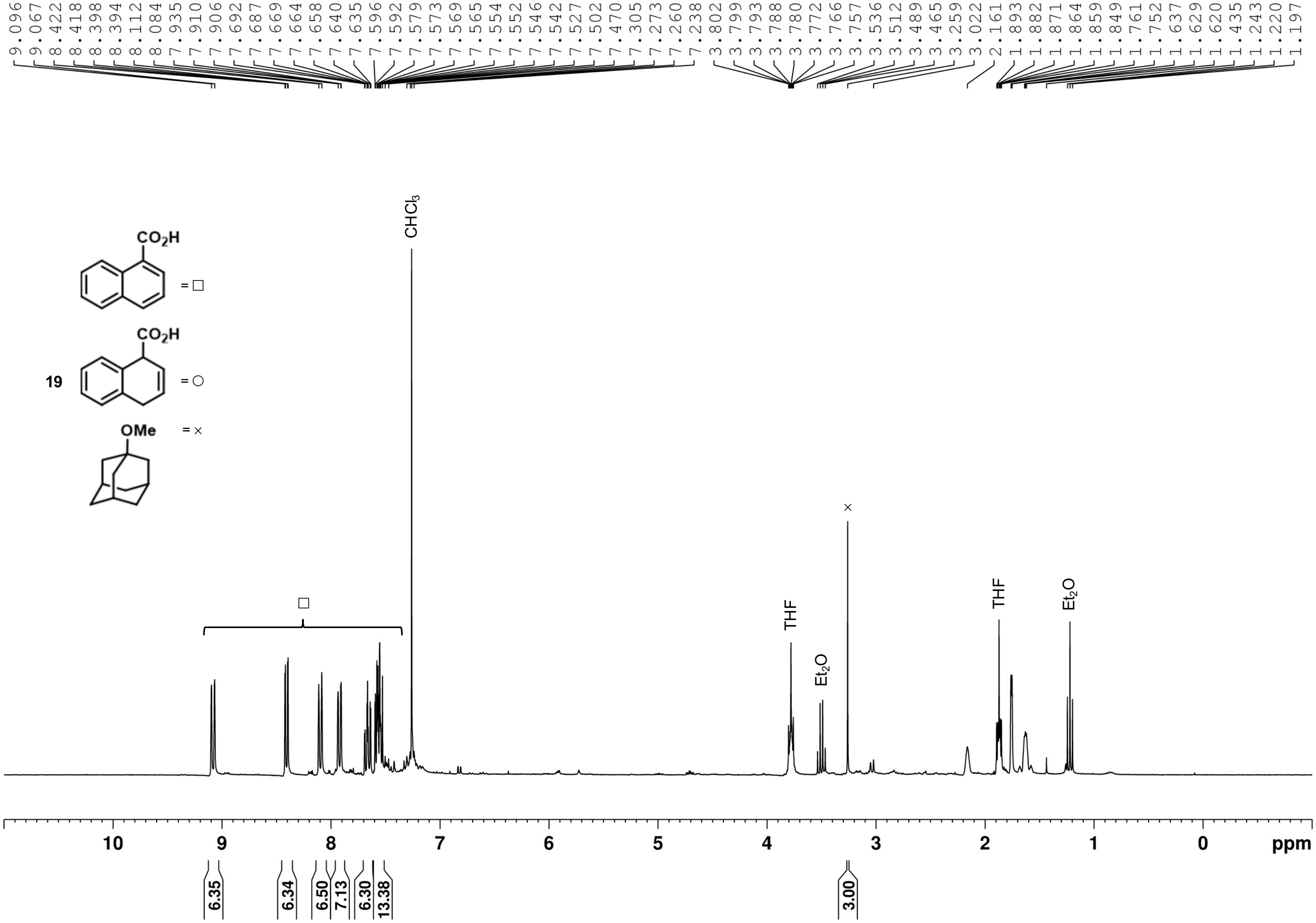

Spectrum S74. ^{1}H NMR spectrum of Table S11, entry 3 (300 MHz, $CDCl_3$, 298 K).

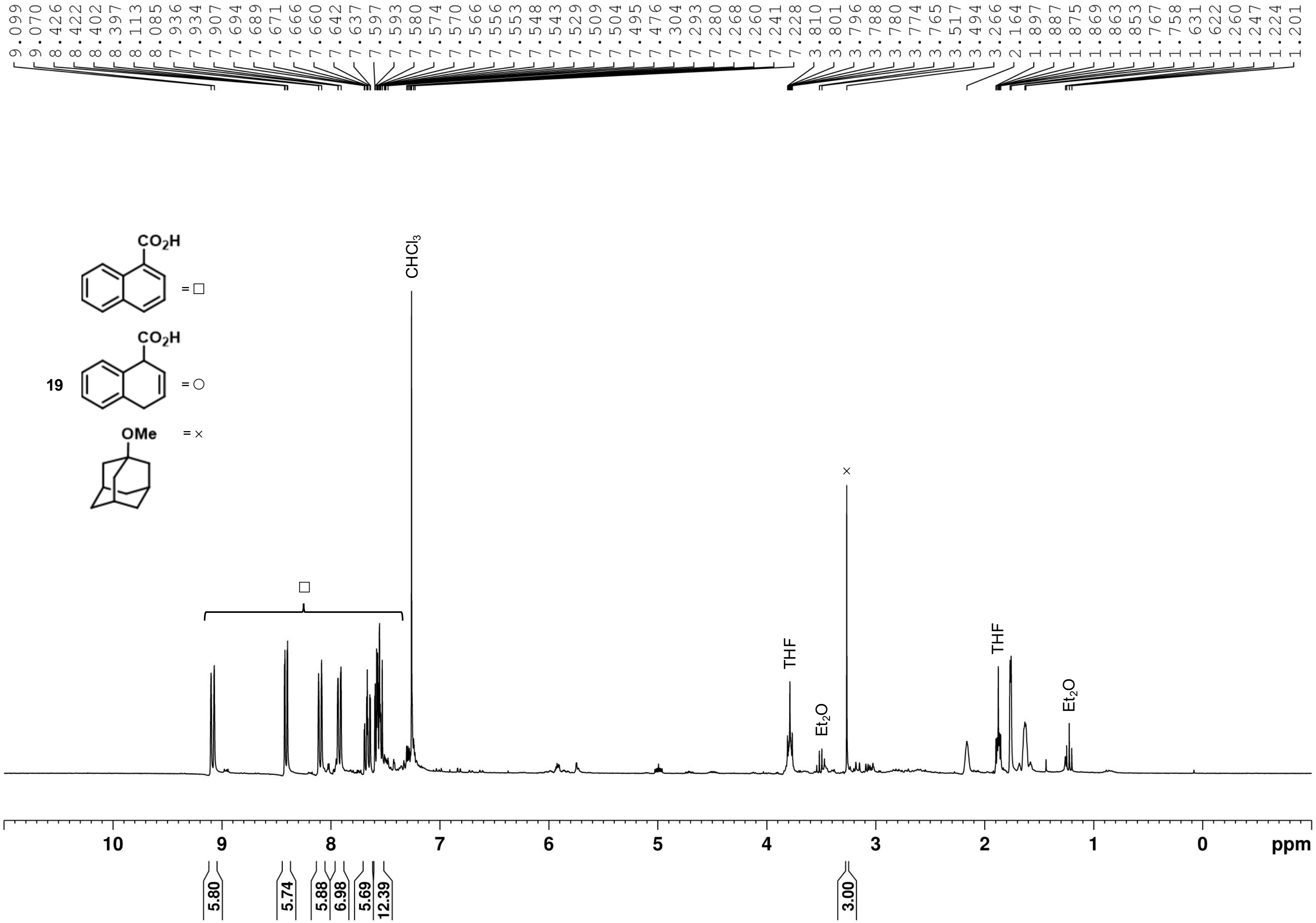

Spectrum S75. ^{1}H NMR spectrum of Table S12, entry 1 (300 MHz, $CDCl_3$, 298 K).

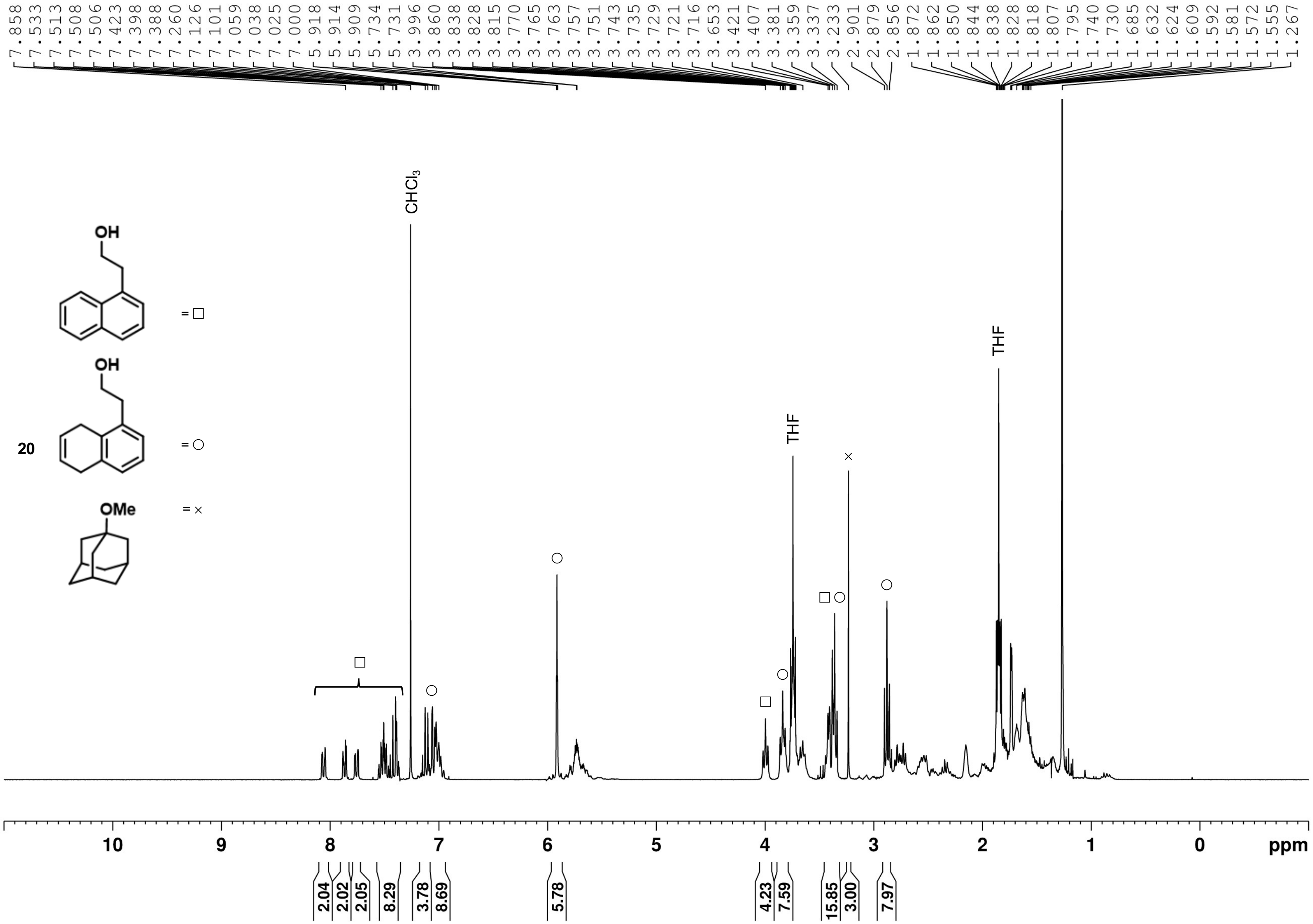

Spectrum S76. ^{1}H NMR spectrum of Table S12, entry 2 (300 MHz, $CDCl_3$, 298 K).

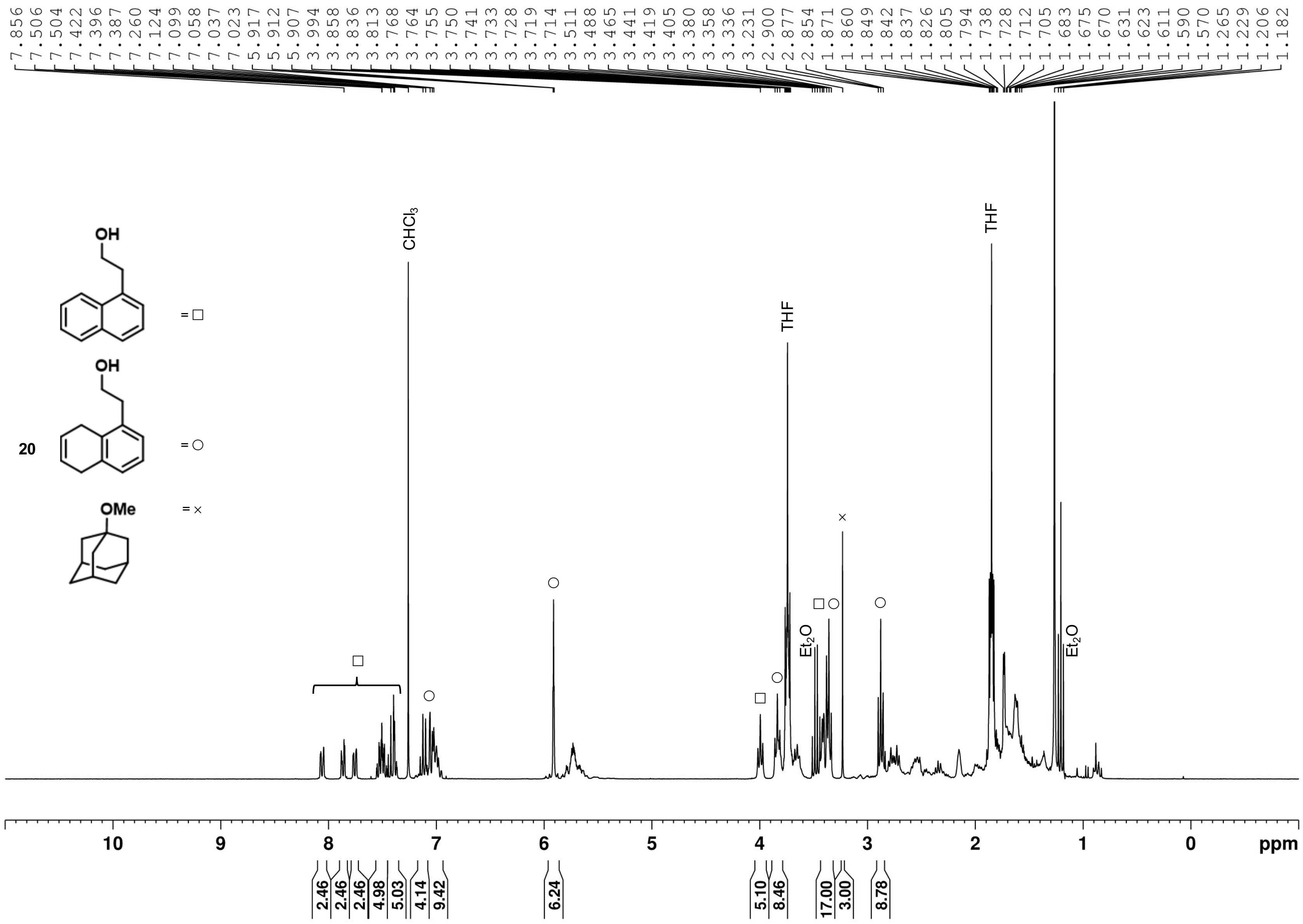

Spectrum S77. ^{1}H NMR spectrum of Table S12, entry 3 (300 MHz, $CDCl_3$, 298 K).

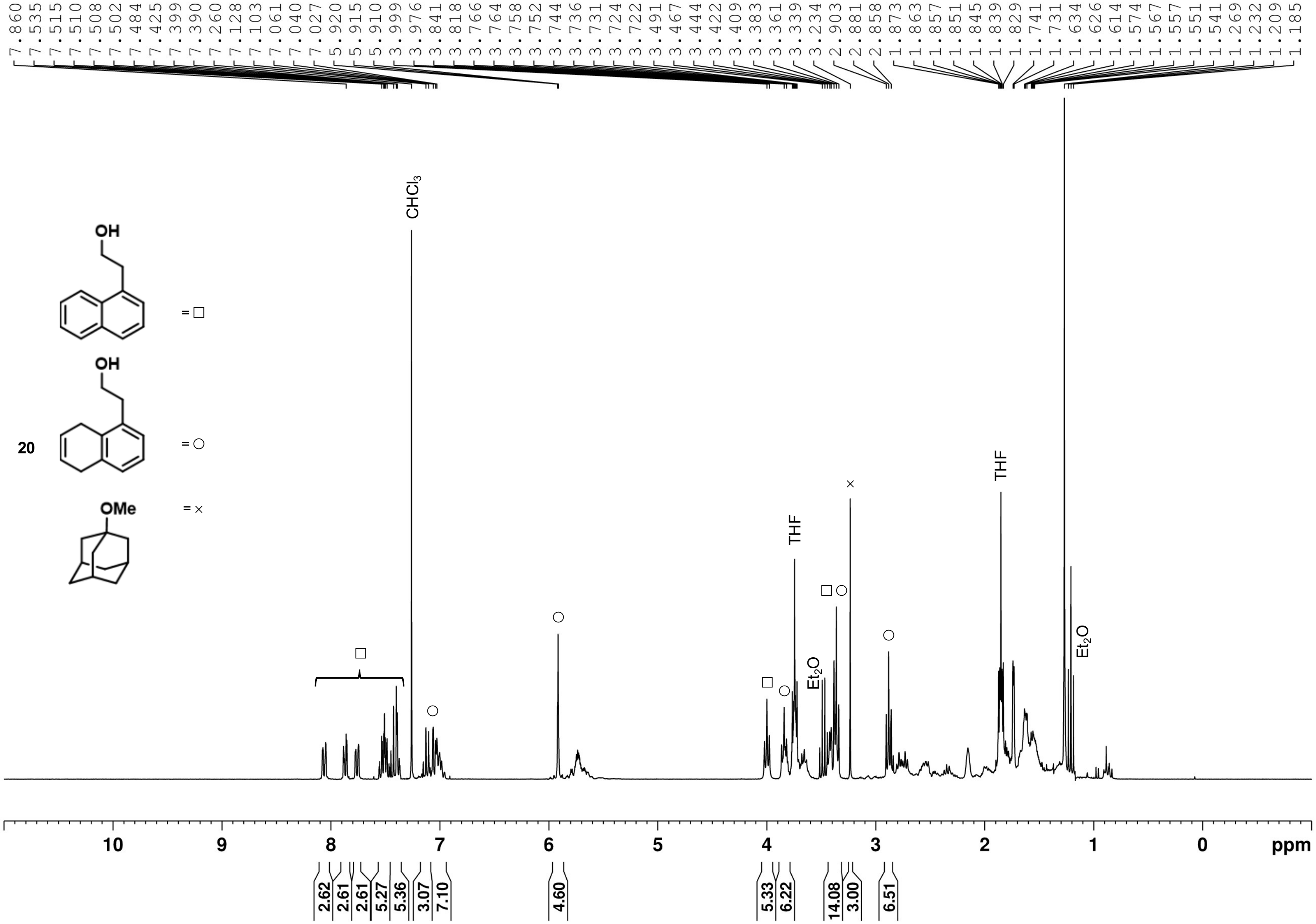

Spectrum S78. ^{1}H NMR spectrum of Table S13, entry 1 (300 MHz, $CDCl_3$, 298 K).

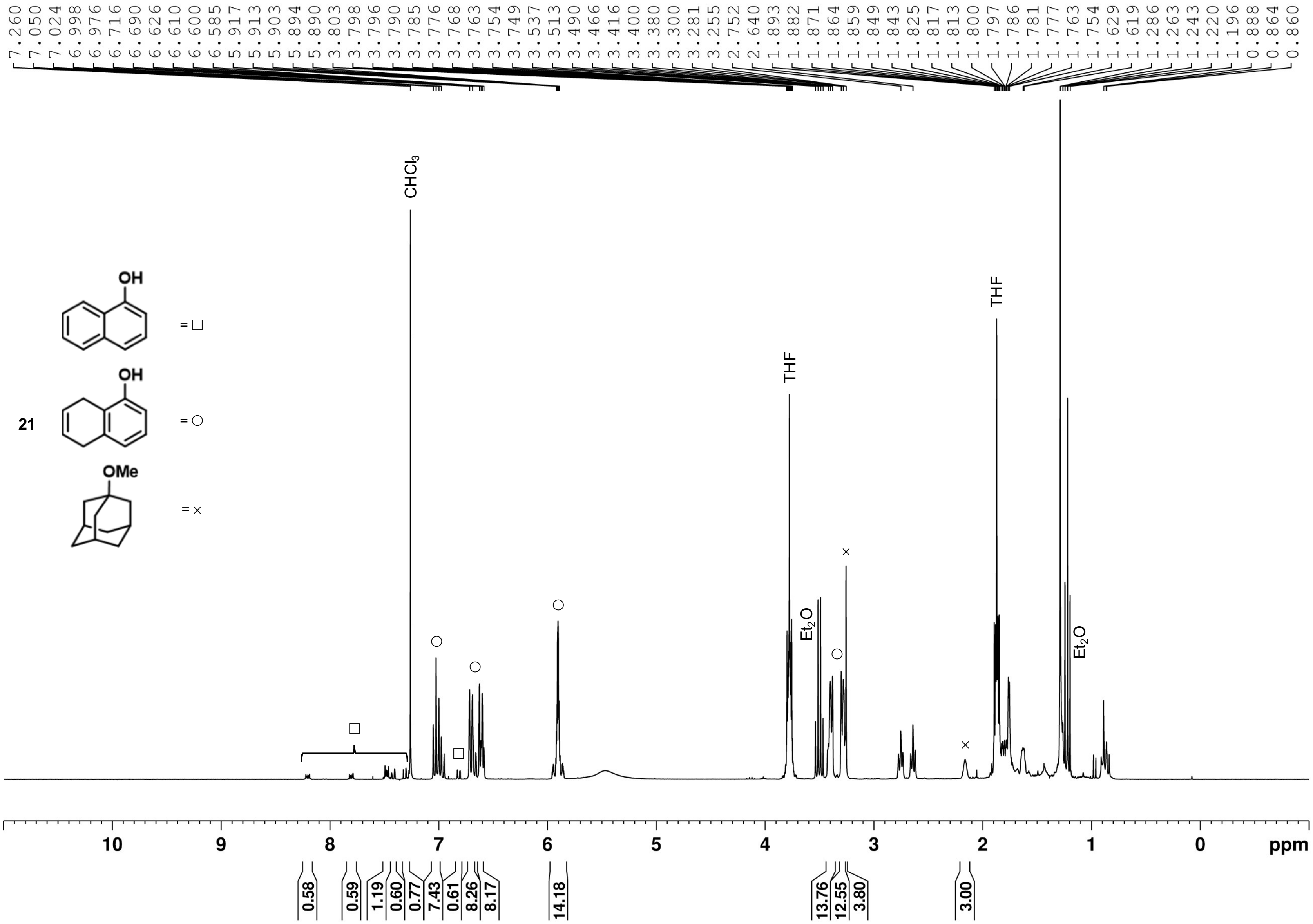

Spectrum S79. ^{1}H NMR spectrum of Table S13, entry 2 (300 MHz, $CDCl_3$, 298 K).

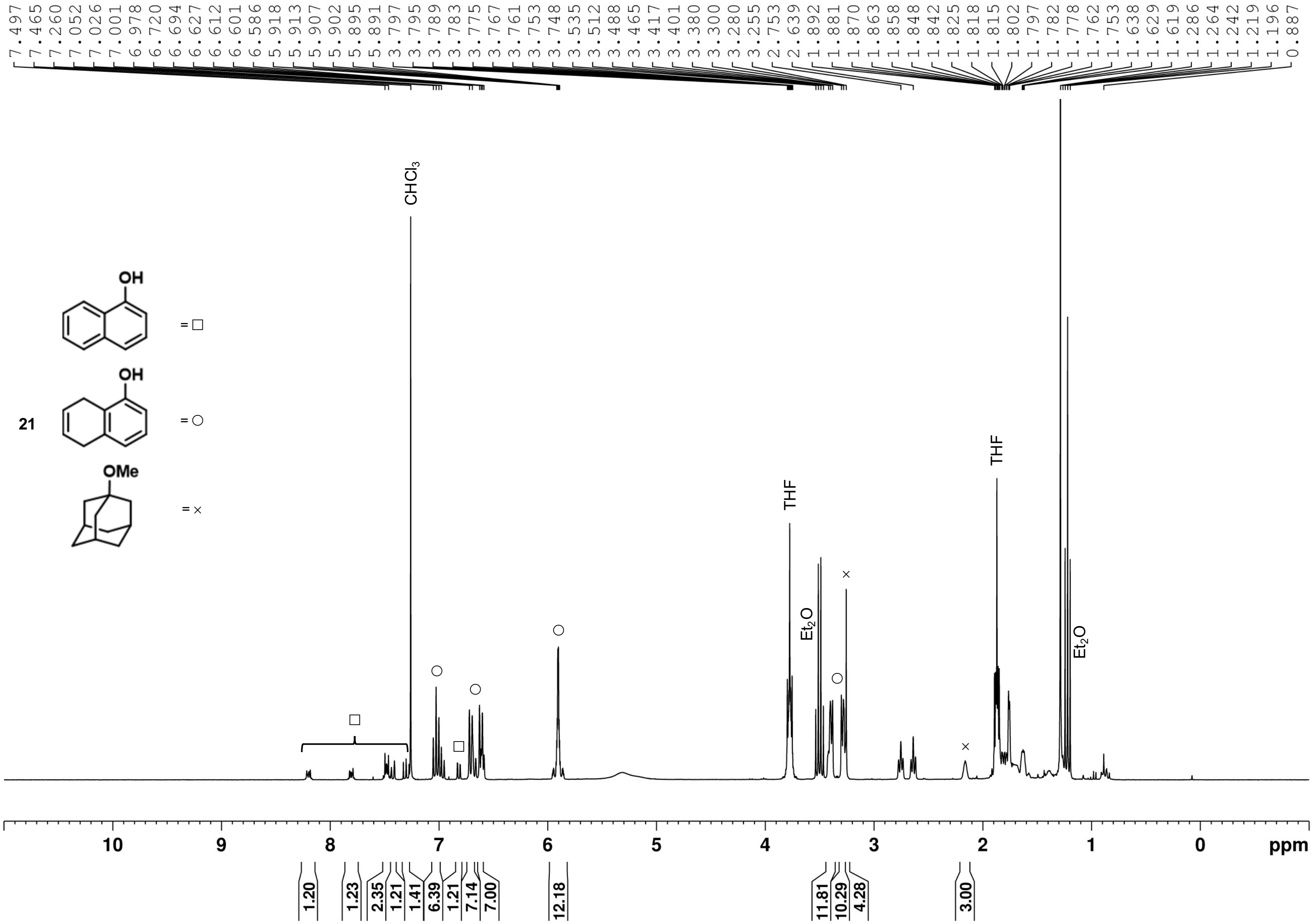

Spectrum S80. ^{1}H NMR spectrum of Table S13, entry 3 (300 MHz, $CDCl_3$, 298 K).

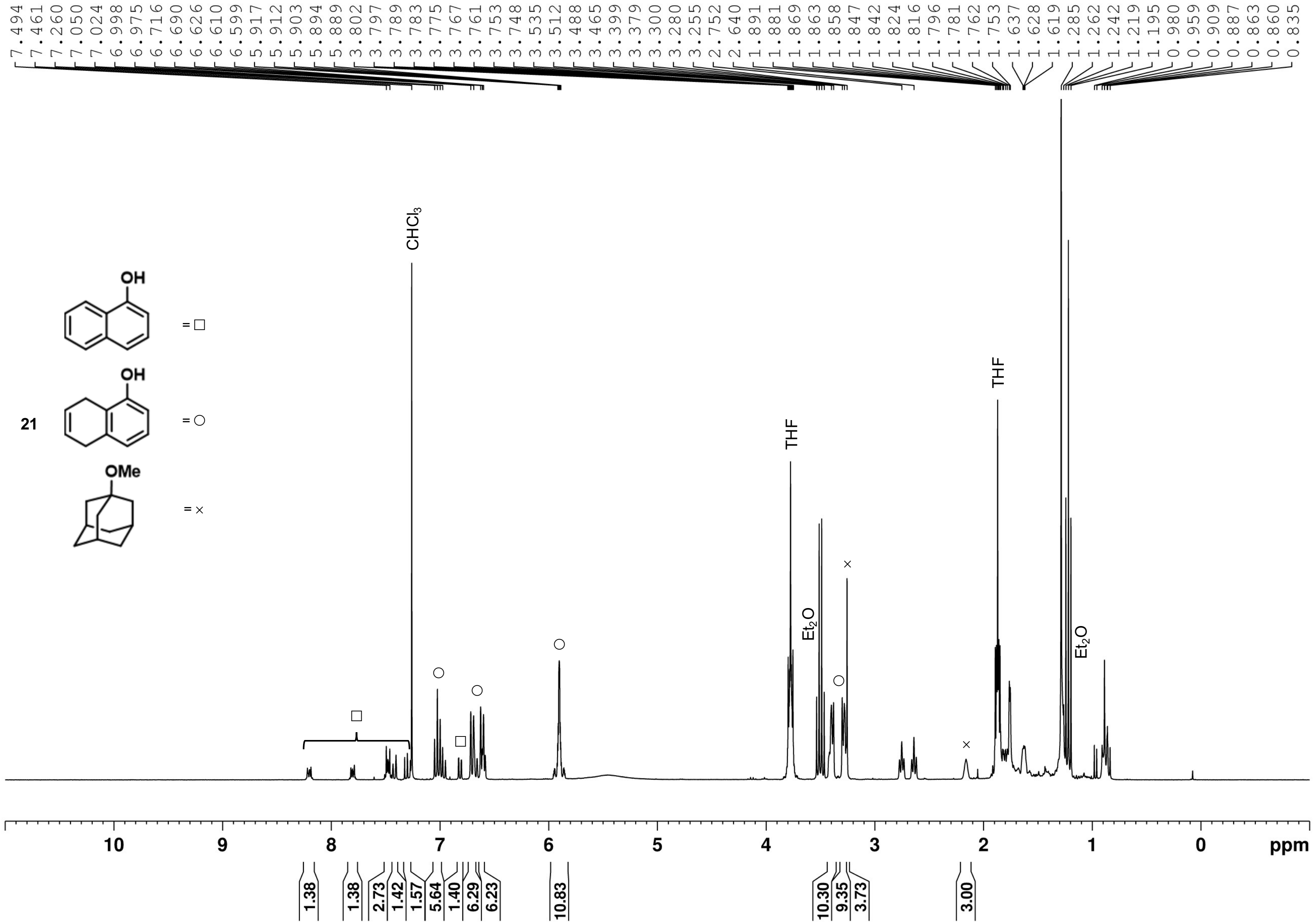

Spectrum S81. ^{1}H NMR spectrum of Table S14, entry 1 (300 MHz, $CDCl_3$, 298 K).

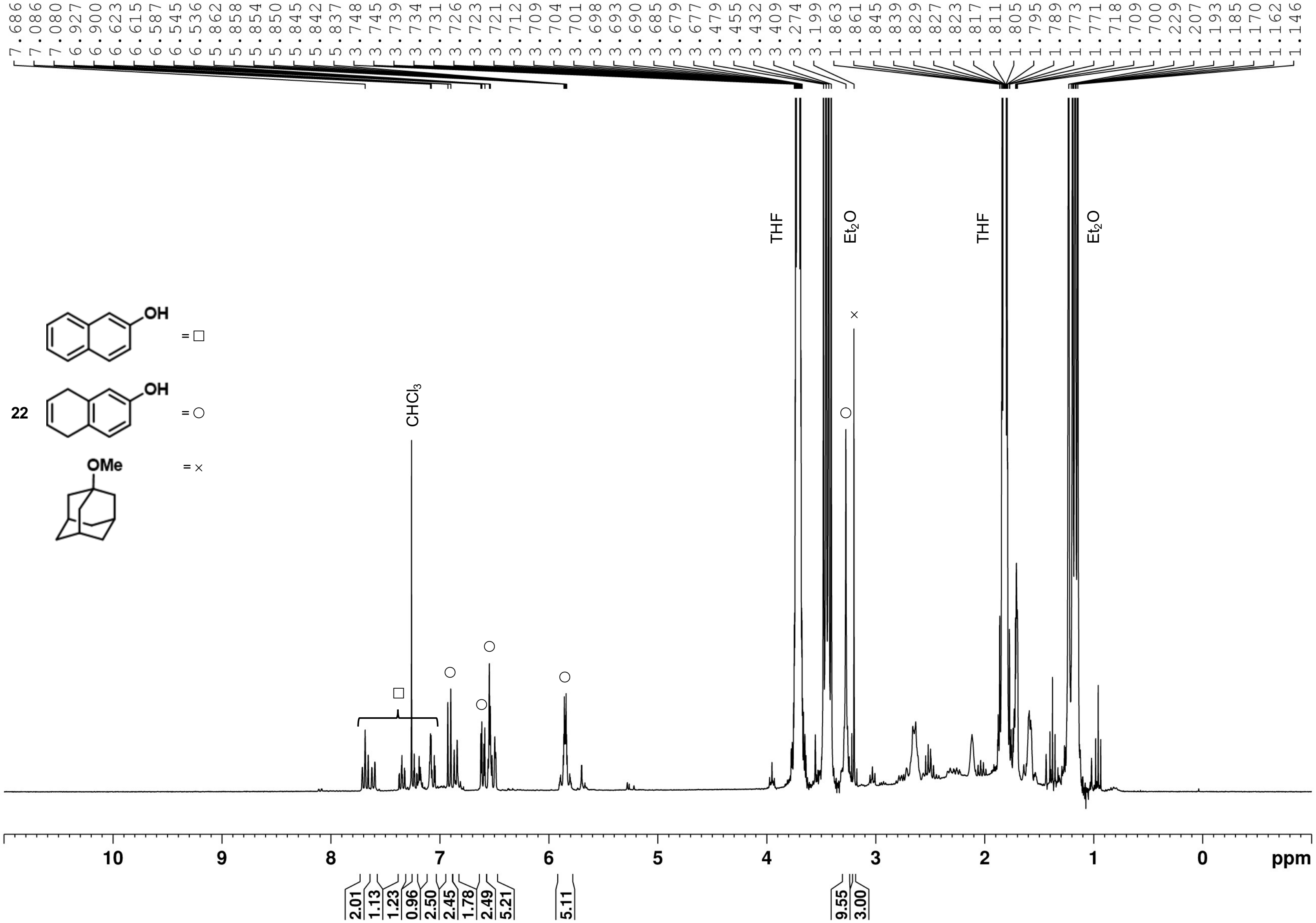

Spectrum S82. ^{1}H NMR spectrum of Table S14, entry 2 (300 MHz, $CDCl_3$, 298 K).

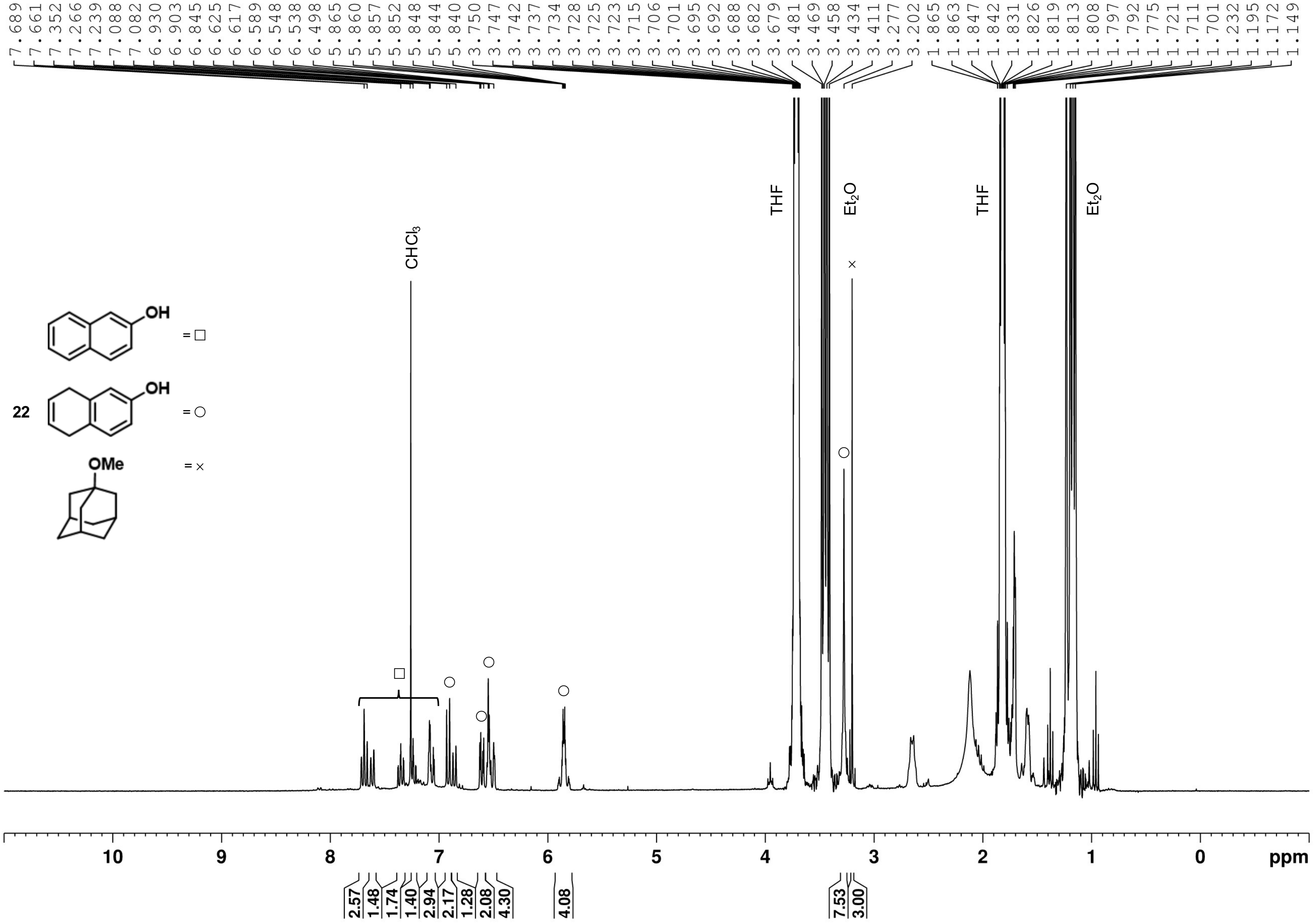

Spectrum S83. ^{1}H NMR spectrum of Table S14, entry 3 (300 MHz, $CDCl_3$, 298 K).

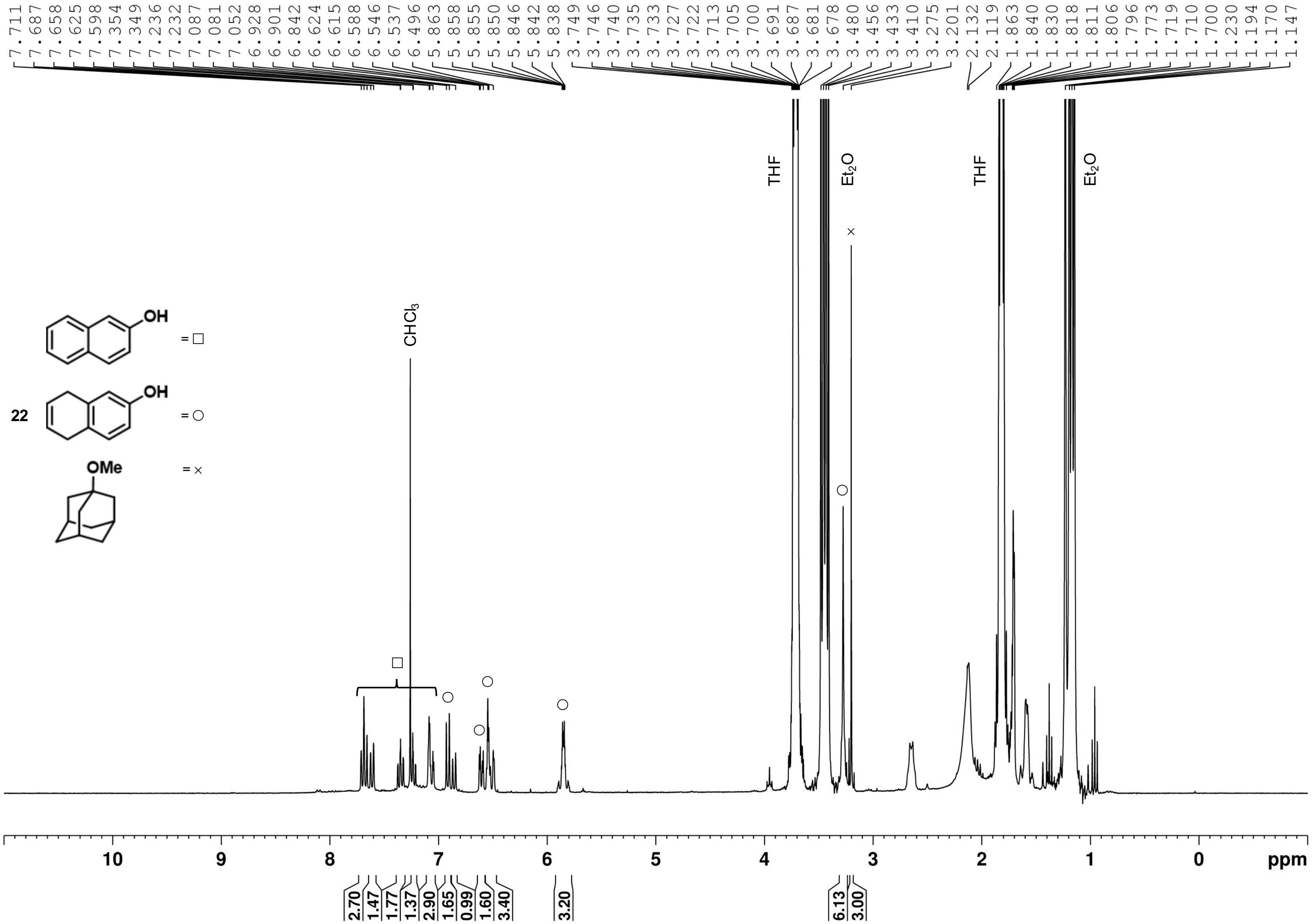

Spectrum S84. ^{1}H NMR spectrum of Table S15, entry 1 (300 MHz, $CDCl_3$, 298 K).

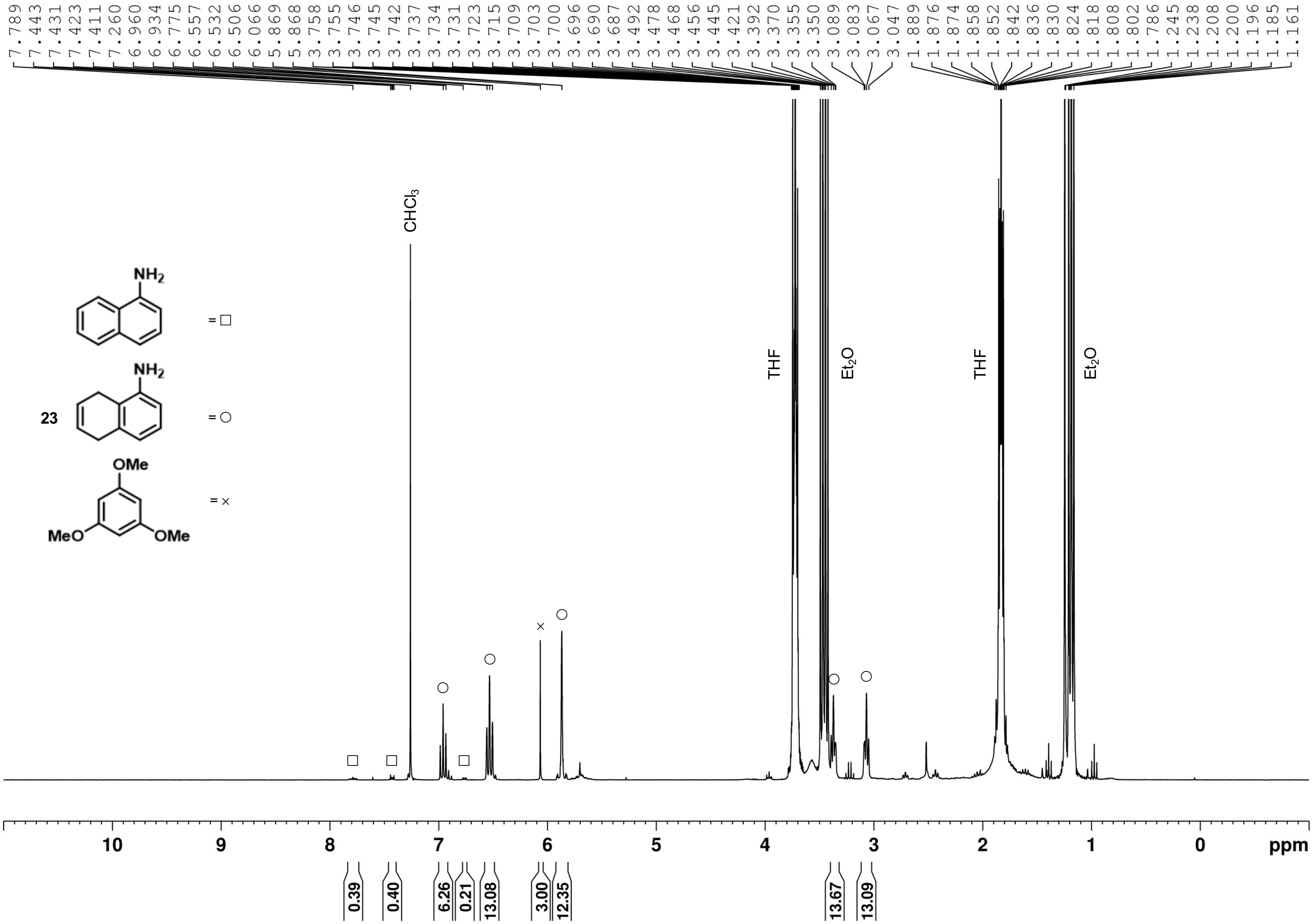

Spectrum S85. ^{1}H NMR spectrum of Table S15, entry 2 (300 MHz, $CDCl_3$, 298 K).

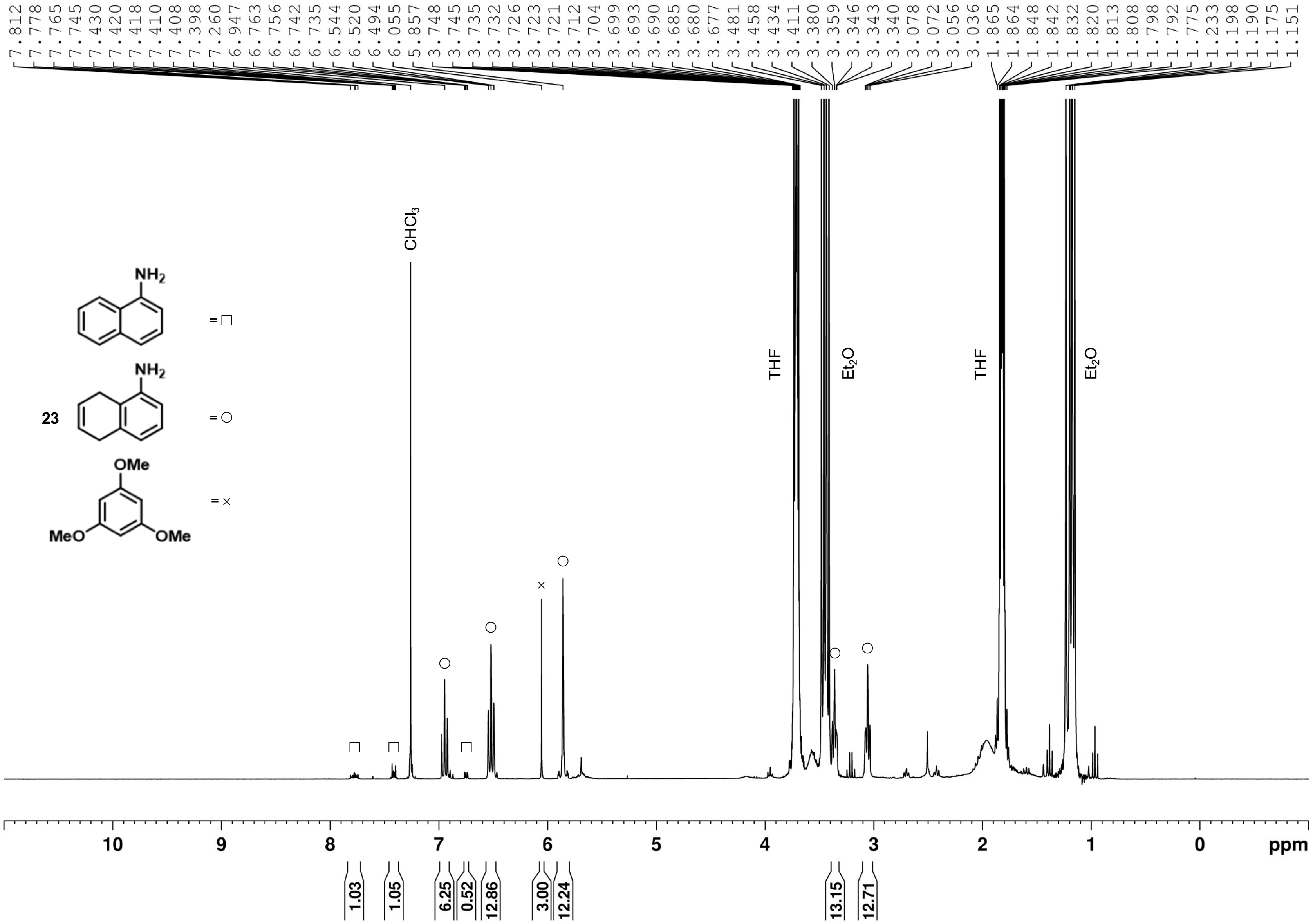

Spectrum S86. ^{1}H NMR spectrum of Table S15, entry 3 (300 MHz, $CDCl_3$, 298 K).

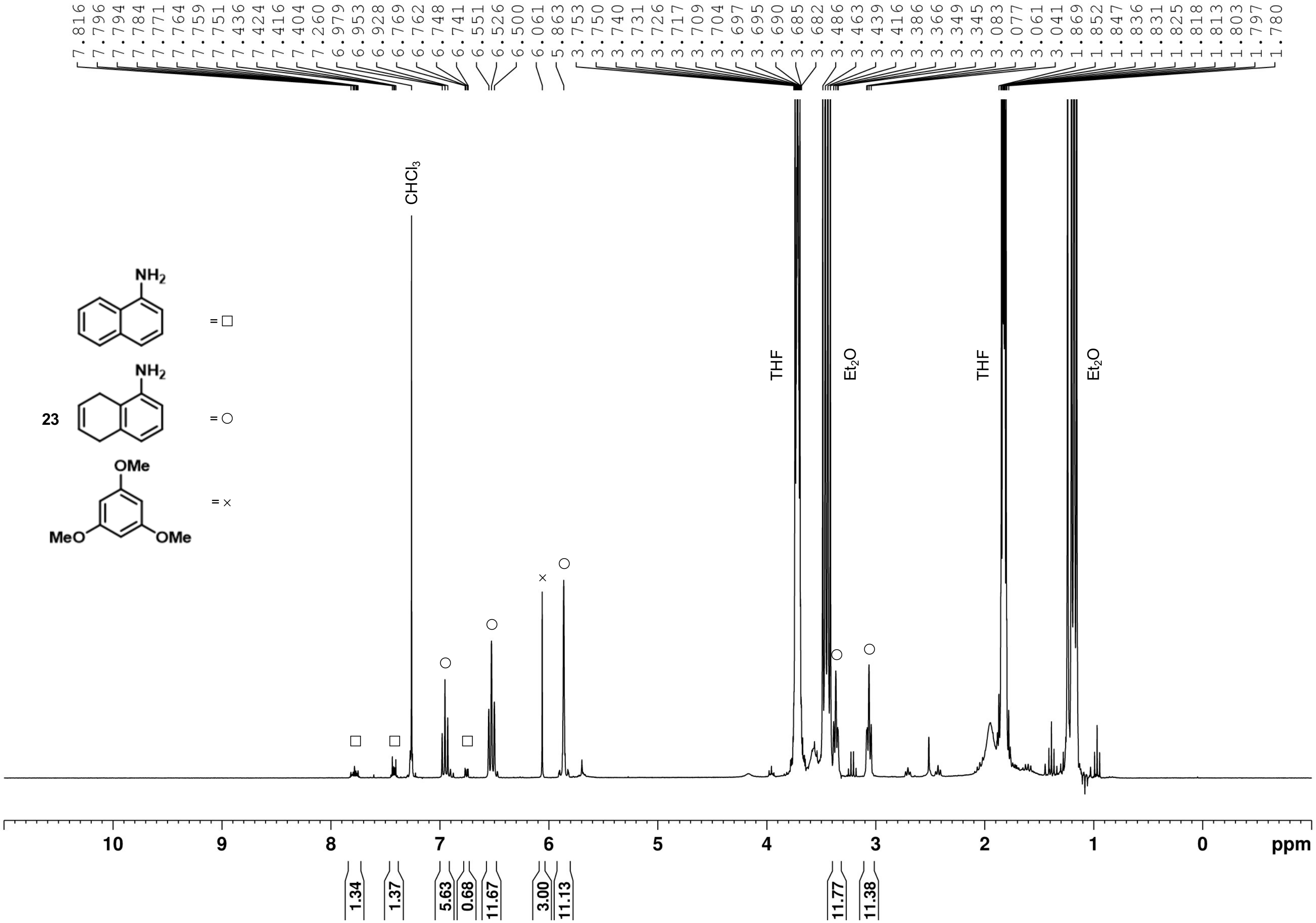

Spectrum S87. ^{1}H NMR spectrum of the reduction of 1-aminonaphthalene on a 20 mmol scale (300 MHz, $CDCl_3$, 298 K).

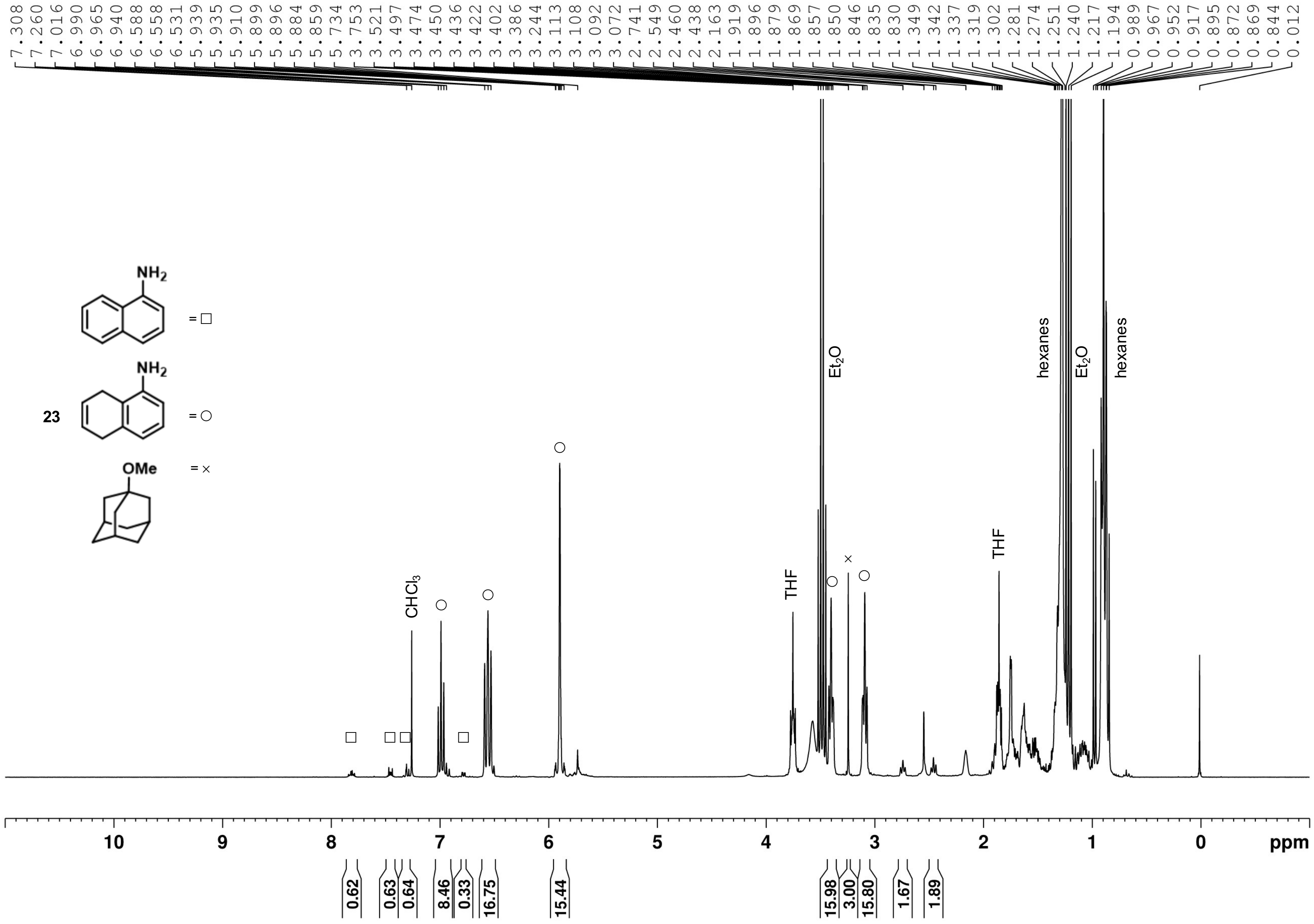

Spectrum S88. ^{1}H NMR spectrum of 5,8-dihydronaphthalene-2,3-diol (**24**) (300 MHz, DMSO-d_6, 298 K).

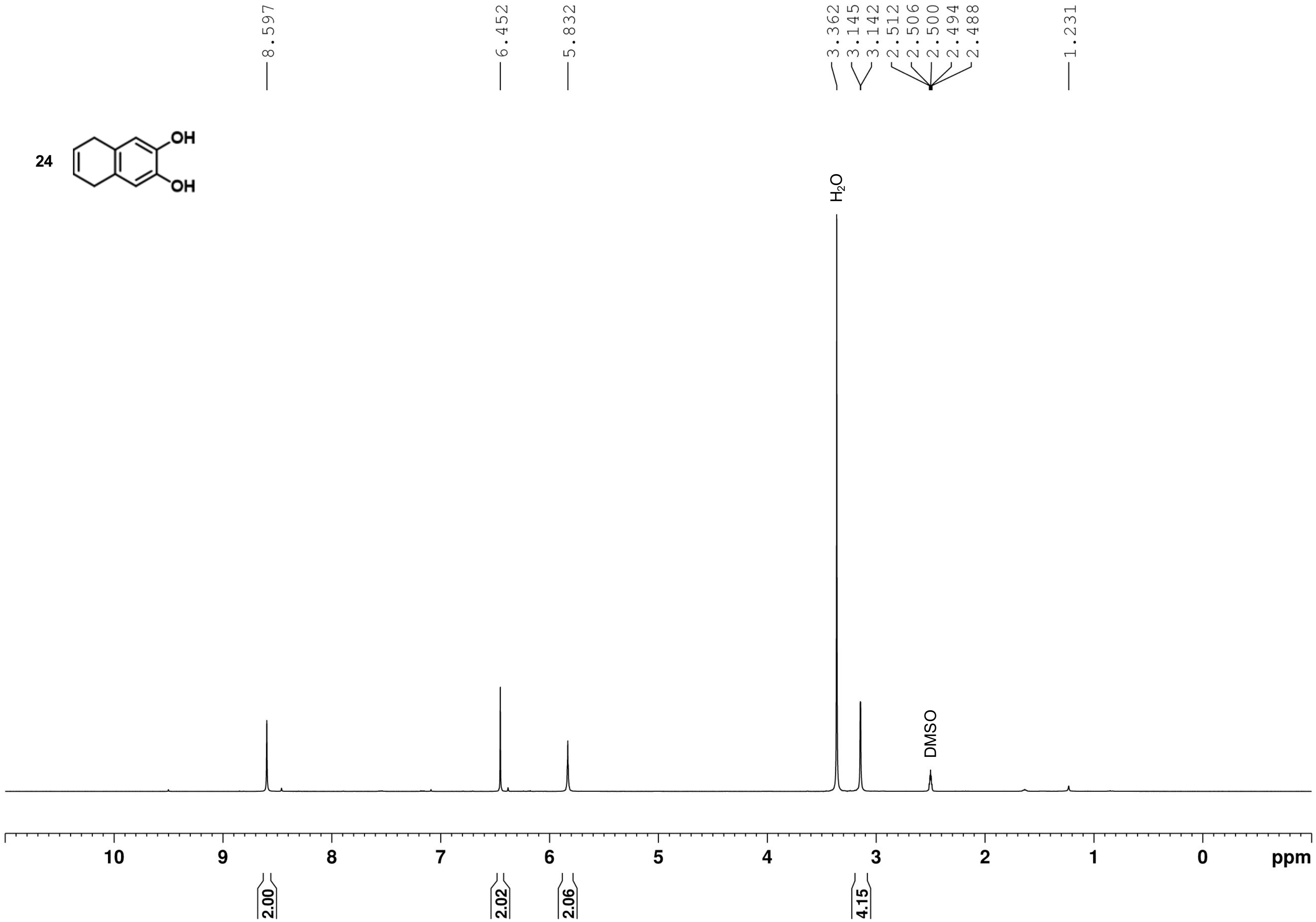

Spectrum S89. ^{13}C NMR spectrum of 5,8-dihydronaphthalene-2,3-diol (**24**) (300 MHz, DMSO-d_6, 298 K).

24

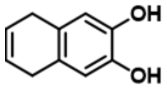

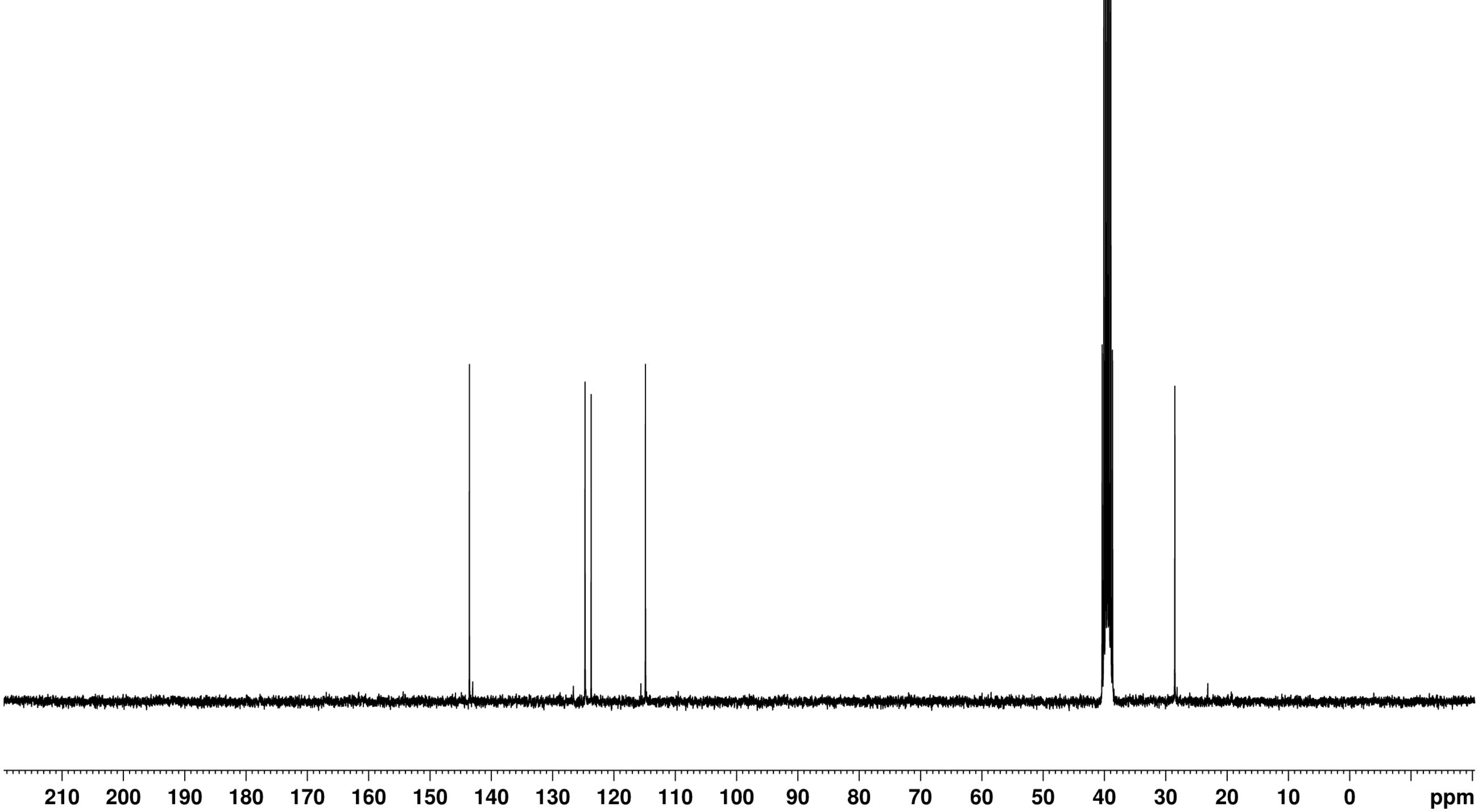

Spectrum S90. ^{1}H NMR spectrum of Table S16, entry 1 (300 MHz, $CDCl_3$, 298 K).

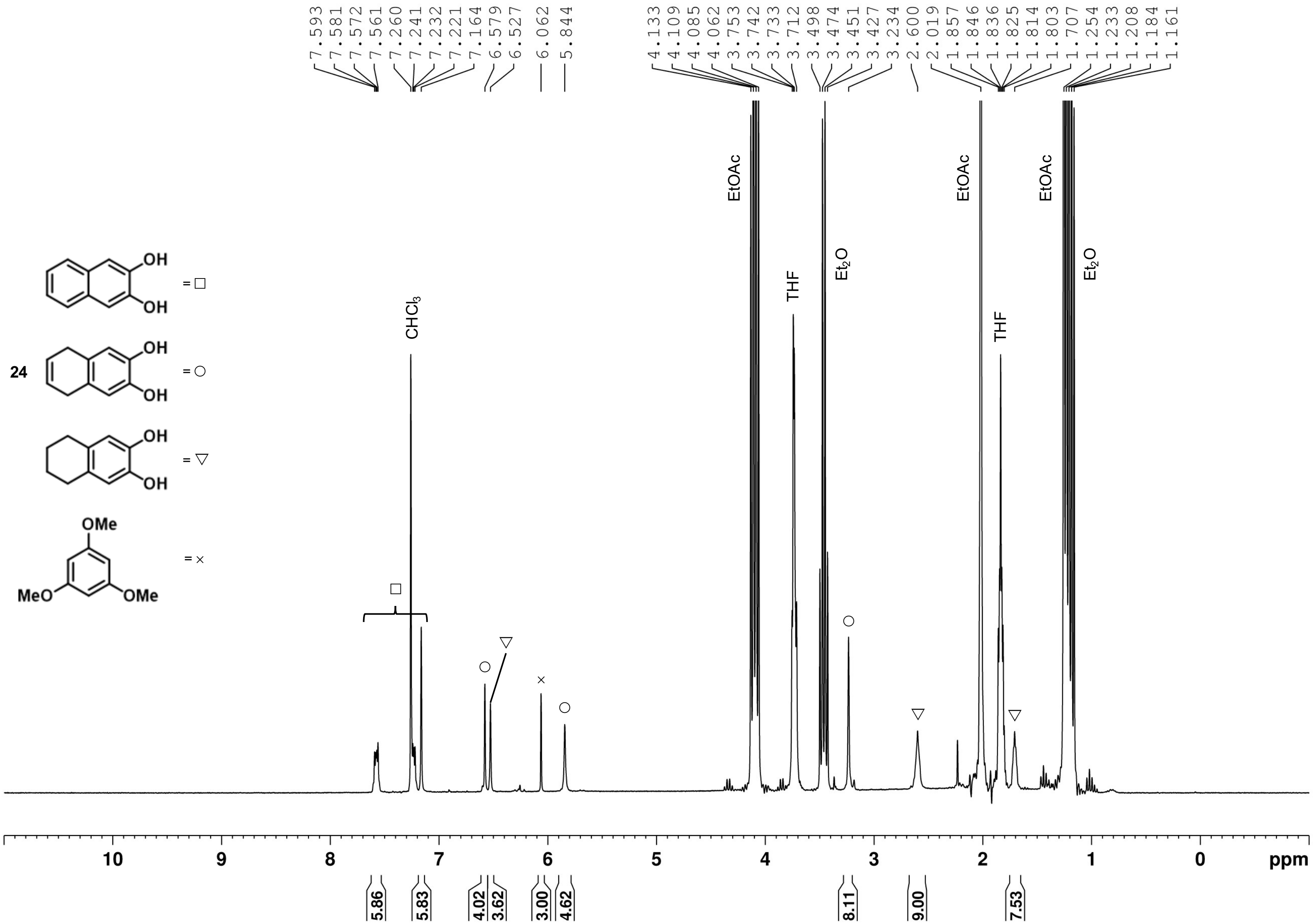

Spectrum S91. ^{1}H NMR spectrum of Table S16, entry 2 (300 MHz, $CDCl_3$, 298 K).

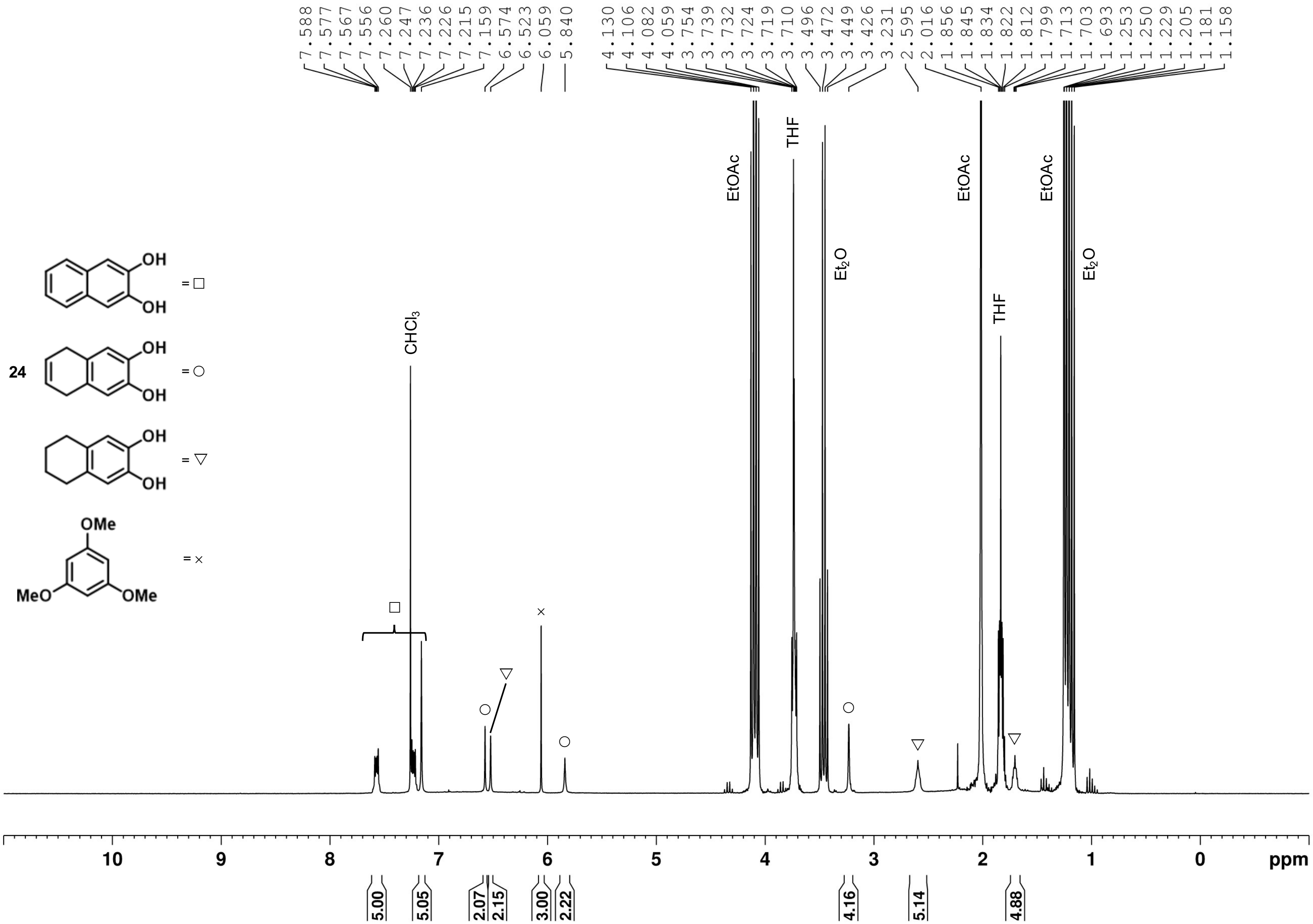

Spectrum S92. ^1H NMR spectrum of Table S16, entry 3 (300 MHz, $CDCl_3$, 298 K).

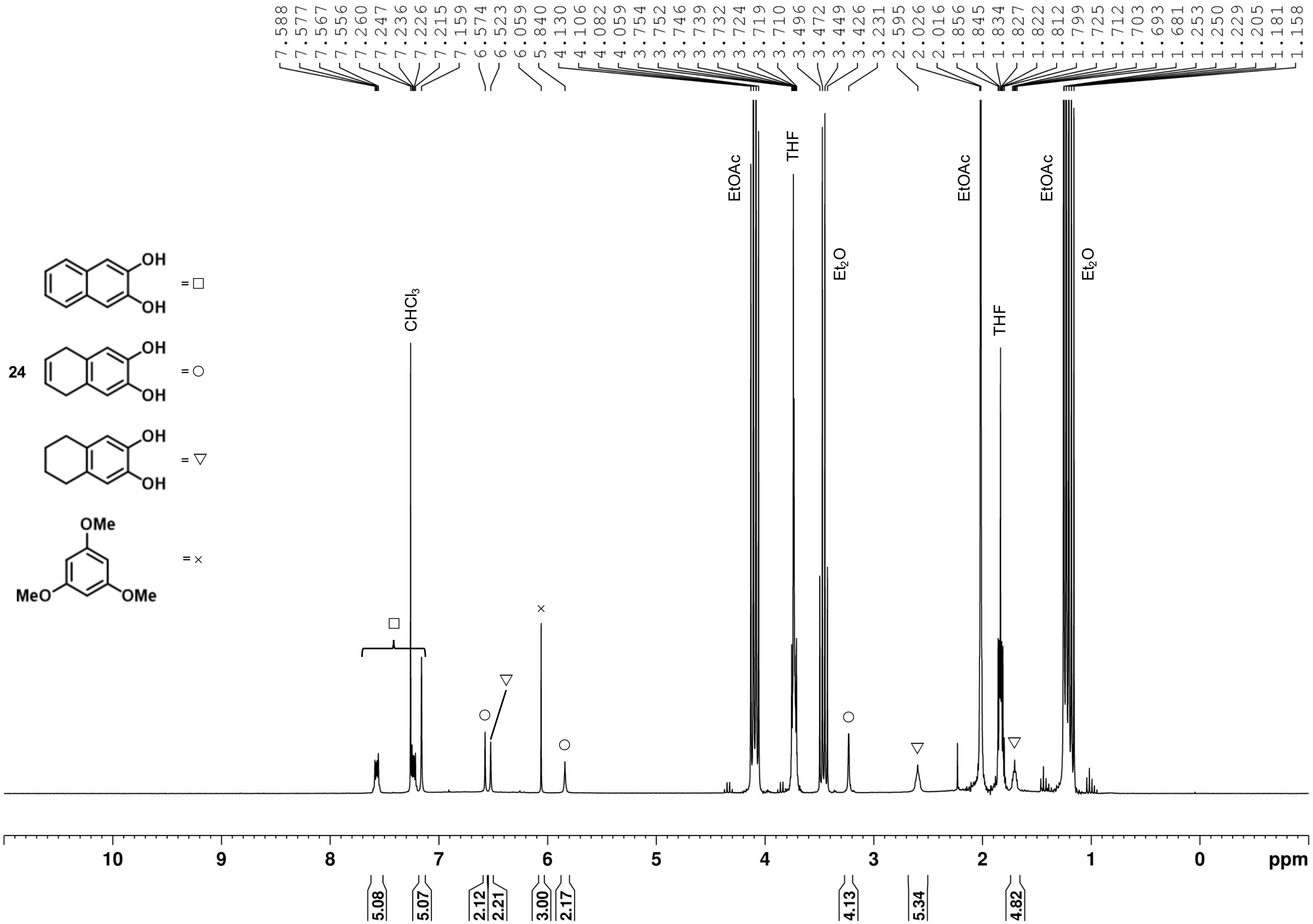

Spectrum S93. ^{1}H NMR spectrum of 1,4,5,8,9,10-hexahydroanthracene (**26**) (300 MHz, $CDCl_3$, 298 K).

26

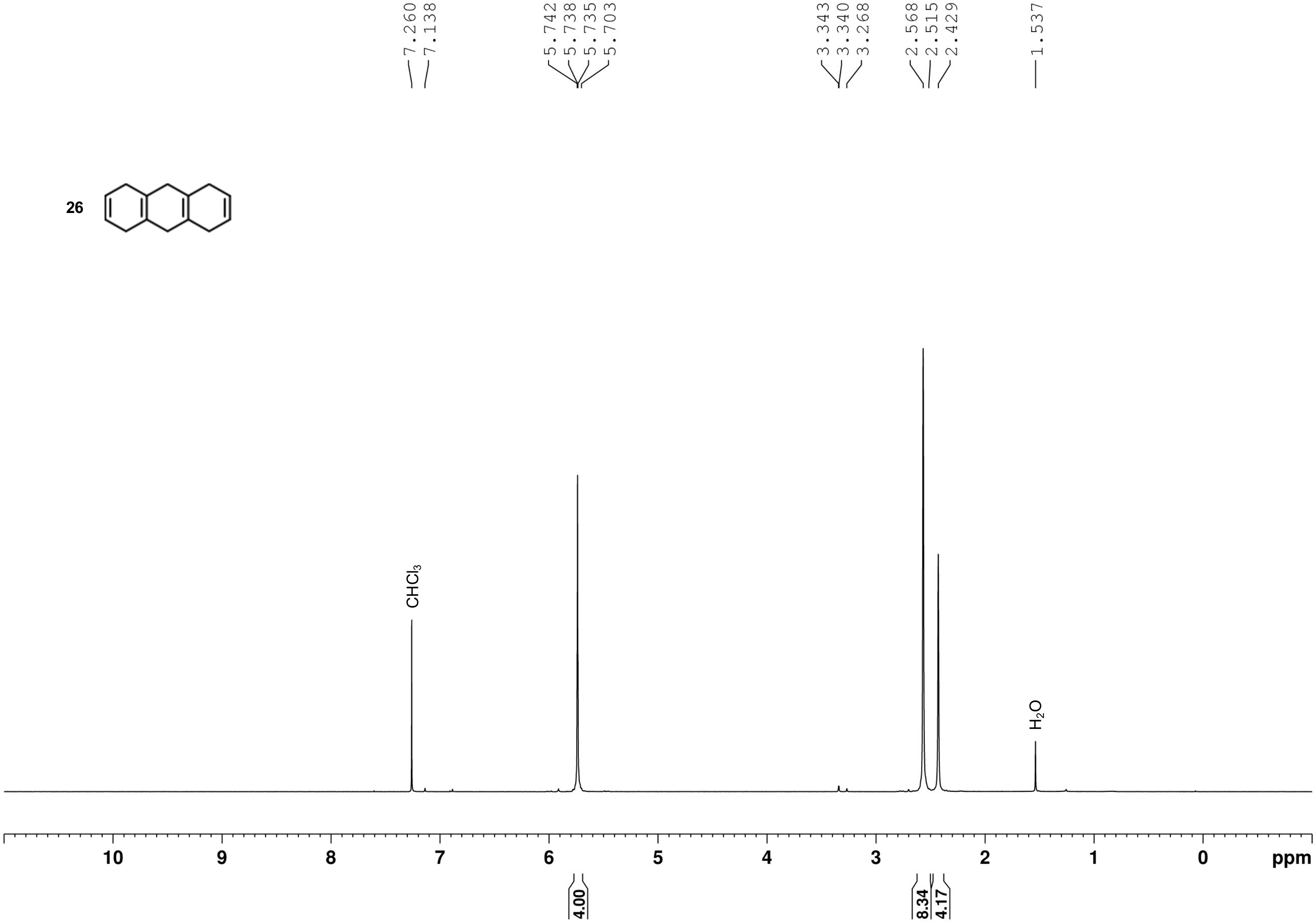

Spectrum S94. ^{13}C NMR spectrum of 1,4,5,8,9,10-hexahydroanthracene (**26**) (75 MHz, $CDCl_3$, 298 K).

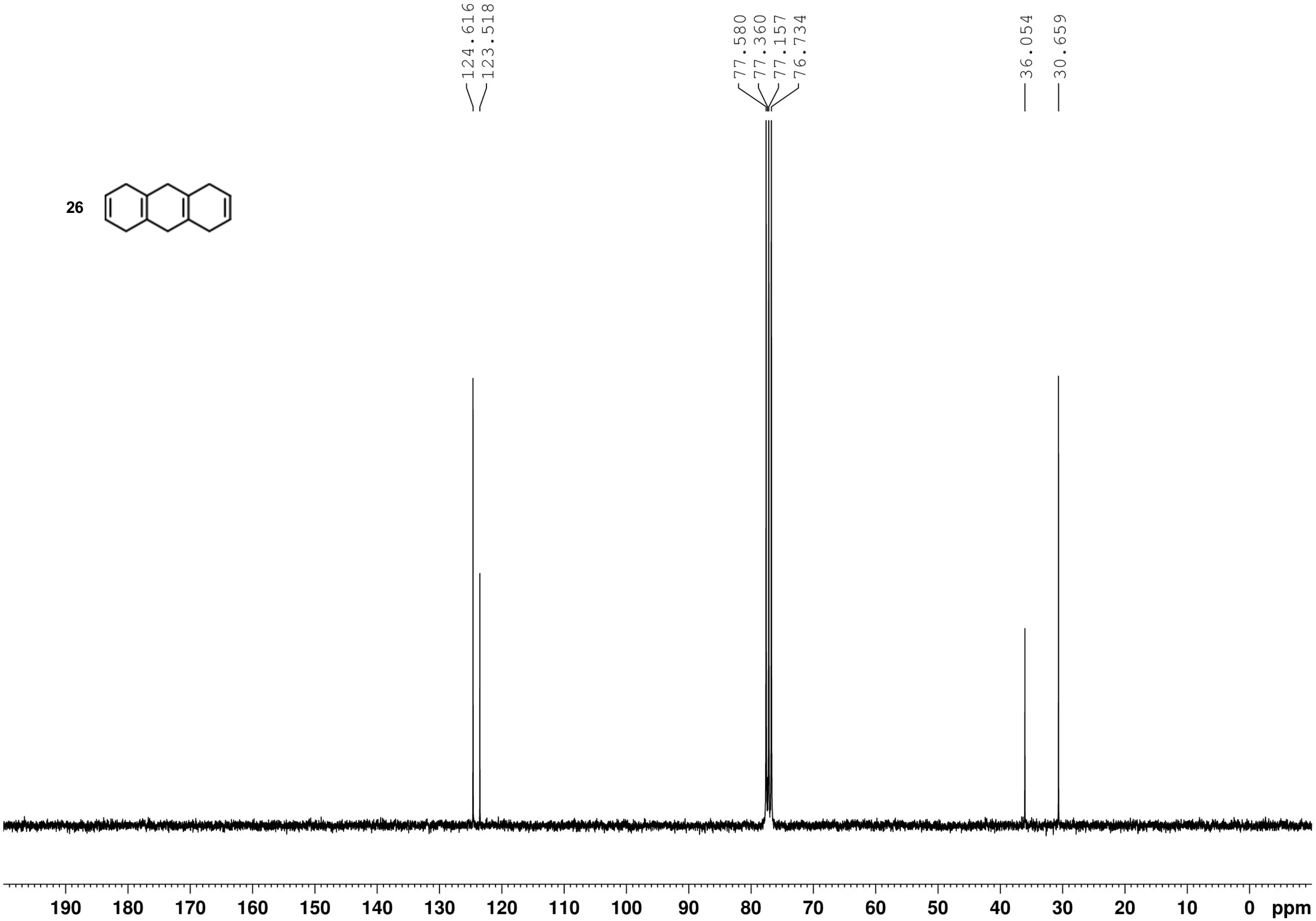

Spectrum S95. ^{1}H NMR spectrum of Table S17, entry 1 (300 MHz, $CDCl_3$, 298 K).

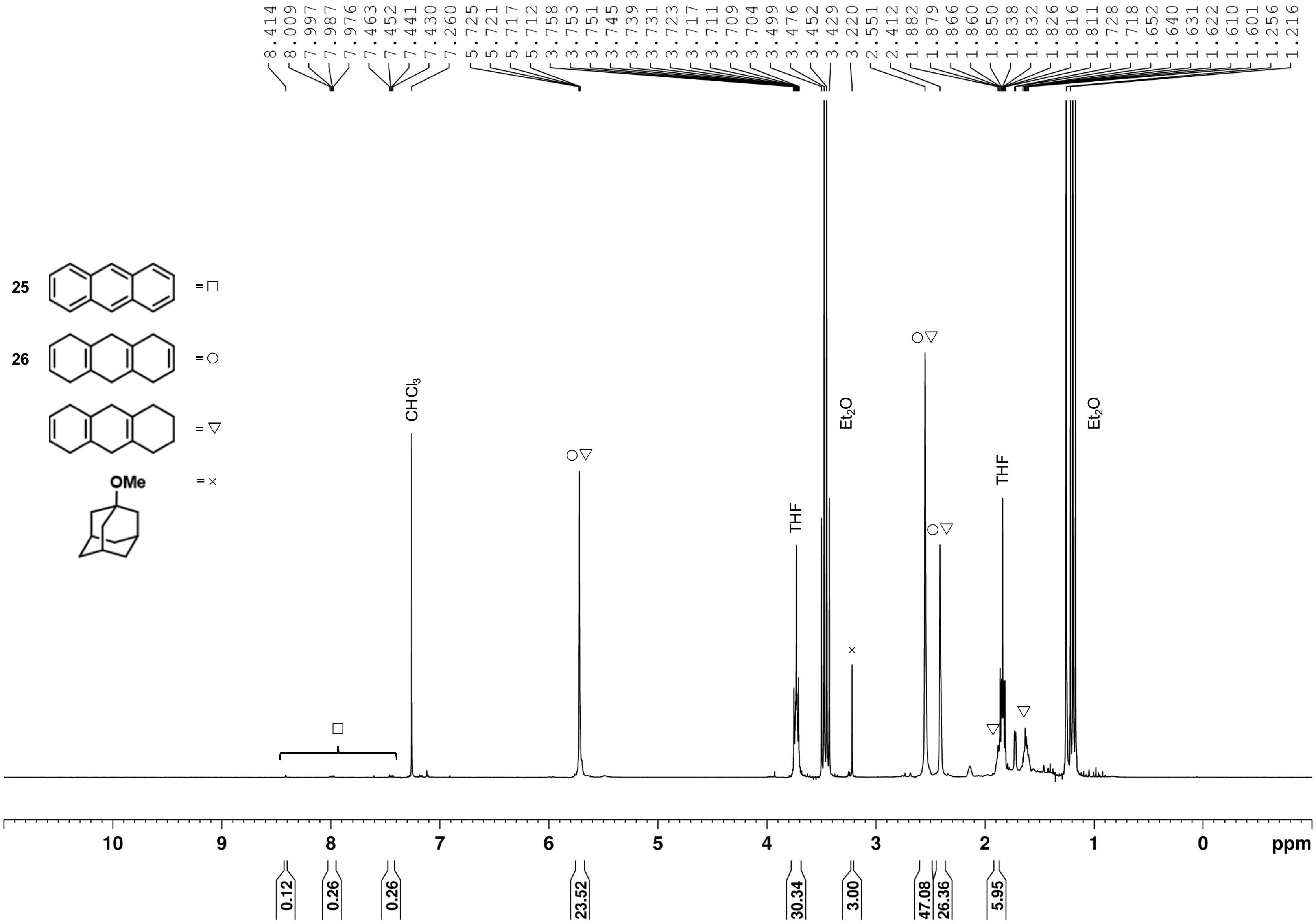

Spectrum S96. ^{1}H NMR spectrum of Table S17, entry 2 (300 MHz, $CDCl_3$, 298 K).

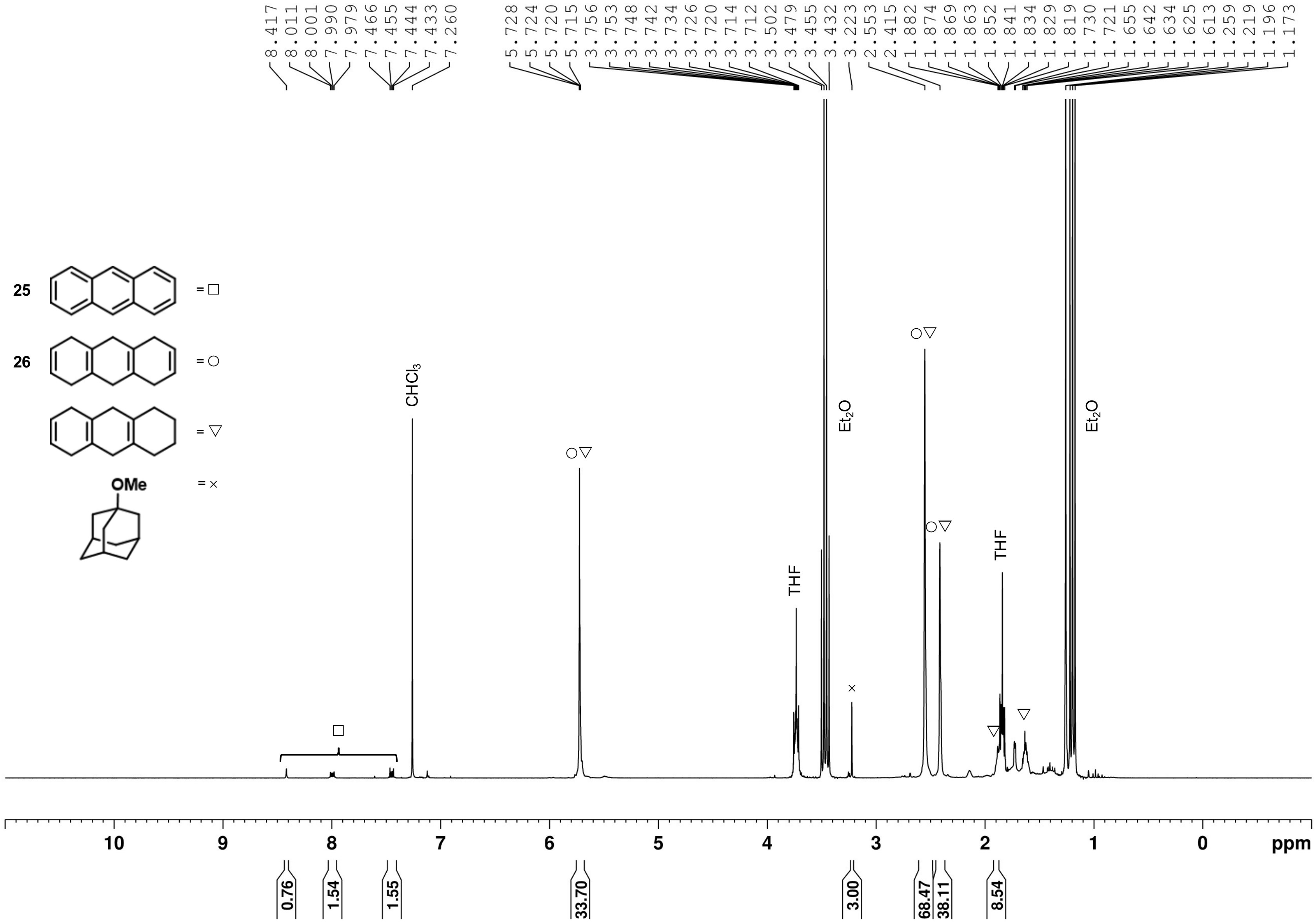

Spectrum S97. ^{1}H NMR spectrum of Table S17, entry 3 (300 MHz, $CDCl_3$, 298 K).

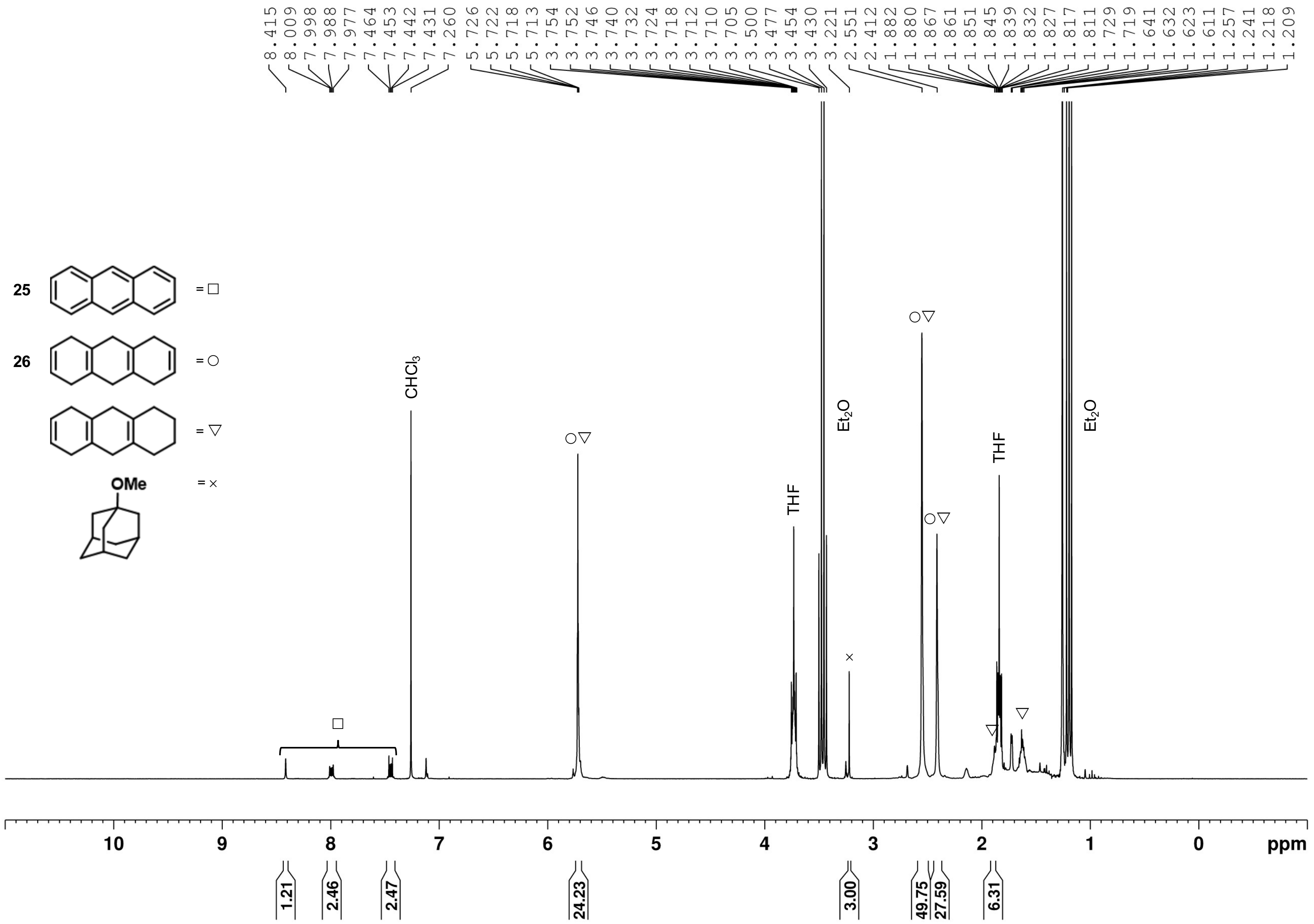

Spectrum S98. ^{1}H NMR spectrum of Table S18, entry 1 (300 MHz, $CDCl_3$, 298 K).

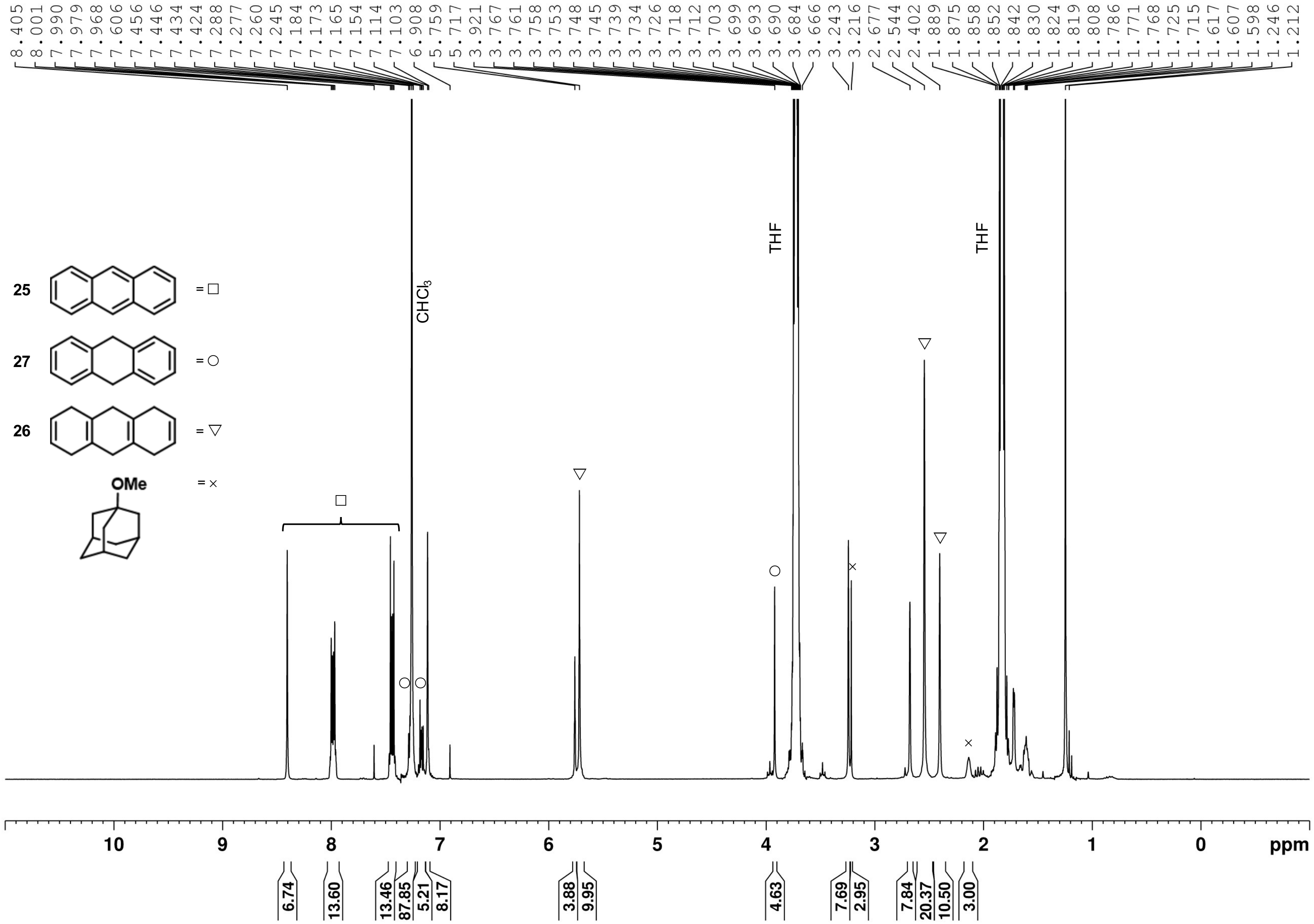

Spectrum S99. ^{1}H NMR spectrum of Table S18, entry 2 (300 MHz, $CDCl_3$, 298 K).

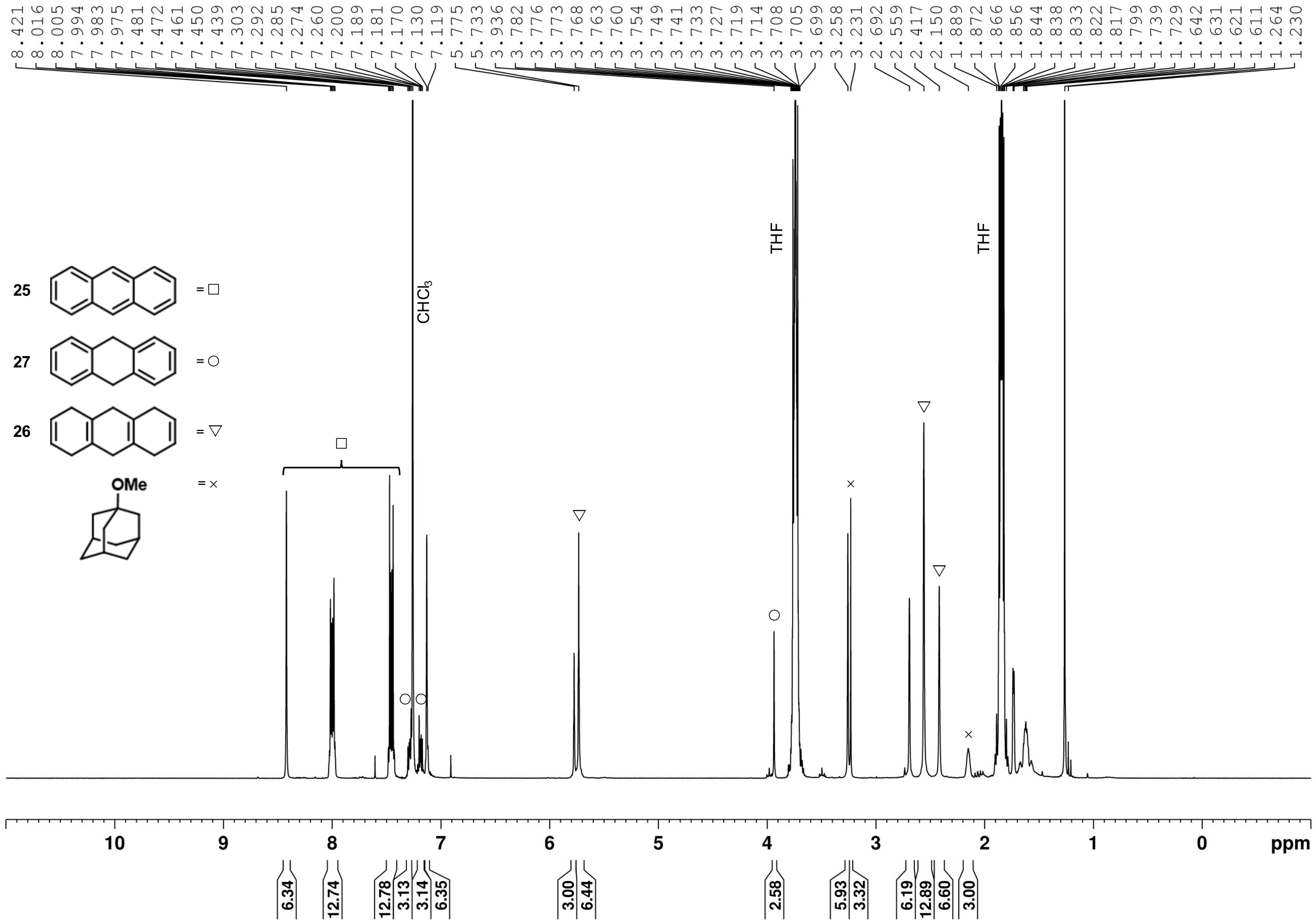

Spectrum S100. ^{1}H NMR spectrum of Table S18, entry 3 (300 MHz, $CDCl_3$, 298 K).

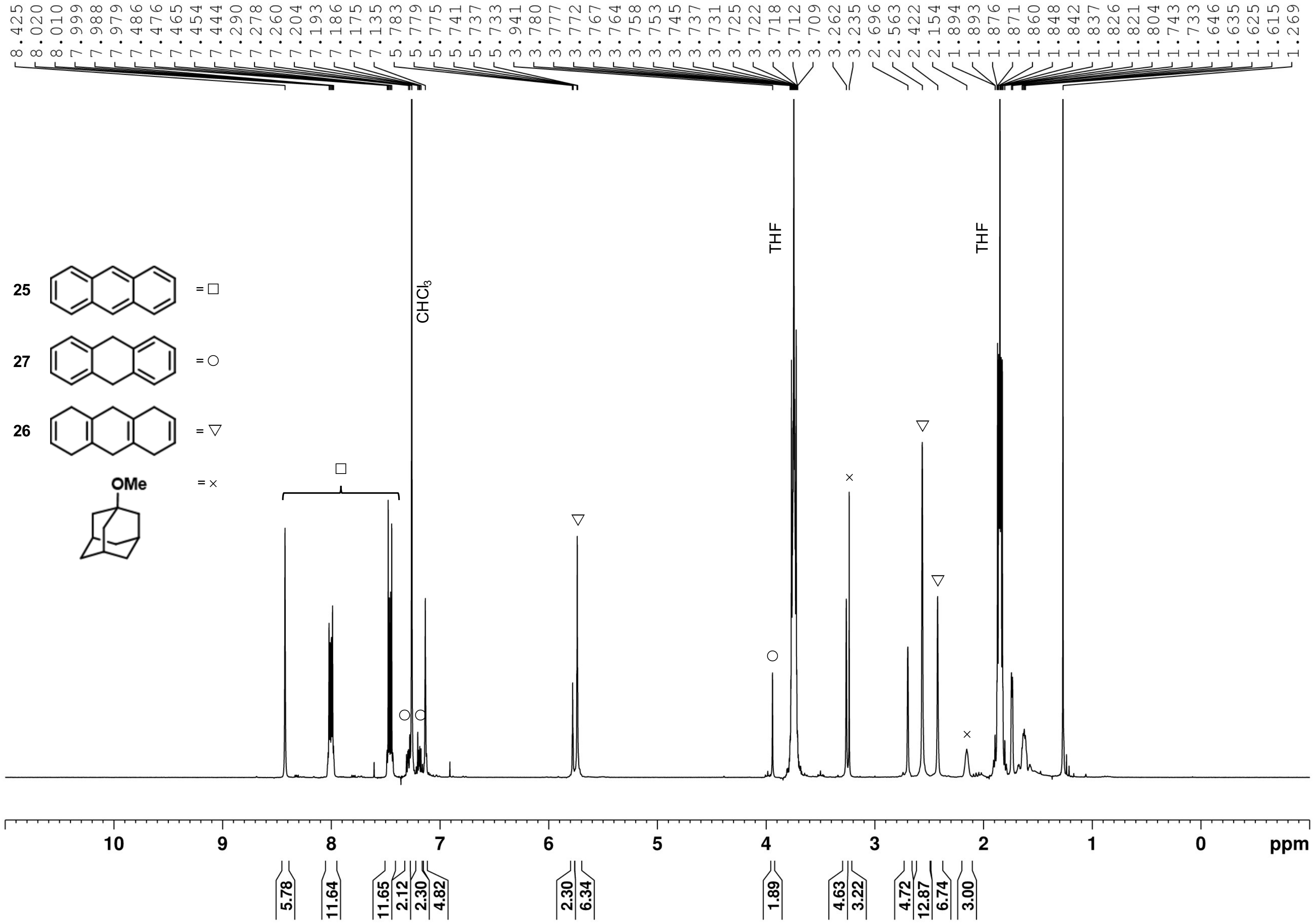

Spectrum S101. ^{1}H NMR spectrum of 1,4,9,10-tetrahydroanthracene (**28**) (300 MHz, $CDCl_3$, 298 K).

28

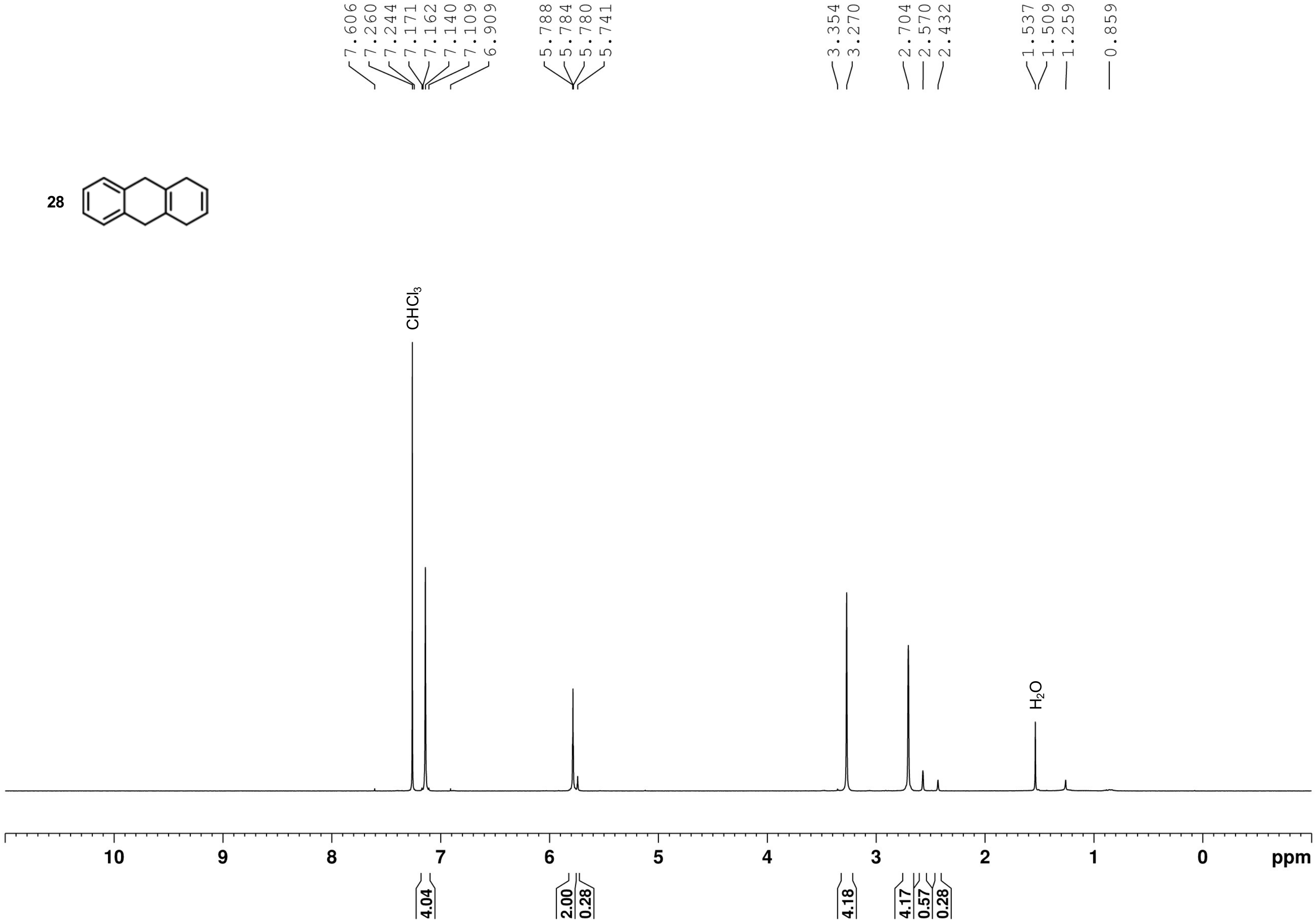

Spectrum S102. ^{13}C NMR spectrum of 1,4,9,10-tetrahydroanthracene (**28**) (300 MHz, $CDCl_3$, 298 K).

28 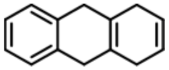

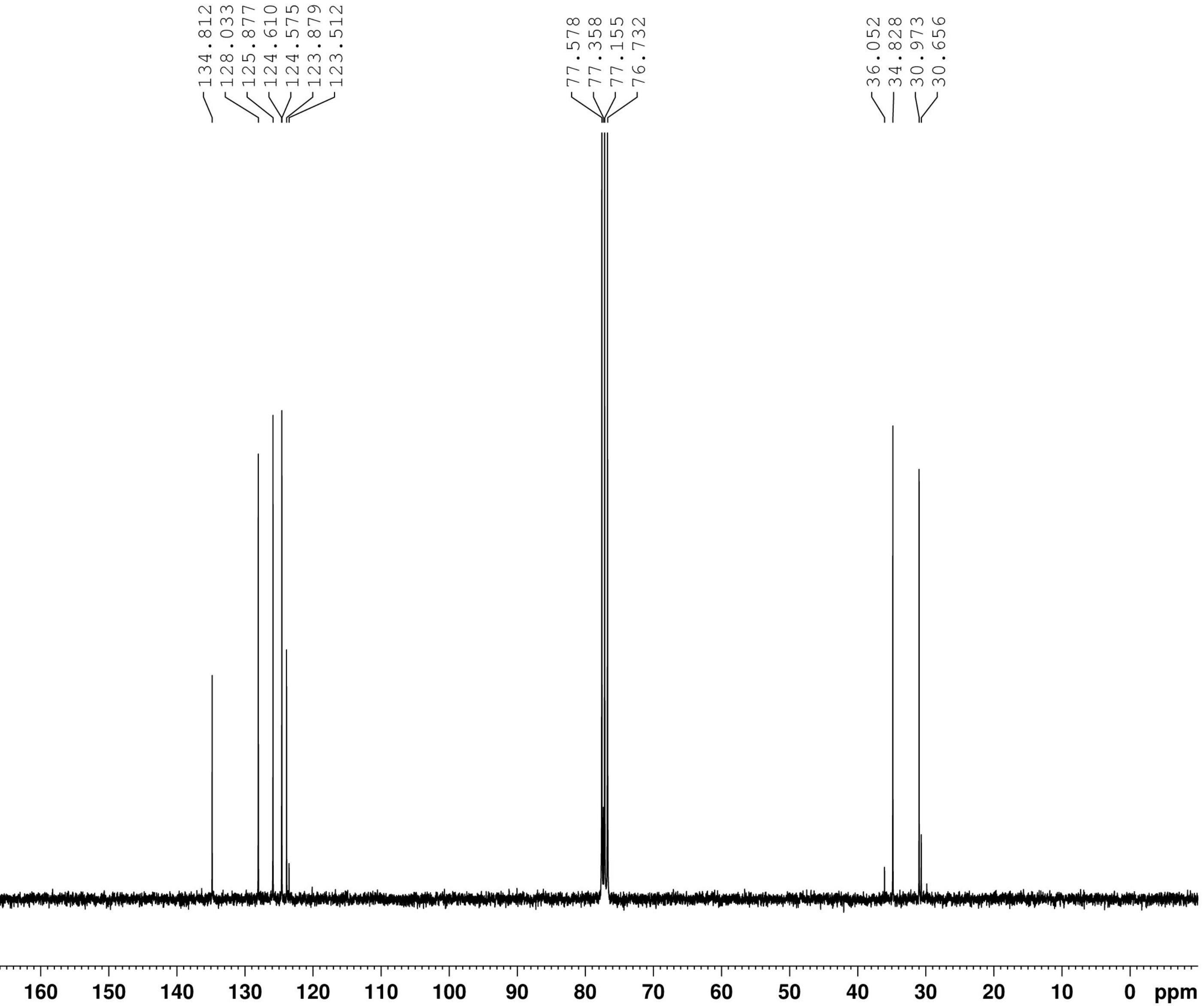

Spectrum S103. ^{1}H NMR spectrum of Table S19, entry 1 (300 MHz, $CDCl_3$, 298 K).

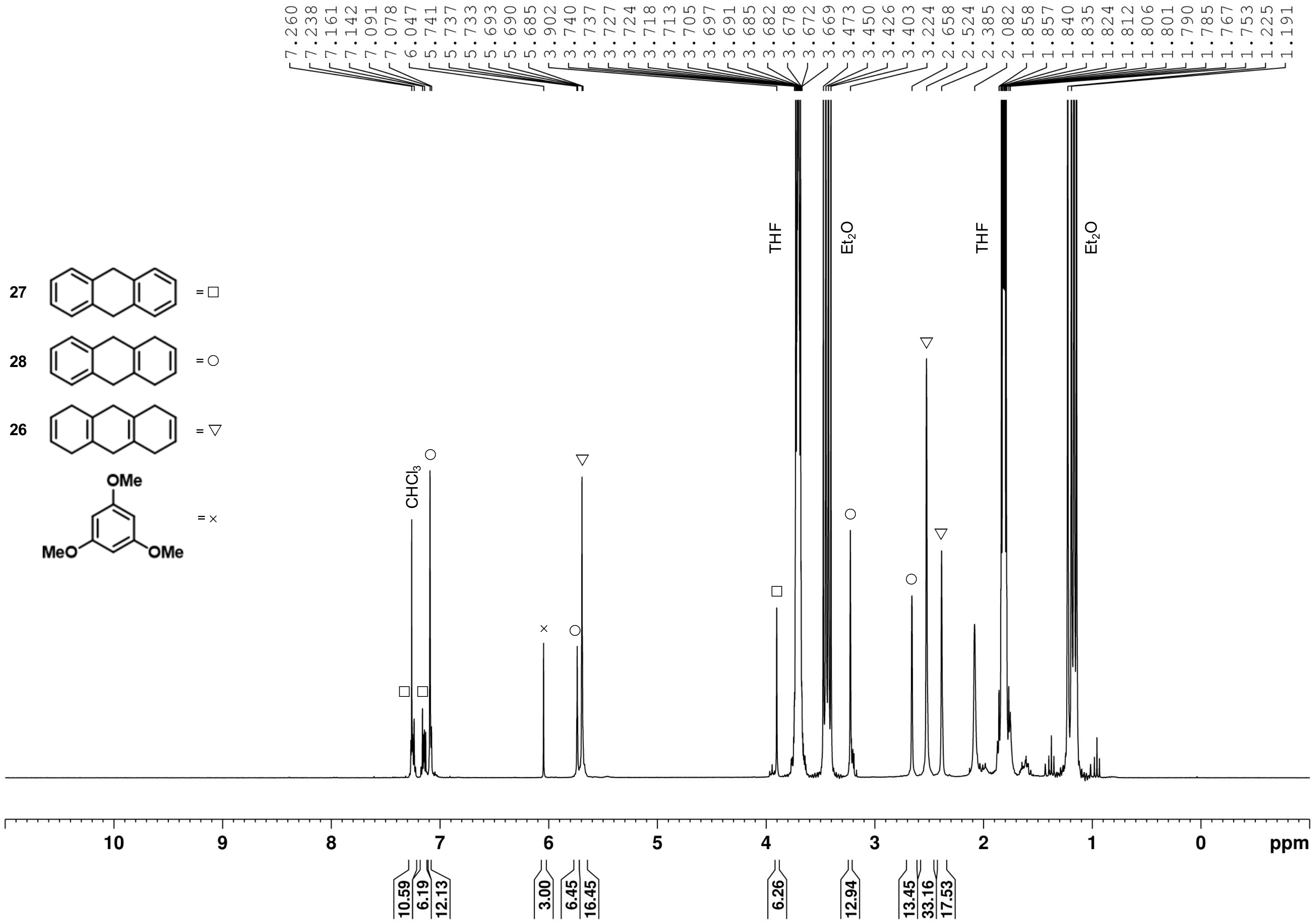

Spectrum S104. ^{1}H NMR spectrum of Table S19, entry 2 (300 MHz, $CDCl_3$, 298 K).

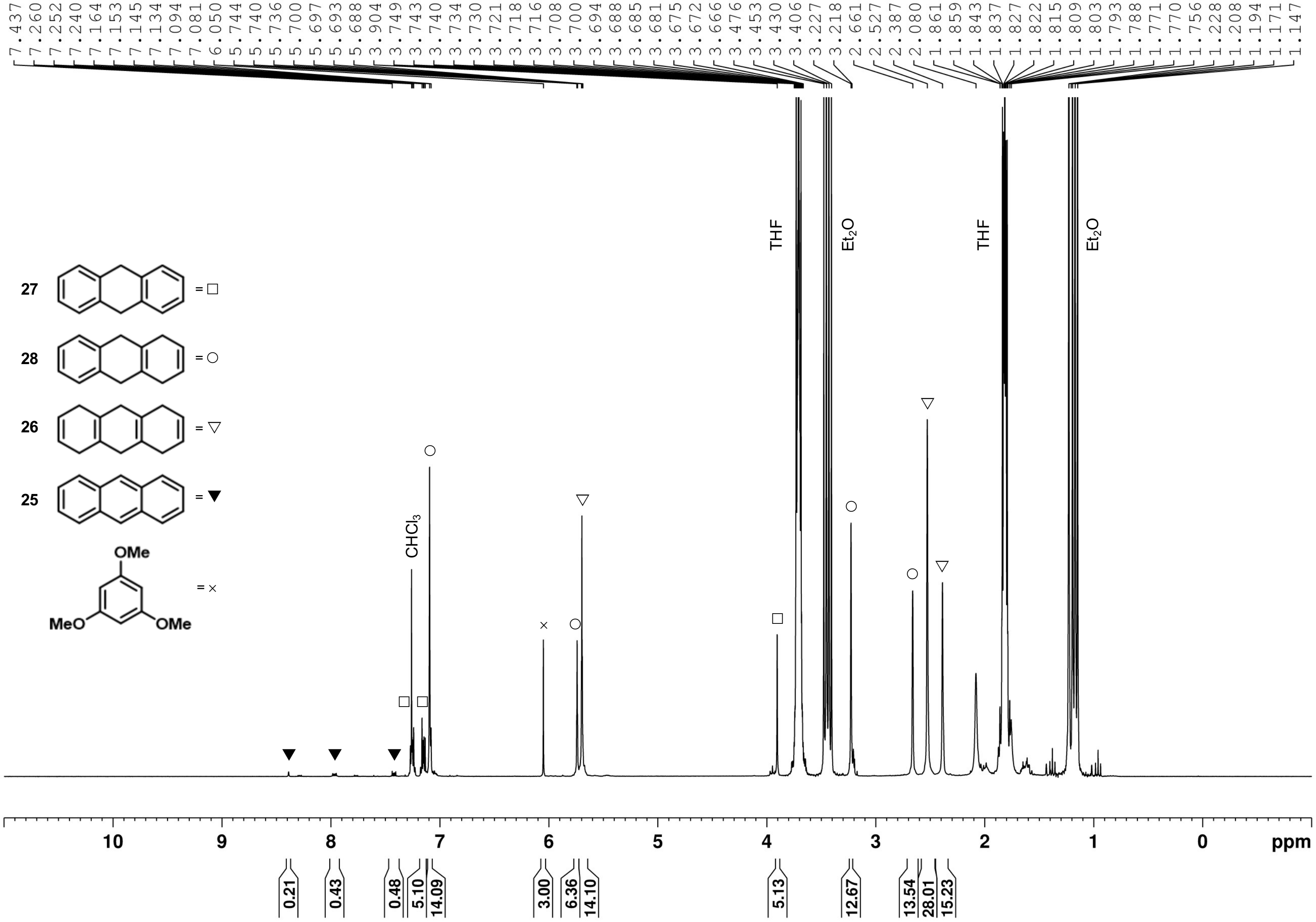

Spectrum S105. ^{1}H NMR spectrum of Table S19, entry 3 (300 MHz, $CDCl_3$, 298 K).

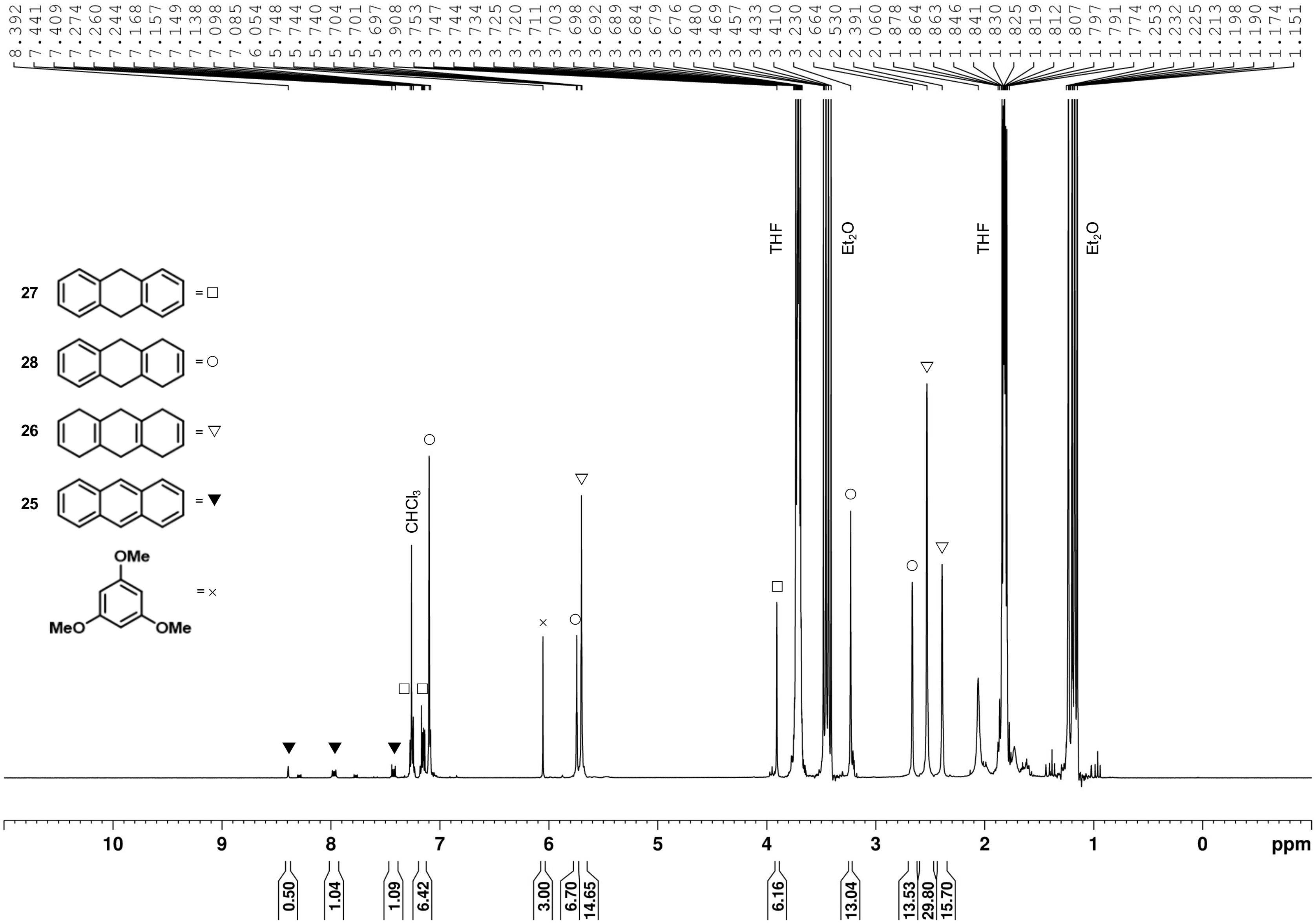

Spectrum S106. ^{1}H NMR spectrum of Table S20, entry 1 (300 MHz, $CDCl_3$, 298 K).

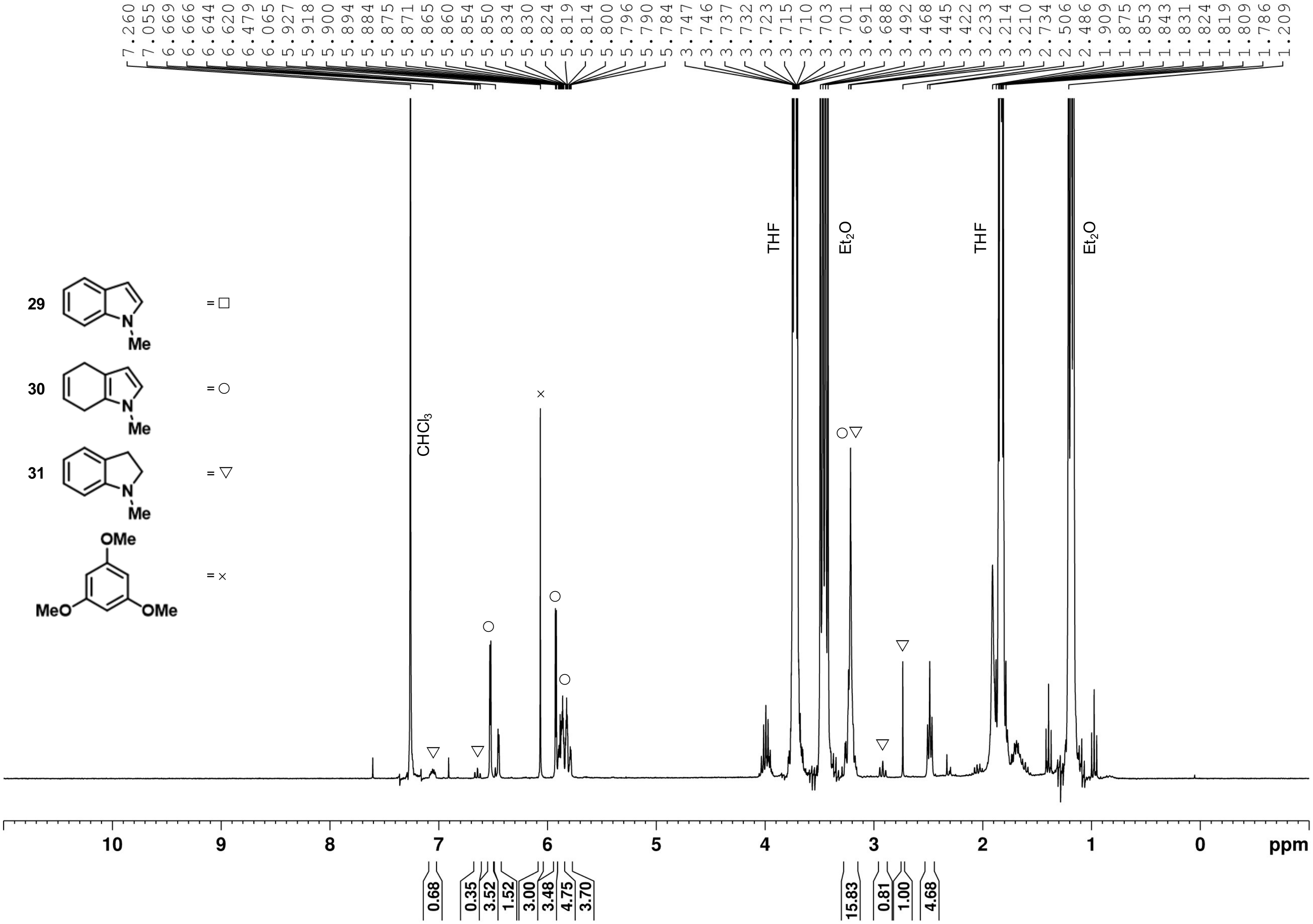

Spectrum S107. ^{1}H NMR spectrum of Table S20, entry 2 (300 MHz, $CDCl_3$, 298 K).

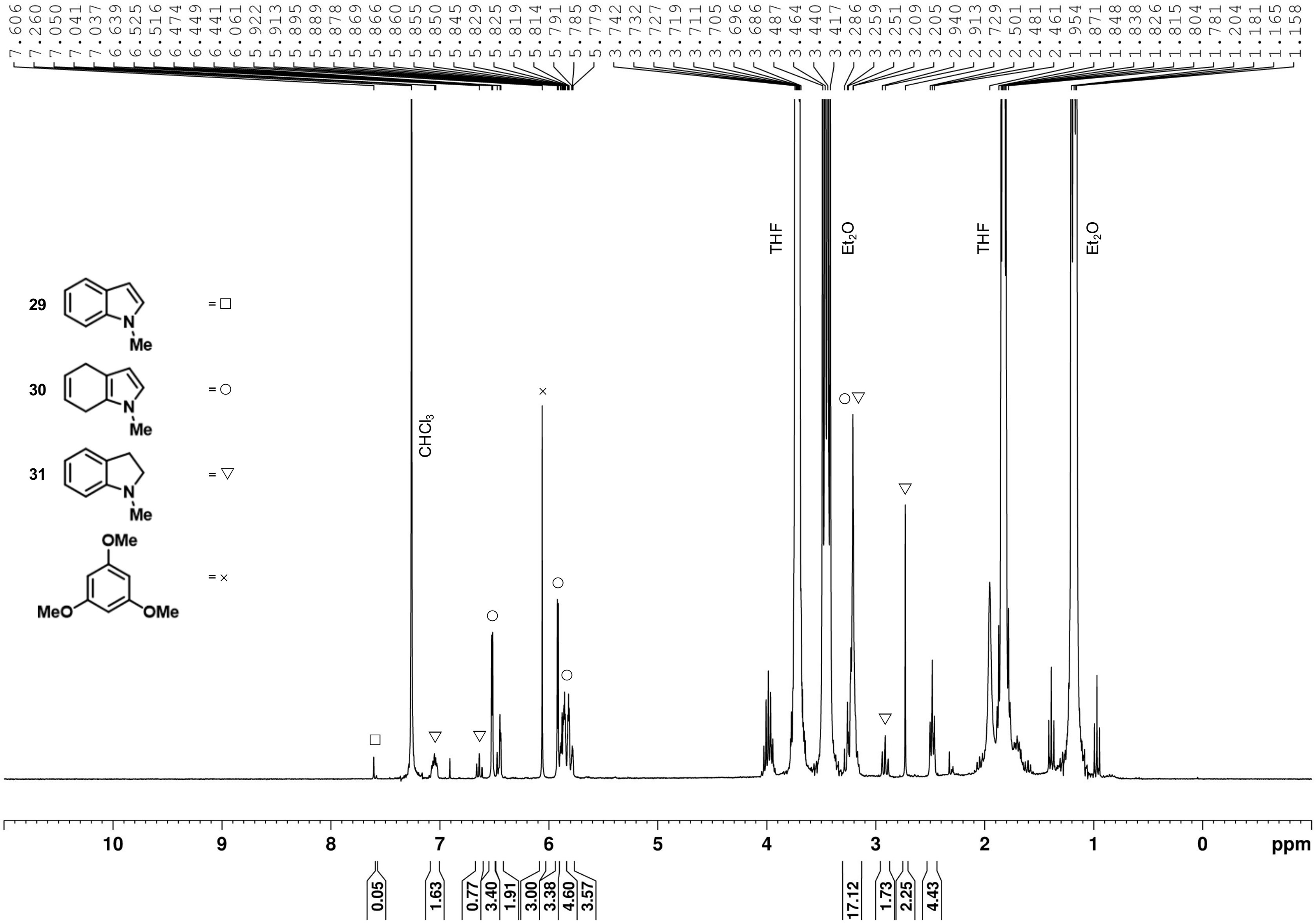

Spectrum S108. ^{1}H NMR spectrum of Table S20, entry 3 (300 MHz, $CDCl_3$, 298 K).

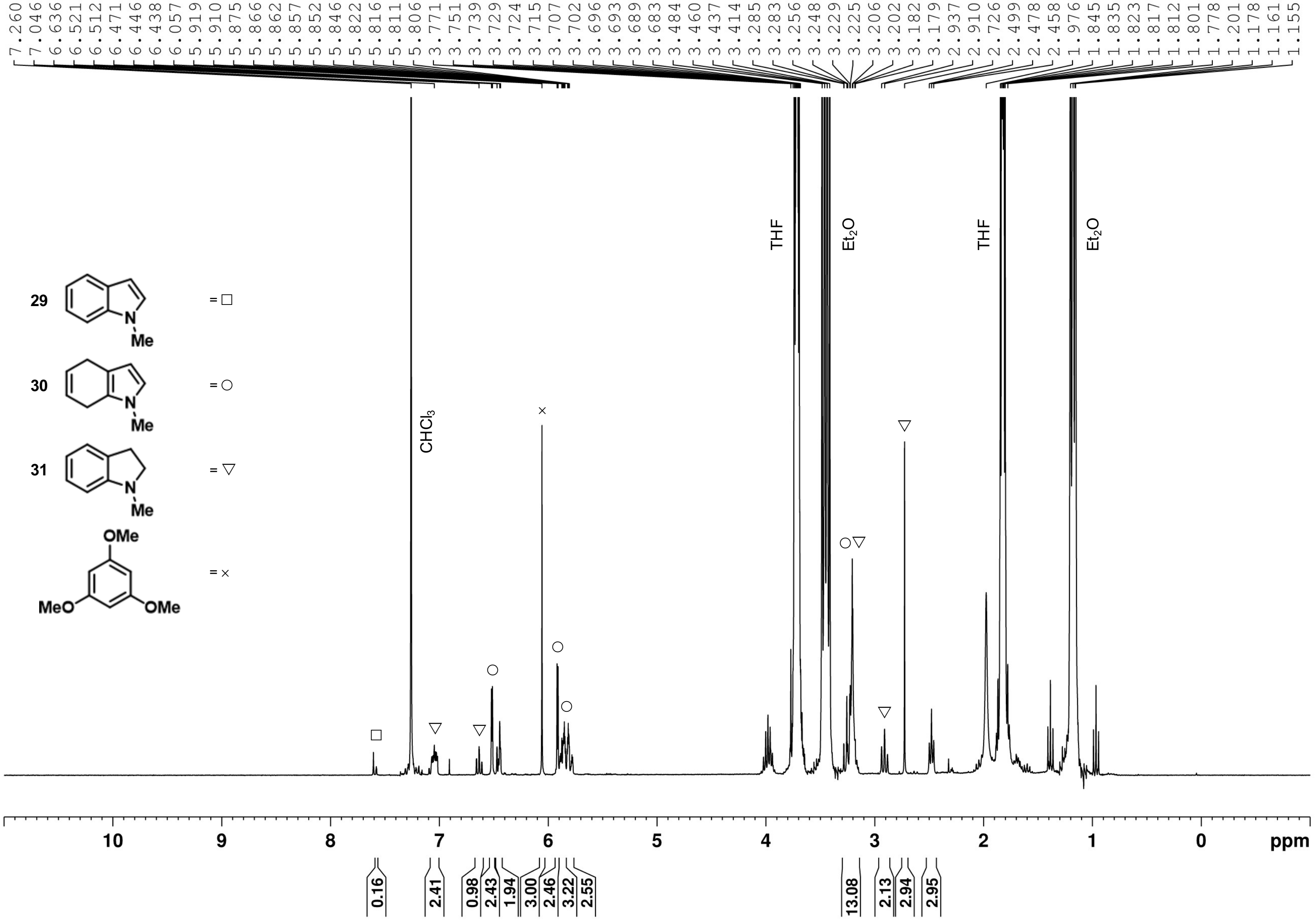

Spectrum S109. ^1H NMR spectrum of Table S21, entry 1 (300 MHz, $CDCl_3$, 298 K).

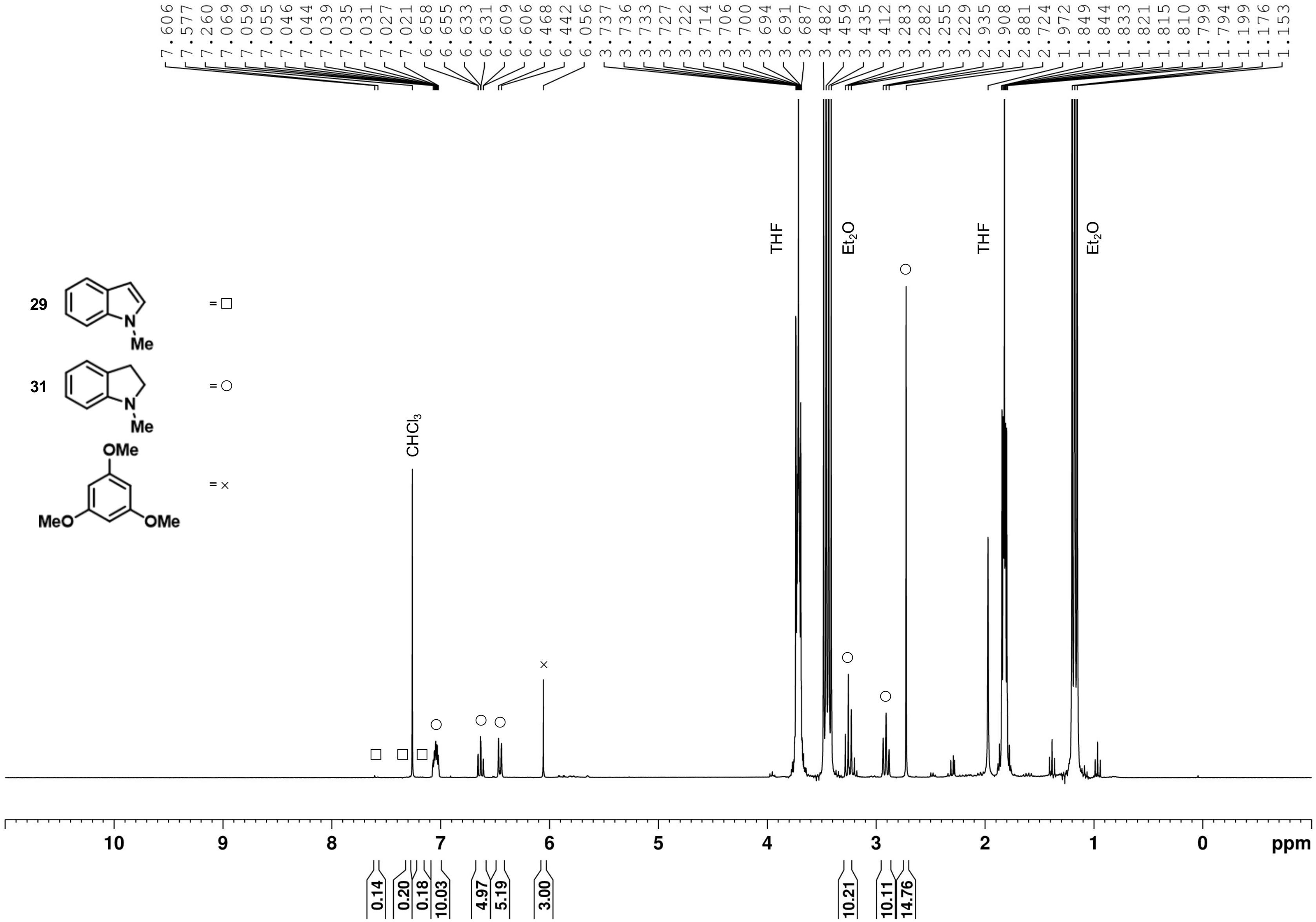

Spectrum S110. ^{1}H NMR spectrum of Table S21, entry 2 (300 MHz, $CDCl_3$, 298 K).

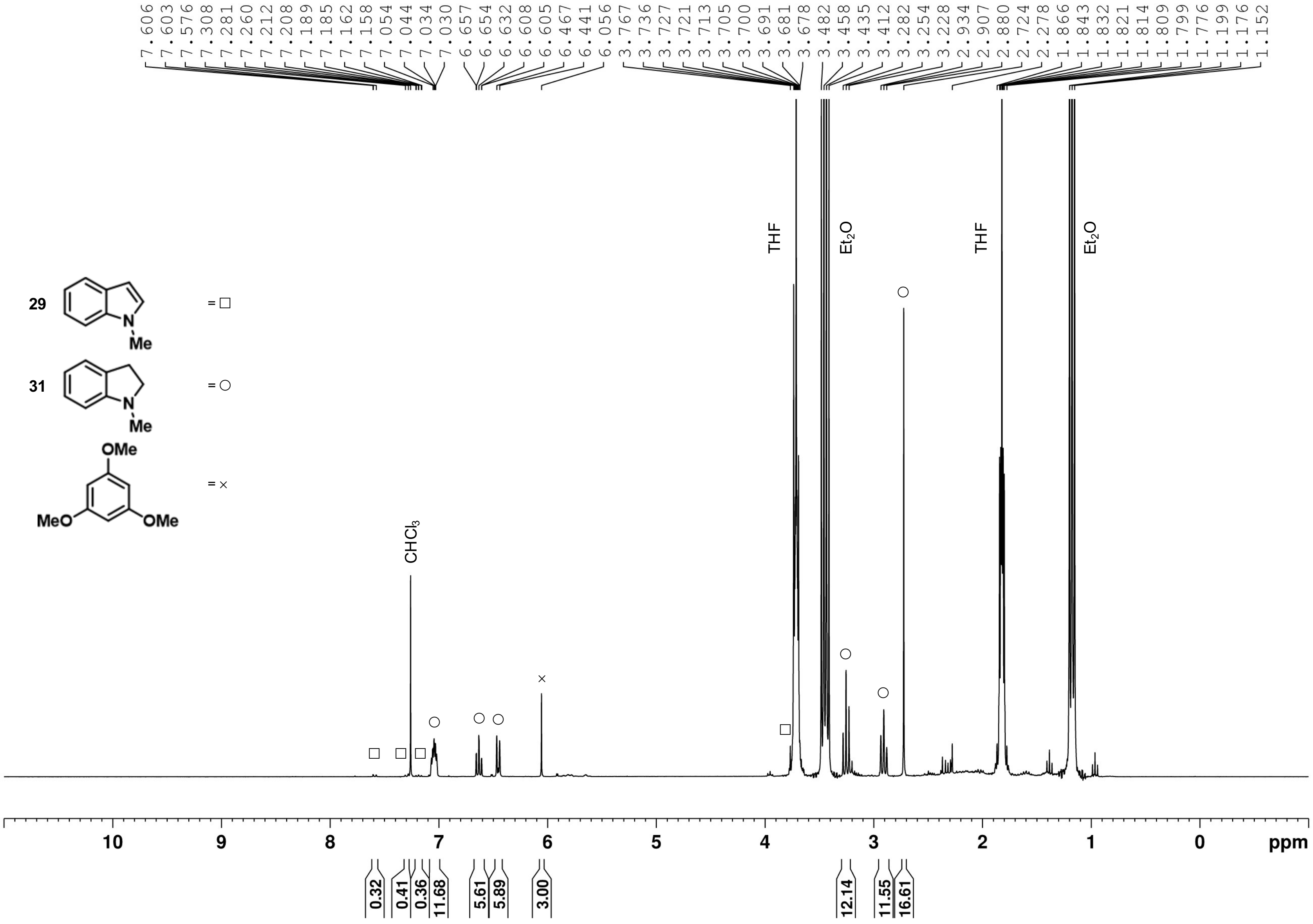

Spectrum S111. ^{1}H NMR spectrum of Table S21, entry 3 (300 MHz, $CDCl_3$, 298 K).

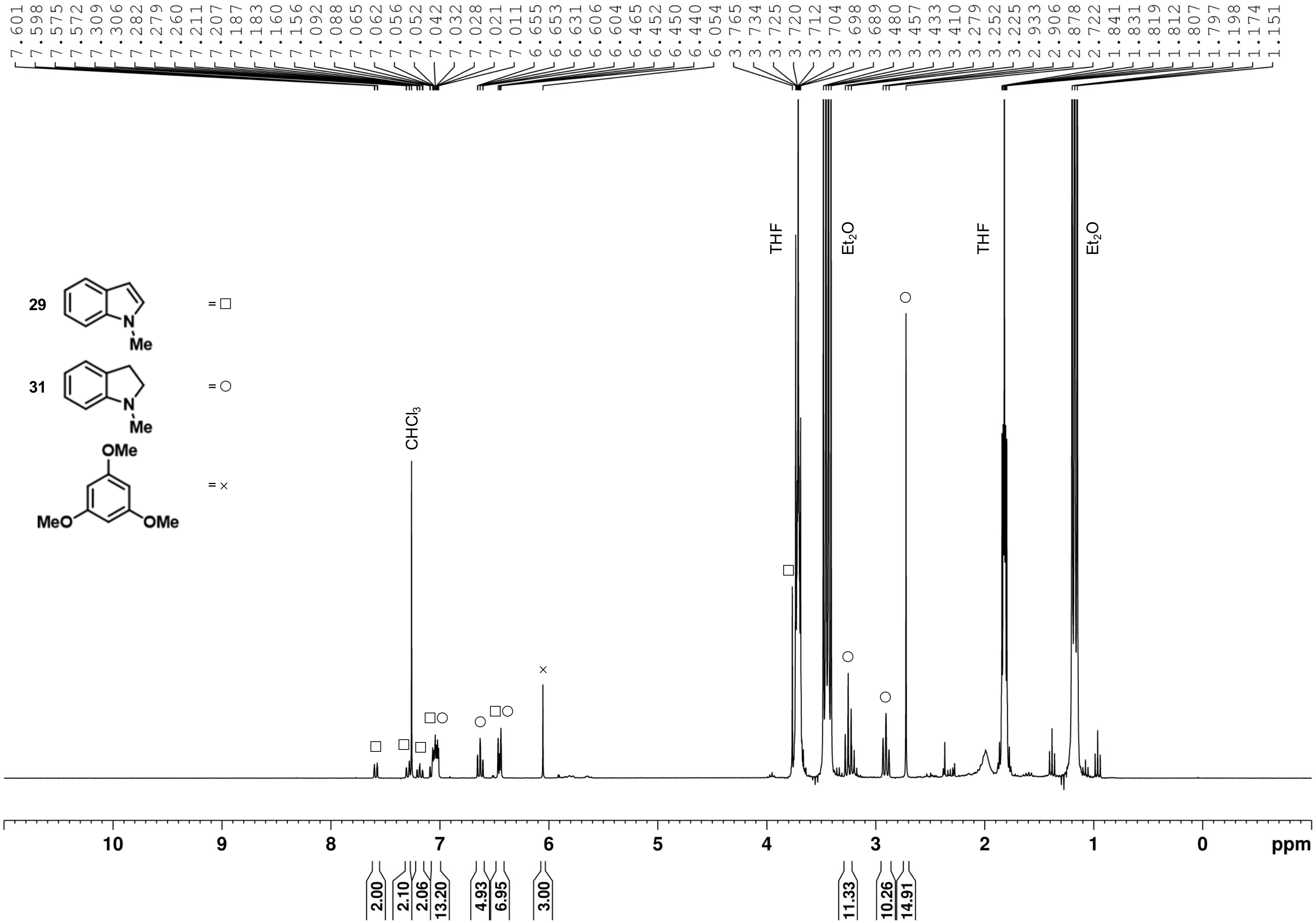

Spectrum S112. ^1H NMR spectrum of Table S22, entry 1 (300 MHz, $CDCl_3$, 298 K).

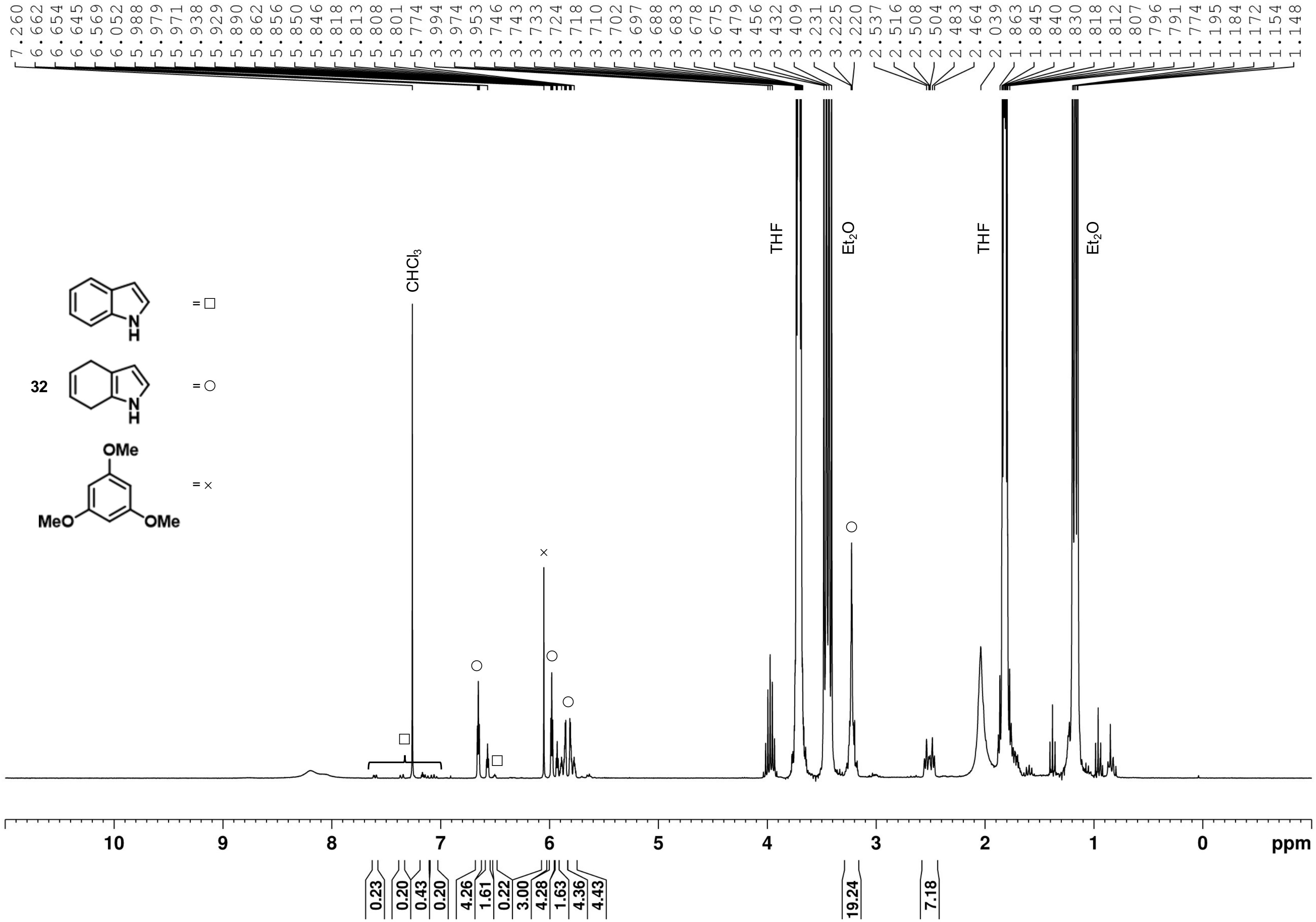

Spectrum S113. ^{1}H NMR spectrum of Table S22, entry 2 (300 MHz, $CDCl_3$, 298 K).

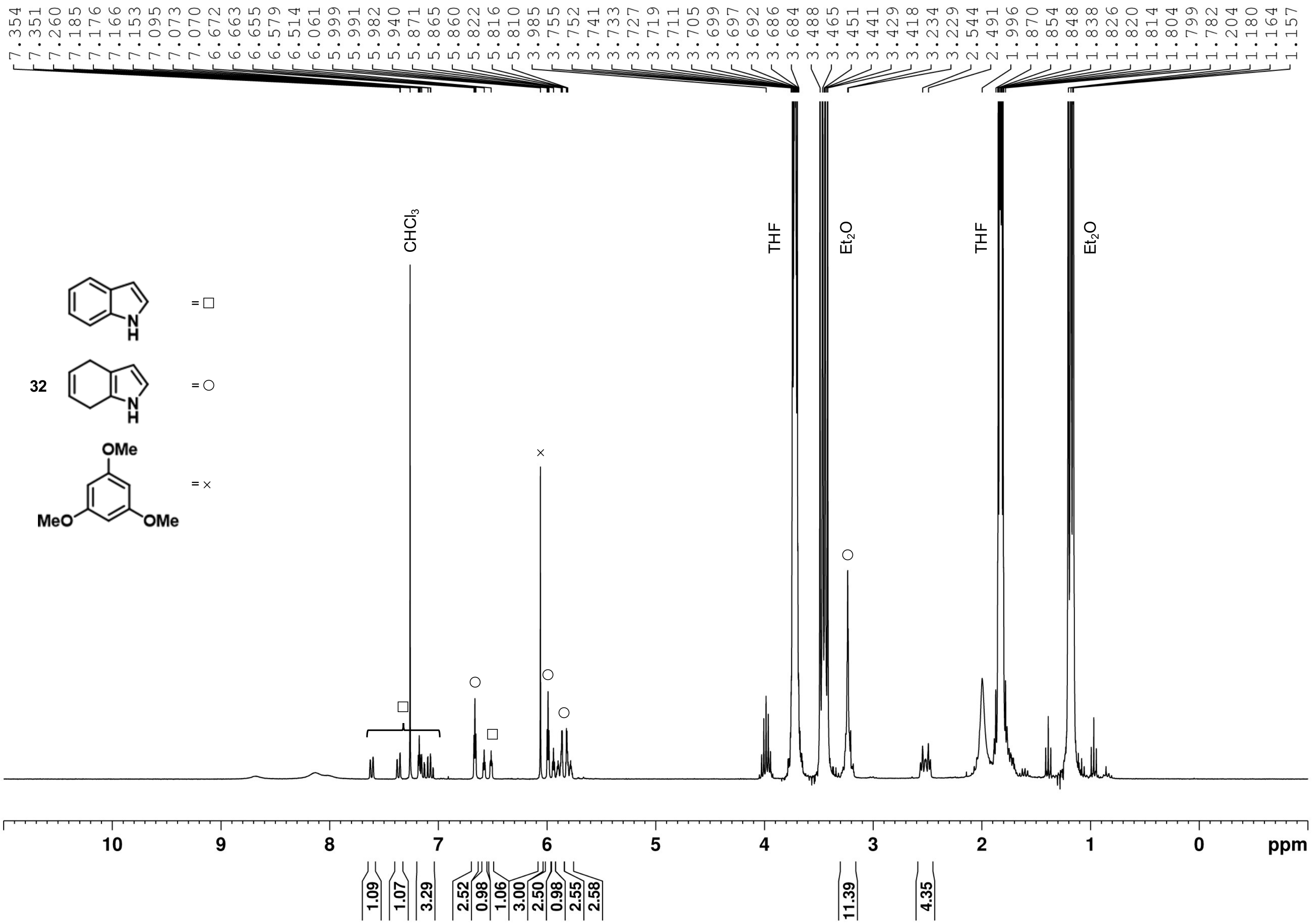

Spectrum S114. ^{1}H NMR spectrum of Table S22, entry 3 (300 MHz, $CDCl_3$, 298 K).

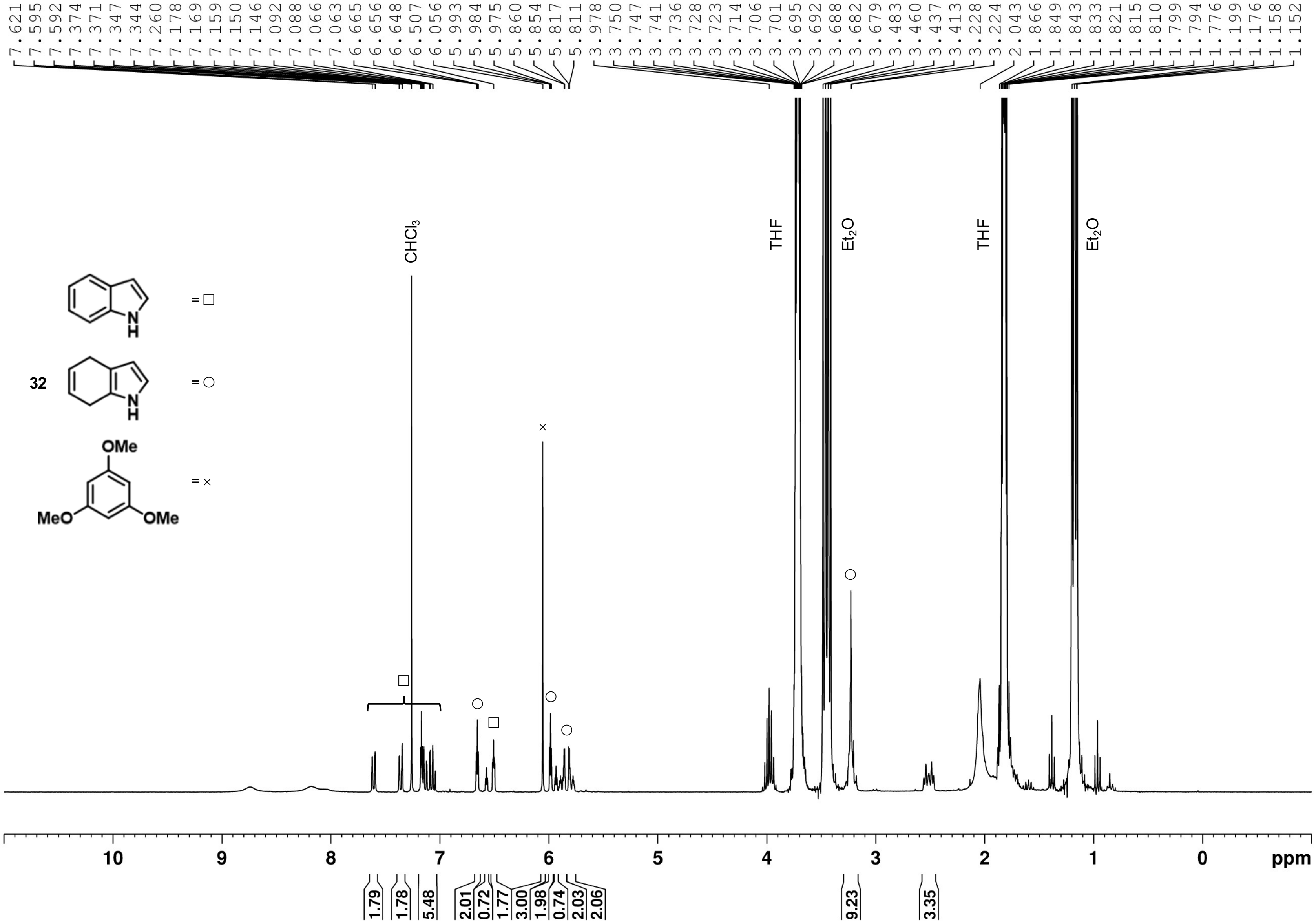

Spectrum S115. ^{1}H NMR spectrum of Table S23, entry 1 (300 MHz, $CDCl_3$ + 1% CD_3OD, 298 K).

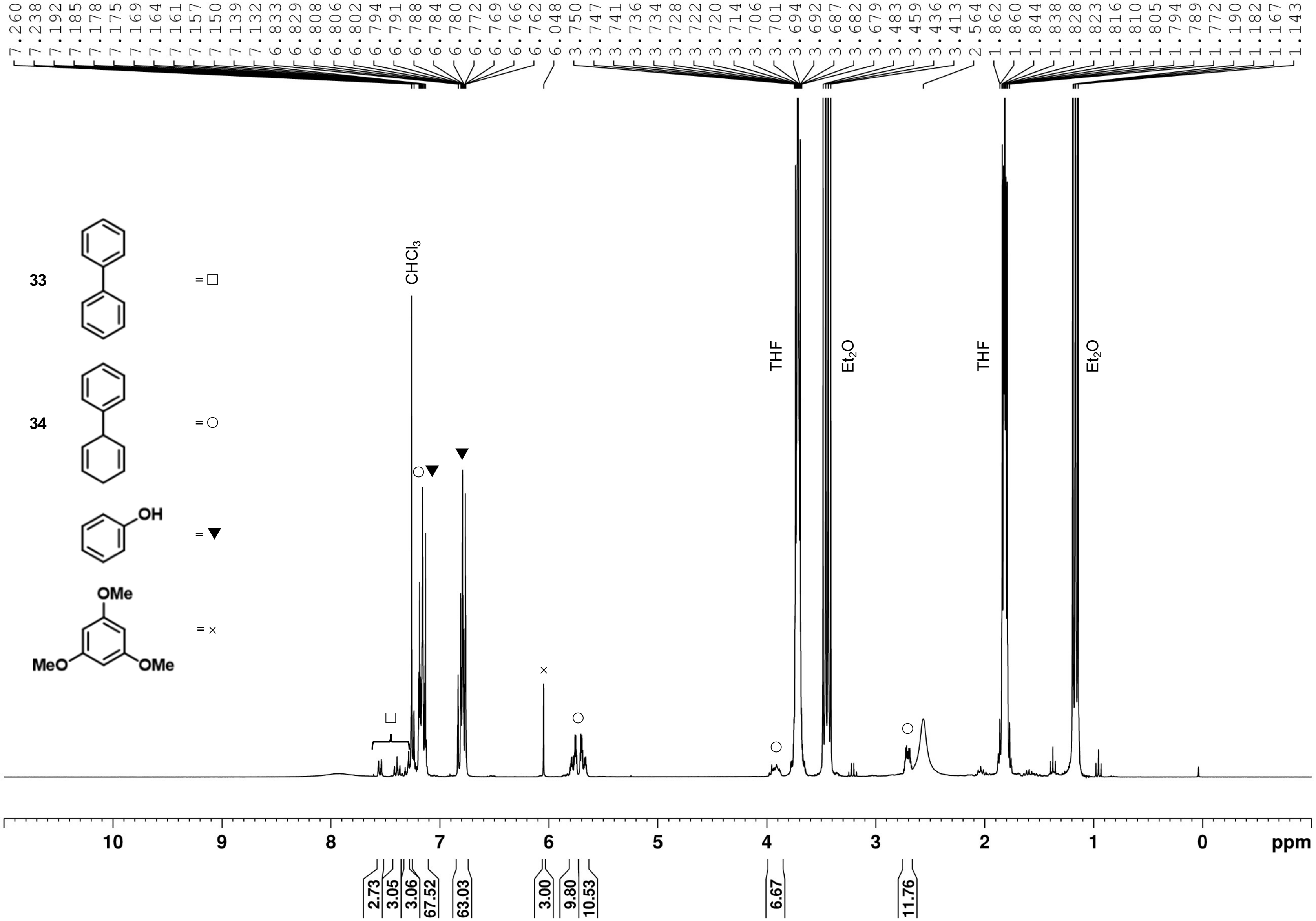

Spectrum S116. ^{1}H NMR spectrum of Table S23, entry 2 (300 MHz, $CDCl_3$ + 1% CD_3OD, 298 K).

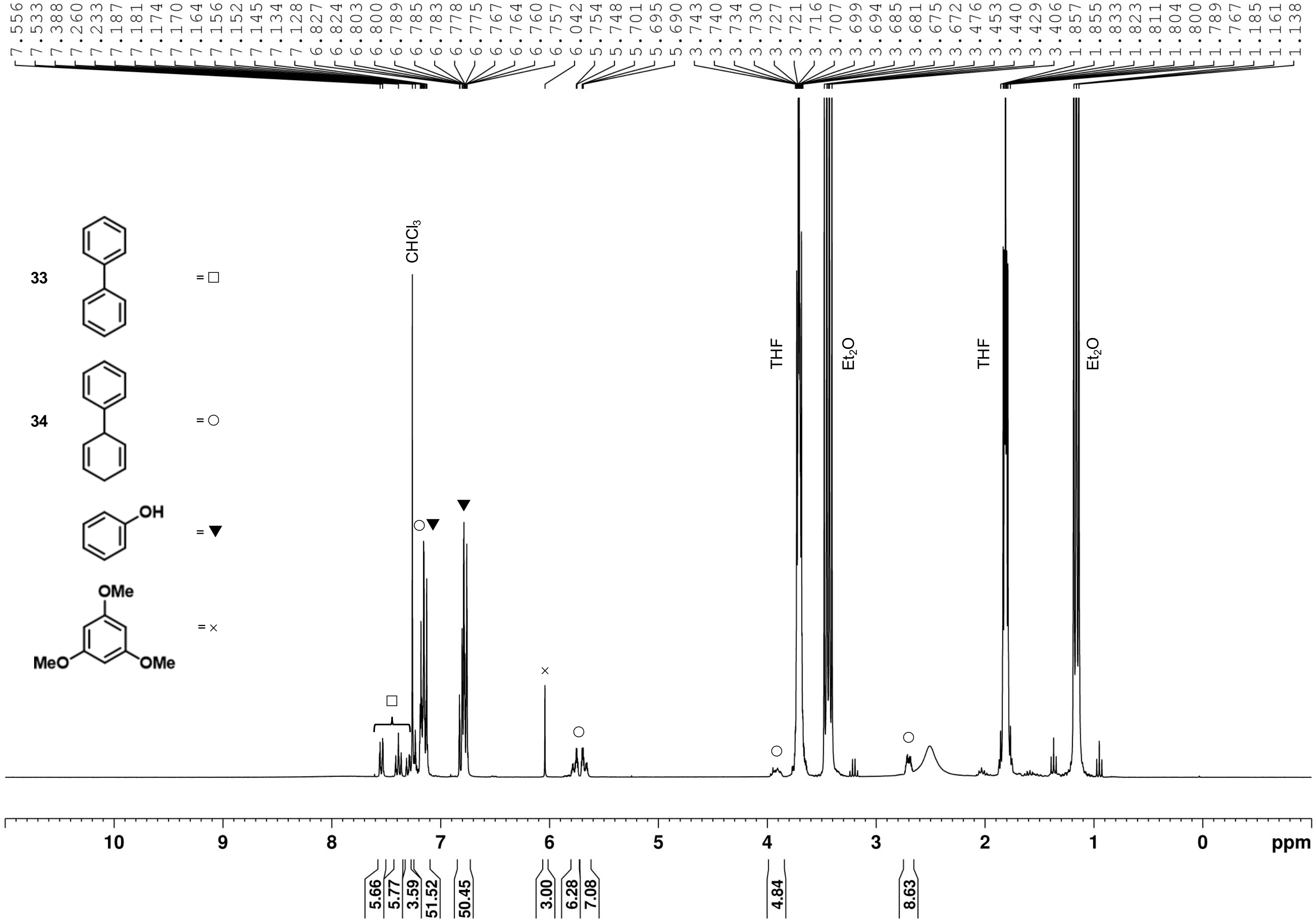

Spectrum S117. ^{1}H NMR spectrum of Table S23, entry 3 (300 MHz, $CDCl_3$ + 1% CD_3OD, 298 K).

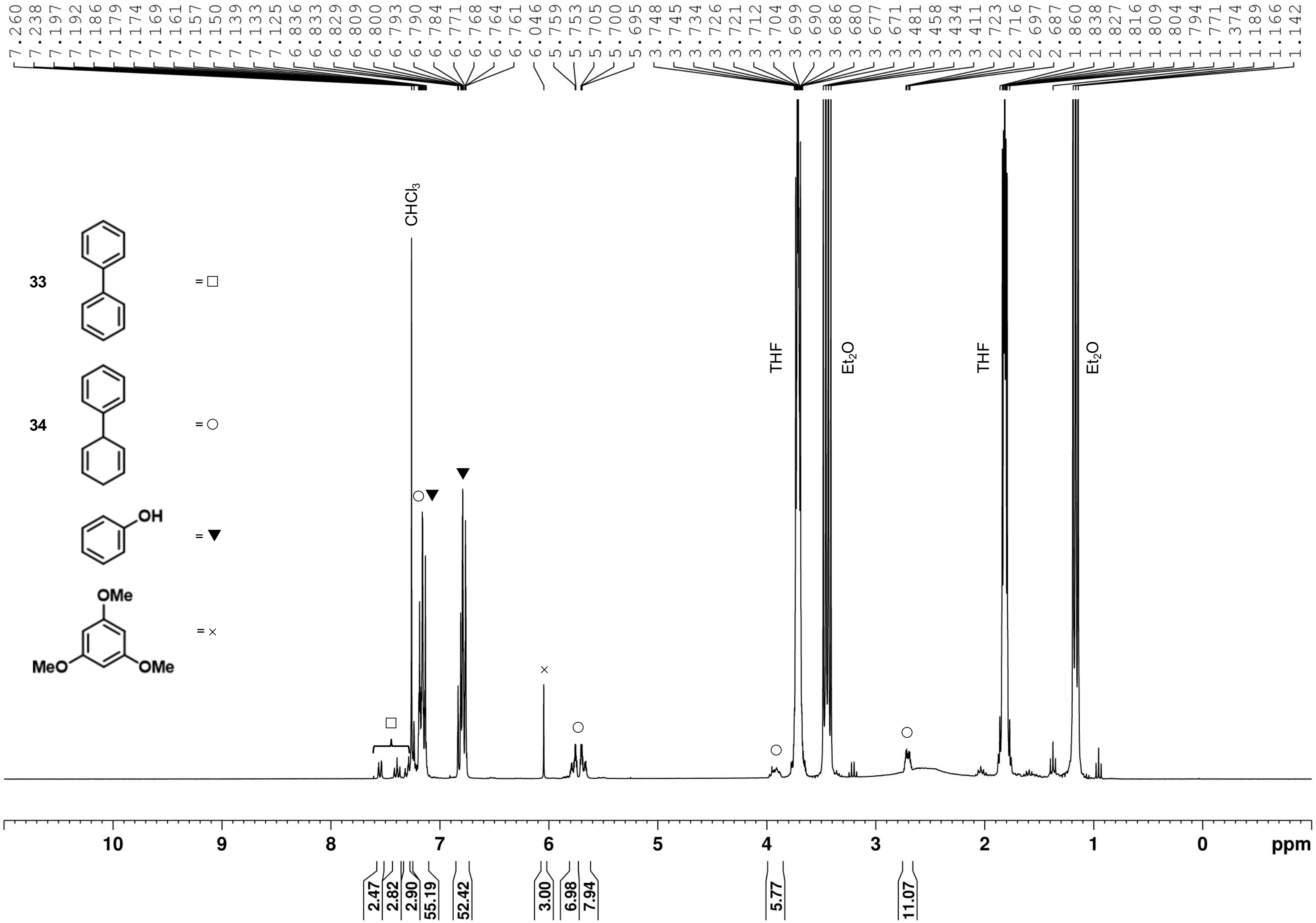

Spectrum S118. ^{1}H NMR spectrum of 1',4'-dihydro-[1,1'-biphenyl]-3-ol (**36**) (300 MHz, $CDCl_3$, 298 K).

36

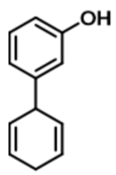

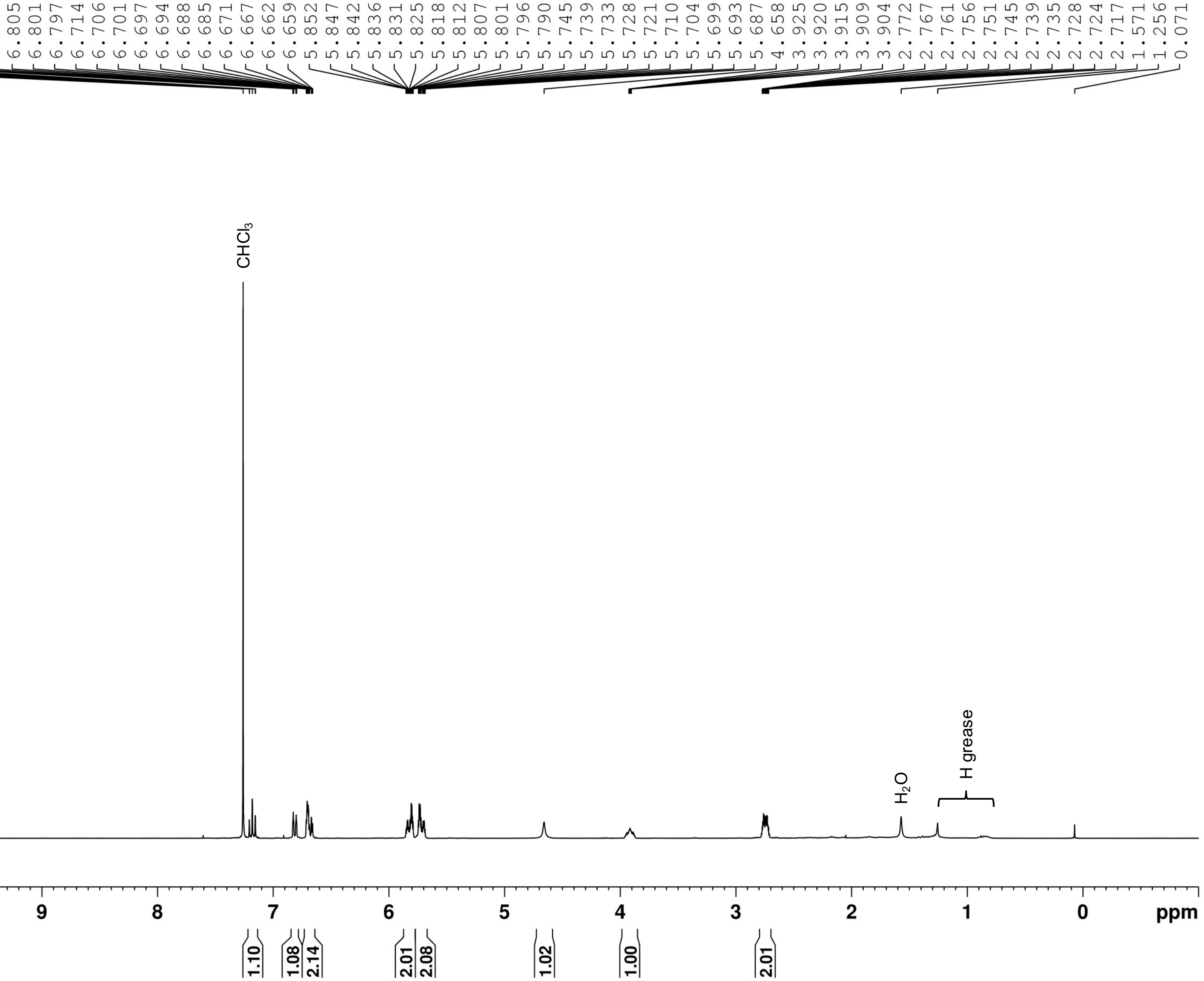

Continuation of Spectrum S118.

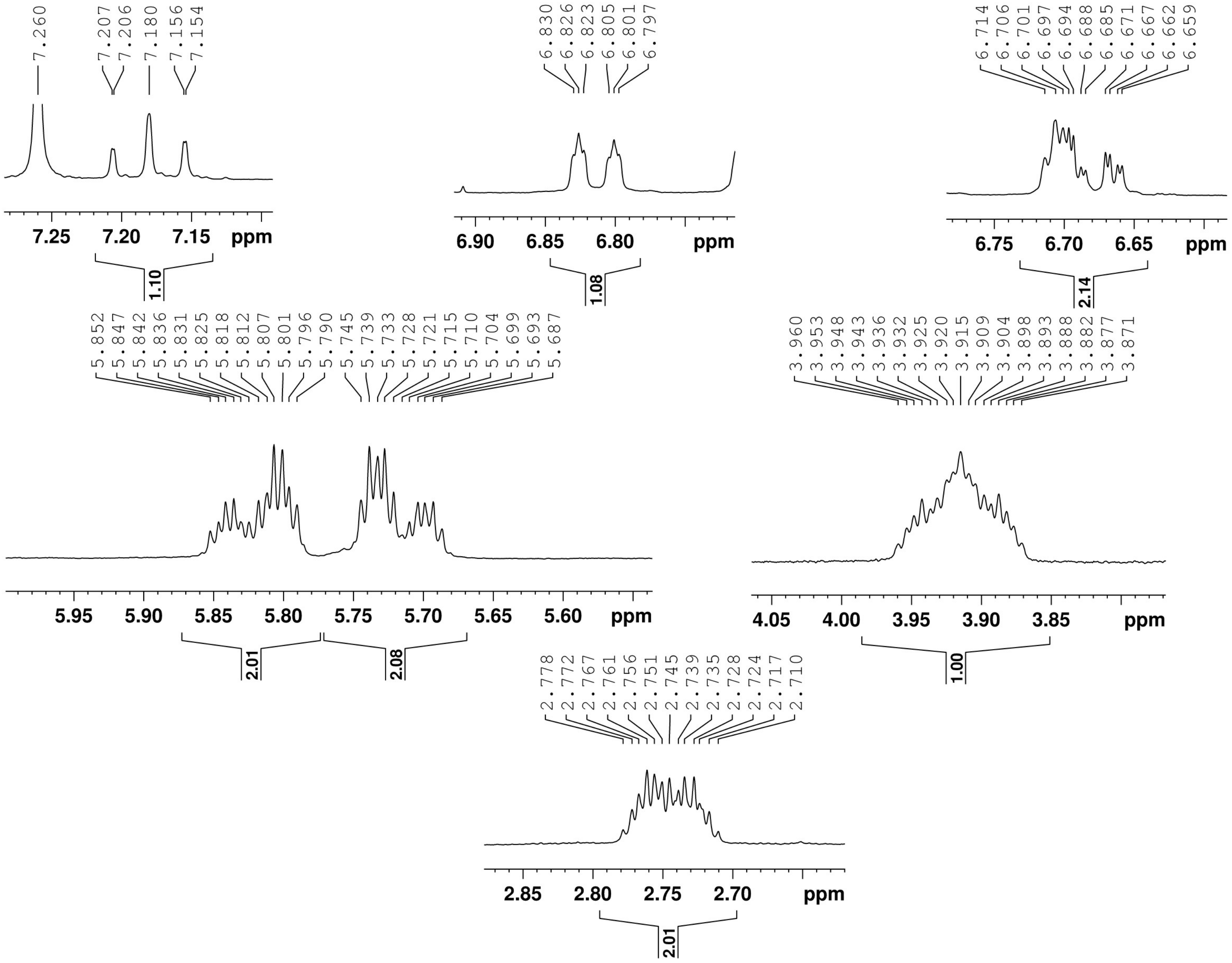

Spectrum S119. ^{13}C NMR spectrum of 1',4'-dihydro-[1,1'-biphenyl]-3-ol (**36**) (300 MHz, $CDCl_3$, 298 K).

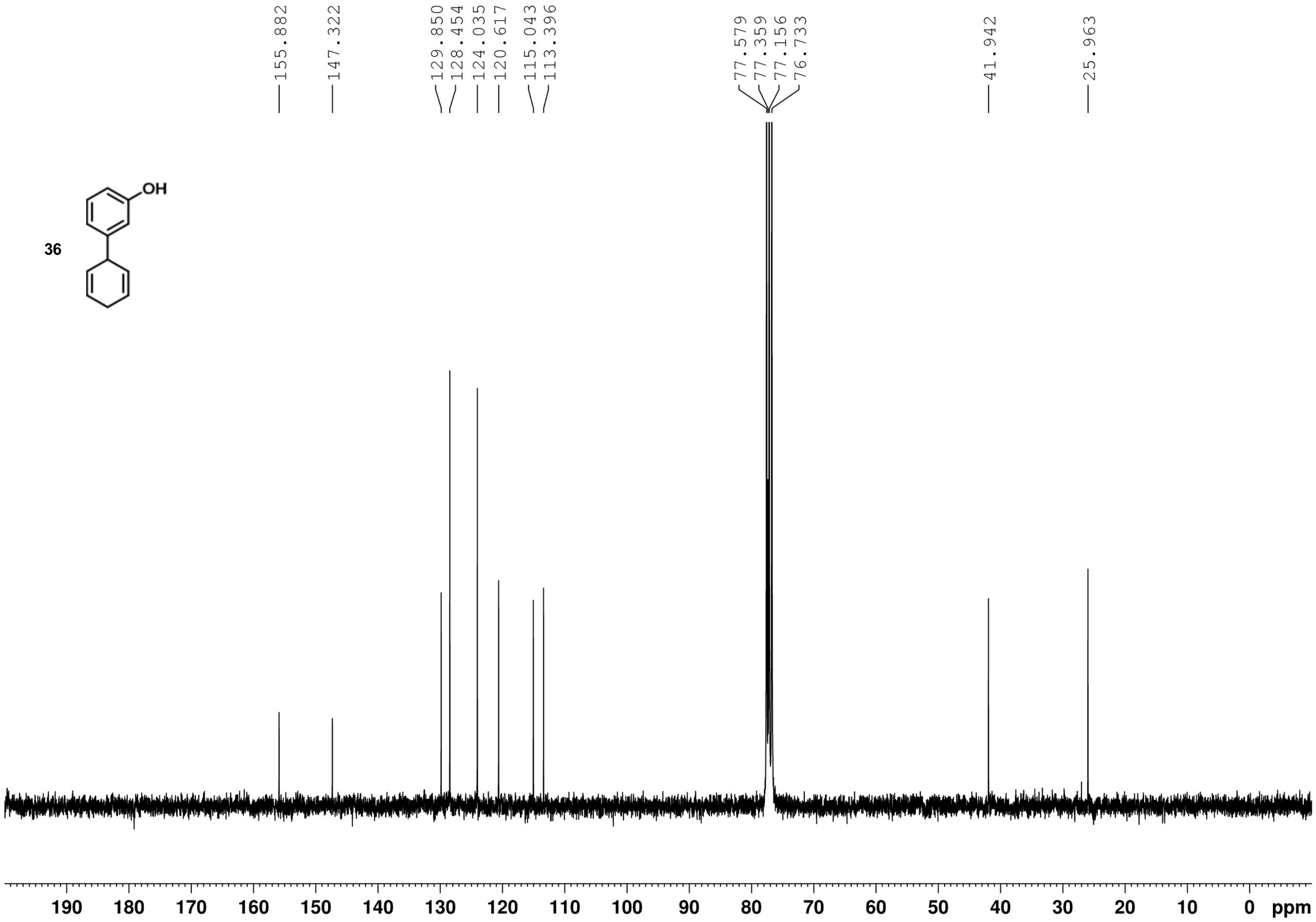

Spectrum S120. ^{1}H NMR spectrum of Table S24, entry 1 (300 MHz, $CDCl_3$, 298 K).

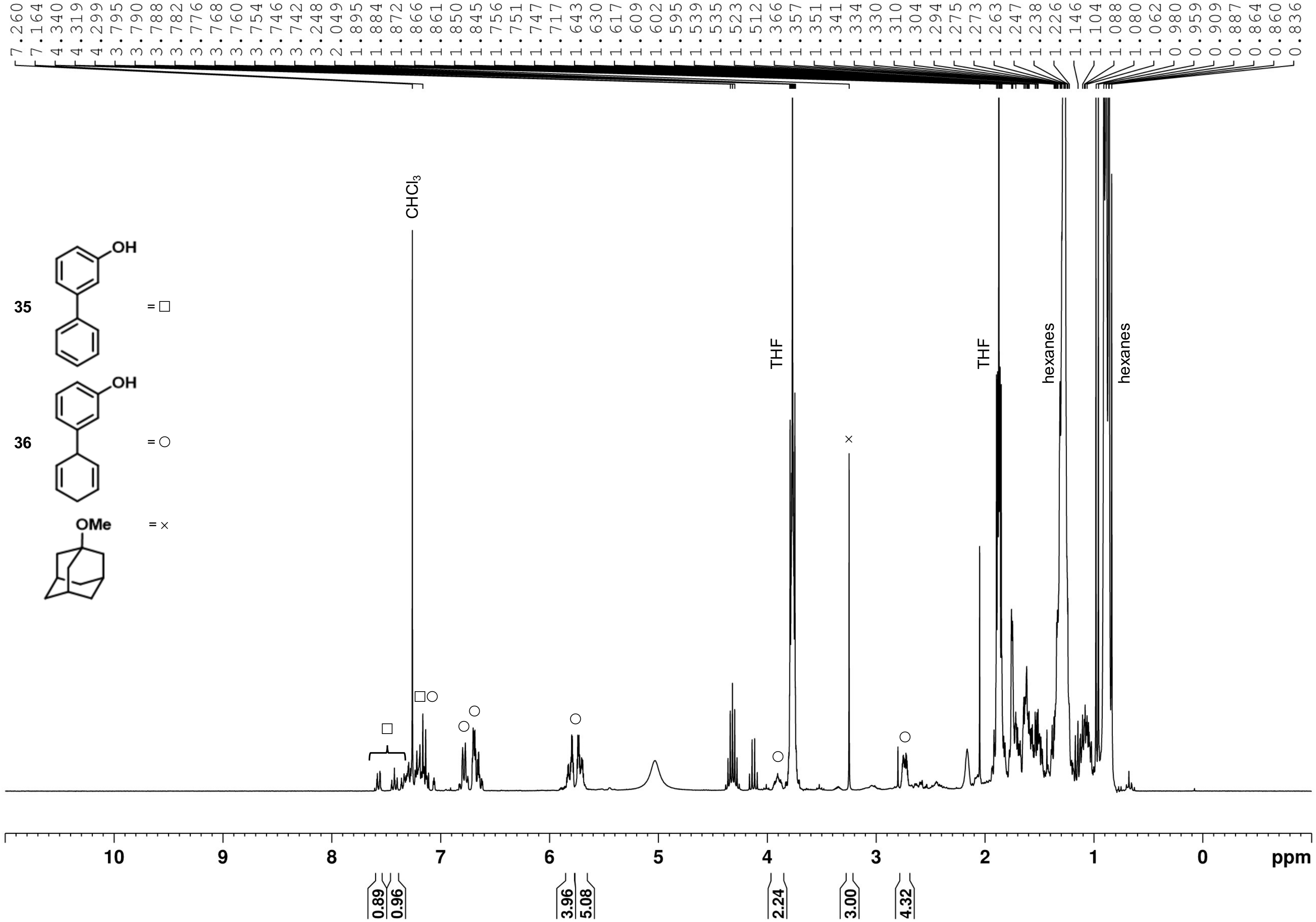

Spectrum S121. ^{1}H NMR spectrum of Table S24, entry 2 (300 MHz, $CDCl_3$, 298 K).

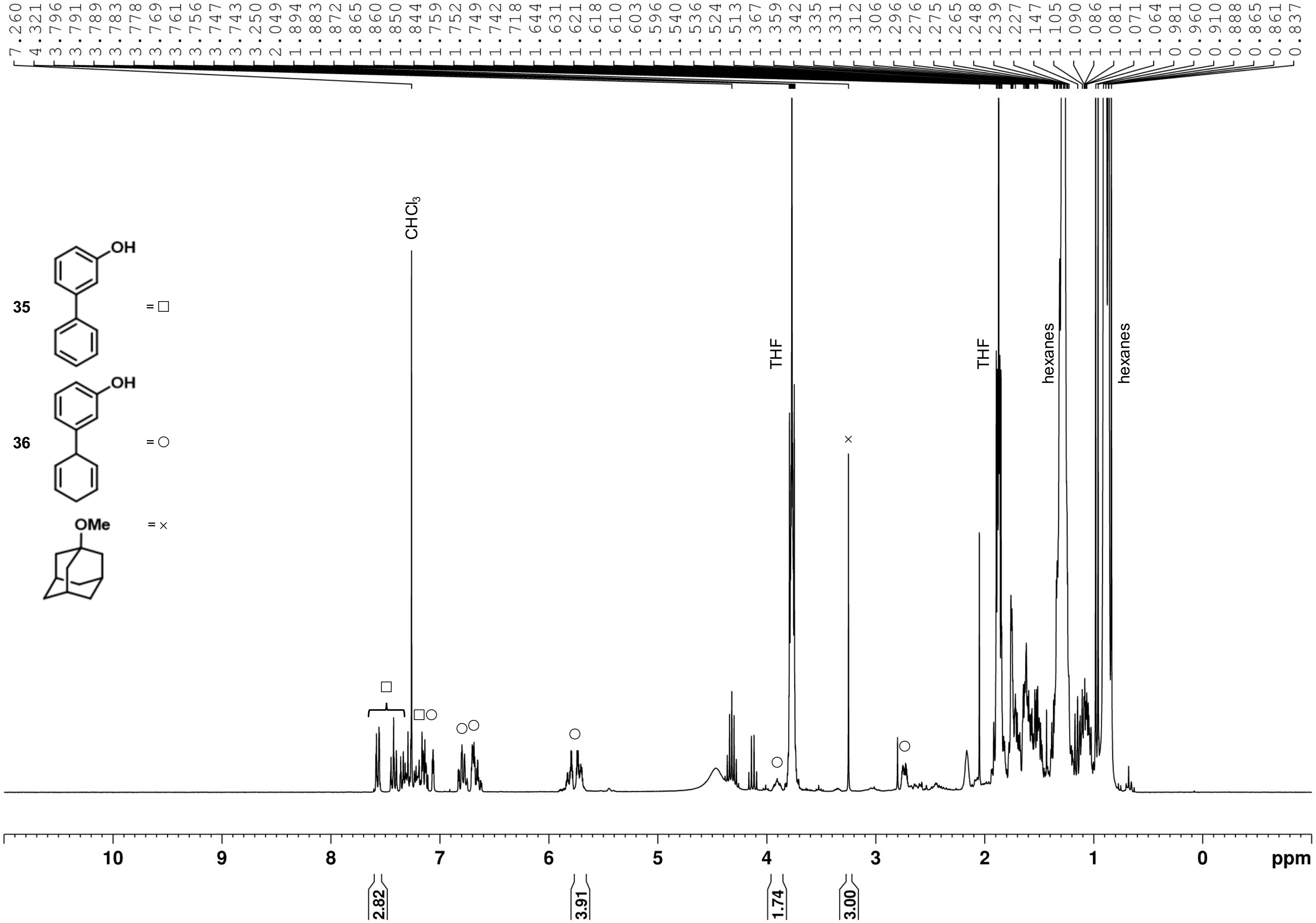

Spectrum S122. ^{1}H NMR spectrum of Table S24, entry 3 (300 MHz, $CDCl_3$, 298 K).

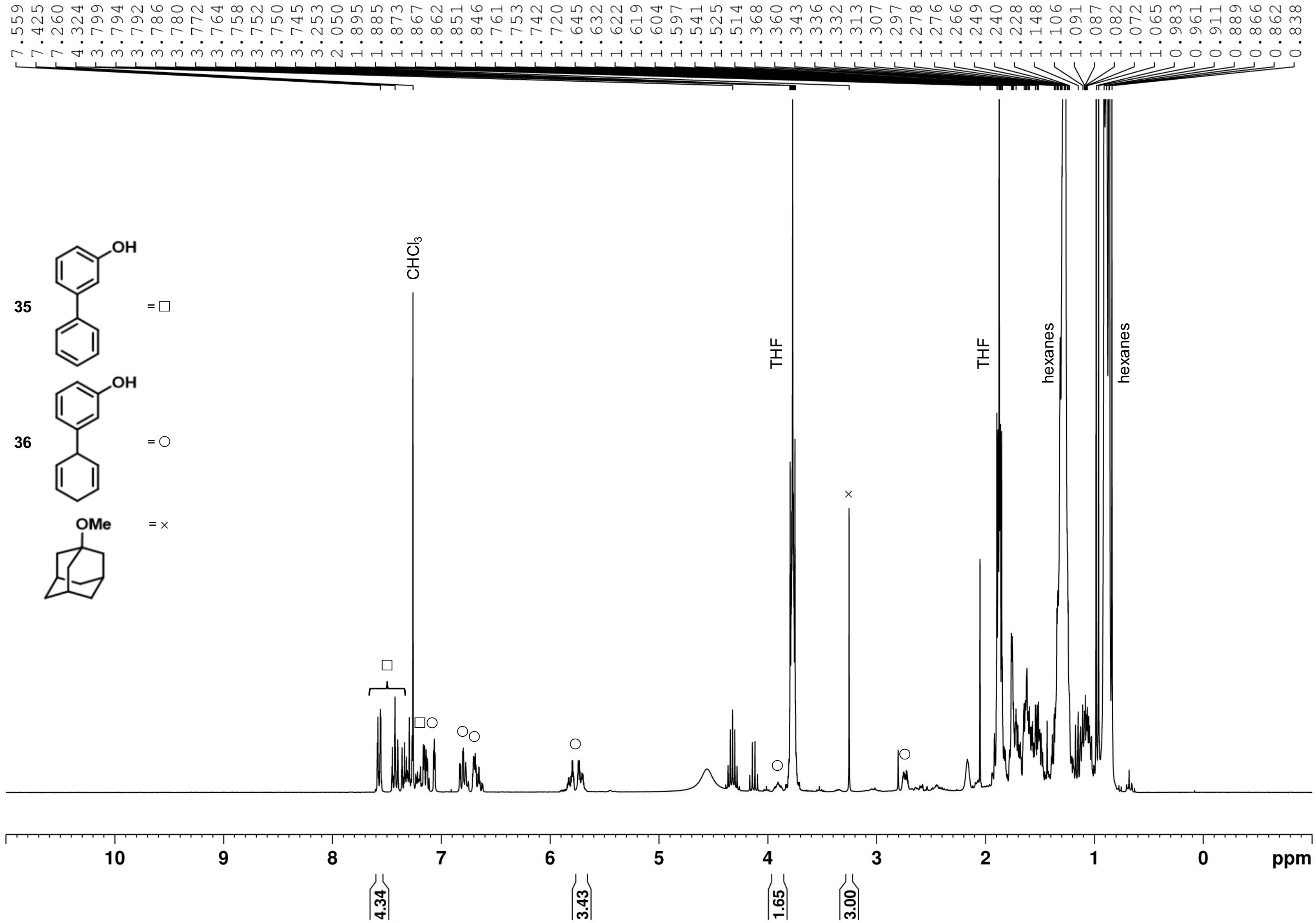

Citted References

(1) Thompson, J. K.; Kleinberg, J. The oxidation of lithium and the alkaline earth metals in liquid ammonia. *J. Am. Chem. Soc.* **1951**, *73* (3), 1243–1245. DOI: 10.1021/ja01147a109.

(2) Hayyan, M.; Hashim, M. A.; AlNashef, I. M. Superoxide ion: Generation and chemical implications. *Chem. Rev.* **2016**, *116* (5), 3029–3085. DOI: 10.1021/acs.chemrev.5b00407.

(3) Zhang, X.; Guo, L.; Gan, L.; Zhang, Y.; Wang, J.; Johnson, L. R.; Bruce, P. G.; Peng, Z. LiO_2: Cryosynthesis and chemical/electrochemical reactivities. *J. Phys. Chem. Lett.* **2017**, *8* (10), 2334–2338. DOI: 10.1021/acs.jpclett.7b00680.

(4) Renaud, P.; Fox, M. A. Electrochemical behavior of lithium dialkylamides: the effect of aggregation. *J. Am. Chem. Soc.* **1988**, *110* (17), 5702–5705. DOI: 10.1021/ja00225a020.

(5) Beatty, J. W.; Stephenson, C. R. J. Amine functionalization via oxidative photoredox catalysis: Methodology development and complex molecule synthesis. *Acc. Chem. Res.* **2015**, *48* (5), 1474–1484. DOI: 10.1021/acs.accounts.5b00068.

(6) Roque, J. B.; Sarpong, R.; Musaev, D. G. Key mechanistic features of the silver(I)-mediated deconstructive fluorination of cyclic amines: Multistate reactivity versus single-electron transfer. *J. Am. Chem. Soc.* **2021**, *143* (10), 3889–3900. DOI: 10.1021/jacs.0c13061.

(7) Gunasekera, D.; Mahajan, J. P.; Wanzi, Y.; Rodrigo, S.; Liu, W.; Tan, T.; Luo, L. Controlling one- or two-electron oxidation for selective amine functionalization by alternating current frequency. *J. Am. Chem. Soc.* **2022**, *144* (22), 9874–9882. DOI: 10.1021/jacs.2c02605.
